# Identifying risk factors of young-onset dementia and evaluating evidence hierarchy: a meta-analysis and umbrella review

**DOI:** 10.1016/j.tjpad.2025.100467

**Published:** 2026-01-01

**Authors:** Jiayu Zhang, Dandan Yang, Jian Liang, Yin Hu, Liping Rao, Jun Huang, Qijun Wu, Bo Jiang

**Affiliations:** a Center of Clinical Pharmacology, The Second Affiliated Hospital, Zhejiang University School of Medicine, Hangzhou 310009, China; b Department of Clinical Epidemiology, Clinical Research Center, Key Laboratory of Precision Medical Research on Major Chronic Disease, Shengjing Hospital of China Medical University, Shenyang 110004, China

**Keywords:** Young-onset dementia, Prevention, Umbrella review, Meta-analysis

## Abstract

**Background:**

Young-onset dementia (YOD) directly affects the working-age population. The premature onset of dementia intensifies peer caregiving responsibilities and diverts medical and nursing resources. While modifiable risk factors for late-onset dementia have been well established, uncertainty remains regarding the applicability of these findings to YOD. We aim to identify modifiable risk factors for YOD and evaluate the strength of evidence.

**Methods:**

We searched PubMed, Embase, Web of Science, and Ovid Medline from inception to 22 May 2025 for epidemiological studies on non-genetic risk factors for YOD. We used random-effects meta-analyses with the inverse variance method to pool relative risks (RRs) and 95% confidence intervals (CIs). A series of statistical tests were designed to classify the strength of evidence of significant associations as convincing, highly suggestive, suggestive, or weak evidence.

**Results:**

From 25,731 initial and 2289 updated search records, 36 studies examining 31 non-genetic risk factors for YOD were identified. Of the 31 associations examined, 21 were nominally statistically significant at *P* < 0.05 based on random-effects models. Prior stroke was convincingly associated with an increased risk of YOD. Evidence of association was highly suggestive for alcohol use disorders, diabetes, depression, mood disorders, Parkinson's disease, multiple sclerosis, use of antidepressants/antipsychotics, and asthma.

**Conclusion:**

We found that the risk of dementia in young individuals may be closely related to neuropsychiatric symptoms and clinical alcohol disorders. Future research should further validate these findings and explore intervention strategies to reduce dementia risk in younger individuals.

## Graphical abstract

**Figure:**
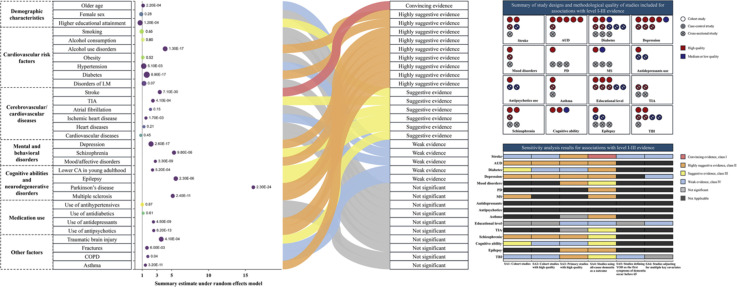

## 1 Introduction

Dementia represents an escalating global health challenge, with the number of affected individuals projected to nearly triple by 2050 compared to 2019 [1]. While late-onset dementia (LOD) remains the most common form, young-onset dementia (YOD)—defined by a provisional consensus as the onset of symptoms before the age of 65 [2]—has attracted increasing attention. YOD significantly disrupts career trajectories, places substantial caregiving responsibilities on partners and families, and imposes considerable strain on healthcare systems [[3], [4], [5]]. Two recent large-scale studies have provided invaluable insights into the global prevalence and incidence of YOD, reporting age-standardized estimates of 119 and 11 per 100,000 population, respectively [6,7]. According to the latest data from the Global Burden of Disease Study 2021 (GBD 2021), the age-standardized death rate (ASDR) and age-standardized disability-adjusted life years (DALYs) rate for YOD among individuals aged 40–64 years were 3.37 (95 % uncertainty interval: 0.6–10.67) and 173.29 (77.88–407.67), respectively, in 2021[8]. Furthermore, the average annual percentage change (AAPC) from 1990 to 2021 in both ASDR and age-standardized DALYs rates exhibited a consistent upward trend, with AAPCs of 0.07 (0.05 to 0.10) and 0.08 (0.05 to 0.10), respectively [8].

Although genetic factors are widely acknowledged as significant contributors to YOD, inherited forms of dementia may remain relatively rare in clinical practice [9]. A previous study also suggested that only 6 % of individuals with young-onset Alzheimer's disease (YOAD) carry known autosomal dominant mutations in the amyloid precursor protein (APP) or presenilin 1 and 2 (PSEN1/2) genes, which have been associated with AD [10]. This suggests that other, yet unidentified, genetic risk factors or environmental factors may play a critical role in the pathogenesis of YOD. The 2024 update of the Lancet Commission on dementia highlighted the potential to prevent or delay nearly half of all dementia cases through the elimination of 14 modifiable risk factors across the lifespan [11]. However, the majority of existing evidence predominantly focuses on LOD, resulting in a relative paucity of research into the specific risk profiles associated with YOD. In this study, we aim to conduct a comprehensive synthesis of the available evidence to identify specific modifiable risk factors associated with the onset of YOD. Additionally, we apply a rigorous framework for the classification of evidence credibility [[12], [13], [14]], categorizing significant findings into four levels.

## 2 Methods

### 2.1 Search strategy and selection criteria

This umbrella review reported following the 2020 Preferred Reporting Items for Systematic Reviews and Meta-analyses (PRISMA) reporting guideline (Supplementary Table 1) and was prospectively registered with PROSPERO (CRD42024580988).

One investigator (JYZ) conducted a systematic search of PubMed, Embase, Web of Science, and Ovid MEDLINE from inception to July 8, 2024, for studies on non-genetic risk factors associated with YOD. The complete search strategy for each database is detailed in Supplementary Table 2. A secondary search conducted on 22 May 2025 used the identical search strategy to identify relevant literature published between 2024 and 2025. No filters (e.g., article type or language) were applied to the search results. Additionally, the investigator manually screened the reference lists of relevant systematic reviews [[15], [16], [17], [18]] and eligible articles to identify any studies that may have been overlooked in the initial searches. According to pre-established eligibility criteria, de-duplicated articles underwent a meticulous review of the title/abstract and, if necessary, the full text. The study selection process was independently conducted by two investigators (JYZ and DDY), and any discrepancies were resolved through discussion with an epidemiology expert (QJW). No language restrictions were imposed during the screening process.

Our study aims to compare the incidence or prevalence of YOD across different categories of modifiable risk factors. For this purpose, we included only peer-reviewed epidemiological studies (e.g., cohort studies [CSs], case-control studies [CCSs], cross-sectional studies [CSSs]). The definition of YOD adheres to that established in the original study. Generally, YOD refers to the onset of dementia symptoms prior to the age of 65. However, some studies define YOD as a diagnosis of dementia before the age of 65 when the timing of symptom onset is unavailable. Original studies that focused exclusively on outcomes related to the progression and/or mortality of YOD were also excluded. Risk factors were defined as any attribute, characteristic, or exposure of an individual that increases the likelihood of developing a disease or injury.

### 2.2 Data analysis

We utilized the Newcastle-Ottawa Scale (NOS) to evaluate the methodological quality of CSs and CCSs. Due to the inherent limitations in the design of CSSs, we did not conduct separate quality assessments for these studies, instead considering their quality inherently low. Two investigators (JYZ and JL) independently performed the evaluations using the NOS tool, and any discrepancies were resolved through discussion to reach a consensus. The level of agreement between the investigators' assessments for CSs was quantified using Cohen's kappa.

Data extraction was customized for each study design and conducted independently in duplicate by JYZ and YH. Disagreements were resolved through discussion or, if necessary, by arbitration from a third author (QJW). The data extracted from each eligible CS included the first author, publication year, study location, data source (cohort or registry name), recruitment period, follow-up information (e.g., median length of follow-up), number of YOD cases and participants, proportion of males, cut-off age criterion for YOD, modifiable risk factors examined, dementia identification, and International Classification of Diseases (ICD) codes used for dementia diagnosis and Anatomical Therapeutic Chemical (ATC) codes for identifying anti-dementia medications (if available). From each included CCS or CSS, we extracted the first author, publication year, study location, case source and control selection (sample source and study period for CSSs), number of YOD cases and controls (sample size for CSSs), and additional information comparable to that extracted from CSs. Fully or maximally adjusted factor-level effect estimates from original studies were extracted, along with 95 % CIs, metrics used for analysis (e.g., odds ratio [OR], relative risk [RR], hazard ratio [HR]), stratifications for risk factor comparisons, number of YOD cases and total sample size (or person-years) in each stratum (number of YOD cases and controls in each stratum for CCSs or CSSs), as well as covariate adjustments.

When an original study reported multiple risk factors, we extracted the estimates separately. In studies that repeatedly examined the same risk factor using the same data source, we selected the effect size reported by the study with either the largest number of YOD cases or the largest sample size (when the number of YOD cases was not reported) for subsequent analysis. A systematic review and meta-analysis estimated the global age-standardized incidence rate of YOD in individuals aged 30–64 years at 11 per 100,000 person-years [6]. We considered various effect metrics for binary endpoints as approximate estimates of the relative risk (RR) under the rare outcome assumption [19,20]. We summarized effect estimates for an individual risk factor when at least three studies from distinct populations reported consistent exposure-outcome associations. Data synthesis was conducted using inverse variance weighted random-effects meta-analysis, accounting for the expected heterogeneity in the definitions of outcomes and risk factors. The pooled results were presented as RR with 95 % CIs. Between-study heterogeneity was assessed using Cochran's Q test and I^2^ statistics based on the DerSimonian-Laird method. We calculated the 95 % prediction intervals (PIs) for the summary random-effects estimates to further account for between-study heterogeneity. This interval reflects the likely range within which effect estimates for the associations between risk factors and YOD are expected to fall in future studies. A 95 % PI excluding the null indicates that the association is likely to remain in future studies [21]. Egger's asymmetry test was used to evaluate potential publication and small-study effects (large studies having significantly more conservative results than smaller studies) biases [22]. A *p-*value of < 0.10 in Egger's test was considered indicative of small-study effect bias. Subgroup analyses were performed based on stratification by study design.

In addition to the statistical tests mentioned above, we also checked the statistical significance of the largest study, which was determined based on the smallest standard error. We also conducted the excess statistical significance test for each meta-analysis to determine whether the observed number (O) of studies with statistically significant results exceeded the expected number (E) of such studies [23]. The expected number of studies with significant results was calculated as the sum of the statistical power estimates. The power of each original study was computed using an algorithm based on a non-central t distribution, assuming the true effect size was equal to that of the largest study [24,25]. Excess significance for each meta-analysis was determined using a threshold of *p* < 0.10. Finally, we recalculated the summary effect estimates using random-effects models after applying a 10 % credibility ceiling to account for potential methodological limitations in observational studies that could lead to spurious significance [26].

### 2.3 Evaluating evidence hierarchy

We further evaluated the credibility of evidence for each significant association between risk factors and YOD by applying criteria consistent with previously published methods. The strength of each statistically significant association was classified as convincing, highly suggestive, suggestive, or weak. Convincing associations (Class I Evidence) were defined by the following criteria: *p*-value under random-effects models<1 × 10^–6^, more than 1000 YOD cases, *p*-value of the largest study<0.05, no large heterogeneity (I^2^<50 %), 95 % PI excluding the null value, no evidence of small-study effect (*p* > 0.10 in Egger's test), no signs of excess significance bias (*p* > 0.10), *p*-value of the random-effects summary estimate under 10 % credibility ceiling<0.05. Highly suggestive associations (Class II Evidence) were defined by highly significant summary estimates (*p*-value under random-effects models<1 × 10^–6^), more than 1000 YOD cases, and a *p*-value<0.05 in the largest study. Suggestive associations (Class III Evidence) required a *p*-value<10^–3^ from random-effects models and more than 1000 YOD cases. Weak associations (Class IV Evidence) were those that only met the *p*-value<0.05 criterion under random-effects models.

### 2.4 Sensitivity analysis

We conducted a series of sensitivity analyses for associations initially classified as evidence levels I-III. These analyses included reassessing the credibility of evidence in CSs, CSs with high methodological quality (scores≥7), original studies with high methodological quality, studies using all-cause dementia rather than dementia subtypes as outcomes, studies defining YOD as the onset of dementia symptoms before age 65, and studies adjusted for multiple key covariates. Furthermore, we used the summary random effects estimates, estimates from studies with the largest sample sizes, and estimates from studies with the largest number of YOD cases as alternative plausible effect sizes to reassess excess significance bias in the association of evidence level I. Finally, we reassessed whether the associations with level I evidence remained statistically significant at credibility ceilings of 15 % and 20 %.

## 3 Results

**Fig. 1:**
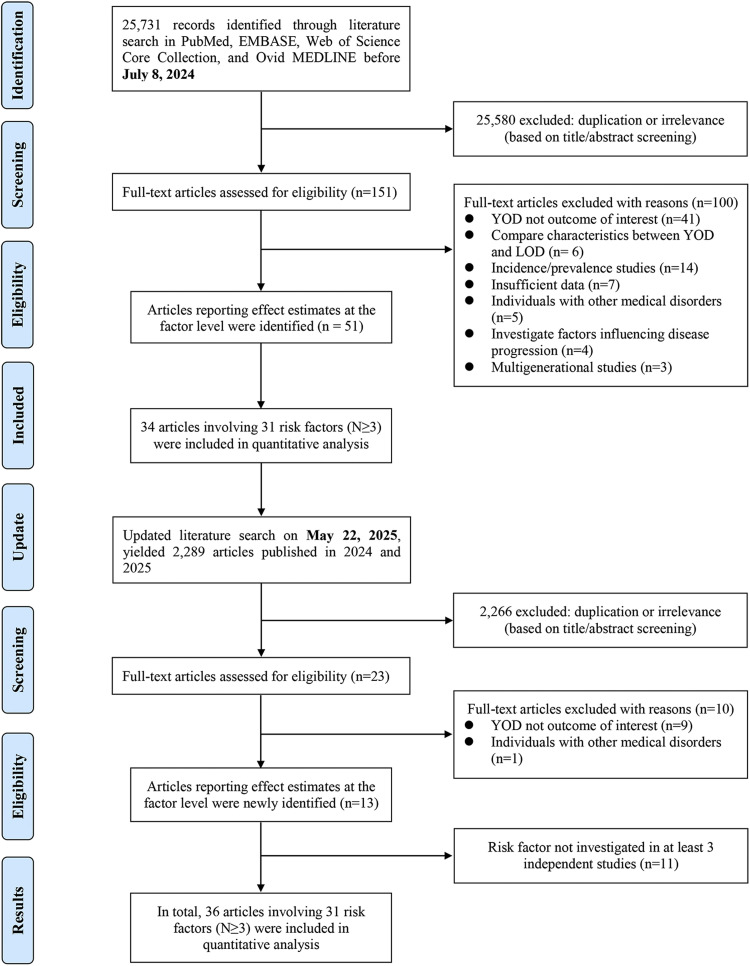
Flowchart of selection of studies on non-genetic risk factors associated with young-onset dementia.

We ultimately identified 64 publications that provided effect estimates at the factor levels and included 31 associations of risk factors with YOD risk from 36 publications [[27], [28], [29], [30], [31], [32], [33], [34], [35], [36], [37], [38], [39], [40], [41], [42], [43], [44], [45], [46], [47], [48], [49], [50], [51], [52], [53], [54], [55], [56], [57], [58], [59], [60], [61], [62]] for subsequent quantitative analysis (Fig. 1, Supplementary Table 3).

The descriptive characteristics of the included studies, classified by study design, factor-level effect estimates (including adjustments), and the ICD or ATC codes used in studies with passive case ascertainment, are presented in Supplementary Tables 4–6. A total of 26 CSs, 22 CCSs, and 5 CSSs meeting the eligibility criteria were published between 1991 and 2025. Of the 26 CSs, 18 examined modifiable risk factors derived from three or more distinct databases and were included in subsequent analyses [31,36,38,39,[44], [45], [46], [47],[50], [51], [52], [53], [54], [55], [56],^59^,^60^,^62^]. Five studies used insurance claims data from the Korean National Health Insurance Service [31,36,44,60,62]; three utilized data from the UK Biobank [38,46,56]; and three used data from the Swedish Military Service Conscription Registry, linked to medical records from other Swedish national registries [[50], [51], [52]]. Three studies were conducted in Finland: one using the Helsinki Birth Cohort linked to Finnish national registries [54], another with pooled individual-level data from two Finnish cohorts [45], and the third with longitudinal Finnish population registry data linked to other Finnish national registries [39]. Two studies were conducted in Denmark: one using the Danish Conscription Database linked to medical records from other Danish national registries [53], and the other constructing a population-based cohort using six Danish national databases [55]. The remaining two used utilized U.S. insurance claims data [47,59]. Most studies (16/18) distinguished YOD from LOD using a diagnostic age criterion of less than 65 years. One study applied a diagnostic age threshold of less than 60 years [53], while another used a recruitment age criterion of less than 60 years [36]. Except for one study that focused on Alzheimer's disease and related dementias [47], all others considered all-cause dementia as the outcome. All CSs employed passive case ascertainment, identifying dementia through ICD codes in insurance claims or medical records, and/or using ATC codes for anti-dementia medication prescriptions.

**Fig. 2:**
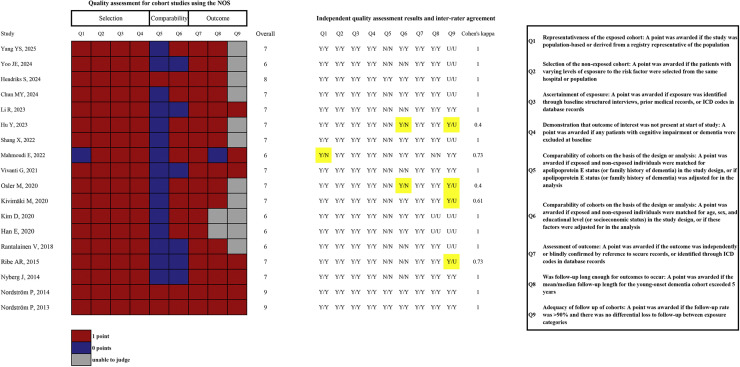
Methodological quality of cohort studies included in the meta-analysis.

A total of 14 CCSs [27,29,30,32,33,35,[41], [42], [43],^48^,^49^,^57^,^58^,^61^] and 4 CSSs [28,34,37,40] were included in the quantitative analysis, examining a range of modifiable risk factors. Among the 18 CCSs or CSSs, 3 were from Italy [27,35,48]; 2 from Denmark [32,33], the United States [42,49], Australia [29,34], France [28,30], and the Netherlands [57,58], respectively; and 1 from the United Kingdom [37], Canada [40], Japan [41], Israel [43], and South Korea [61], respectively. Among the 14 CCSs, 8 focused on AD as the primary outcome [30,32,33,41,43,49,57,58], 5 investigated risk factors for all-cause dementia [27,29,35,48,61], and 1 considered both AD and frontotemporal dementia as outcomes of interest [42]. All 4 CSSs examined all-cause dementia as the primary outcome [28,34,37,40]. The methods for identifying dementia and the age-related cut-off criteria for YOD show greater variability across these studies. The details of the quality assessment results for the CSSs and CCSs included in the quantitative analysis are shown in Fig. 2 and Supplementary Table 7. The median scores (IQR) for the CSs and CCSs were 7 (6–7) and 6 (6–7), respectively. Two CSs, one CS, and 15 studies (including 10 CSs and 5 CCSs) received scores of 9, 8, and 7, respectively. The quality assessment results for studies not included in the quantitative analysis are presented in Supplementary Table 8.

A total of 31 modifiable risk factors across seven categories were supported by evidence from at least three distinct datasets (Supplementary Table 9). Among the selected demographic characteristics, older age (RR=1.12, 95 %CI: 1.05–1.18, I^2^=96 %, *k* = 4) and lower educational attainment (RR for high vs. low=0.71, 95 %CI: 0.60–0.85, I^2^=92 %, *k* = 8) were identified as risk factors for YOD. Certain cardiovascular risk factors, including prior alcohol use disorders (AUD) (RR=4.11, 95 %CI: 2.97–5.68, I^2^=91 %, *k* = 7), hypertension (RR=1.20, 95 %CI: 1.06–1.36, I^2^=84 %, *k* = 9), and diabetes (RR=1.61, 95 %CI: 1.44–1.80, I^2^=88 %, *k* = 11), were associated with an increased risk of YOD. In the examined cerebrovascular/cardiovascular diseases, prior stroke (RR=3.38, 95 %CI: 2.74–4.17, I^2^=12 %, *k* = 5), TIA (RR=2.45, 95 %CI: 1.49–4.04, I^2^=78 %, *k* = 4), and ischemic heart disease (RR=1.50, 95 %CI: 1.16–1.93, I^2^=78 %, *k* = 3) were associated with an increased risk of YOD. All selected mental and behavioral disorders and neurodegenerative disorders were reported to be related to YOD. Regarding medication exposure, the use of antidepressants (RR=2.52, 95 %CI: 1.85–3.44, I^2^=82 %, *k* = 3) and antipsychotics (RR=2.56, 95 %CI: 1.98–3.31, I^2^=64 %, *k* = 3) was linked to a higher risk of YOD. Finally, we also observed associations between TBI (RR=3.58, 95 %CI: 1.77–7.26, I^2^=99 %, *k* = 7), fractures (RR=1.64, 95 %CI: 1.15–2.34, I^2^=88 %, *k* = 4), COPD (RR=1.76, 95 %CI: 1.02–3.03, I^2^=91 %, *k* = 3), and asthma (RR=1.43, 95 %CI: 1.28–1.58, I^2^=26 %, *k* = 3) with an increased risk of YOD. The forest plots for the 31 risk factors assessed, along with the results of the subgroup analyses, are shown in Supplementary Figures 1–58.

We further categorized the strength of evidence for each identified association using a series of statistical tests (Supplementary Tables 9–10). The risk factor with a convincing association was a prior history of stroke. The risk factors with highly suggestive associations were alcohol use disorders, diabetes, depression, mood/affective disorders, Parkinson's disease, multiple sclerosis, use of antidepressants, use of antipsychotics, asthma. Evidence of association was suggestive for lower educational attainment, TIA, schizophrenia, lower cognitive ability in young adulthood, epilepsy, traumatic brain injury (Table 1). 5 were graded as weak evidence, and the remaining 10 did not show statistically significant associations (Table 1). We did a series of sensitivity analyses on meta-analyses of 16 risk factors graded as convincing, highly suggestive, or suggestive evidence (Supplementary Tables 11–17). Subset analysis limited to CSs revealed that four associations (AUD [class II], depression [class II], MS [class II], and lower cognitive ability in young adulthood [class III]) retained their original ranking, while one class III association (schizophrenia) was reclassified as class II. We also applied different assumed true effect sizes and credible ceilings to the class I evidence and found that the result diverged from the original analysis when a 20 % credible ceiling was used (Supplementary Table 18).

**Table 1:** Summary of level of evidence for associations between modifiable factors and risk of young-onset dementia.

Class of evidence	Grading criteria	Risk factors
Convincing evidence (class I)	p < 1 × 10^–6^ under random effects; >1000 cases of young-onset dementia; p < 0.05 of the largest study; no large heterogeneity (I^2^<50 %); 95 % prediction interval excludes the null value; no evidence of small-study effect (p > 0.10); no signs of excess significance bias (p > 0.10); retained statistical significance in 10 % credibility ceiling	Stroke
Highly suggestive evidence (class II)	p < 1 × 10^–6^ under random effects; >1000 cases of young-onset dementia; p < 0.05 of the largest study	Alcohol use disorders, diabetes, depression, mood/affective disorders, Parkinson's disease, multiple sclerosis, use of antidepressants, use of antipsychotics, asthma
Suggestive evidence (class III)	p < 1 × 10^–3^ under random effects; >1000 cases of young-onset dementia	Higher educational attainment, transient ischemic attack, schizophrenia, lower cognitive ability in young adulthood, epilepsy, traumatic brain injury
Weak evidence (class IV)	p < 0.05 under random effects	Older age, hypertension, ischemic heart disease, fractures, chronic obstructive pulmonary disease
Not significant	p > 0.05 under random effects	Female sex, smoking, alcohol consumption, obesity, disorders of lipoprotein metabolism, atrial fibrillation, heart diseases, cardiovascular diseases, use of antihypertensives, use of antidiabetics

## 4 Discussion

This study presents a comprehensive quantitative synthesis of observational data to identify 21 potential risk factors for YOD. Using established classification criteria for credibility of evidence, we further assessed the strength of these associations. Stroke was classified as convincing evidence, while 9 associations were rated as highly suggestive, 6 as suggestive, and 5 as weak evidence. However, sensitivity analyses revealed that many associations lost their primary strength due to insufficient supporting data. Only two associations—AUD and depression—retained highly suggestive evidence following sensitivity analyses, whereas the association between schizophrenia and YOD was upgraded from suggestive evidence to highly suggestive evidence.

To the best of our knowledge, this is the first comprehensive quantitative synthesis of global evidence identifying modifiable risk factors for YOD. Moreover, it is the first study to apply a hierarchical classification to the credibility of these findings, offering a more robust framework for evaluating the strength of the evidence. Two prior systematic reviews and one meta-analysis have assessed risk factors for YOD [15,17,18]. Both systematic reviews focused on environmental or modifiable risk factors, while the meta-analysis specifically investigated the association between atrial fibrillation and YOD. The latest systematic review [17], consolidating findings from 10 CSs and 12 CCs, identified several potential risk factors for YOD, including traumatic brain injury, atrial fibrillation, stroke, diabetes, hypercholesterolemia, alcohol consumption, low educational attainment, lack of participation in leisure activities, and unhealthy dietary patterns. These inferences were made based on effect sizes and statistical significance reported in the included studies. An earlier systematic review [15], incorporating 14 observational studies, first identified potential risk factors for YOD through a qualitative evidence-based summary, including cardiovascular disease, psychiatric disorders, heavy alcohol consumption, and estrogen-related factors. However, the evidence supporting these associations was often insufficient and inconsistent across studies, and the absence of quantitative analysis raised concerns regarding the reliability of these conclusions. Our quantitative analysis further corroborated some of these associations, while others were not identified as significant risk factors for YOD.

A meta-analysis [18] of 6 studies investigating the association between atrial fibrillation and YOD revealed heterogeneous results. Variability in findings was attributed to differences in the age threshold used to define YOD. Consistent with our findings, when the age threshold for YOD was set at 65 years, the combined analysis did not demonstrate a significant association between atrial fibrillation and YOD. This age criterion is often linked to retirement age, which is commonly set at 65 years in many countries, and is thus frequently adopted for defining YOD [2]. However, when the age threshold was raised to 70 years, a significant positive association between atrial fibrillation and dementia emerged. This finding suggests that the risk factor profile for YOD may differ from that of LOD. The 2024 update of the Lancet Commission on dementia suggests that proactive measures to mitigate 14 modifiable risk factors for LOD (low educational attainment, hearing impairment, high low-density lipoprotein cholesterol, depression, traumatic brain injury, physical inactivity, smoking, diabetes, hypertension, obesity, excessive alcohol consumption, social isolation, air pollution, and untreated vision loss) could potentially prevent up to half of all cases [11]. In line with these findings, we also identified low educational level, AUD, diabetes, hypertension, depression, and traumatic brain injury as significant risk factors for YOD. However, we did not find an association between lipid metabolism-related diseases, smoking, or obesity and YOD. Additionally, several risk factors established for LOD could not be assessed in relation to YOD due to the limited number of available studies. Overall, our findings suggest that YOD is more prevalent among individuals with a history of neurological or psychiatric disorders, as well as those who experienced lower educational attainment and cognitive deficits early in life. In contrast, metabolic disorders and sensory impairments appear to contribute less significantly to the onset of YOD, particularly when compared to LOD. These observations highlight the multifactorial nature of YOD and underscore the need for further research to refine our understanding of the distinct risk factors that may contribute to its development.

Along with identifying specific risk factors for YOD, we also assessed the credibility of these associations to enhance the interpretability of our findings. Convincing evidence indicates that a history of stroke is associated with an increased risk of YOD, which may reflect underlying dementia etiologies, such as post-stroke cognitive impairment and dementia (PSCID). The shared genetic and environmental susceptibilities between stroke and dementia may help explain this phenomenon [[63], [64], [65]]. Age and stroke severity are key determinants of PSCID [66,67]. A population-based study from Oxfordshire, UK, observed a lower absolute risk of dementia in younger stroke patients compared to older patients [68]. However, relative to age-matched controls, the relative effect of stroke remained significant in patients aged 64 years or younger, which aligns with our findings [68]. Additionally, the differential risk of dementia between stroke and transient ischemic attack (TIA) supports our findings [68], with a history of TIA associated with a smaller effect size for YOD compared to stroke, which is recognized as suggestive evidence. Notably, two original studies [29,37] analyzed stroke and TIA as a combined exposure, which may have led to exposure misclassification, potentially resulting in an underestimation or overestimation of the association. While the association between stroke and YOD was confirmed across various sensitivity analyses, the reduced confidence in the evidence suggests that further high-quality CSs are needed to reinforce our findings.

Clinical alcohol disorders (e.g., AUD) and heavy drinking (e.g., more than 21 UK units of ethanol per week) are closely associated with the onset of dementia [45,69]. Ethanol, as a neurotoxic substance, can cross the blood-brain barrier and directly affect neurons [70]. Prolonged exposure to high concentrations of ethanol and its metabolite acetaldehyde can lead to permanent structural and functional brain damage [70]. Moreover, alcohol-related thiamine deficiency, epilepsy, hepatic encephalopathy, and head trauma further exacerbate neurobiological damage, thereby accelerating cognitive decline and increasing the risk of dementia [[71], [72], [73], [74]]. In this study, highly suggestive evidence supports a significant association between AUD and an elevated risk of YOD, with this association remaining robust across a range of sensitivity analyses. This finding is consistent with the results of a national retrospective CS [69] involving over 30 million participants and more than one million dementia cases, which found a strong link between alcohol use disorder and dementia for both sexes. Within this cohort, a secondary analysis of nearly 60,000 cases of YOD revealed that AUD plays a particularly significant role in this population, with 57 % of YOD patients also diagnosed with AUD [69]. Given the higher prevalence of AUD among young populations [75], implementing targeted alcohol control policies, enhancing health education to raise awareness of alcohol's harmful effects, reducing the social stigma surrounding treatment-seeking, and promoting early diagnosis and integrated treatment approaches are crucial for preventing or reducing the early onset of alcohol-related dementia. Compared to the strong association between AUD and YOD, further analysis did not find a correlation between alcohol consumption and YOD. This may be attributable to differences in sample sizes, exposure measurement methods, and the categorization of exposure across studies. Of the four original studies included in the meta-analysis, one is a large CS from the UK Biobank [46], while the conclusions of the other three small-sample CCSs [29,30,35] lack precision and statistical power, and may be subject to chance. When considering the methods of exposure measurement and classification, these studies exhibit greater heterogeneity. For example, the study from the UK Biobank defined excessive alcohol consumption based on participants' self-reported frequency and quantity of alcohol intake, with more than one drink (containing 8 *g* of ethanol) per day for women and more than two drinks per day for men considered excessive [46]. Another CCS from France used a weekly dietary survey to define regular drinking as consuming more than three standard drinks per day (the ethanol content of a standard drink was not specified) and observed a lower frequency of regular drinking in the YOD population [30]. This unconventional protective effect was suggested by the authors to possibly be related to the coarse exposure assessment method in the case group, which was reported by the patients or their caregivers. Additionally, due to the long delay between the onset and diagnosis of dementia, most observational studies measured alcohol consumption close to the time of diagnosis, which may reflect a changed drinking behavior. Two other studies assessed alcohol intake using a dietary frequency questionnaire and the Alcohol Use Disorder Identification Test-Consumption (AUDIT-C) score [29,35]. Given the significant methodological differences across studies, we are unable to accurately determine the quantitative relationship between alcohol consumption and YOD based on the currently available evidence. Future studies should aim to minimize potential biases through appropriate study design and statistical analysis, such as measuring early dietary habits before the onset of dementia and adjusting for previous alcohol consumption among those classified as former drinkers in the analysis.

Highly suggestive evidence supports the association between depression and YOD, despite significant heterogeneity across studies. Although these studies differ in terms of region, gender composition of the population, methods for identifying depression and dementia, definitions of early onset, and matching or adjustment factors, all studies consistently indicate that individuals with depression have an increased risk of YOD. Sensitivity analysis shows that the highly suggestive association between depression and YOD remains stable in CSs, high-quality CSs, and studies with all-cause dementia as the outcome. Notably, although only two studies [29,51] matched or adjusted for key confounders, a significant association between depression and YOD was still observed in subsequent meta-analyses. There is ongoing debate about whether depression is an early clinical manifestation of dementia, a reactive symptom (reverse causality), an independent risk factor, or a combination of these [11,76]. The mechanisms underlying the relationship between depression and dementia risk remain unclear, with several potential mechanisms proposed, including the loss of noradrenergic neurons in the locus coeruleus [77,78], the loss of serotonergic neurons in the dorsal raphe nucleus [77], chronic glucocorticoid secretion [79,80], and the release of inflammatory cytokines [81]. Our findings also suggest that a history of antidepressant use is associated with an increased risk of YOD. A registry-based nested CCS assessed the use of prescription medications in the 10 years prior to an YOAD diagnosis and compared it with prescription medication usage in the general population of the region [33]. The study found that proximal exposure to antidepressants was associated with a higher risk of YOAD, while distal exposure was associated with a lower risk [33]. Evidence from the UK Biobank suggests that depression patients receiving pharmacological treatment had a lower risk of dementia compared to those who did not receive treatment [82]. However, whether pharmacological treatment for depression can reduce the risk of YOD remains to be further validated in future studies. In addition to the association between depression and YOD, our study also found that schizophrenia and other mental and behavioral disorders are associated with a higher risk of YOD. Therefore, effective prevention of mental disorders should include strengthening mental health education, enhancing social connections and support, early identification and intervention of high-risk groups, and maintaining a healthy lifestyle. These strategies represent a more cost-effective approach to reducing the incidence of YOD in younger populations. Effective prevention of mental disorders in younger populations—through measures such as strengthening mental health education, enhancing social connectivity and support, early identification and intervention of high-risk groups, and promoting healthy lifestyles—remains crucial. Such strategies may represent a more cost-effective approach to mitigating the incidence of YOD in this population.

Several limitations necessitate cautious interpretation of the results. First, due to the nature of observational studies, the current work only examines the associations between various potential factors and YOD, presented in terms of correlation. While the reported associations may be significant, they do not directly imply causality. Additionally, variations in the selection of covariates and differences in data quality across the original studies mean that residual confounding bias cannot be ruled out, although such bias is a known limitation of observational research [83]. Second, many of the identified associations exhibit considerable heterogeneity, which may be attributed to variations in the definition of "young-onset" and differences in diagnostic practices across studies. YOD is genetically and environmentally distinct from LOD, yet there is no consensus on how to define or even term this specific group. A provisional consensus, developed using the Delphi method, defines YOD as dementia with symptom onset before the age of 65 [2]. However, only a small proportion of studies included in our analysis adhered to this definition; many others defined YOD based on diagnosis before the age of 65. This discrepancy may reflect the greater availability of diagnostic age data compared with age of symptom onset. There is an urgent need for further consensus to improve understanding of this population and to standardize and harmonize the identification of YOD in future research, while considering the variability in age thresholds based on operational definitions. Third, due to the limited number of observational studies, especially CSs, in meta-analyses examining specific risk factors (such as alcohol consumption, obesity, atrial fibrillation, ischemic heart disease) and their association with the risk of YOD, statistical power is insufficient, leading to lower confidence in the evidence.

## 5 Conclusions

Our results indicate that the risk of dementia in young individuals is closely associated with neuropsychiatric symptoms and AUD, while metabolic factors were not identified as high-priority risk factors for YOD, in contrast to the risk factors for LOD. Future research should build on these findings to further explore the potential mechanisms of neuropsychiatric symptoms and AUD in YOD and develop more targeted early screening and intervention strategies to reduce dementia risk in younger populations.

## Declaration of generative AI and AI-assisted technologies in the writing process

We have not used any AI at all.

## Funding

No funding was received for this study.

## Data availability

All data analyzed during this study are included in supplementary materials.

## CRediT authorship contribution statement

**Jiayu Zhang:** Conceptualization, Data curation, Formal analysis, Methodology, Software, Visualization, Writing – original draft, Writing – review & editing, Investigation. **Dandan Yang:** Data curation, Formal analysis, Investigation, Methodology. **Jian Liang:** Data curation, Formal analysis, Investigation, Methodology. **Yin Hu:** Data curation, Formal analysis, Investigation, Methodology. **Liping Rao:** Data curation, Investigation, Methodology. **Jun Huang:** Data curation, Investigation, Methodology. **Qijun Wu:** Conceptualization, Formal analysis, Methodology, Software, Supervision, Project administration, Validation. **Bo Jiang:** Conceptualization, Project administration, Supervision, Validation.

## Declaration of competing interest

The authors declare that they have no known competing financial interests or personal relationships that could have appeared to influence the work reported in this paper.

## Appendix Supplementary materials

Image, application 1
