# Supplementary material for: Identifying risk factors of young-onset dementia and evaluating evidence hierarchy: a meta-analysis and umbrella review
Source: J Prev Alzheimers Dis. 2026 Jan 1;13(3):100467. doi: 10.1016/j.tjpad.2025.100467 (PMC12988373; doi:10.1016/j.tjpad.2025.100467)
Supplement: Supplementary file 1 [file mmc1.docx]

**Identifying risk factors of young-onset dementia and evaluating evidence hierarchy: a meta-analysis and umbrella review**

**List of Supplementary files**

[Supplementary Table 1. PRISMA checklist 5](#_Toc196123047)

[Supplementary Table 2. Search strategy from database inception to July 8, 2024, for original articles on non-genetic risk factors associated with young-onset dementia 10](#_Toc196123048)

[Supplementary Table 3. List of excluded studies, based on full-text evaluation 11](#_Toc196123049)

[Supplementary Table 4. Characteristics of studies investigating risk factors for the development of young-onset dementia by study design 18](#_Toc196123050)

[Supplementary Table 5. Effect estimates of associations between risk factors and young-onset dementia risk by study design 31](#_Toc196123051)

[Supplementary Table 6. Diagnostic codes and/or prescription records for case ascertainment 101](#_Toc196123052)

[Supplementary Table 7. Quality assessment for case-control studies included in the quantitative analysis using the Newcastle-Ottawa Scale^*^ 112](#_Toc196123053)

[Supplementary Table 8. Quality assessment for studies not included in the quantitative analysis using the Newcastle-Ottawa Scale 113](#_Toc196123054)

[Supplementary Table 9. Pooled relative risks of predictors for young-onset dementia 114](#_Toc196123055)

[Supplementary Table 10. The quality of evidence for the associations between modifiable risk factors and young-onset dementia 129](#_Toc196123056)

[Supplementary Table 11. Sensitivity analyses of Class I-III evidence by including only cohort studies 132](#_Toc196123057)

[Supplementary Table 12. Sensitivity analyses of Class I-III evidence by including only cohort studies with high methodological quality 136](#_Toc196123058)

[Supplementary Table 13. Sensitivity analyses of Class I-III evidence by including only studies with high methodological quality 140](#_Toc196123059)

[Supplementary Table 14. Sensitivity analyses of Class I-III evidence with primary studies using all-cause dementia as an outcome 145](#_Toc196123060)

[Supplementary Table 15. Sensitivity analyses of Class I-III evidence with primary studies defining YOD as onset of dementia symptoms before age 65 151](#_Toc196123061)

[Supplementary Table 16. Sensitivity analyses of Class I-III evidence with primary studies adjusting for multiple key covariates (age, sex, and educational level/socioeconomic status, and apolipoprotein E status/family history of dementia) 154](#_Toc196123062)

[Supplementary Table 17. Summary of sensitivity analyses 157](#_Toc196123063)

[Supplementary Table 18. Sensitivity analyses using various credibility ceilings and assumed true effect sizes in meta-analyses of prior stroke and risk of YOD meeting convincing evidence criteria 159](#_Toc196123064)

[Supplementary Figure 1. Forest plot of the association between older age and risk of young-onset dementia 160](#_Toc196123065)

[Supplementary Figure 2. Subgroup analysis of older age and young-onset dementia risk by study design 160](#_Toc196123066)

[Supplementary Figure 3. Forest plot of the association between female and risk of young-onset dementia 161](#_Toc196123067)

[Supplementary Figure 4. Subgroup analysis of female and young-onset dementia risk by study design 161](#_Toc196123068)

[Supplementary Figure 5. Forest plot of the association between higher educational attainment and risk of young-onset dementia 162](#_Toc196123069)

[Supplementary Figure 6. Subgroup analysis of higher educational attainment and young-onset dementia risk by study design 162](#_Toc196123070)

[Supplementary Figure 7. Forest plot of the association between smoking and risk of young-onset dementia 163](#_Toc196123071)

[Supplementary Figure 8. Subgroup analysis of smoking and young-onset dementia risk by study design 163](#_Toc196123072)

[Supplementary Figure 9. Forest plot of the association between alcohol consumption and risk of young-onset dementia 164](#_Toc196123073)

[Supplementary Figure 10. Subgroup analysis of alcohol consumption and young-onset dementia risk by study design 164](#_Toc196123074)

[Supplementary Figure 11. Forest plot of the association between alcohol use disorders and risk of young-onset dementia 165](#_Toc196123075)

[Supplementary Figure 12. Subgroup analysis of alcohol use disorders and young-onset dementia risk by study design 165](#_Toc196123076)

[Supplementary Figure 13. Forest plot of the association between obesity and risk of young-onset dementia 166](#_Toc196123077)

[Supplementary Figure 14. Subgroup analysis of obesity and young-onset dementia risk by study design 166](#_Toc196123078)

[Supplementary Figure 15. Forest plot of the association between hypertension and risk of young-onset dementia 167](#_Toc196123079)

[Supplementary Figure 16. Subgroup analysis of hypertension and young-onset dementia risk by study design 167](#_Toc196123080)

[Supplementary Figure 17. Forest plot of the association between diabetes and risk of young-onset dementia 168](#_Toc196123081)

[Supplementary Figure 18. Subgroup analysis of diabetes and young-onset dementia risk by study design 168](#_Toc196123082)

[Supplementary Figure 19. Forest plot of the association between disorders of lipoprotein metabolism and risk of young-onset dementia 169](#_Toc196123083)

[Supplementary Figure 20. Subgroup analysis of disorders of lipoprotein metabolism and young-onset dementia risk by study design 169](#_Toc196123084)

[Supplementary Figure 21. Forest plot of the association between stroke and risk of young-onset dementia 170](#_Toc196123085)

[Supplementary Figure 22. Subgroup analysis of stroke and young-onset dementia risk by study design 170](#_Toc196123086)

[Supplementary Figure 23. Forest plot of the association between TIA and risk of young-onset dementia 171](#_Toc196123087)

[Supplementary Figure 24. Forest plot of the association between atrial fibrillation and risk of young-onset dementia 172](#_Toc196123088)

[Supplementary Figure 25. Subgroup analysis of atrial fibrillation and young-onset dementia risk by study design 172](#_Toc196123089)

[Supplementary Figure 26. Forest plot of the association between ischemic heart disease and risk of young-onset dementia 173](#_Toc196123090)

[Supplementary Figure 27. Forest plot of the association between heart diseases and risk of young-onset dementia 174](#_Toc196123091)

[Supplementary Figure 28. Subgroup analysis of heart diseases and young-onset dementia risk by study design 174](#_Toc196123092)

[Supplementary Figure 29. Forest plot of the association between cardiovascular diseases and risk of young-onset dementia 175](#_Toc196123093)

[Supplementary Figure 30. Subgroup analysis of cardiovascular diseases and young-onset dementia risk by study design 175](#_Toc196123094)

[Supplementary Figure 31. Forest plot of the association between depression and risk of young-onset dementia 176](#_Toc196123095)

[Supplementary Figure 32. Subgroup analysis of depression and young-onset dementia risk by study design 176](#_Toc196123096)

[Supplementary Figure 33. Forest plot of the association between schizophrenia and risk of young-onset dementia 177](#_Toc196123097)

[Supplementary Figure 34. Subgroup analysis of schizophrenia and young-onset dementia risk by study design 177](#_Toc196123098)

[Supplementary Figure 35. Forest plot of the association between mood/affective disorders and risk of young-onset dementia 178](#_Toc196123099)

[Supplementary Figure 36. Subgroup analysis of mood/affective disorders and young-onset dementia risk by study design 178](#_Toc196123100)

[Supplementary Figure 37. Forest plot of the association between lower cognitive ability in young adulthood and risk of young-onset dementia 179](#_Toc196123101)

[Supplementary Figure 38. Forest plot of the association between epilepsy and risk of young-onset dementia 180](#_Toc196123102)

[Supplementary Figure 39. Subgroup analysis of epilepsy and young-onset dementia risk by study design 180](#_Toc196123103)

[Supplementary Figure 40. Forest plot of the association between Parkinson’s disease and risk of young-onset dementia 181](#_Toc196123104)

[Supplementary Figure 41. Subgroup analysis of Parkinson’s disease and young-onset dementia risk by study design 181](#_Toc196123105)

[Supplementary Figure 42. Forest plot of the association between multiple sclerosis and risk of young-onset dementia 182](#_Toc196123106)

[Supplementary Figure 43. Subgroup analysis of multiple sclerosis and young-onset dementia risk by study design 182](#_Toc196123107)

[Supplementary Figure 44. Forest plot of the association between use of antihypertensives and risk of young-onset dementia 183](#_Toc196123108)

[Supplementary Figure 45. Forest plot of the association between use of antidiabetics and risk of young-onset dementia 184](#_Toc196123109)

[Supplementary Figure 46. Subgroup analysis of use of antidiabetics and young-onset dementia risk by study design 184](#_Toc196123110)

[Supplementary Figure 47. Forest plot of the association between use of antidepressants and risk of young-onset dementia 185](#_Toc196123111)

[Supplementary Figure 48. Subgroup analysis of use of antidepressants and young-onset dementia risk by study design 185](#_Toc196123112)

[Supplementary Figure 49. Forest plot of the association between use of antipsychotics and risk of young-onset dementia 186](#_Toc196123113)

[Supplementary Figure 50. Subgroup analysis of use of antipsychotics and young-onset dementia risk by study design 186](#_Toc196123114)

[Supplementary Figure 51. Forest plot of the association between traumatic brain injury and risk of young-onset dementia 187](#_Toc196123115)

[Supplementary Figure 52. Subgroup analysis of traumatic brain injury and young-onset dementia risk by study design 187](#_Toc196123116)

[Supplementary Figure 53. Forest plot of the association between fractures and risk of young-onset dementia 188](#_Toc196123117)

[Supplementary Figure 54. Subgroup analysis of fractures and young-onset dementia risk by study design 188](#_Toc196123118)

[Supplementary Figure 55. Forest plot of the association between chronic obstructive pulmonary disease and risk of young-onset dementia 189](#_Toc196123119)

[Supplementary Figure 56. Subgroup analysis of chronic obstructive pulmonary disease and young-onset dementia risk by study design 189](#_Toc196123120)

[Supplementary Figure 57. Forest plot of the association between asthma and risk of young-onset dementia 190](#_Toc196123121)

[Supplementary Figure 58. Subgroup analysis of asthma and young-onset dementia risk by study design 190](#_Toc196123122)

# Supplementary Table 1. PRISMA checklist

| **Section and Topic** | **Item #** | **Checklist item** | **Location where item is reported** |
| --- | --- | --- | --- |
| **TITLE** | | |  |
| Title | 1 | Identify the report as a systematic review. | Title |
| **ABSTRACT** | | |  |
| Abstract | 2 | See the PRISMA 2020 for Abstracts checklist. | Abstract |
| **INTRODUCTION** | | |  |
| Rationale | 3 | Describe the rationale for the review in the context of existing knowledge. | Introduction, paragraphs 1–2 |
| Objectives | 4 | Provide an explicit statement of the objective(s) or question(s) the review addresses. | Introduction, paragraph 3 |
| **METHODS** | | |  |
| Eligibility criteria | 5 | Specify the inclusion and exclusion criteria for the review and how studies were grouped for the syntheses. | Methods, paragraph 3 |
| Information sources | 6 | Specify all databases, registers, websites, organisations, reference lists and other sources searched or consulted to identify studies. Specify the date when each source was last searched or consulted. | Methods, paragraph 2 |
| Search strategy | 7 | Present the full search strategies for all databases, registers and websites, including any filters and limits used. | Supplementary Table 2 |
| Selection process | 8 | Specify the methods used to decide whether a study met the inclusion criteria of the review, including how many reviewers screened each record and each report retrieved, whether they worked independently, and if applicable, details of automation tools used in the process. | Methods, paragraph 2 |
| Data collection process | 9 | Specify the methods used to collect data from reports, including how many reviewers collected data from each report, whether they worked independently, any processes for obtaining or confirming data from study investigators, and if applicable, details of automation tools used in the process. | Methods, paragraph 5 |
| Data items | 10a | List and define all outcomes for which data were sought. Specify whether all results that were compatible with each outcome domain in each study were sought (e.g. for all measures, time points, analyses), and if not, the methods used to decide which results to collect. | Methods, paragraph 5 |
|  | 10b | List and define all other variables for which data were sought (e.g. participant and intervention characteristics, funding sources). Describe any assumptions made about any missing or unclear information. | Methods, paragraph 5 |
| Study risk of bias assessment | 11 | Specify the methods used to assess risk of bias in the included studies, including details of the tool(s) used, how many reviewers assessed each study and whether they worked independently, and if applicable, details of automation tools used in the process. | Methods, paragraph 4 |
| Effect measures | 12 | Specify for each outcome the effect measure(s) (e.g. risk ratio, mean difference) used in the synthesis or presentation of results. | Methods, paragraph 6 |
| Synthesis methods | 13a | Describe the processes used to decide which studies were eligible for each synthesis (e.g. tabulating the study intervention characteristics and comparing against the planned groups for each synthesis (item #5)). | Methods, paragraph 6 |
|  | 13b | Describe any methods required to prepare the data for presentation or synthesis, such as handling of missing summary statistics, or data conversions. | Methods, paragraph 6 |
|  | 13c | Describe any methods used to tabulate or visually display results of individual studies and syntheses. | Methods, paragraph 6 |
|  | 13d | Describe any methods used to synthesize results and provide a rationale for the choice(s). If meta-analysis was performed, describe the model(s), method(s) to identify the presence and extent of statistical heterogeneity, and software package(s) used. | Methods, paragraph 6 |
|  | 13e | Describe any methods used to explore possible causes of heterogeneity among study results (e.g. subgroup analysis, meta-regression). | Methods, paragraph 6 |
|  | 13f | Describe any sensitivity analyses conducted to assess robustness of the synthesized results. | Methods, paragraph 9 |
| Reporting bias assessment | 14 | Describe any methods used to assess risk of bias due to missing results in a synthesis (arising from reporting biases). | Methods, paragraph 6 |
| Certainty assessment | 15 | Describe any methods used to assess certainty (or confidence) in the body of evidence for an outcome. | Methods, paragraphs 7-8 |
| **RESULTS** | | |  |
| Study selection | 16a | Describe the results of the search and selection process, from the number of records identified in the search to the number of studies included in the review, ideally using a flow diagram. | Results, paragraph 1; Supplementary Figure 1 |
|  | 16b | Cite studies that might appear to meet the inclusion criteria, but which were excluded, and explain why they were excluded. | Supplementary Table 3 |
| Study characteristics | 17 | Cite each included study and present its characteristics. | Results, paragraphs 2-3; Supplementary Tables 4-6 |
| Risk of bias in studies | 18 | Present assessments of risk of bias for each included study. | Results, paragraph 3; Supplementary Figure 2; Supplementary Tables 7-8 |
| Results of individual studies | 19 | For all outcomes, present, for each study: (a) summary statistics for each group (where appropriate) and (b) an effect estimate and its precision (e.g. confidence/credible interval), ideally using structured tables or plots. | Supplementary Table 5 |
| Results of syntheses | 20a | For each synthesis, briefly summarise the characteristics and risk of bias among contributing studies. | Supplementary Table 9 |
|  | 20b | Present results of all statistical syntheses conducted. If meta-analysis was done, present for each the summary estimate and its precision (e.g. confidence/credible interval) and measures of statistical heterogeneity. If comparing groups, describe the direction of the effect. | Results, paragraph 4; Supplementary Figures 3-60 |
|  | 20c | Present results of all investigations of possible causes of heterogeneity among study results. | Supplementary Figures 3-60 |
|  | 20d | Present results of all sensitivity analyses conducted to assess the robustness of the synthesized results. | Supplementary Tables 11-18 |
| Reporting biases | 21 | Present assessments of risk of bias due to missing results (arising from reporting biases) for each synthesis assessed. | Supplementary Table 9 |
| Certainty of evidence | 22 | Present assessments of certainty (or confidence) in the body of evidence for each outcome assessed. | Results, paragraph 5; Supplementary Table 10 |
| **DISCUSSION** | | |  |
| Discussion | 23a | Provide a general interpretation of the results in the context of other evidence. | Discussion, paragraph 1 |
|  | 23b | Discuss any limitations of the evidence included in the review. | Discussion, paragraph 7 |
|  | 23c | Discuss any limitations of the review processes used. | Discussion, paragraph 7 |
|  | 23d | Discuss implications of the results for practice, policy, and future research. | Conclusions, paragraph 1 |
| **OTHER INFORMATION** | | |  |
| Registration and protocol | 24a | Provide registration information for the review, including register name and registration number, or state that the review was not registered. | Methods, paragraph 1 |
|  | 24b | Indicate where the review protocol can be accessed, or state that a protocol was not prepared. | Not available |
|  | 24c | Describe and explain any amendments to information provided at registration or in the protocol. | Methods, paragraph 1 |
| Support | 25 | Describe sources of financial or non-financial support for the review, and the role of the funders or sponsors in the review. | Funding |
| Competing interests | 26 | Declare any competing interests of review authors. | Competing Interests |
| Availability of data, code and other materials | 27 | Report which of the following are publicly available and where they can be found: template data collection forms; data extracted from included studies; data used for all analyses; analytic code; any other materials used in the review. | Data Availability Statement |

*From:*  Page MJ, McKenzie JE, Bossuyt PM, Boutron I, Hoffmann TC, Mulrow CD, et al. The PRISMA 2020 statement: an updated guideline for reporting systematic reviews. BMJ 2021;372:n71. doi: 10.1136/bmj.n71

For more information, visit: <http://www.prisma-statement.org/>

# Supplementary Table 2. Search strategy from database inception to July 8, 2024, for original articles on non-genetic risk factors associated with young-onset dementia

| **PubMed** | | |
| --- | --- | --- |
| Search | Query | Results |
| #1 | "dementia"[MeSH Terms] OR "alzheimer disease"[MeSH Terms] OR "dement*"[Title/Abstract] OR "alzheimer*"[Title/Abstract] | 342,392 |
| #2 | "early onset"[Title/Abstract] OR "early-onset"[Title/Abstract] OR "young* onset"[Title/Abstract] OR "young-onset"[Title/Abstract] OR "under 65"[Title/Abstract] OR "before 65"[Title/Abstract] OR "younger than 65"[Title/Abstract] | 56,853 |
| #3 | #1 AND #2 | 5700 |
| **EMBASE (ELSEVIER)** | | |
| Search | Query | Results |
| #1 | 'dementia'/exp OR 'alzheimer disease'/exp OR dement*:ab,ti OR alzheimer*:ab,ti | 545,799 |
| #2 | 'early onset':ab,ti OR 'young* onset':ab,ti OR 'young onset':ab,ti OR 'under 65':ab,ti OR 'before 65':ab,ti OR 'younger than 65':ab,ti | 84,198 |
| #3 | #1 AND #2 | 8,656 |
| **Web of Science Core Collection** | | |
| Search | Query | Results |
| #1 | ALL=(dementia OR "alzheimer disease") OR TI=(dement* OR alzheimer*) OR AB=(dement* OR alzheimer*) | 383,073 |
| #2 | TI=("early onset" OR "early-onset" OR "young* onset" OR "young-onset" OR "under 65" OR "before 65" OR "younger than 65") OR AB=("early onset" OR "early-onset" OR "young* onset" OR "young-onset" OR "under 65" OR "before 65" OR "younger than 65") | 57,968 |
| #3 | #1 AND #2 | 5790 |
| **Ovid MEDLINE(R) ALL** | | |
| Search | Query | Results |
| #1 | exp dementia/ | 216,814 |
| #2 | exp alzheimer disease/ | 126,542 |
| #3 | (dement* or alzheimer*).ab,ti. | 292,980 |
| #4 | #1 OR #2 OR #3 | 336,156 |
| #5 | ("early onset" or "early-onset" or "young* onset" or "young-onset" or "under 65" or "before 65" or "younger than 65").ab,ti. | 56,272 |
| #6 | #4 AND #5 | 5,585 |

# Supplementary Table 3. List of excluded studies, based on full-text evaluation

| **Reasons for exclusion** | **References** |
| --- | --- |
| Young-onset dementia not outcome of interest | 1. Age at Onset of Heart Failure and Subsequent Risk of Dementia: A Longitudinal Cohort Study 2. Age at cardiovascular disease onset, dementia risk, and the role of lifestyle factors 3. Associations Between Onset Age of Orthostatic Hypotension and Incident Myocardial Infarction, Stroke, and Dementia: A Prospective Cohort Study 4. Nonarteritic Anterior Ischemic Optic Neuropathy and the Risk of Dementia: A Nationwide Cohort Study 5. Early-Onset Ocular Motor Cranial Neuropathy Is a Strong Predictor of Dementia: A Nationwide, Population-Based Cohort Study 6. Diverticular disease and risk of dementia: a Danish population-based cohort study 7. Incidence and Risk Factors Epilepsy in Patients with Dementia: A Population-Based Study Using Regional Healthcare Databases in Umbria 8. Leisure-Time Television Viewing and Computer Use, Family History, and Incidence of Dementia 9. Age at Diagnosis of Atrial Fibrillation and Incident Dementia 10. Exploring the Association Between Opioid Use Disorder and Alzheimer's Disease and Dementia Among a National Sample of the U.S. Population 11. Negative Wealth Shock and Cognitive Decline and Dementia in Middle-Aged and Older US Adults 12. Acute Pancreatitis and the Risk of Dementia in Diabetes: A Nationwide Cohort Study Using Korean Healthcare Claims Database 13. Association Between Onset Age of Coronary Heart Disease and Incident Dementia: A Prospective Cohort Study 14. The Effect of Cataract Surgery on the Risk of Dementia: A Nationwide Cohort Study 15. Impact of Clinical Association Between Gout and Dementia: A Nationwide Population-Based Cohort Study in Korea 16. Positive Results from the Fecal Immunochemical Test Can Be Related to Dementia: A Nationwide Population-Based Study in South Korea 17. Risk factors for delayed-onset dementia after stroke or transient ischemic attack-A five-year longitudinal cohort study 18. Early-Onset Subgroup of Type 2 Diabetes and Risk of Dementia, Alzheimer's Disease and Stroke: A Cohort Study 19. Blood cholesterol and risk of dementia in more than 1·8 million people over two decades: a retrospective cohort study 20. Association Between Age at Diabetes Onset and Subsequent Risk of Dementia 21. Association Between Atrial Fibrillation and the Risk of Dementia in the Korean Elderly: A 10-Year Nationwide Cohort Study 22. Association between the severity of hearing loss and the risk of dementia within the 2010-2017 national insurance service survey in South Korea 23. Risk of dementia in stroke-free patients diagnosed with atrial fibrillation: data from a population-based cohort 24. Alcohol consumption and risk of dementia: 23 year follow-up of Whitehall II cohort study 25. Traumatic brain injury and the risk of dementia diagnosis: A nationwide cohort study 26. Midlife cardiovascular fitness and dementia: A 44-year longitudinal population study in women 27. High proportions of dementia among SLE patients: A big data analysis 28. Long-term risk of dementia among people with traumatic brain injury in Denmark: a population-based observational cohort study 29. Polycystic kidney disease is significantly associated with dementia risk 30. Female sex, early-onset hypertension, and risk of dementia 31. Dementia onset, incidence and risk in type 2 diabetes: a matched cohort study with the Fremantle Diabetes Study Phase I 32. Long-Term Risk of Dementia Among Survivors of Ischemic or Hemorrhagic Stroke 33. Marital status and risk of dementia: a nationwide population-based prospective study from Sweden 34. Depression with melancholic features is associated with higher long-term risk for dementia 35. Risk and prediction of dementia in patients with atrial fibrillation--a nationwide population-based cohort study 36. Effect of depression and diabetes mellitus on the risk for dementia: a national population-based cohort study 37. Association Between Atrial Fibrillation and Dementia in the General Population 38. Risk of dementia after anaesthesia and surgery 39. Atrial fibrillation is independently associated with senile, vascular, and Alzheimer's dementia 40. Postmenopausal hormone therapy and Alzheimer's disease risk: interaction with age 41. Late-life depression as a possible predictor of dementia: cross-sectional and short-term follow-up results 42. Association of dietary diversity, genetic susceptibility, and the risk of incident dementia: A prospective cohort study 43. Age, sex, and APOE gene-specific associations between dynapenic obesity and dementia in a large cohort 44. UK Biobank data demonstrate long-term exposure to floods is a risk factor for incident dementia 45. Age at cardiovascular disease onset, dementia risk, and the role of lifestyle factors 46. Age at diagnosis of diabetes, obesity, and the risk of dementia among adult patients with type 2 diabetes 47. Associations of early-onset coronary heart disease and genetic susceptibility with incident dementia and white matter hyperintensity: A prospective cohort study 48. Diagnosis of Anosmia in Middle Age, But Not in Older Adulthood, Increases Alzheimer Dementia Risk 49. Nonarteritic Anterior Ischemic Optic Neuropathy and the Risk of Dementia: A Nationwide Cohort Study 50. Type 2 diabetes and late-onset Alzheimer’s disease and related dementia: A longitudinal cohort study integrating polygenic risk score |
| Investigate factors influencing disease progression in people with young-onset dementia | 1. Predictors of Disease Progression in Early-Onset Alzheimer's Dementia: A Retrospective Cohort Study 2. The Progression of Dementia and Cognitive Decline in a Dutch 2-Year Cohort Study of People with Young-Onset Dementia 3. Predictive factors for disease progression in patients with early-onset Alzheimer's disease 4. Factors associated with mortality in patients with early-onset Alzheimer's disease: a five-year longitudinal study |
| Compare characteristics between young-onset and late-onset dementia | 1. Neuropsychiatric symptom burden in early-onset and late-onset Alzheimer's disease as a function of age 2. The role of ethnicity, biological sex, and psychotropic agents in early and late onset Alzheimer's disease 3. Clinico-demographics of people with younger-onset dementia and neuropsychiatric symptoms referred to an Australian dementia support service: A comparison study with older-onset dementia 4. The severity of neuropsychiatric symptoms is higher in early-onset than late-onset Alzheimer's disease 5. Sociodemographic and Clinical Characteristics of 1350 Patients With Young Onset Dementia: A Comparison With Older Patients 6. Progression of neuropsychiatric symptoms in young-onset versus late-onset Alzheimer's disease 7. Characteristics of Young-Onset and Late-Onset Dementia Patients at a Remote Memory Clinic 8. Early-versus Late-Onset Alzheimer Disease: Long-Term Functional Outcomes, Nursing Home Placement, and Risk Factors for Rate of Progression 9. Early- versus late-onset Alzheimer's disease in clinical practice: cognitive and global outcomes over 3 years 10. Cognitive and Neuroanatomical Correlates in Early Versus Late Onset Parkinson's Disease Dementia 11. Mortality Risk after Diagnosis of Early-Onset Alzheimer's Disease versus Late-Onset Alzheimer's Disease: A Propensity Score Matching Analysis 12. Comparing the Overall Health, Stress, and Characteristics of Canadians with Early-Onset and Late-Onset Dementia 13. Prevalence of Comorbidity in Patients With Young-Onset Alzheimer Disease Compared With Late-Onset: A Comparative Cohort Study 14. The Role of Ethnicity in Alzheimer's Disease: Findings From The C-PATH Online Data Repository 15. Cognitive profiles and neuropsychiatric symptoms in Korean early-onset Alzheimer's disease patients: a CREDOS study 16. Differentiated clinical presentation of early and late-onset Alzheimer's disease: is 65 years of age providing a reliable threshold? 17. Early-onset Alzheimer's disease: a global cross-sectional analysis 18. Differences in anxiety among patients with early- versus late-onset Alzheimer's disease 19. Differences in routine clinical practice between early and late onset Alzheimer's disease: data from the Swedish Dementia Registry (SveDem) 20. Differences between early and late onset Alzheimer's disease 21. Prevalence of neuropsychiatric symptoms in young-onset compared to late-onset Alzheimer's disease - part 1: findings of the two-year longitudinal NeedYD-study 22. Early onset dementia: characteristics in a large cohort from academic memory clinics 23. Early-versus late-onset Alzheimer's disease: more than age alone 24. Early-onset dementia: frequency and causes compared to late-onset dementia 25. Neuropsychological performance in early and late onset Alzheimer's disease: comparisons in a memory clinic population 26. A comparison of family history of psychiatric disorders among patients with early- and late-onset Alzheimer's disease |
| Individuals with other medical disorders | 1. The effect of sleep disorders on dementia risk in patients with traumatic brain injury: A large-scale cohort study 2. Predictive Factors for Conversion to Dementia in Individuals with Early-Onset Mild Cognitive Impairment 3. Clinical Markers May Identify Patients at Risk for Early Parkinson's Disease Dementia: A Prospective Study 4. Incidence and Risk Factors for Dementia in Type 2 Diabetes Mellitus: A Nationwide Population-Based Study in Korea 5. Onset of dementia is associated with age at menopause in women with Down's syndrome 6. Association between L-α glycerylphosphorylcholine use and delayed dementia conversion: A nationwide longitudinal study in South Korea |
| Insufficient data | 1. Pre-Diagnostic Symptoms of Young-Onset Dementia in the General Practice up to Five Years Before Diagnosis 2. Contribution of alcohol use disorders to the burden of dementia in France 2008-13: a nationwide retrospective cohort study 3. Hypertensive Disorders of Pregnancy Appear Not to Be Associated with Alzheimer's Disease Later in Life 4. Childhood mental ability and dementia 5. Is smoking associated with the risk of developing Alzheimer's disease? Results from three Canadian data sets 6. Risk factors in clinically diagnosed presenile dementia of the Alzheimer type: a case-control study in northern England 7. Clinical and epidemiological aspects of Alzheimer's disease with presenile onset: a case control study |
| Multigenerational studies assessing the associations of exposures in descendants and dementia in prior generations | 1. Externalizing Behaviors and Alzheimer's Disease and Any Dementia: A Multigeneration Cohort Study in Sweden 2. Attention-deficit/hyperactivity disorder and Alzheimer's disease and any dementia: A multi-generation cohort study in Sweden 3. Does Schizophrenia in Offspring Increase the Risk of Developing Alzheimer's Dementia |
| Incidence or prevalence studies | 1. Prevalence and Incidence Rates of Dementia: A Nationwide Population-Based Study of Electronic Health Records in Israel 2. Incidence of Young Onset Dementia in Waikato, New Zealand: A Population-Based Study 3. Incidence of Young Onset Dementia in Central Norway: A Population-Based Study 4. Incidence and distribution of subtypes of early-onset dementia in Japan: A nationwide analysis based on annual performance reports of the Medical Centers for Dementia 5. Prevalence and subtype distribution of early-onset dementia in Japan 6. Prevalence and correlates of psychotropic drug use in Dutch nursing home patients with young-onset dementia 7. The Prevalence and Subtypes of Young Onset Dementia in Central Norway: A Population-Based Study 8. Incidence of dementia in elderly Latin Americans: Results of the Maracaibo Aging Study 9. The prevalence and causes of younger onset dementia in Eastern Sydney, Australia 10. Multicenter population-based study on the prevalence of early onset dementia in Japan: vascular dementia as its prominent cause 11. Incidence and subtypes of early-onset dementia in a geographically defined general population 12. Frequency and causes of early-onset dementia in a tertiary referral center in Athens 13. Prevalence and causes of early-onset dementia in Japan: a population-based study 14. Incidence of early-onset dementias in Cambridgeshire, United Kingdom |

# Supplementary Table 4. Characteristics of studies investigating risk factors for the development of young-onset dementia by study design

| **Cohort studies (N = 26)** | | | | | | | | | | |
| --- | --- | --- | --- | --- | --- | --- | --- | --- | --- | --- |
| **Study** | **Location** | **Data source** | **Recruitment (period)** | | **Follow-up (y)** | **Cases/participants** | **Male, n/%** | **Cut-off age criterion** | **Exposures** | **Dementia identification** |
| Yang YS, 2025 | Korea | KNHIS database | 2009 | | 8.2 [8.0-8.5] | 4,495/964,536 | 0% | Diagnosis age <65 years | Obesity | ICD-10 codes and prescriptions |
| Yuan S, 2024 | UK | UK Biobank | 2006-2010 | | 12.4 [11.4, 13.0)] | 863/368,498 | NR | Diagnosis age <65 years | 14 digestive system diseases | ICD codes |
| Yoo JE, 2024 | Korea | KNHIS database | 2009 | | 8.7 ± 1.5 | Premenopausal women: 2,195/946,931;  Postmenopausal women: 7,002/674,420 | 0% | Diagnosis age <65 years | Depression, age at menarche, age at menopause | ICD-10 codes and prescriptions |
| Olié V, 2024 | France | FNHIIS database | 2010-2018 | | 9.0 ± 2.6 | 128/1,966,323 | 0% | NR | Hypertensive disorders of  pregnancy | Primary diagnosis for hospitalization |
| Hendriks S, 2024 | UK | UK Biobank | 2006-2010 | | 9.18 [4.05-12.09] | 485/356,052 | 159,016/44.7% | Diagnosis age <65 years | 38 potential risk factors identified based on previous systematic reviews | ICD codes |
| Clouston SAP, 2024 | USA | The parent WTC Health and Wellness Program | 2014-2016 | | 2015-2020 (period) | 228/5,010 | 4,573/91.3% | Diagnosis age <65 years | WTC exposure severity | The 2011 guidelines of the National Institute on Aging-Alzheimer’s Association workgroups |
| Chun MY, 2024 | Korea | KNHIS database | 2009 | | 11-year follow-up period | 2,368/421,229 | 206,289/49.0% | Diagnosis age <65 years | Hypertension, diabetes mellitus, atrial fibrillation, hyperlipidemia, and osteoporosis | ICD-10 codes and prescriptions |
| Shang X, 2023 | UK | UK Biobank | 2006-2010 | | 2006-2020/2021 (period) | 452/NR | NR | Diagnosis age <65 years | Sex | ICD codes |
| Li R, 2023 | UK | UK Biobank | 2006-2010 | | 11.9 [9.0, 12.9] | 502/257,345 | 115,570/44.9% | Diagnosis age <65 years | Socioeconomic status, lifestyle and other potential risk factors | ICD codes and prescriptions at baseline (identify prevalent dementia cases at baseline) |
| Hu Y, 2023 | Finland | The Finnish national registers | December 31, 1999 | | 2000-2014 (period) | Men: 345/90,260;  Women: 347/90,589 | 90,260/49.9% | Diagnosis age <65 years | AUD, CVD, and CVD risk factors (diabetes mellitus, hypertension, and hyperlipidemia) | ICD codes, records of medication expense reimbursement for AD or purchases of any anti-dementia medication |
| Shang X, 2022 | UK | UK Biobank | 2006-2010 | | 11.9 [11.2, 12.6] | 503/377,214 | NR | Diagnosis age <65 years | 27 chronic conditions and multimorbidity | ICD codes |
| Mahmoudi E, 2022 | USA | A national, private administrative claims from the Clinformatics DataMart (OptumInsight) | 2007-2017 | | 4-year follow-up period | Unmatched: 2,013/515,538;  Matched: 103/8,119 | NR | Diagnosis age <65 years | Multiple sclerosis | ICD codes |
| Vivanti G, 2021 | USA | Medicaid Analytic eXtract data | 2008 | | 5-year follow-up period | ASD only vs. GP: NR/152,654; ASD+ID vs. GP: NR/167,310; ID only vs. GP: NR/455,140 | 237,386/49.0% | Diagnosis age <65 years | ASD only, ID only, ASD+ID, any ASD, and covariates | ICD-9 codes |
| Mahmoudi E, 2021 | USA | A national, private administrative claims from the Clinformatics DataMart (OptumInsight) | 2007-2017 | | 4-year follow-up period | Unmatched: 1,873/513,594;  Matched: 49/4,544 | NR | Diagnosis age <65 years | Traumatic spinal cord injury | ICD codes |
| Osler M, 2020 | Denmark | Linkage of the Danish Conscription Database to the Danish national registers | 1957-1984 | | 39.3 [38.0-39.5] | 4,164/NR | 100% | Diagnosis age <60 years | Traumatic brain injury and covariates | ICD codes and individual patients’ refill of at least one redeemed prescription |
| Kivimäki M, 2020 | Finland | Pooled data from Health and Social Support and Finnish Public Sector studies | 1986-2012 | | Mean follow-up, 12.6 years | 181/95,946 | NR | Diagnosis age <65 years | Alcohol-induced loss of consciousness | ICD-10 codes |
| Kim D, 2020 | Korea | The KNHIS-Health Screening cohort | 2005-2010 | | 2005-2013 (period) | Unmatched: 1,112/419,322;  Matched:  138/39,023 | NR | Diagnosis age <65 years | Atrial fibrillation | ICD-10 codes and prescriptions |
| Han E, 2020 | Korea | KNHIS database | 2007-2008 | | Mean follow-up, 7.6 years | 19,915/NR | NR | Age at the time of study recruitment <60 years | γ-GT, NAFLD, glycemic status, diabetes treatment status | ICD-10 codes and prescriptions |
| Rantalainen V, 2018 | Finland | Linkage of the Helsinki Birth Cohort Study to the Hospital Discharge and Causes of Death Registers | 1952-1972 | | 1952-2013 (period) | 20/2,726 | 100% | Diagnosis age <65 years | Cognitive ability at 20.1 years | ICD codes |
| Basit S, 2018 | Denmark | The Danish national registers | 1978-2015 | | 21.1 [11.3-23.4] | 1,225/NR | 0% | Diagnosis age <65 years | Pre-eclampsia | ICD-10 codes |
| Bagge CN, 2018 | Denmark | The Danish national registers | 1963-2012 | | 1981-2012 (period) | 178/NR | NR | Diagnosis age <65 years | Congenital heart disease | ICD codes |
| Ribe AR, 2015 | Denmark | The Danish national registers | 1995-2013 | | 11.0 ± 6.0 | 8,750/NR | NR | Diagnosis age <65 years | Schizophrenia | ICD codes and/or prescriptions |
| Nyberg J, 2014 | Sweden | Linkage of the Swedish Military Service Conscription Register to Swedish national registers | 1968-2005 | | 25.7 ± 9.3 | Analysis for cardiovascular fitness: 662/1,174,483;  For cognitive performance:  657/1,172,190 | 100% | Diagnosis age <65 years | Cardiovascular fitness and cognitive performance at age 18 | ICD codes |
| Nordström P, 2014 | Sweden | Linkage of the Swedish conscription register to Swedish national registers | 1969-1986 | | Median follow-up, 33 years | 566/811,622 | 100% | Diagnosis age <65 years | Traumatic brain injury | ICD codes |
| Nordström P, 2013 | Sweden | Linkage of the Swedish Military Service Conscription Register to Swedish national registers | 1969-1979 | | Median follow-up, 37 years (range, 0-41) | 487/488,448 | 100% | Diagnosis age <65 years | Potential risk factors identified based on previous studies | ICD codes |
| Phung TK, 2010 | Denmark | The Danish national registers | NR | | 1977-2006 (period) | 4,046/2,313,388 | 0% | Diagnosis age <60 years | Hysterectomy and oophorectomy | ICD codes |
| **Case-control studies (N = 22)** | | | | | | | | | | |
| **Study** | **Location** | **Case source** | | **Control selection** | | **Controls/cases** | **Male, n/%** | **Young-onset definition** | **Exposures** | **Dementia identification** |
| Park D, 2025 | Korea | KNHIS database (2004-2019) | | KNHIS database (age-, sex- and Charlson comorbidity index-matched, 1:1) | | 5,157/5,157 | 4,648/45.1% | Diagnosis age <65 years | Modifiable risk factors | ICD-10 codes and prescriptions |
| Mazzoli R, 2024 | Italy | The two Cognitive Neurology Centers (2016-2019) | | The caregivers of dementia patients | | 54/58 | 48/42.9% | Age of symptom onset <65 years | Atrial fibrillation, CAS, diabetes, dyslipidemia, hypertension, MI, stroke, and VHD | The Diagnostic and Statistical Manual of Mental Disorders, 5th Edition |
| Damsgaard L, 2024 | Denmark | The Danish Quality Database for Dementia (2016-2020) | | The entire Danish population (age- and sex-matched, 1:3) | | 5,235/1,745 | 2,904/41.6% | Diagnosis age <70 years | Medication use | NR |
| Damsgaard L, 2024 | Denmark | The Danish Quality Database for Dementia (2016-2020) | | The entire Danish population (age- and sex-matched, 1:3) | | 5,235/1,745 | 2,904/41.6% | Diagnosis age <70 years | Morbidities | The 2011 guidelines of the National Institute on Aging-Alzheimer’s Association workgroups |
| Mazzoleni E, 2023 | Italy | The two Cognitive Neurology Centers (2017-2019) | | The caregivers of dementia patients | | 54/58 | 48/42.9% | Age of symptom onset <65 years | Outdoor artificial light at night | Evaluated by a neurologist |
| Damsgaard L, 2023 | Denmark | The Danish Quality Database for Dementia (2016-2018) | | The entire Danish population (age- and sex-matched, 1:3) | | 3,246/1,082 | 1,824/42.1% | Diagnosis age <70 years | Healthcare utilization | NR |
| Wieland DR, 2022 | China | The Taiwanese National Health Insurance Research Database (2006-2013) | | The Longitudinal Health Insurance Database (age-, sex-, and index date-matched, 1:1) | | NR | NR | Diagnosis age <65 years | Hypothyroidism, hyperthyroidism, acquired hypothyroidism | ICD-9-CM codes with a certificate of catastrophic illness, and ≥ 3 outpatient visits or 1 inpatient diagnosis followed by another inpatient or outpatient visit |
| Kuang TT, 2022 | China | The Taiwanese National Health Insurance Research Database (2010-2017) | | The remaining National Health Insurance beneficiaries (PSM using age, sex, monthly income, geographic location, urbanization level, and relevant medical comorbidities,1:3) | | 109,731/36,577 | 94,302/64.5% | Diagnosis age <65 years | Prior diagnosis of macular degeneration | ICD codes (a dementia diagnosis in at least two claims) |
| Kennedy E, 2022 | USA | National Veterans Affairs and Department of Defense health system data | | National Veterans Affairs and Department of Defense health system data (sociodemographic factors and military history-matched, 1:1) | | 973/973 | 1,642/84.4% | Diagnosis age <65 years | Traumatic brain injury | ICD codes |
| Cheng YF, 2021 | China | The Taiwanese National Health Insurance Research Database (2010-2016) | | The remaining National Health Insurance beneficiaries (PSM using age, sex, monthly income, geographic location, urbanization level, and relevant medical comorbidities,1:3) | | 1,308/1,308 | 1,022/39.1% | Diagnosis age <65 years | Prior diagnosis of tinnitus | ICD codes (a documented diagnosis of dementia at least two medical encounters) |
| Filippini T, 2020 | Italy | The two Cognitive Neurology Centers (2016-2019) | | The caregivers of dementia patients | | 54/54 | 47/43.5% | Age of symptom onset <65 years | Dietary patterns and dietary factors | NR |
| Adani G, 2020 | Italy | The two Cognitive Neurology Centers (2016-2019) | | The caregivers of dementia patients | | 54/58 | 48/42.9% | Age of symptom onset <65 years | A wide range of environmental risk factors | NR |
| Keret O, 2018 | Israel | Dementia patients of Jewish origin attending the Cognitive Neurology Clinic of Rabin Medical Center (1999-2016) | | Cognitively healthy Jewish subjects from the Second Israeli National Health Interview Survey (age- and geographically- matched, 2007-2010) | | 1,811/129 | 825/42.5% | Diagnosis age <65 years | Demographic and vascular risk factors | The 2011 guidelines of the National Institute on Aging-Alzheimer’s Association workgroups |
| Cations M, 2018 | Australia | Pooling cases from two cross-sectional studies (the INSPIRED and KGOW studies) | | Participants from the two studies who passed cognitive screening and were free from dementia or any condition at high risk for developing dementia | | 179/96 | 165/60.0% | Age of symptom onset <65 years | A wide range of non-genetic risk factors | NR |
| Kadohara K, 2017 | Japan | The Japan Medical Data Center database (2005-2015) | | The Japan Medical Data Center database (age at the index month-, sex-, and type of hospital-matched, 1:4) | | 1,484/371 | 965/52.0% | Age at first prescription of AD medication < 65 | Diabetes mellitus, prescribed drug use, and polypharmacy | Prescriptions |
| Chen Y, 2017 | France | The French Cohorte Malades Alzheimer Jeunes prospective cohort study (2009-2014) | | The French MONA-LISA population-based survey between 2005 and 2007 (age-, gender-matched, 1:3) | | 306/102 | 164/40.2% | Age of symptom onset <60 years | Lifestyle factors and vascular risk factors | The IWG-2 criteria |
| Mendez MF, 2015 | USA | The National Alzheimer’s Coordinating Center database (2005-2014) | | The National Alzheimer’s Coordinating Center database (sex-, and years of education-matched, 1:1) | | 1,449/1,449 | 593/40.8% | Age of symptom (cognitive decline) onset <65 years | Traumatic brain injury | NR |
| Wu KY, 2013 | China | The Taiwanese National Health Insurance Research Database (2000-2009) | | The Taiwanese National Health Insurance Research Database (age-, sex-matched, 1:6) | | 7,278/1,213 | NR | Diagnosis age <65 years | Bipolar disorder | ICD-9- CM codes |
| Slooter AJ, 1999 | The Netherlands | The four northern provinces of the Netherlands and metropolitan Rotterdam (1980-1987) | | The municipal population register (age- and place of residence-matched, 1:1) | | 119/109 | 0% | Age of onset < 65 years | Estrogen use | NINCDS-ADRDA criteria |
| van Duijn CM, 1995 | The Netherlands | The four northern provinces of the Netherlands and metropolitan Rotterdam (1980-1987) | | Original controls: the municipal population register (age- and place of residence-matched, 1:1)  Additional controls: the Rotterdam elderly study | | Original controls: 159  Additional controls: 457  Cases: 175 | 319/40.3% | Age of onset < 65 years | History of cigarette smoking | NINCDS-ADRDA criteria |
| van Duijn CM, 1992 | The Netherlands | The four northern provinces of the Netherlands and metropolitan Rotterdam (1980-1987) | | The municipal population register (age-, sex- and place of residence-matched, 1:1) | | 198/198 | 148/37.4% | Diagnosis age <70 years | Head trauma with loss of consciousness, family history of dementia in first-degree relatives, education | NINCDS-ADRDA criteria |
| van Duijn CM, 1991 | The Netherlands | The four northern provinces of the Netherlands and metropolitan Rotterdam (1980-1987) | | The municipal population register (age-, sex- and place of residence-matched, 1:1) | | 198/198 | 148/37.4% | Diagnosis age <70 years | Nicotine intake | NINCDS-ADRDA criteria |
| **Cross-sectional studies (N = 5)** | | | | | | | | | | |
| **Study** | **Location** | **Study period** | **Sample source** | | | **Sample size** | **Male, n/%** | **Young-onset definition** | **Exposures** | **Dementia identification** |
| Jaakkimainen L, 2022 | Canada | 2016 | The Electronic Medical Record Primary Care database | | | 6,398,483 | 3,084,321/48.2% | Diagnosis age <65 years | Comorbidities | An algorithm for identifying YOD |
| Downing KF, 2022 | USA | 2015-2017 | The IBM® MarketScan® Commercial Database | | | 3,954,327 | 1,812,335/45.8% | Diagnosis age <65 years | Congenital heart defects | ≥1 inpatient ICD-9-CM or ICD-10-CM dementia diagnosis codes or ≥2 outpatient dementia codes diagnosed on different days |
| Carcaillon-Bentata L, 2021 | France | 2016 | The French national health data system | | | NR | NR | Diagnosis age <65 years | Comorbidities | The presence of at least 1 of the following criteria:  1) ≥2 reimbursements over a 1-year period of anti-Alzheimer drugs  2) Long-term chronic diseases registration for dementia (ICD-10 codes)  3) ≥1 inpatient diagnosis of dementia (ICD-10 codes) |
| Heath CA, 2015 | UK | All patients who were alive and permanently registered with a participating practice on 31 March 2007 | A data set of 1,751,841 people registered with 314 general practices in Scotland, covering approximately one-third of the Scottish population | | | 616,245 | 310,999/50.5% | Diagnosis age <65 years | Vascular comorbidities | The presence ever of one of a specified set of Read Codes or the prescription ever of an anticholinesterase inhibitor |
| Draper B, 2011 | Australia | 2006-2007 | The New South Wales Admitted Patient Care Database | | | NR | NR | Diagnosis age <65 years | Selected principal diagnosis for hospital admission, selected principal procedures and interventions | ICD-10-AM codes |

Abbreviation: γ-GT, gamma-glutamyltransferase; AD, Alzheimer's disease; ASD, autism spectrum disorder; AUD, alcohol use disorder; CAS, carotid artery stenosis; CVD, cardiovascular disease; FNHIIS, French National Health Insurance Information System; GP, general population; ICD, International Classification of Disease; ICD-10, International Classification of Diseases, 10th Revision; ICD-10-AM, International Classification of Diseases, 10th Revision, Australian Modification; ICD-10-CM, International Classification of Diseases, 10th Revision, Clinical Modification; ICD-9, International Classification of Diseases, 9th Revision; ICD-9-CM, International Classification of Diseases, 9th Revision, Clinical Modification; ID, intellectual disability; INSPIRED, The ‘Improving Service Provision in Younger Onset Dementia’ study; KGOW, The ‘Koori Growing Old Well Study’; KNHIS, Korean National Health Insurance Service; MI, myocardial infarction; NAFLD, non-alcoholic fatty liver disease; NR, Not reported; PSM, propensity score matching; VHD, valvular heart disease; WTC, World Trade Center; YOD, young-onset dementia.

# Supplementary Table 5. Effect estimates of associations between risk factors and young-onset dementia risk by study design

| **Cohort studies (N = 26)** | | | | | | | | | | |
| --- | --- | --- | --- | --- | --- | --- | --- | --- | --- | --- |
| **Study** | **Effect metric** | **Outcome** | **Comparison between exposure strata** | | | | | **Cases/participants or PYs at risk** | **Effect estimates** | **Adjustments** |
| Yang YS, 2025 | HR | YOD | 1. WC (cm)   <75 (ref.)  <85  <95  ≥95   1. Per 1 cm increase in WC 2. BMI   <18.5  <23 (ref.)  <25  <30  ≥30 | | | | | 1. WC (cm)   1,320/413,249  2,048/398,540  905/129,472  222/23,275   1. NA 2. BMI   96/22,568  1,541/422,942  1,196/240,476  1,418/244,865  244/33,685 | 1. WC (cm)   1.00 (ref.)  1.13 (1.05-1.21)  1.24 (1.14-1.36)  1.55 (1.34-1.79)   1. 1.012 (1.008-1.016) 2. BMI   1.39 (1.13-1.71)  1.00 (ref.)  1.08 (1.00-1.17)  1.09 (1.01-1.17)  1.26 (1.10-1.45) | Age, smoking status, alcohol consumption, regular exercise, low household income, diabetes mellitus, hypertension, dyslipidemia, depression, atrial fibrillation and stroke |
|  |  | AD (YO) | 1. WC (cm)   <75 (ref.)  <85  <95  ≥95   1. Per 1 cm increase in WC 2. BMI   <18.5  <23 (ref.)  <25  <30  ≥30 | | | | | 1. WC (cm)   922/413,249  1,353/398,540  616/129,472  147/23,275   1. NA 2. BMI   63/22,568  1,063/422,942  804/240,476  954/244,865  154/33,685 | 1. WC (cm)   1.00 (ref.)  1.06 (0.98-1.16)  1.21 (1.09-1.35)  1.49 (1.25-1.78)   1. 1.010 (1.006-1.015) 2. BMI   1.32 (1.02-1.71)  1.00 (ref.)  1.05 (0.96-1.15)  1.07 (0.98-1.17)  1.19 (1.00-1.41) |  |
|  |  | VaD (YO) | 1. WC (cm)   <75 (ref.)  <85  <95  ≥95   1. Per 1 cm increase in WC 2. BMI   <18.5  <23 (ref.)  <25  <30  ≥30 | | | | | 1. WC (cm)   922/413,249  1,353/398,540  616/129,472  147/23,275   1. NA 2. BMI   12/22,568  249/422,942  232/240,476  273/244,865  57/33,685 | 1. WC (cm)   1.00 (ref.)  1.06 (0.98-1.16)  1.21 (1.09-1.35)  1.49 (1.25-1.78)   1. 1.010 (1.006-1.015) 2. BMI   1.07 (0.60-1.92) 1.00 (ref.)  1.30 (1.09-1.56)  1.27 (1.06-1.52)  1.65 (1.23-2.22) |  |
| Yuan S, 2024 | HR | YOD | 1. Gastroesophageal reflux disease (+ vs. -) 2. Gastritis and duodenitis (+ vs. -) 3. Celiac disease (+ vs. -) 4. Crohn’s disease (+ vs. -) 5. Ulcerative colitis (+ vs. -) 6. Intestinal diverticular disease (+ vs. -) 7. Irritable bowel syndrome (+ vs. -) 8. Peptic ulcer (+ vs. -) 9. Pancreatitis (+ vs. -) 10. Gallbladder disease (+ vs. -) 11. Non, alcoholic fatty liver disease (+ vs. -) 12. Cirrhosis (+ vs. -) 13. Appendicitis (+ vs. -) 14. Overall gastrointestinal cancer (+ vs. -) | | | | | NR | 1. 1.87 (1.57, 2.24) 2. 2.18 (1.81, 2.63) 3. 1.30 (0.62, 2.75) 4. 2.21 (1.22, 4.02) 5. 2.20 (1.39, 3.47) 6. 1.15 (0.91, 1.44) 7. 1.40 (1.07, 1.82) 8. 2.07 (1.57, 2.71) 9. 1.99 (1.15, 3.45) 10. 1.85 (1.45, 2.35) 11. 2.85 (2.06, 3.93) 12. 5.18 (3.67, 7.32) 13. 1.60 (1.03, 2.50) 14. 1.19 (0.73, 1.93) | Age, sex, Townsend deprivation index, educational attainment, BMI, physical activity, diet, smoking status, alcohol consumption, hypertension, stroke, family history of dementia, depression, and PRS |
| Yoo JE, 2024 | HR | YOD | Premenopausal women | | | | Depression (+ vs. -) | 233/31,336 vs. 1972/915,595 | 2.67 (2.32, 3.07) | Age, smoking status, regular exercise, alcohol consumption, BMI, hypertension, type 2 diabetes, dyslipidemia, chronic kidney disease, age at menarche, age at menopause, parity, breast feeding, oral contraceptive use, and hormone replacement therapy |
|  |  | AD (YO) |  |  |  |  |  | 163/31,336 vs. 1,254/915,595 | 3.05 (2.59, 3.59) |  |
|  |  | VaD (YO) |  |  |  |  |  | 39/31,336 vs. 457/915,595 | 2.03 (1.46, 2.82) |  |
|  |  | YOD | Premenopausal women without depression | | | | Age at menarche (y):  ≤12  13-14  15-16 (ref.)  >16 | 57/40,544  418/288,936  862/419,801 (ref.)  635/166,314 | 0.94 (0.72, 1.23)  0.87 (0.77, 0.98)  1 (ref.)  1.36 (1.22, 1.50) |  |
|  |  | AD (YO) |  |  |  |  |  | 41/40,544  255/288,936  528/419,801 (ref.)  430/166,314 | 1.14 (0.83, 1.57)  0.89 (0.76, 1.03)  1 (ref.)  1.46 (1.28, 1.66) |  |
|  |  | VaD (YO) |  |  |  |  |  | 9/40,544  113/288,936  212/419,801 (ref.)  123/166,314 | 0.54 (0.28, 1.06)  0.89 (0.71, 1.13)  1 (ref.)  1.15 (0.91, 1.44) |  |
|  |  | YOD | Premenopausal women with depression | | | | Age at menarche (y):  ≤12  13-14  15-16(ref.)  >16 | 6/1,319  54/8,912  92/14,181 (ref.)  71/6,924 | 0.96 (0.42, 2.20)  1.12 (0.80, 1.57)  1 (ref.)  1.16 (0.85, 1.59) |  |
|  |  | AD (YO) |  |  |  |  |  | 4/1,319  40/8,912  69/14,181 (ref.)  50/6,924 | 0.89 (0.32, 2.43)  1.14 (0.77, 1.68)  1 (ref.)  1.07 (0.74, 1.53) |  |
|  |  | VaD (YO) |  |  |  |  |  | 1/1,319  9/8,912  14/14,181 (ref.)  15/6,924 | 0.97 (0.13, 7.38)  1.15 (0.50, 2.66)  1 (ref.)  1.72 (0.83, 3.58) |  |
|  |  | YOD | Postmenopausal women | | | | Depression (+ vs. -) | 1,004/39,885 vs. 5,998/634,535 | 2.50 (2.34, 2.67) |  |
|  |  | AD (YO) |  |  |  |  |  | 750/39,885 vs. 4,260/634,535 | 2.63 (2.43, 2.85) |  |
|  |  | VaD (YO) |  |  |  |  |  | 122/39,885 vs. 985/634,535 | 1.85 (1.53, 2.23) |  |
|  |  | YOD | Postmenopausal women without depression | | | | Age at menopause (y):  <40  40-44  45-50  51-54 (ref.)  ≥55 | 98/8,083  320/28,964  1,635/180,459  3,447/367,975 (ref.)  498/49,054 | 1.67 (1.36, 2.04)  1.59 (1.42, 1.79)  1.19 (1.12, 1.26)  1 (ref.)  0.93 (0.85, 1.02) |  |
|  |  | AD (YO) |  |  |  |  |  | 74/8,083  234/28,964  1,184/180,459  2,415/367,975 (ref.)  353/49,054 | 1.83 (1.45, 2.31)  1.69 (1.48, 1.93)  1.24 (1.16, 1.33)  1 (ref.)  0.95 (0.84, 1.06) |  |
|  |  | VaD (YO) |  |  |  |  |  | 17/8,083  43/28,964  251/180,459  599/367,975 (ref.)  75/49,054 | 1.61 (0.99, 2.61)  1.19 (0.87, 1.63)  1.02 (0.88, 1.19)  1 (ref.)  0.85 (0.66, 1.08) |  |
|  |  | YOD | Postmenopausal women with depression | | | | Age at menopause (y):  <40  40-44  45-50  51-54 (ref.)  ≥55 | 21/605  66/1,982  296/11,566  555/22,630 (ref.)  66/3,102 | 1.62 (1.05, 2.50)  1.76 (1.36, 2.27)  1.24 (1.07, 1.42)  1 (ref.)  0.76 (0.59, 0.98) |  |
|  |  | AD (YO) |  |  |  |  |  | 16/605  44/1,982  217/11,566  425/22,630 (ref.)  48/3,102 | 1.64 (0.99, 2.70)  1.56 (1.14, 2.12)  1.19 (1.01, 1.41)  1 (ref.)  0.73 (0.54, 0.98) |  |
|  |  | VaD (YO) |  |  |  |  |  | 3/605  8/1,982  42/11,566  58/22,630 (ref.)  11/3,102 | 2.19 (0.69, 6.98)  2.00 (0.95, 4.19)  1.64 (1.11, 2.45)  1 (ref.)  1.28 (0.67, 2.44) |  |
| Olié V, 2024 | HR | YOD | Without hypertensive disorders of pregnancy (ref.)  Gestational hypertension  Pre-eclampsia  Severe preeclampsia  Early preeclampsia  Superimposed on chronic hypertension | | | | | 108/NR (ref.)  11/NR  9/NR  2/NR  3/NR  3/NR | 1 (ref.)   - 1. (0.78, 2.71)   2. (1.34, 5.22)   1.45 (0.36, 5.88)  4.15 (1.3, 13.14)  4.76 (1.49-15.22) | Obesity, diabetes, tobacco smoking, drug or alcohol addiction, and social deprivation |
| Hendriks S, 2024 | HR | YOD | Socio-demographic factors   1. Educational attainment   Lower degree (ref.)  Academic or other professional degree   1. Socioeconomic status   Low deprivation (ref.)  Middle deprivation  High deprivation   1. Gender   Female (ref.)  Male | | | | | 1. Educational attainment   166/172,713 (ref.)  299/176,544   1. Socioeconomic status   58/70,014 (ref.)  232/212,256  194/73,301   1. Gender   234/197,036 (ref.)  251/159,016 | 1. Educational attainment   1 (ref.)  0.63 (0.48-0.81)   1. Socioeconomic status   1 (ref.)  1.26 (0.85-1.88)  1.82 (1.19-2.77)   1. Gender   1 (ref.)  1.25 (0.98-1.59) | 1) and 2) Educational attainment, socioeconomic status, APOE-status, alcohol use, alcohol use disorder, social isolation, Vitamin D, C-reactive protein, eGFR function, stroke, diabetes, hypoglycemia, heart disease, orthostatic hypotension, depression, hearing impairment, handgrip strength  3) Educational attainment, socioeconomic status, APOE-status, alcohol use, alcohol use disorder, social isolation, Vitamin D, C-reactive protein, eGFR function, stroke, hypertension, diabetes, hypoglycemia, heart disease, BMI, orthostatic hypotension |
|  |  |  | Lifestyle factors   1. Physical activity   Low level (ref.)  High level   1. Alcohol use   Abstainers (ref.)  Moderate  Heavy   1. Alcohol use disorder 2. Smoking   Never (ref.)  Former  Current   1. Diet   Unhealthy diet (ref.)  Healthy diet   1. Cognitive activity   Low level (ref.)  High level   1. Social isolation   Yes (ref.)  No   1. Marriage   Not married (ref.)  Married | | | | | 1. Physical activity   NR (ref.)  295/255,194   1. Alcohol use   NR (ref.)  206/172,156  188/148,837   1. 30/2,156 2. Smoking   NR (ref.)  133/115,083  110/40,095   1. Diet   NR (ref.)  193/166,168   1. Cognitive activity   NR (ref.)  19/16,362   1. Social isolation   NR (ref.)  333/269,726   1. Marriage   NR (ref.)  274/257,244 | 1. Physical activity   1 (ref.)  0.81 (0.63-1.05)   1. Alcohol use   1 (ref.)  0.72 (0.52-1.00)  0.64 (0.46-0.90)   1. 2.39 (1.37-4.15) 2. Smoking   1 (ref.)  0.86 (0.63-1.18)  1.12 (0.75-1.67)   1. Diet   1 (ref.)  0.84 (0.70-1.02)   1. Cognitive activity   1 (ref.)  1.07 (0.67-1.73)   1. Social isolation   1 (ref.)  1.53 (1.18-1.98)   1. Marriage   1 (ref.)  1.18 (0.82-1.70) | 5), 6), and 10) Educational attainment, socioeconomic status, APOE-status, alcohol use, alcohol use disorder, social isolation, Vitamin D, C-reactive protein, eGFR function, stroke, diabetes, hypoglycemia, heart disease, orthostatic hypotension, depression, hearing impairment, handgrip strength  4) Educational attainment, socioeconomic status, sex, APOE-status, alcohol use, alcohol use disorder, social isolation, Vitamin D, C-reactive protein, eGFR function  7) and 11) Sex, APOE status, physical activity, alcohol use, alcohol use disorder, smoking, social isolation, and marriage  8) and 9) Sex and APOE status |
|  |  |  | Environmental factors   1. Nitrogen oxide, μg/m^3^ 2. Particulate matter, μg/m^3^ 3. Diesel   Never (ref.)  Sometimes  Often   1. Overall pesticide   Never (ref.)  Sometimes or often | | | | | 1. NA 2. NA 3. Diesel   NR (ref.)  NR  NR   1. Overall pesticide   NR (ref.)  0/1723 | 1. 1.00 (0.99-1.01) 2. 1.08 (0.97-1.20) 3. Diesel   1 (ref.)  0.89 (0.21-3.88)  1.69 (0.22-12.92)   1. NA | 12) Sex, APOE status, and particulate matter  13) Educational attainment, socioeconomic status, sex, APOE-status, physical activity, alcohol use, alcohol use disorder, and social isolation  14) Sex and APOE status |
|  |  |  | Blood markers   1. Vitamin D   >20 ng/mL (ref.)  10-20 ng/mL  <10 ng/mL   1. C-reactive protein   <1 mg/dL (ref.)  >1 mg/dL   1. eGFR function   ≥90 mL/min/1.73 m^2^ (ref.)  60-90 mL/min/1.73 m^2^  <60 mL/min/1.73 m^2^   1. Albumin   no albuminuria (ref.)  Microalbumin  Microalbumin | | | | | 1. Vitamin D   NR (ref.)  170/135,487  107/54,894   1. C-reactive protein   NR (ref.)  72/37,168   1. eGFR function   NR (ref.)  153/112,785  36/11,387   1. Albumin   NR (ref.)  30/13,331  7/1,233 | 1. Vitamin D   1 (ref.)  1.10 (0.82-1.47)  1.59 (1.15-2.18)   1. C-reactive protein   1 (ref.)  1.54 (1.03-2.30)   1. eGFR function   1 (ref.)  1.02 (0.78-1.32)  1.55 (0.99-2.44)   1. Albumin   1 (ref.)  1.07 (0.68-1.68)  1.15 (0.36-3.73) | 16), 17) and 18) Educational attainment, socioeconomic status, APOE-status, alcohol use, alcohol use disorder, social isolation, Vitamin D, C-reactive protein, eGFR function, stroke, diabetes, hypoglycemia, heart disease, orthostatic hypotension, depression, hearing impairment, handgrip strength  19) Sex, APOE status, and Vitamin D, C-reactive protein, eGFR function |
|  |  |  | Cardiometabolic factors   1. Stroke (+ vs. -) 2. Hypertension (+ vs. -) 3. Diabetes (+ vs. -) 4. Hypoglycemia (+ vs. -) 5. Heart disease (+ vs. -) 6. Atrial fibrillation (+ vs. -) 7. Aspirin use (+ vs. -) 8. BMI   Normal (ref.)  Overweight (25-30)  Obesity (>30)   1. Orthostatic hypotension (+ vs. -) | | | | | 1. 29/4,325 (+) 2. 182/91,585 (+) 3. 59/16,604 (+) 4. 3/249 (+) 5. 46/11,773 (+) 6. 6/3,113 (+) 7. 91/39,857 (+) 8. BMI   NR (ref.)  154/131,323  124/67,984   1. 3/184 (+) | 1. 2.07 (1.26-3.41) 2. 1.13 (0.86-1.48) 3. 1.65 (1.15-2.36) 4. 2.25 (0.54-9.36) 5. 1.61 (1.10-2.36) 6. 1.00 (0.42-2.45) 7. 1.03 (0.75-1.42) 8. BMI   1 (ref.)  0.78 (0.59-1.04)  0.86 (0.63-1.17)   1. 4.20 (1.30-13.62) | 20), 22), 23) 24) and 28) Educational attainment, socioeconomic status, APOE-status, alcohol use, alcohol use disorder, social isolation, Vitamin D, C-reactive protein, eGFR function, stroke, diabetes, hypoglycemia, heart disease, orthostatic hypotension, depression, hearing impairment, handgrip strength  21) and 27) Educational attainment, socioeconomic status, sex, APOE-status, alcohol use, alcohol use disorder, social isolation, Vitamin D, C-reactive protein, eGFR function, stroke, hypertension, diabetes, hypoglycemia, heart disease, BMI, orthostatic hypotension  25) Sex and APOE status  26) Sex, APOE status, stroke, hypertension, diabetes, hypoglycemia, heart disease, BMI and orthostatic hypotension |
|  |  |  | Psychiatric factors   1. Depression (+ vs. -) 2. Anxiety (+ vs. -) 3. Benzodiazepine use (+ vs. -) 4. Delirium (+ vs. -) 5. Sleep problems   Never (ref.)  Sometimes  Usually | | | | | 1. 34/3,307 (+) 2. 28/31,522 (+) 3. 3/428 (+) 4. 1/34 (+) 5. Sleep problems   NR (ref.)  191/168,858  165/97,882 | 1. 3.25 (2.08-5.09) 2. 0.80 (0.52-1.21) 3. 2.57 (0.64-10.34) 4. NR 5. Sleep problems   1 (ref.)  0.86 (0.62-1.18)  1.16 (0.85 -1.60) | 29) Educational attainment, socioeconomic status, APOE-status, alcohol use, alcohol use disorder, social isolation, Vitamin D, C-reactive protein, eGFR function, stroke, diabetes, hypoglycemia, heart disease, orthostatic hypotension, depression, hearing impairment, handgrip strength  30), 31) and 33) Sex, APOE status, depression, anxiety, benzodiazepine use, delirium, sleep problems  32) Educational attainment, socioeconomic status, APOE-status, alcohol use, alcohol use disorder, social isolation, Vitamin D, C-reactive protein, eGFR function, stroke, diabetes, hypoglycemia, heart disease, orthostatic hypotension, and depression |
|  |  |  | Other factors   1. Traumatic brain injury 2. Rheumatoid arthritis 3. Thyroid dysfunction 4. Hearing impairment 5. Handgrip strength   Low (ref.)  Normal | | | | | 1. 0/42 2. 2/548 3. 0/2 4. 151/79,078 5. Handgrip strength   NA (ref.)  159/158,018 | 1. NA 2. 2.20 (0.55-8.84) 3. NA 4. 1.56 (1.22-2.01) 5. Handgrip strength   1 (ref.)  0.58 (0.52-0.88) | 35) Sex, APOE status  37), 38) Educational attainment, socioeconomic status, APOE-status, alcohol use, alcohol use disorder, social isolation, Vitamin D, C-reactive protein, eGFR function, stroke, diabetes, hypoglycemia, heart disease, orthostatic hypotension, depression, hearing impairment, handgrip strength |
| Clouston SAP, 2024 | HR | YOD | WTC Exposure Severity  Low exposure or no dust or wore PPE (ref.)  Mild exposure  Moderate exposure  High exposure  Severe exposure | | | | | 3/342 (ref.)  106/2805  76/1450  31/324  12/89 | 1.00 (ref.)  4.61 (1.63-13.07)  5.15 (1.78-14.9)  8.45 (2.80-25.48)  9.47 (2.70-33.14) | Age, sex, race and ethnicity, untrained responder, educational level, hypertension, diabetes, heart disease, history of head trauma, smoking status, history of heavy or binge drinking, and per-recruiter propensity for successful assessment |
| Chun MY, 2024 | HR | YOD | 1. Hypertension (+ vs. -) 2. Diabetes mellitus (+ vs. -) 3. Atrial fibrillation (+ vs. -) 4. Hyperlipidemia (+ vs. -) 5. Osteoporosis (+ vs. -) | | | | | 1. 1,057/115,954 vs. 620/115,954 2. 910/89,105 vs. 411/89,105 3. 54/5,269 vs. 177/26,345 4. 989/123,711 vs. 656/123,711 5. 417/49,302 vs 293/49,302 | 1. 1.15 (1.03-1.28) 2. 1.68 (1.47-1.92) 3. 0.86 (0.60-1.22) 4. 0.95 (0.85-1.07) 5. 1.18 (1.01-1.38) | Age, sex, hypertension, diabetes, atrial fibrillation, hyperlipidemia, osteoporosis, Charlson comorbidity index, socioeconomic status, and residential area |
| Shang X, 2023 | HR | YOD | Sex (male vs. female) | | | | | NR | 1.42 (1.18-1.71) | Age |
| Li R, 2023 | HR | YOD | 1. Age (continuous) 2. Male sex (vs. female) 3. Low socioeconomic status (vs. high) 4. Low education (vs. high) 5. Low income (vs. high) 6. Unemployed (vs. employed) 7. Hearing difficulty (vs. no) 8. Hypertension (vs. no) 9. Obesity (vs. no) 10. Healthy lifestyle score 0-1 (vs. score 3-4) 11. Healthy lifestyle score 2 (vs. score 0-1)^*^ 12. Healthy lifestyle score 3-4 (vs. score 0-1)^*^ 13. Excessive alcohol consumption (vs. no) 14. Smoking (vs. never) 15. Physical inactivity (vs. physical activity) 16. Unhealthy diet (vs. healthy diet) 17. Depression (vs. no) 18. Social isolation (vs. no) 19. Diabetes (vs. no) 20. Air pollution (vs. low) | | | | | NR | 1. 1.17 (1.14–1.19) 2. 1.54 (1.29–1.84) 3. 4.40 (3.43–5.65) 4. 3.76 (2.90–4.87) 5. 5.89 (4.54–7.65) 6. 5.06 (4.21–6.08) 7. 1.82 (1.53–2.17) 8. 1.50 (1.25–1.80) 9. 1.42 (1.13–1.78) 10. 2.20 (1.77–2.75) 11. 0.71 (0.56, 0.90)^*^ 12. 0.61 (0.49, 0.77)^*^ 13. 1.55 (1.30–1.86) 14. 1.46 (1.22–1.74) 15. 1.28 (1.04–1.57) 16. 1.31 (1.10–1.57) 17. 2.97 (2.37–3.73) 18. 1.53 (1.28–1.82) 19. 3.05 (2.35–3.94) 20. 1.56 (1.26–1.93) | Age and sex, with the exception of individual effects for age and sex, which were mutually adjusted  ^*^Age, sex, self-reported race, study center, social isolation, sleep quality, depressive symptoms, APOE4 carriers, BMI, HDL-C, LDL-C, triglycerides, HbA_1c_, prevalent comorbidities (hearing impairment, hypertension, and cardiovascular disease), family history of dementia, and socioeconomic status |
| Hu Y, 2023 | HR | YOD | Men | | 1. Alcohol use disorders (+ vs. -) 2. Diabetes (+ vs. -) 3. Hypertension (+ vs. -) 4. Hyperlipidemia (+ vs. -) 5. Cardiovascular disease (+ vs. -) | | | 1. 93/7,166 vs. 252/83,130 2. NR 3. NR 4. NR 5. NR | 1. 5.49 (4.23, 7.13) 2. 1.18 (0.86, 1.62) 3. 0.91 (0.71, 1.16) 4. 0.84 (0.63, 1.11) 5. 1.43 (1.12, 1.83) | Calendar year, time invariant region, education, household income, labor-force status, living arrangement, and occupational social class, time-varying hypertension, hyperlipidemia, diabetes, and cardiovascular disease |
|  |  |  | Women | |  |  |  | 1. 35/2,175 vs. 312/88,414 2. NR 3. NR 4. NR 5. NR | 1. 6.08 (4.15-8.89) 2. 1.34 (0.95-1.90) 3. 0.84 (0.67-1.05) 4. 1.17 (0.91-1.52) 5. 1.11 (0.87-1.43) |  |
| Shang X, 2022 | HR | YOD | 1. Hearing impairment (+ vs. -) 2. Obesity (+ vs. -) 3. Hypertension (+ vs. -) 4. High cholesterol (+ vs. -) 5. Coronary heart disease (+ vs. -) 6. Heart failure (+ vs. -) 7. Other cardiac problem (+ vs. -) 8. Stroke (+ vs. -) 9. Diabetes (+ vs. -) 10. Chronic obstructive pulmonary disease (+ vs. -) 11. Asthma (+ vs. -) 12. Depression (+ vs. -) 13. Anxiety (+ vs. -) 14. Schizophrenia (+ vs. -) 15. Parkinson's disease (+ vs. -) 16. Multiple Sclerosis (+ vs. -) 17. Alcohol problems (+ vs. -) 18. Other psychoactive substance abuse (+ vs. -) 19. Epilepsy (+ vs. -) 20. Painful conditions (+ vs. -) 21. Connective tissue disorders (+ vs. -) 22. Osteoporosis (+ vs. -) 23. Fracture (+ vs. -) 24. Dyspepsia (+ vs. -) 25. Chronic kidney disease (+ vs. -) 26. Diverticular disease (+ vs. -) 27. Chronic liver disease (+ vs. -) 28. Multimorbidity   Zero  One  Two  Three  Four  Five  ≥Six | | | | | 1. 274/2,361,183 (PY) vs. 230/3,210,302 2. 155/1,340,239 vs. 349/4,231,563 3. 157/1,432,724 vs. 347/4,139,079 4. 70/667,018 vs. 434/4,904,785 5. 38/245,047 vs. 5,326,756 6. 2/3,483 vs. 502/5,568,320 7. 2/17,801 vs. 502/5,554,002 8. 33/71,605 vs. 471/5,500,198 9. 43/214,790 vs. 461/5,357,013 10. 7/18,603 vs. 497/5,553,200 11. 93/645,062 vs. 411/4,926,740 12. 93/315,729 vs. 411/5,256,074 13. 22/76,591 vs. 482/5,495,212 14. 15/21,023 vs. 489/5,550,780 15. 12/7,219 vs. 492/5,564,584 16. 7/20,407 vs. 497/5,551,396 17. 10/9,082 vs. 494/5,562,720 18. 2/1,058 vs. 502/5,570,744 19. 20/45,240 vs. 484/5,526,562 20. 77/544,393 vs. 433/5,027,409 21. 19/123,229 vs. 485/5,448,573 22. 53/458,884 vs. 451/5,112,919 23. 4/8,013 vs. 500/5,563,790 24. 54/435,764 vs. 450/5,136,039 25. 4/13,813 vs. 500/5,557,990 26. 4/61,908 vs. 500/5,509,895 27. 4/10,969 vs. 500/5,560,834 28. 63/1,279,629 (ref.)   123/1,740,689  111/1,233,316  69/707,360  63/358,158  26/160,967  49/91,683 | 1. 1.65 (1.38-1.98) 2. 1.44 (1.06-1.96) 3. 1.43 (1·16-1.77) 4. 1.33 (1.02-1.73) 5. 1.66 (1.17-2.37) 6. 4.95 (1.23-19.99) 7. 1.23 (0.31-4.95) 8. 4.06 (2.80-5.88) 9. 1.84 (1.25-2.69) 10. 2.92 (1.37-6.22) 11. 1.51 (1.20-1.90) 12. 2.59 (2.05-3.28) 13. 2.24 (1.45-3.45) 14. 3.77 (2.23-6.38) 15. 20.67 (11.56-36.96) 16. 3.12 (1.48-6.59) 17. 3.20 (1.66-6.17) 18. 4.95 (1.22-20.15) 19. 2.97 (1.88-4.67) 20. 1.34 (1.04-1.72) 21. 1.72 (1.09-2.74) 22. 1.40 (1.04-1.88) 23. 5.10 (1.90-13.67) 24. 1.32 (0.99-1.76) 25. 2.39 (0.89-6.41) 26. 0.83 (0.31-2.24) 27. 2.74 (1.02-7.38) 28. 1.00 (ref.)   1.65 (1.21-2.23)  2.36 (1.71-3.25)  2.83 (1.97-4.07)  5.27 (3.60-7.73)  4.83 (2.95-7.90)  14.64 (9.52-22.51) | Age, gender, education, income, BMI, smoking, physical activity, alcohol consumption, sleep duration, diet, blood pressure, HDL-C, LDL-C, triglycerides, and HbA_1c_ |
| Mahmoudi E, 2022 | HR | ADRD (YO) | Unmatched Cohort: multiple sclerosis (+ vs. -)  Matched Cohort: multiple sclerosis (+ vs. -) | | | | | Unmatched Cohort: 87/4,186 vs. 1,926/511,352  Matched Cohort: 86/4,078 vs. 17/4,041 | Unmatched Cohort: 4.25 (3.40-5.32)  Matched Cohort: 4.49 (2.62-7.69) | Sex, race/ethnicity, U.S. Census Division, Elixhauser comorbidity index, education, net worth; PSM using age, sex, race/ethnicity, chronic conditions including cardiometabolic, psychologic, and musculoskeletal, U.S. Census Division, and socioeconomic variables |
| Vivanti G, 2021 | HR | YOD | 1. ASD Only vs. GP 2. Age at First Enrollment 3. Female vs. male 4. Race/Ethnicity   White (ref)  Black  Asian/Pacific Islander  Hispanic/Latino  Other   1. Urbanicity   Urban (ref)  Suburban  Rural  Missing   1. Medicaid Enrollment Category   Poverty  Disability (ref)  Other   1. Depression (+ vs. -) 2. Any Other Mental Disorders (+ vs. -) 3. Cardiovascular Disease Risk Factors (+ vs. -) 4. State | | | | | NR | 1. 1.96 (1.70, 2.28) 2. 1.096 (1.090, 1.103) 3. 0.77 (0.71, 0.84) 4. Race/Ethnicity   1 (ref.)  1.05 (0.95, 1.16)  1.02 (0.79, 1.31)  0.88 (0.76, 1.02)  0.94 (0.78, 1.12)   1. Urbanicity   1 (ref.)  0.83 (0.72, 0.95)  0.95 (0.82, 1.11)  0.39 (0.10, 1.58)   1. Medicaid Enrollment Category   0.41 (0.31, 0.54)  1 (ref.)  0.35 (0.28, 0.45)   1. 2.05 (1.88, 2.24) 2. 2.55 (2.28, 2.86) 3. 1.76 (1.59, 1.95) 4. NA | Age, sex, race/ethnicity, urbanicity status, Medicaid eligibility category, depression, any other psychiatric condition, cardiovascular disease risk factors, and state |
|  |  |  | 1. ASD+ID vs. GP 2. Age at First Enrollment 3. Female vs. male 4. Race/Ethnicity   White (ref)  Black  Asian/Pacific Islander  Hispanic/Latino  Other   1. Urbanicity   Urban (ref)  Suburban  Rural  Missing   1. Medicaid Enrollment Category   Poverty  Disability (ref)  Other   1. Depression (+ vs. -) 2. Any Other Mental Disorders (+ vs. -) 3. Cardiovascular Disease Risk Factors (+ vs. -) 4. State | | | | |  | 1. 2.89 (2.63, 3.17) 2. 1.083 (1.078, 1.089) 3. 0.81 (0.75, 0.88) 4. Race/Ethnicity   1 (ref.)  1.06 (0.97, 1.16)  1.16 (0.93, 1.46)  0.92 (0.8, 1.05)  0.95 (0.81, 1.12)   1. Urbanicity   1 (ref.)  0.87 (0.77, 0.98)  0.86 (0.75, 0.99)  0.61 (0.23, 1.62)   1. Medicaid Enrollment Category   0.36 (0.27, 0.47)  1 (ref.)  0.35 (0.28, 0.44)   1. 1.91 (1.77, 2.06) 2. 2.50 (2.25, 2.77) 3. 1.69 (1.55, 1.84) 4. NA |  |
|  |  |  | 1. ID only vs. GP 2. Age at First Enrollment 3. Female vs. male 4. Race/Ethnicity   White (ref)  Black  Asian/Pacific Islander  Hispanic/Latino  Other   1. Urbanicity   Urban (ref)  Suburban  Rural  Missing   1. Medicaid Enrollment Category   Poverty  Disability (ref)  Other   1. Depression (+ vs. -) 2. Any Other Mental Disorders (+ vs. -) 3. Cardiovascular Disease Risk Factors (+ vs. -) 4. State | | | | |  | 1. 3.01 (2.87, 3.15) 2. 1.078 (1.075, 1.080) 3. 0.99 (0.96, 1.02) 4. Race/Ethnicity   1 (ref.)  0.99 (0.95, 1.03)  1.06 (0.93, 1.22)  0.97 (0.9, 1.04)  0.95 (0.88, 1.03)   1. Urbanicity   1 (ref.)  0.87 (0.83, 0.91)  0.89 (0.84, 0.94)  0.68 (0.5, 0.93)   1. Medicaid Enrollment Category   0.35 (0.28, 0.45)  1 (ref.)  0.4 (0.34, 0.48)   1. 1.76 (1.71, 1.82) 2. 2.14 (2.05, 2.23) 3. 1.21 (1.17, 1.25) 4. NA |  |
|  |  |  | Any ASD vs. GP | | | | |  | 2.62 (2.40 2.85) |  |
| Mahmoudi E, 2021 | HR | ADRD (YO) | Unmatched Cohort: spinal cord injury (+ vs. -)  Matched Cohort: spinal cord injury (+ vs. -) | | | | | Unmatched Cohort: 36/2,474 vs. 1,837/511,120  Matched Cohort: 33/2,317 vs. 16/2,227 | Unmatched Cohort: 3.19 (2.30-4.44)  Matched Cohort: 1.93 (1.06-3.51) | Sex, race/ethnicity, U.S. Census Division, Elixhauser comorbidity index, education, net worth; PSM using age, sex, race/ethnicity, chronic conditions including cardiometabolic, psychologic, and musculoskeletal, U.S. Census Division, and socioeconomic variables |
| Osler M, 2020 | HR | YOD | 1. Traumatic brain injury (+ vs. -) 2. Cognitive ability   Lowest tertile (ref.)  Middle tertile  Highest tertile   1. Education   Low (ref.)  Middle  High   1. Alcohol abuse 2. Depression 3. Fractures | | | | | NR | 1. 5.49 (4.97–6.06) 2. Cognitive ability   1.00 (ref.)  0.68 (0.63–0.73)  0.51 (0.46–0.56)   1. Education   1.00 (ref.)  0.98 (0.90–1.04)  0.88 (0.77–1.00)   1. 3.87 (3.22–4.66) 2. 1.87 (1.39–2.80) 3. 1.92 (1.78–2.06) | Traumatic brain injury, cognitive ability, education, alcohol abuse, depression, and fractures |
| Kivimäki M, 2020 | HR | YOD | No loss of consciousness and moderate consumption (1-14 U/week) [ref.]  Loss of consciousness irrespective of alcohol consumption | | | | | NR | 1.00 (ref.)  2.21 (1.46-3.34) | Age, sex, educational level, occupational position, and cohort |
| Kim D, 2020 | HR | YOD | Unmatched Cohort: atrial fibrillation (+ vs. -)  Matched Cohort: atrial fibrillation (+ vs. -) | | | | | Unmatched Cohort: 74/10,177 vs. 1,038/409,145  Matched Cohort: 69/ 9,550 vs. 69/29,473 | Unmatched Cohort: 3.81 (2.75–5.29)  Matched Cohort: 3.99 (2.70-5.89) | Age, sex, hypertension, diabetes mellitus, dyslipidemia, heart failure, previous myocardial infarction, peripheral artery disease, osteoporosis, chronic kidney disease, chronic obstructive pulmonary disease, malignant neoplasm, liver disease, CHA2DS2-VASc score, cardiovascular medications, economic status, alcohol consumption, smoking status, exercise habits, follow-up duration, BMI, systolic and diastolic blood pressure, blood glucose, total cholesterol, and blood hemoglobin level |
| Han E, 2020 | HR | YOD | 1. γ-GT   Q1 (ref.)  Q2  Q3  Q4   1. NAFLD (+ vs. -) 2. Glycemic status   Normoglycemia (ref.)  Impaired fasting glucose  Incident diabetes  Prevalent diabetes   1. Diabetes treatment status   Normoglycemia (ref.)  Impaired fasting glucose  Incident diabetes  Treatment maintenance  Treatment failure | | | | | 1. γ-GT   4,117/8,999,668 (ref.) [PY]  4,385/8,417,327  5,066/8,542,570  6,347/8,086,032   1. 5,513/7,968,320 vs. 14,402/26,077,278 2. Glycemic status   12,207/24,168,225 (ref.)  4,421/7,324,881  680/899,137  2,607/1,653,354   1. Diabetes treatment status   NR | 1. γ-GT   1.00 (ref.)   1. (0.96–1.04)   1.01 (0.97–1.06)   - 1. 1.17–1.27)  1. 1.02 (0.98–1.06) 2. Glycemic status 3. (ref.)   1.04 (1.00; 1.07)  1.21 (1.12; 1.30)  1.73 (1.66; 1.81)   1. Diabetes treatment status   1.00 (ref.)  1.04 (1.01–1.07)  1.21 (1.12–1.30)  1.70 (1.62–1.78)  2.07 (1.83–2.34) | Age, sex, BMI, smoking status, alcohol use, exercise, hypertension, diabetes, dyslipidemia, myocardial infarction, stroke, and socioeconomic status |
| Rantalainen V, 2018 | HR | YOD | Cognitive ability at 20.1 years (per 1 SD disadvantage in cognitive ability)  Total  Verbal  Arithmetic  Visuospatial | | | | | NR | 1.77 (1.07–2.95)  1.69 (1.01–2.63)  1.63 (0.98–2.69)  1.57 (0.96–2.57) | Age at cognitive ability testing, father’s occupation in childhood, mother’s age at delivery, birthweight, and parity |
|  |  |  |  |  |  |  |  |  | 1.47 (0.85–2.55)  1.45 (0.85–2.48)  1.33 (0.78–2.27)  1.34 (0.80–2.23) | Age at cognitive ability testing and own highest attained education |
|  |  |  |  |  |  |  |  |  | 1.85 (1.12–3.03)  1.77 (1.07–2.94)  1.69 (1.03–2.94)  1.63 (1.03–2.65) | Age at cognitive ability testing and diagnoses of stroke or coronary heart disease |
| Basit S, 2018 | HR | YOD | Pre-eclampsia (+ vs. -) | | | | | 73/957 (PY) vs. 1,152/18,999 | 1.10 (0.87-1.40) | Maternal birth year, parity, region of most recent delivery, cardiovascular disease, stroke, chronic kidney disease, hypertension, and diabetes |
|  |  | VaD (YO) |  |  |  |  |  | 7/NR vs. 65/NR | 1.41 (0.64-3.10) |  |
|  |  | AD (YO) |  |  |  |  |  | 19/NR vs. 382/NR | 1.08 (0.68-1.72) |  |
|  |  | Other/unspecified dementia (YO) |  |  |  |  |  | 47/NR vs. 705/NR | 1.13 (0.84-1.52) |  |
| Bagge CN, 2018 | HR | YOD | Congenital heart disease (+ vs. -) | | | | | 33/NR vs. 145/NR | 2.59 (1.76-3.81) | Sex and birth year |
| Ribe AR, 2015 | IRR | YOD | Schizophrenia (+ vs. -) | | | | | 211/NR vs. 8,539/NR | 3.77 (3.29-4.33) | Age, sex, and calendar period |
| Nyberg J, 2014 | HR | YOD | 1. Cardiovascular fitness at age 18   High (ref.)  Medium  Low   1. Cognitive performance at age 18   High (ref.)  Medium  Low | | | | | NR | 1. Cardiovascular fitness   1.00 (ref.)  1.28 (1.08-1.53)  1.92 (1.43-2.58)   1. Cognitive performance   1.00 (ref.)  1.72 (1.35-2.20)  3.82 (2.96-4.93) | Calendar year, BMI, region, conscription test centre, parental education, cognitive performance at age 18, and cardiovascular fitness at age 18 |
| Nordström P, 2014 | HR | YOD | Traumatic brain injury  no traumatic brain injury (ref.)  1 mild traumatic brain injury  > 1 mild traumatic brain injury  ≥1 severe traumatic brain injury | | | | | Traumatic brain injury  NR (ref.)  59/NR  18/NR  25/NR | Traumatic brain injury  1.00 (ref.)  1.5 (1.1-2.0)  1.8 (1.1-3.0)  2.3 (1.5-3.6) | Age, place, year of conscription, overall cognitive function, alcohol intoxication, weight, height, knee extension strength, traumatic brain injury in parents, dementia in parents, income, educational level, systolic blood pressure, drug intoxication, depression, and cerebrovascular disease |
|  |  | AD (YO) | Traumatic brain injury  no traumatic brain injury (ref.)  1 mild traumatic brain injury  > 1 mild traumatic brain injury  ≥1 severe traumatic brain injury | | | | | Traumatic brain injury  NR (ref.)  8/NR  3/NR  1/NR | Traumatic brain injury  1.00 (ref.)  1.0 (0.5-2.0)  2.5 (0.8-8.1)  0.7 (0.1-5.2) |  |
|  |  | Other forms of dementia (YO) | Traumatic brain injury  no traumatic brain injury (ref.)  1 mild traumatic brain injury  > 1 mild traumatic brain injury  ≥1 severe traumatic brain injury | | | | | Traumatic brain injury  NR (ref.)  51/NR  15/NR  24/NR | Traumatic brain injury  1.00 (ref.)  1.7 (1.2-2.3)  1.7 (1.0-2.9)  2.6 (1.6-4.1) |  |
| Nordström P, 2013 | HR | YOD | 1. Year of conscription 2. Age at conscription (per 1-SD decrease) 3. Weight at conscription (per 1-SD decrease) 4. Height at conscription (per 1-SD decrease) 5. Knee muscle strength at conscription (per 1-SD decrease) 6. Systolic blood pressure at conscription (per 1-SD decrease) 7. Dementia in father, assessed at conscription 8. Dementia in mother, assessed at conscription 9. Overall cognitive function at conscription (per 1-SD decrease) 10. Elementary school education only, assessed 15 years after conscription 11. Total income per year, assessed 15 years after conscription (per 1-SD decrease) 12. Alcohol intoxication 13. Other drug intoxication 14. Depression or use of antidepressants 15. Myocardial infarction 16. Stroke 17. Neuroleptics 18. Antidiabetics | | | | | NR | 1. 1.05 (1.00-1.10) 2. 0.88 (0.79-0.99) 3. 0.95 (0.85-1.07) 4. 1.16 (1.04-1.29) 5. 1.02 (0.92-1.12) 6. 0.90 (0.82-0.99) 7. 1.65 (1.22-2.24) 8. 1.09 (0.79-1.50) 9. 1.26 (1.14-1.40) 10. 1.02 (0.83-1.25) 11. 1.03 (0.93-1.15) 12. 4.82 (3.83-6.05) 13. 1.54 (1.06-2.24) 14. 1.89 (1.53-2.34) 15. 0.99 (0.61-1.59) 16. 2.96 (2.02-4.35) 17. 2.75 (2.09-3.60) 18. 0.77 (0.55-1.09) | Year of conscription, age, weight, height, knee muscle strength, systolic blood pressure, dementia in father/mother, overall cognitive function, education, income, alcohol intoxication, other drug intoxication, depression or use of antidepressants, myocardial infarction, stroke, neuroleptics, and antidiabetics |
| Phung TK, 2010 | RR | YOD | Dementia diagnosed at ages 40 to 49 | 1. Hysterectomy (+ vs. -) 2. Ovarian status   Referent group (ref.)  Unilateral oophorectomy only  Bilateral oophorectomy only  Hysterectomy only  Hysterectomy and unilateral oophorectomy  Hysterectomy and bilateral oophorectomy | | | | 1. 96/583,340 (PY) vs. 1,012/10,377,474 2. Ovarian status   NR | 1. 1.58 (1.28-1.95) 2. Ovarian status   1.00 (ref.)  1.18 (0.71-1.96)  2.36 (0.76-7.34)  1.38 (1.07-1.78)  2.10 (1.28-3.45)  2.33 (1.44-3.77) | Calendar period and age at dementia diagnosis, both in 1-year groups |
|  |  |  | Dementia diagnosed at ages 50 to 59 | 1. Hysterectomy (+ vs. -) 2. Ovarian status   Referent group (ref.)  Unilateral oophorectomy only  Bilateral oophorectomy only  Hysterectomy only  Hysterectomy and unilateral oophorectomy  Hysterectomy and bilateral oophorectomy | | | | 1. 259/816,923 (PY) vs. 2,679/8,849,448 2. Ovarian status   NR | 1. 0.98 (0.86-1.11) 2. Ovarian status   1.00 (ref.)  1.08 (0.78-1.50)  0.75 (0.31-1.80)  0.89 (0.76-1.05)  0.88 (0.61-1.26)  1.27 (1.01-1.60) |  |
| **Case-control or cross-sectional studies (N = 27)** | | | | | | | | | | |
| **Study** | **Effect metric** | **Outcome** | **Comparison between exposure strata** | | | | | **Cases/controls** | **Effect estimates** | **Adjustments** |
| Park D, 2025 | OR | YOD | 1. BMI   Noral weight (ref.)  Underweight  Overweight  Obesity   1. SES   Low (ref.)  Middle  High   1. Urban residence 2. Alcohol intake   None (ref.)  Social  Heavy   1. Smoking   None (ref.)  Quitter  Current   1. Physical activity frequency 2. Systolic blood pressure 3. Diastolic blood pressure 4. Hemoglobin 5. Fasting blood sugar 6. Total cholesterol | | | | | NR | 1. BMI   1.00 (ref.)  1.284 (0.966-1.708)  0.985 (0.892-1.088)  0.875 (0.797-0.961)   1. SES   1.00 (ref.)  0.933 (0.838-1.039)  0.805 (0.725-0.893)   1. 0.942 (0.872-1.018) 2. Alcohol intake   1.00 (ref.)  0.879 (0.802-0.964)  1.149 (0.928-1.422)   1. Smoking   1.00 (ref.)  0.895 (0.762-1.051)  0.906 (0.806-1.020)   1. 0.969 (0.951-0.988) 2. 0.999 (0.997-1.001) 3. 0.998 (0.995-1.001) 4. 0.976 (0.944-1.009) 5. 1.000 (0.999-1.001) 6. 1.000 (0.999-1.001) | Age, sex and Charlson comorbidity index |
| Mazzoli R, 2024 | OR | YOD | 1. Atrial fibrillation (+ vs. -) 2. Atrial fibrillation^*^ (+ vs. -) 3. Carotid artery stenosis (+ vs. -) 4. Diabetes (+ vs. -) 5. Dyslipidemia (+ vs. -) 6. Hypertension (+ vs. -) 7. Myocardial infarction (+ vs. -) 8. Stroke (+ vs. -) 9. Stroke^#^ (+ vs. -) 10. Valvular heart disease (+ vs. -) | | | | | 1. 5/7 vs. 48/98 2. 5/7 vs. 48/98 3. 6/13 vs. 46/86 4. 9/12 vs. 40/88 5. 22/39 vs. 14/34 6. 19/41 vs. 20/30 7. 0/3 vs. 58/106 8. 4/5 vs. 50/102 9. 4/5 vs. 50/102 10. 1/2 vs. 56/108 | 1. 1.90(0.32-11.28) 2. 2.07 (0.35-12.27) 3. 0.79 (0.24-2.61) 4. 2.72 (0.67-10.97) 5. 1.43 (0.63-3.27) 6. 0.54 (0.23-1.24) 7. NA 8. 3.78 (0.39-36.76) 9. 4.01 (0.41-39.19) 10. 0.83 (0.04-15.22) | Age, sex, educational attainment, and smoking habits  ^*^Further adjusted for stroke  ^#^Further adjusted for Atrial fibrillation |
| Damsgaard L, 2024 | IRR | MCI or dementia due to AD (YO) | The entire 10-year period | 1. Alimentary tract and metabolism 2. Drugs for acid related disorders 3. Drugs for constipation 4. Antidiarrheals, intestinal anti-inflammatory agents etc. 5. Digestives, vitamins and mineral supplements 6. Drugs used in diabetes 7. Other alimentary tract and metabolism products 8. Blood and blood forming organs 9. Antithrombotic agents 10. Antianemic preparations 11. Other hematological agents 12. Cardiovascular system 13. Cardiac therapy 14. Antihypertensives 15. Diuretics 16. Beta blocking agents 17. Other cardiovascular system products 18. Dermatologicals 19. Antipruritics, incl. antihistamines, anesthetics, etc. 20. Antibiotics, chemotherapeutics for dermatological use 21. Corticosteroids, dermatological preparations 22. Other dermatological products 23. Genitourinary system and sex hormones 24. Sex hormones and modulators of the genital system 25. Urologicals 26. Other gynecologicals 27. Systemic hormonal preparations 28. Corticosteroids for systemic use 29. Thyroid therapy 30. Other systemic hormonal preparations 31. Anti-infectives for systemic use 32. Antibacterials for systemic use 33. Antimycotics for systemic use 34. Antivirals for systemic use 35. Vaccines 36. Antineoplastic and immunomodulating agents 37. Musculo-skeletal system 38. Antiinflammatory and antirheumatic products 39. Muscle relaxants 40. Drugs for treatment of bone diseases 41. Other musculo-skeletal products 42. Nervous system 43. Analgesics 44. Antiepileptics 45. Antipsychotics 46. Anxiolytics 47. Hypnotics and sedatives 48. Antidepressants 49. Other nervous system products 50. Antiparasitic products, insecticides, and repellents 51. Respiratory system 52. Nasal preparations 53. Drugs for obstructive airway diseases 54. Antihistamines for systemic use 55. Other respiratory system products 56. Sensory organs 57. Ophthalmologicals 58. Otologicals 59. Ophthalmologicals and otological preparations | | | | NR | 1. 1.04 (0.90-1.12) 2. 0.92 (0.82-1.03) 3. 1.48 (1.22-1.78) 4. 0.89 (0.71-1.10) 5. 1.15 (0.97-1.37) 6. 1.03 (0.86-1.24) 7. 1.04 (0.90-1.20) 8. 1.37 (1.24-1.54) 9. 1.27 (1.12-1.45) 10. 1.63 (1.38-1.93) 11. 0.77 (0.45-1.32) 12. 1.07 (0.95-1.20) 13. 0.92 (0.75-1.13) 14. 0.94 (0.84-1.06) 15. 0.83 (0.73-0.95) 16. 0.89 (0.76-1.03) 17. 1.06 (0.92-1.21) 18. 0.99 (0.89-1.11) 19. 1.12 (0.85-1.48) 20. 0.93 (0.81-1.06) 21. 0.96 (0.86-1.07) 22. 1.07 (0.95-1.21) 23. 1.05 (0.93-1.17) 24. 1.07 (0.93-1.24) 25. 1.04 (0.88-1.23) 26. 0.85 (0.63-1.16) 27. 0.93 (0.82-1.06) 28. 0.85 (0.74-0.97) 29. 1.13 (0.91-1.39) 30. 1.01 (0.55-1.86) 31. 0.98 (0.84-1.14) 32. 0.97 (0.84-1.13) 33. 0.93 (0.78-1.12) 34. 1.00 (0.83-1.20) 35. 0.83 (0.65-1.06) 36. 0.78 (0.51-1.13) 37. 0.99 (0.88-1.11) 38. 1.01 (0.90-1.13) 39. 0.95 (0.77-1.18) 40. 1.10 (0.88-1.39) 41. 0.94 (0.74-1.20) 42. 1.41 (1.25-1.60) 43. 0.96 (0.86-1.07) 44. 1.05 (0.88-1.26) 45. 2.08 (1.70-2.54) 46. 1.24 (1.07-1.44) 47. 1.06 (0.92-1.22) 48. 2.46 (2.19-2.78) 49. 1.22 1.03-1.44) 50. 0.93 (0.81-1.06) 51. 1.01 (0.90-1.12) 52. 0.93 (0.81-1.06) 53. 0.90 (0.78-1.03) 54. 1.07 (0.93-1.22) 55. 0.93 (0.82-1.05) 56. 1.00 (0.90-1-12) 57. 1.03 (0.92-1.15) 58. 0.90 (0.73-1.11) 59. 1.08 (0.88-1.33) | Age, sex, highest attained educational level at age 40 years (or at time of diagnosis, whichever came first), and civil status at index date |
|  |  |  | The 10->5-year interval prior to diagnosis | 1. Alimentary tract and metabolism 2. Blood and blood forming organs 3. Cardiovascular system 4. Dermatologicals 5. Genitourinary system and sex hormones 6. Systemic hormonal preparations 7. Anti-infectives for systemic use 8. Antineoplastic and immunomodulating agents 9. Musculo-skeletal system 10. Nervous system 11. Analgesics 12. Antiepileptics 13. Antipsychotics 14. Anxiolytics 15. Hypnotics and sedatives 16. Antidepressants 17. Other nervous system products 18. Antiparasitic products, insecticides, and repellents 19. Respiratory system 20. Sensory organs | | | |  | 1. 0.98(0.87-1.09) 2. 0.99 (0.86-1.14) 3. 1.05 (0.94-1.17) 4. 1.06 (0.95-1.18) 5. 1.06 (0.94-1.20) 6. 0.93 (0.86-1.16) 7. 1.04 (0.91-1.17) 8. 0.60 (0.38-0.97) 9. 1.03 (0.92-1.15) 10. 1.17 (1.05-1.31) 11. 1.00 (0.89-1.13) 12. 1.12 (0.89-1.41) 13. 1.20 (0.92-1.58) 14. 1.06 (0.89-1.26) 15. 1.02 (0.86-1.20) 16. 1.43 (1.25-1.64) 17. 1.22 (1.00-1.49) 18. 1.01 (0.87-1.19) 19. 1.01 (0.90-1.13) 20. 1.12 (1.00-1.25) |  |
|  |  |  | The 5->1-year interval prior to diagnosis | 1. Alimentary tract and metabolism 2. Blood and blood forming organs 3. Cardiovascular system 4. Dermatologicals 5. Genitourinary system and sex hormones 6. Systemic hormonal preparations 7. Anti-infectives for systemic use 8. Antineoplastic and immunomodulating agents 9. Musculo-skeletal system 10. Nervous system 11. Analgesics 12. Antiepileptics 13. Antipsychotics 14. Anxiolytics 15. Hypnotics and sedatives 16. Antidepressants 17. Other nervous system products 18. Antiparasitic products, insecticides, and repellents 19. Respiratory system 20. Sensory organs | | | |  | 1. 0.96 (0.86-1.07) 2. 1.27 (1.12-1.45) 3. 1.07 (0.95-1.19) 4. 0.89 (0.79-0.99) 5. 1.04 (0.92-1.18) 6. 0.91 (0.79-1.07) 7. 0.85 (0.76-0.96) 8. 1.00 (0.65-1.56) 9. 0.95 (0.85-1.06) 10. 1.20 (1.07-1.34) 11. 0.87 (0.78-0.98) 12. 1.05 (0.85-1.29) 13. 1.75 (1.37-2.24) 14. 1.17 (0.96-1.42) 15. 0.89 (0.74-1.07) 16. 2.27 (1.99-2.60) 17. 1.20 (0.97-1.50) 18. 0.85 (0.71-1.02) 19. 0.92 (0.82-1.03) 20. 0.87 (0.77-0.98) |  |
|  |  |  | The ≤1-year interval prior to diagnosis | 1. Alimentary tract and metabolism 2. Blood and blood forming organs 3. Cardiovascular system 4. Dermatologicals 5. Genitourinary system and sex hormones 6. Systemic hormonal preparations 7. Anti-infectives for systemic use 8. Antineoplastic and immunomodulating agents 9. Musculo-skeletal system 10. Nervous system 11. Analgesics 12. Antiepileptics 13. Antipsychotics 14. Anxiolytics 15. Hypnotics and sedatives 16. Antidepressants 17. Other nervous system products 18. Antiparasitic products, insecticides, and repellents 19. Respiratory system 20. Sensory organs | | | |  | 1. 0.95 (0.83-1.09) 2. 1.71 (1.48-1.97) 3. 1.08 (0.95-1.22) 4. 0.86 (0.73-1.02) 5. 0.72 (0.60-0.86) 6. 0.84 (0.68-1.04) 7. 0.94 (0.82-1.08) 8. 0.99 (0.54.1.83) 9. 0.79 (0.67-0.92) 10. 1.57 (1.39-1.78) 11. 0.86 (0.76-0.99) 12. 1.18 (0.90-1.54) 13. 3.45 (2.58-4.63) 14. 1.63 (1.24-2.12) 15. 0.83 (0.63-1.07) 16. 3.62 (3.09-4.24) 17. 1.18 (0.82-1.70) 18. 0.89 (0.62-1.30) 19. 0.62 (0.52-0.73) 20. 0.83 (0.68-1.00) |  |
| Damsgaard L, 2024 | IRR | MCI or dementia due to AD (YO) | The entire 10-year period | 1. Certain infections 2. Erysipelas 3. Other sepsis 4. Bacterial infection of unspecified site 5. Neoplasms 6. Benign neoplasm of colon etc. 7. Malignant neoplasm of breast 8. Benign lipomatous neoplasm 9. Hematological/immunological diseases 10. Other anaemias 11. Iron deficiency anaemia 12. Other coagulation defects 13. Endocrine/metabolic diseases 14. Disorders of lipoprotein metabolism etc. 15. Type 2 diabetes mellitus 16. Obesity 17. Mental and behavioral disorders 18. Alcohol-related mental disorders 19. Reaction to severe stress 20. Depressive episode 21. Organic, including symptomatic, mental disorders 22. Mental and behavioral disorders due to psychoactive substance use 23. Schizophrenia, schizotypal and delusional disorders 24. Mood/affective disorders 25. Neurotic, stress-related and somatoform disorders 26. Behavioral syndromes associated with psychological disturbances and physical factors 27. Disorders of adult personality and behavior 28. Unspecified mental disorder 29. Diseases of the nervous system 30. Mononeuropathies of arm 31. Sleep disorders 32. Transient cerebral ischaemic attacks 33. Diseases of the eye and adnexa 34. Senile cataract 35. Other cataract 36. Other retinal disorders 37. Diseases of the ear and mastoid process 38. Other hearing loss 39. Other disorders of ear 40. Conductive and sensorineural hearing loss 41. Diseases of the circulatory system 42. Essential hypertension 43. Angina pectoris 44. Chronic ischaemic heart disease 45. Diseases of the respiratory system 46. Chronic obstructive pulmonary disease 47. Pneumonia, organism unspecified 48. Asthma 49. Diseases of the digestive system 50. Diverticular disease of intestine 51. Other intestinal disorders 52. Inguinal hernia 53. Diseases of the skin/subcutaneous system 54. Cutaneous abscess etc. 55. Other local infections of skin 56. Psoriasis 57. Diseases of the musculoskeletal system 58. Gonarthrosis 59. Shoulder lesions 60. Internal derangement of knee 61. Diseases of the genitourinary system 62. Other disorders of urinary system 63. Cystitis 64. Female genital prolapse 65. Symptoms/signs not classified elsewhere 66. Abdominal and pelvic pain 67. Symptoms involving cognitive functions 68. Pain in throat and chest 69. Injuries, poisoning, and other external causes 70. Open wound of wrist and hand 71. Fracture of forearm 72. Open wound of head 73. Factors influencing health status etc. 74. Investigations without complaints 75. Observation 76. Screening for neoplasms | | | | NR | - 1. 1.01 (0.82-1.24)  1. 1.06 (0.69-1.63) 2. 1.00 (0.63-1.59) 3. 1.45 (0.84-2.51)    1. 1.03 (0.90-1.17) 4. 1.23 (0.98-1.54) 5. 1.06 (0.74-1.49) 6. 0.83 (0.53-1.31)    1. 1.22 (0.91-1.63) 7. 1.26 0.78-2.03 8. 1.36 0.73-2.52 9. 0.30 0.07-1.29    1. 1.38 (1.21-1.57)    2. 1.64 (1.35-1.99)    3. 1.20 (0.96-1.51)    4. 0.59 (0.43-0.81)    5. 3.18 (2.73-3.71) 10. 2.12 (1.62-2.79) 11. 2.77 (2.06-3.70) 12. 3.48 (2.54-4.77) 13. 8.05 (4.62-14.03) 14. 1.81 (1.43-2.29) 15. 2.28 (1.46-3.57) 16. 3.14 (2.48-3.98) 17. 2.69 (2.11-3.45) 18. 1.79 (0.65-4.95) 19. 1.61 (0.84-3.11) 20. 3.36 (1.81-6.25)     1. 1.52 (1.31-1.77) 21. 0.73 (0.51-1.05) 22. 1.02 (0.71-1.45) 23. 1.56 (1.05-2.31)     1. 1.20 (1.03-1.41) 24. 1.66 (1.31-2.12) 25. 1.03 (0.77-1.65) 26. 0.56 (0.32-0.97)     1. 1.45 (1.20-1.74) 27. 1.51 (1.20-1.90) 28. 1.30 (0.92-1.83) 29. 1.33 (0.91-1.95)     1. 1.26 (1.12-1.41) 30. 1.38 (1.19-1.60) 31. 1.23 (0.95-1.58) 32. 1.11 (0.85-1.44)     1. 1.12 (0.95-1.31) 33. 1.06 (0.79-1.41) 34. 1.07 (0.80-1.43) 35. 1.09 (0.77-1.54)     1. 1.01 (0.89-1.14) 36. 1.09 (0.84-1.43) 37. 1.33 (1.00-1.77) 38. 0.99 (0.72-1.37)     1. 1.17 (0.97-1.42) 39. 1.19 (0.77-1.85) 40. 1.43 (0.87-2.37) 41. 1.24 (0.70-2.19)     1. 0.92 (0.83-1.03) 42. 1.08 (0.88-1.33) 43. 0.92 (0.74-1.14) 44. 0.96 (0.75-1.24)     1. 1.10 (0.96-1.26) 45. 1.34 (1.01-1.78) 46. 1.39 (1.02-1.91) 47. 0.99 (0.69-1.42)     1. 2.45 (2.19-2.74) 48. 1.03 (0.85-1.25) 49. 90.84 (52.27-157.87) 50. 1.27 (0.98-1.64)     1. 1.31 (1.17-1.46) 51. 1.25 (1.02-1.53) 52. 1.45 (1.15-1.84) 53. 1.55 (1.20-1.99)     1. 8.80 (5.74-13.48) 54. 4.65 (3.63-5.96) 55. 2.59 (2.31-2.91) 56. 0.92 (0.78-1.06) | Age, sex, highest attained educational level at age 40 years (or at time of diagnosis, whichever came first), and civil status at index date |
|  |  |  | The 10->5-year interval prior to diagnosis | 1. Certain infections 2. Neoplasms 3. Hematological/immunological diseases 4. Endocrine/metabolic diseases 5. Mental and behavioral disorders   1) Organic, including symptomatic, mental disorders  2) Mental and behavioral disorders due to psychoactive substance use  3) Schizophrenia, schizotypal and delusional disorders  4) Mood/affective disorders  5) Neurotic, stress-related and somatoform disorders  6) Behavioral syndromes associated with psychological disturbances and physical factors  7) Disorders of adult personality and behavior  8) Unspecified mental disorder   1. Diseases of the nervous system 2. Diseases of the eye and adnexa 3. Diseases of the ear and mastoid process 4. Diseases of the circulatory system 5. Diseases of the respiratory system 6. Diseases of the digestive system 7. Diseases of the skin/subcutaneous system 8. Diseases of the musculoskeletal system 9. Diseases of the genitourinary system 10. Symptoms/signs not classified elsewhere 11. Injuries, poisoning, and other external causes 12. Factors influencing health status etc. | | | |  | 1. 0.81 (0.57-1.14) 2. 0.92 (0.76-1.11) 3. 1.41 (0.92-2.15) 4. 1.11 (0.93-1.31) 5. 1.43 (1.14-1.79) 6. 2.99 (0.86-10.33) 7. 1.20 (0.85-1.71) 8. 0.95 (0.49-1.83) 9. 1.45 (1.01-2.07) 10. 1.14 (0.77-1.69) 11. NR 12. 1.08 (0.42-2.76) 13. 2.09 (0.87-5.00) 14. 1.01 (0.81-1.26) 15. 1.03 (0.81-1.30) 16. 1.51 (1.16-1.97) 17. 1.05 (0.90-1.22) 18. 1.01 (0.80-1.28) 19. 1.05 (0.89-1.23) 20. 1.12 (0.85-1.48) 21. 1.00 (0.88-1.14) 22. 1.06 (0.89-1.26) 23. 1.19 (1.03-1.36) 24. 1.13 (1.00-1.27) 25. 1.07 (0.93-1.25) |  |
|  |  |  | The 5->1-year interval prior to diagnosis | 1. Certain infections 2. Neoplasms 3. Hematological/immunological diseases 4. Endocrine/metabolic diseases 5. Mental and behavioral disorders   1) Organic, including symptomatic, mental disorders  2) Mental and behavioral disorders due to psychoactive substance use  3) Schizophrenia, schizotypal and delusional disorders  4) Mood/affective disorders  5) Neurotic, stress-related and somatoform disorders  6) Behavioral syndromes associated with psychological disturbances and physical factors  7) Disorders of adult personality and behavior  8) Unspecified mental disorder   1. Diseases of the nervous system 2. Diseases of the eye and adnexa 3. Diseases of the ear and mastoid process 4. Diseases of the circulatory system 5. Diseases of the respiratory system 6. Diseases of the digestive system 7. Diseases of the skin/subcutaneous system 8. Diseases of the musculoskeletal system 9. Diseases of the genitourinary system 10. Symptoms/signs not classified elsewhere 11. Injuries, poisoning, and other external causes 12. Factors influencing health status etc. | | | |  | 1. 1.13 (0.86-1.48) 2. 1.00 (0.85-1.19) 3. 1.09 (0.71-1.66) 4. 1.06 (0.90-1.25) 5. 2.48 (2.02-3.04) 6. 3.88 (1.89-7.96) 7. 1.33 (0.93-1.91) 8. 1.88 (1.02-3.47) 9. 2.94 (2.11-4.11) 10. 3.40 (2.39-4.82) 11. 3.87 (1.04-14.48) 12. 0.93 (0.30-2.91) 13. 1.40 (0.43-4.57) 14. 1.05 (0.86-1.30) 15. 1.17 (0.96-1.43) 16. 1.45 (1.15-1.81) 17. 1.08 (0.94-1.25) 18. 1.01 (0.82-1.26) 19. 0.97 (0.83-1.13) 20. 1.06 (0.82-1.36) 21. 0.83 (0.73-0.95) 22. 1.08 (0.90-1.28) 23. 1.40 (1.24-1.59) 24. 1.35 (1.20-1.53) 25. 1.63 (1.39-1.92) |  |
|  |  |  | The ≤1-year interval prior to diagnosis | 1. Certain infections 2. Neoplasms 3. Hematological/immunological diseases 4. Endocrine/metabolic diseases 5. Mental and behavioral disorders   1) Organic, including symptomatic, mental disorders  2) Mental and behavioral disorders due to psychoactive substance use  3) Schizophrenia, schizotypal and delusional disorders  4) Mood/affective disorders  5) Neurotic, stress-related and somatoform disorders  6) Behavioral syndromes associated with psychological disturbances and physical factors  7) Disorders of adult personality and behavior  8) Unspecified mental disorder   1. Diseases of the nervous system 2. Diseases of the eye and adnexa 3. Diseases of the ear and mastoid process 4. Diseases of the circulatory system 5. Diseases of the respiratory system 6. Diseases of the digestive system 7. Diseases of the skin/subcutaneous system 8. Diseases of the musculoskeletal system 9. Diseases of the genitourinary system 10. Symptoms/signs not classified elsewhere 11. Injuries, poisoning, and other external causes 12. Factors influencing health status etc. | | | |  | 1. 1.24 (0.79-1.93) 2. 0.98 (0.78-1.25) 3. 1.71 (1.01-2.89) 4. 2.64 (2.17-3.21) 5. 8.19 (6.27-10.70) 6. 26.39 (8.00-87.03) 7. 4.57 (3.03-6.88) 8. 5.37 (2.62-11.01) 9. 9.17 (5.88-14.28) 10. 8.21 (4.62-14.59) 11. 2.93 (0.41-20.92) 12. 3.70 (0.99-13.84) 13. NR 14. 3.16 (2.48-4.03) 15. 1.45 (1.08-1.93) 16. 1.41 (1.01-1.98) 17. 2.14 (1.81-2.53) 18. 1.38 (1.05-1.81) 19. 0.98 (0.78-1.24) 20. 2.00 (1.37-2.94) 21. 1.02 (0.85-1.22) 22. 1.24 (0.94-1.62) 23. 5.69 (4.89-6.61) 24. 1.39 (1.16-1.66) 25. 7.01 (6.01-8.17) |  |
| Mazzoleni E, 2023 | OR | YOD | Nighttime luminance exposure (nW/cm^2^/sr)   1. Per 1-unit increase (continuous) 2. Per 10-unit increase (continuous)   Exposure-Median   1. < median (ref.) 2. > median   Exposure-Tertiles   1. T1 (ref.) 2. T2 3. T3   Exposure-Fixed categories   1. <10 (ref.) 2. 10-40 3. ≥40 | | | | | 1. NA 2. NA 3. 28/26 (ref.) 4. 30/28 5. 19/18 (ref.) 6. 21/18 7. 18/18 8. 15/15 (ref.) 9. 32/27 10. 11/12 | 1. 1.01 (0.98–1.03) 2. 1.05 (0.82–1.35) 3. 1.00 (ref.) 4. 1.10 (0.50–2.40) 5. 1.00 (ref.) 6. 1.25 (0.49–3.20) 7. 1.10 (0.42–2.90) 8. 1.00 (ref.) 9. 1.36 (0.54–3.39) 10. 1.04 (0.32–3.34) | Sex, age and level of education |
|  |  | AD (YO) |  |  |  |  |  | 1. NA 2. NA 3. 14/26 (ref.) 4. 18/28 5. 9/18 (ref.) 6. 11/18 7. 12/18 8. 7/15 (ref.) 9. 17/27 10. 8/12 | - 1. 1.02 (0.99–1.05)   2. 1.22 (0.91–1.65)   3. 1.00 (ref.)   4. 1.30 (0.52–3.29)   5. 1.00 (ref.)   6. 1.43 (0.46–4.52)   7. 1.69 (0.52–5.43)   8. 1.00(ref.)   9. 1.60 (0.51–4.99)   10. 1.89 (0.47–7.54) |  |
|  |  | FTD (YO) |  |  |  |  |  | - 1. NA   2. NA   3. 9/26 (ref.)   4. 10/28   5. 6/18 (ref.)   6. 7/18   7. 6/18   8. 4/15 (ref.)   9. 12/27   10. 3/12 | 1. 1.00 (0.96–1.03) 2. 0.98 (0.68–1.40) 3. 1.00 (ref.) 4. 1.07 (0.35–3.28) 5. 1.00 (ref.) 6. 1.20 (0.32–4.52) 7. 0.95 (0.23–3.83) 8. 1.00 (ref.) 9. 1.58 (0.41–6.10) 10. 0.77 (0.13–4.69) |  |
| Damsgaard L, 2023 | CRR | MCI or dementia due to AD (YO) | The 10->5-year interval prior to diagnosis | 1. Primary care 2. Any general practitioner contact 3. General practitioner face-to-face contacts 4. General practitioner telephone/email contacts 5. Physiotherapist 6. Psychologist 7. Private practice medical specialist 8. Secondary care 9. Somatic inpatient admissions 10. Somatic outpatient contacts 11. Somatic emergency admissions 12. Psychiatric inpatient admissions 13. Psychiatric emergency admissions | | | | NR | 1. Primary care 2. 1.10 (1.03–1.17) 3. 1.08 (1.01–1.14) 4. 1.13 (1.04–1.23) 5. 1.10 (0.81–1.49) 6. 1.37 (0.87–2.18) 7. 1.15 (0.99–1.33) 8. Secondary care 9. 0.98 (0.85–1.13) 10. 1.08 (1.01–1.16) 11. 1.13 (1.00–1.28) 12. 0.65 (0.31–1.36) 13. 0.84 (0.42–1.68) | Age, sex, highest attained educational level at age 40 years (or at time of diagnosis, whichever came first), and civil status at index date |
|  |  |  | The 5->1-year interval prior to diagnosis | 1. Primary care 2. Any general practitioner contact 3. General practitioner face-to-face contacts 4. General practitioner telephone/email contacts 5. Physiotherapist 6. Psychologist 7. Private practice medical specialist 8. Secondary care 9. Somatic inpatient admissions 10. Somatic outpatient contacts 11. Somatic emergency admissions 12. Psychiatric inpatient admissions 13. Psychiatric emergency admissions | | | |  | 1. Primary care 2. 1.20 (1.13–1.27) 3. 1.20 (1.13–1.28) 4. 1.19 (1.10–1.30) 5. 0.84 (0.61–1.15) 6. 2.69 (1.58–4.56) 7. 1.08 (0.95–1.23) 8. Secondary care 9. 1.11 (0.94–1.31) 10. 1.17 (1.09–1.26) 11. 1.34 (1.18–1.52) 12. 2.15 (1.09–4.24) 13. 1.74 (0.86–3.56) |  |
|  |  |  | The ≤1-year interval prior to diagnosis | 1. Primary care 2. Any general practitioner contact 3. General practitioner face-to-face contacts 4. General practitioner telephone/email contacts 5. Physiotherapist 6. Psychologist 7. Private practice medical specialist 8. Secondary care 9. Somatic inpatient admissions 10. Somatic outpatient contacts 11. Somatic emergency admissions 12. Psychiatric inpatient admissions 13. Psychiatric emergency admissions | | | |  | 1. Primary care 2. 1.73 (1.64–1.83) 3. 1.67 (1.58–1.76) 4. 1.80 (1.66–1.96) 5. 0.59 (0.41–0.85) 6. 4.63 (2.36–9.06) 7. 1.10 (0.93–1.31) 8. Secondary care 9. 1.66 (1.36–2.04) 10. 2.01 (1.85–2.19) 11. 1.89 (1.55–2.30) 12. 8.31 (3.35–20.60) 13. 8.69 (4.29–17.62) |  |
| Wieland DR, 2022 | OR | YOD | Without Thyroid Disorders (ref.)  With hypothyroidism  With hyperthyroidism  With acquired hypothyroidism  Others | | | | | NR | 1 (ref.)  2.43 (0.83-7.12)  0.67 (0.22-1.99)  NR  0.70 (0.38-1.31) | Sex, age, history of hypertension, diabetes, coronary artery disease, depression, hyperlipidemia, alcohol dependence syndrome, tinnitus, hearing loss, and radioactive iodine treatment |
| Kuang TT, 2022 | OR | YOD | Prior macular degeneration (+ vs. -) | | | | | 399/1,091 vs. 36,178/108,640 | 1.10 (0.98-1.24) | Age, sex, monthly income, geographic region, urbanization level, hyperlipidemia, diabetes, coronary heart disease, traumatic brain injury, tobacco use disorder, alcohol dependency, obesity, and stroke |
| Kennedy E, 2022 | OR | YOD | 1. Traumatic brain injury (+ vs. -) 2. Epilepsy (+ vs. -) 3. Memory loss (+ vs. -) 4. Other neurological conditions (+ vs. -) 5. Cardiac disease (+ vs. -) 6. Depression (+ vs. -) | | | | | NR | 1. 3.05 (2.42-3.83) 2. 4.8 (3.3-6.97) 3. NA 4. 2.0 (1.35-2.97) 5. 1.36 (1.1-1.67) 6. 1.35 (1.12-1.63) | Age, sex, race/ethnicity, employment, marital status, epilepsy, and other neurological conditions |
| Jaakkimainen L, 2022 | OR | YOD | 1. History of Acute Myocardial Infarction (+ vs. -) 2. Congestive Heart Failure (+ vs. -) 3. Hypertension (+ vs. -) 4. Diabetes (+ vs. -) 5. Asthma (+ vs. -) 6. Chronic Obstructive Pulmonary Disease (+ vs. -) 7. Parkinsonism (+ vs. -) 8. Epilepsy (+ vs. -) 9. Multiple Sclerosis (+ vs. -) 10. Schizophrenia (+ vs. -) 11. Any Mental Health (+ vs. -) 12. HIV (+ vs. -) | | | | | NR | 1. 1.58 (1.49-1.69) 2. 3.10 (2.95-3.24) 3. 1.42 (1.39-1.46 4. 1.92 (1.87-1.98) 5. 1.45 (1.40-1.50) 6. 2.09 (2.03-2.15) 7. 12.53 (11.84-13.27) 8. 11.35 (10.91-11.81) 9. 10.27 (9.55-11.03) 10. 16.71 (16.03-17.41) 11. 3.26 (3.18-3.34) 12. 4.20 (3.61-4.87) | Age, sex, rurality, and neighborhood income quintiles |
| Downing KF, 2022 | PR | YOD | Congenital heart defects (+ vs. -) | | | | | 35/12,250 vs. 4800/3,942,077 | 2.2 (1.6-3.0) | Age and sex |
| Cheng YF, 2021 | OR | YOD | Prior diagnosis of tinnitus (+ vs. -) | | | | | 281/190 vs. 1027/1,118 | 1.63 (1.32-2.01) | Age, sex, monthly income, geographic region, urbanization level, hyperlipidemia, diabetes, hypertension, coronary heart disease, hearing loss, obesity, and alcohol abuse |
| Carcaillon-Bentata L, 2021 | OR | YOD | 1. Sex (male vs. female) 2. Morbid obesity (+ vs. -) 3. Diabetes (+ vs. -) 4. Acute cerebrovascular disease (excluding transient attacks) (+ vs. -) 5. Sequelae of cerebrovascular disease or history of acute cerebrovascular disease (+ vs. -) 6. Acute ischemic heart disease (+ vs. -) 7. Chronic ischemic heart disease or history of acute ischemic heart disease (+ vs. -) 8. Cardiac arrhythmias and conduction disorders (+ vs. -) 9. Antihypertensive drug therapy (+ vs. -) 10. Parkinson disease (+ vs. -) 11. Epilepsy (+ vs. -) 12. Multiple sclerosis (+ vs. -) 13. Substance abuse disorders (drugs, alcohol, and cannabis) (+ vs. -) 14. Schizophrenia and psychotic disorders (+ vs. -) 15. Traumatic brain injury (+ vs. -) | | | | | NR | 1. 1.48 (1.39-1.57) 2. 1.75 (1.57-1.95) 3. 1.36 (1.24-1.49) 4. 16.27 (13.55-19.52) 5. 9.64 (8.75-10.63) 6. 2.29 (1.52-3.45) 7. 1.55 (1.36-1.76) 8. 3.00 (2.61-3.44) 9. 1.06 (0.99-1.13) 10. 26.21 (23.15-29.68) 11. 16.06 (14.43-17.89) 12. 8.99 (7.35-10.97) 13. 18.34 (16.79-20.04) 14. 7.12 (6.36-7.97) 15. 14.02 (12.35-15.92) | Age and sex |
| Filippini T, 2020 | OR | YOD | 1. GM diet   T1 (ref.)  T2  T3  Per 1-unit increase   1. DASH diet   T1 (ref.)  T2  T3  Per 1-unit increase   1. MIND diet   T1 (ref.)  T2  T3  Per 1-unit increase   1. Cereals and cereal products   T1 (ref.)  T2  T3  Per 10 g increase   1. Pasta and other grains   T1 (ref.)  T2  T3  Per 10 g increase   1. Rice   T1 (ref.)  T2  T3  Per 10 g increase  Per 1 g increase   1. Bread   T1 (ref.)  T2  T3  Per 10 g increase   1. Pizza, crackers, and other salty snacks   T1 (ref.)  T2  T3  Per 10 g increase   1. Meats and meat products   T1 (ref.)  T2  T3  Per 10 g increase   1. Red meat   T1 (ref.)  T2  T3  Per 10 g increase   1. White meat   T1 (ref.)  T2  T3  Per 10 g increase   1. Processed meat   T1 (ref.)  T2  T3  Per 10 g increase   1. Offal   T1 (ref.)  T2  T3  Per 10 g increase  Per 1 g increase   1. Milk and dairy products   T1 (ref.)  T2  T3  Per 10 g increase   1. Milk and yogurt   T1 (ref.)  T2  T3  Per 10 g increase   1. Milk   T1 (ref.)  T2  T3  Per 10 g increase   1. Yogurt   T1 (ref.)  T2  T3  Per 10 g increase   1. Cheese   T1 (ref.)  T2  T3  Per 10 g increase  Per 1 g increase   1. Fresh cheese   T1 (ref.)  T2  T3  Per 10 g increase  Per 1 g increase   1. Aged cheese   T1 (ref.)  T2  T3  Per 10 g increase  Per 1 g increase   1. Eggs   T1 (ref.)  T2  T3  Per 10 g increase  Per 1 g increase   1. Fish and seafood   T1 (ref.)  T2  T3  Per 10 g increase   1. Fish   T1 (ref.)  T2  T3  Per 10 g increase   1. Preserved and tinned fish   T1 (ref.)  T2  T3  Per 10 g increase  Per 1 g increase   1. Non-piscivorous fish   T1 (ref.)  T2  T3  Per 10 g increase  Per 1 g increase   1. Piscivorous fish   T1 (ref.)  T2  T3  Per 10 g increase  Per 1 g increase   1. Crustaceans and molluscs   T1 (ref.)  T2  T3  Per 10 g increase  Per 1 g increase   1. All vegetables   T1 (ref.)  T2  T3  Per 10 g increase   1. Leafy vegetables   T1 (ref.)  T2  T3  Per 10 g increase  Per 1 g increase   1. Tomatoes   T1 (ref.)  T2  T3  Per 10 g increase   1. Root vegetables   T1 (ref.)  T2  T3  Per 10 g increase  Per 1 g increase   1. Cabbage   T1 (ref.)  T2  T3  Per 10 g increase  Per 1 g increase   1. Other vegetables   T1 (ref.)  T2  T3  Per 10 g increase  Per 1 g increase   1. Mushrooms   T1 (ref.)  T2  T3  Per 10 g increase  Per 1 g increase   1. Legumes   T1 (ref.)  T2  T3  Per 10 g increase  Per 1 g increase   1. Potatoes   T1 (ref.)  T2  T3  Per 10 g increase  Per 1 g increase   1. Fresh fruit   T1 (ref.)  T2  T3  Per 10 g increase   1. Citrus fruit   T1 (ref.)  T2  T3  Per 10 g increase   1. All other fruit   T1 (ref.)  T2  T3  Per 10 g increase   1. Dry fruits, nuts and seeds   T1 (ref.)  T2  T3  Per 1 g increase   1. Dry fruits   T1 (ref.)  T2  T3  Per 1 g increase   1. Nuts and seeds   T1 (ref.)  T2  T3  Per 1 g increase   1. Sweets, chocolate, cakes, etc.   T1 (ref.)  T2  T3  Per 10 g increase   1. Sugar, non-chocolate confectionery   T1 (ref.)  T2  T3  Per 10 g increase   1. Chocolate, candy bars, etc.   T1 (ref.)  T2  T3  Per 10 g increase  Per 1 g increase   1. Ice-cream   T1 (ref.)  T2  T3  Per 10 g increase  Per 1 g increase   1. Cakes, pies and pastries   T1 (ref.)  T2  T3  Per 10 g increase   1. Biscuits, dry cakes   T1 (ref.)  T2  T3  Per 10 g increase  Per 1 g increase   1. Oils and fats   T1 (ref.)  T2  T3  Per 10 g increase  Per 1 g increase   1. Vegetable fats and non-olive oils   T1 (ref.)  T2  T3  Per 10 g increase  Per 1 g increase   1. Olive oil   T1 (ref.)  T2  T3  Per 10 g increase  Per 1 g increase   1. Butter and other animal fats   T1 (ref.)  T2  T3  Per 10 g increase  Per 1 g increase   1. Coffee and tea   T1 (ref.)  T2  T3  Per 10 g increase   1. Coffee   T1 (ref.)  T2  T3  Per 10 g increase   1. Tea   T1 (ref.)  T2  T3  Per 10 g increase   1. Wine   T1 (ref.)  T2  T3  Per 10 g increase   1. Red wine   T1 (ref.)  T2  T3  Per 10 g increase   1. White wine   T1 (ref.)  T2  T3  Per 10 g increase   1. Aperitif wines and beers   T1 (ref.)  T2  T3  Per 10 g increase   1. Spirits and liqueurs   T1 (ref.)  T2  T3  Per 1 g increase   1. Fruit juices   T1 (ref.)  T2  T3  Per 10 g increase   1. Soft drinks   T1 (ref.)  T2  T3  Per 10 g increase   1. Alcohol   T1 (ref.)  T2  T3  Per 1 g increase | | | | | 1. GM diet   24/19 (ref.)  18/18  12/17  NA   1. DASH diet   21/19 (ref.)  22/19  11/16  NA   1. MIND diet   30/16 (ref.)  15/22  9/16  NA   1. Cereals and cereal products   21/18 (ref.)  7/18  26/18  NA   1. Pasta and other grains   12/17 (ref.)  10/19  32/18  NA   1. Rice   23/25 (ref.)  11/15  20/14  NA  NA   1. Bread   21/18 (ref.)  15/18  18/18  NA   1. Pizza, crackers, and other salty snacks   29/18 (ref.)  11/18  14/18  NA   1. Meats and meat products   15/18 (ref.)  19/18  20/18  NA   1. Red meat   19/19 (ref.)  15/17  20/18  NA   1. White meat   15/20 (ref.)  13/17  26/17  NA   1. Processed meat   20/18 (ref.)  11/17  23/19  NA   1. Offal   42/42 (ref.)  8/7  4/5  NA  NA   1. Milk and dairy products   16/18 (ref.)  14/18  24/18  NA   1. Milk and yogurt   23/25 (ref.)  12/15  19/14  NA   1. Milk   26/28 (ref.)  11/14  17/12  NA   1. Yogurt   29/34  10/8  15/12  NA   1. Cheese   13/18  16/18  25/18  NA  NA   1. Fresh cheese   20/23  16/16  15/18  NA  NA   1. Aged cheese   18/20  13/17  23/17  NA  NA   1. Eggs   21/20  13/17  20/17  NA  NA   1. Fish and seafood   22/20  18/17  14/17  NA   1. Fish   26/21  13/17  15/16  NA   1. Preserved and tinned fish   19/22  19/18  16/14  NA  NA   1. Non-piscivorous fish   26/27  14/14  14/13  NA  NA   1. Piscivorous fish   34/29  14/13  6/12  NA  NA   1. Crustaceans and molluscs   28/26  9/14  17/14  NA  NA   1. All vegetables   29/18  7/18  18/18  NA   1. Leafy vegetables   26/18  16/18  12/18  NA  NA   1. Tomatoes   21/18  16/18  17/18  NA   1. Root vegetables   27/18  14/18  13/18  NA  NA   1. Cabbage   32/31  12/12  10/11  NA  NA   1. Other vegetables   25/18  18/18  11/18  NA  NA   1. Mushrooms   30/24  9/10  15/20  NA  NA   1. Legumes   17/18  16/18  21/18  NA  NA   1. Potatoes   22/18  15/18  17/18  NA  NA   1. Fresh fruit   17/18  16/18  21/18  NA   1. Citrus fruit   21/19  20/17  13/18  NA   1. All other fruit   17/18  12/18  25/18  NA   1. Dry fruits, nuts and seeds   31/19  14/18  9/17  NA   1. Dry fruits   29/20  18/22  7/12  NA   1. Nuts and seeds   32/20  13/15  9/19  NA   1. Sweets, chocolate, cakes, etc.   11/18  15/18  28/18  NA   1. Sugar, non-chocolate confectionery   25/25  13/13  16/16  NA   1. Chocolate, candy bars, etc.   37/33  8/10  9/11  NA  NA   1. Ice-cream   18/24  12/15  24/15  NA  NA   1. Cakes, pies and pastries   13/22  11/16  30/16  NA   1. Biscuits, dry cakes   24/28  9/13  21/13  NA  NA   1. Oils and fats   25/18  10/18  19/18  NA  NA   1. Vegetable fats and non-olive oils   31/28  13/12  10/14  NA  NA   1. Olive oil   19/18  15/18  20/18  NA  NA   1. Butter and other animal fats   16/22  24/16  14/16  NA  NA   1. Coffee and tea   27/19  11/17  16/18  NA   1. Coffee   22/19  12/14  20/21  NA   1. Tea   40/36  7/7  7/11  NA   1. Wine   33/33  1/7  20/14  NA   1. Red wine   38/37  3/9  13/8  NA   1. White wine   38/37  14/9  2/8  NA   1. Aperitif wines and beers   44/33  6/11  4/10  NA   1. Spirits and liqueurs   48/36  5/11  1/7  NA   1. Fruit juices   30/38  12/8  12/8  NA   1. Soft drinks   43/42  5/4  6/8  NA   1. Alcohol   22/22  14/16  16/16  NA | 1. GM diet   1.00 (ref.)  0.76 (0.30-1.96)  0.45 (0.16-1.26)  0.84 (0.65-1.09)   1. DASH diet   1.00 (ref.)  0.87 (0.35-2.15)  0.60 (0.21-1.72)  0.98 (0.90-1.06)   1. MIND diet   1.00 (ref.)  0.32 (0.12-0.83)  0.31 (0.11-0.90)  0.66 (0.47-0.91)   1. Cereals and cereal products   1.00 (ref.)  0.30 (0.09-0.96)  0.80 (0.27-2.38)  1.01 (0.96-1.06)   1. Pasta and other grains   1.00 (ref.)  0.74 (0.24-2.25)  2.57 (0.87-7.55)  1.15 (1.00-1.32)   1. Rice   1.00 (ref.)  0.78 (0.28-2.15)  1.45 (0.56-3.77)  1.86 (0.94-3.97)  1.06 (0.99-1.14)   1. Bread   1.00 (ref.)  0.80 (0.30-2.16)  0.63 (0.22-1.82)  1.02 (0.96-1.08)   1. Pizza, crackers, and other salty snacks   1.00 (ref.)  0.30 (0.11-0.84)  0.33 (0.12-0.93)  0.86 (0.75-0.99)   1. Meats and meat products   1.00 (ref.)  1.37 (0.51-3.68)  1.18 (0.41-3.45)  1.02 (0.94-1.11)   1. Red meat   1.00 (ref.)  0.80 (0.29-2.23)  1.04 (0.38-2.86)  1.01 (0.91-1.12)   1. White meat   1.00 (ref.)  0.80 (0.28-2.29)  1.85 (0.71-4.84)  1.12 (0.92-1.36)   1. Processed meat   1.00 (ref.)  0.51 (0.18-1.47)  0.94 (0.35-2.54)  0.98 (0.78-1.24)   1. Offal   1.00 (ref.)  1.28 (0.40-4.07)  0.57 (0.13-2.43)  0.58 (0.24-1.42)  0.95 (0.87-1.04)   1. Milk and dairy products   1.00 (ref.)  1.05 (0.37-3.00)  1.25 (0.48-3.25)  1.02 (1.00-1.04)   1. Milk and yogurt   1.00 (ref.)  0.85 (0.32-2.28)  1.25 (0.50-3.16)  1.02 (1.00-1.04)   1. Milk   1.00 (ref.)  0.73 (0.26-1.06)  1.25 (0.48-3.26)  1.02 (0.99-1.04)   1. Yogurt   1.00 (ref.)  1.40 (0.46-4.21)  1.65 (0.64-4.28)  1.03 (0.98-1.08)   1. Cheese   1.00 (ref.)  1.03 (0.36-2.90)  1.88 (0.60-5.91)  1.09 (0.91-1.31)  1.01 (0.99-1.03)   1. Fresh cheese   1.00 (ref.)  1.39 (0.53-3.65)  1.47 (0.53-4.12)  1.06 (0.77-1.47)  1.01 (0.97-1.04)   1. Aged cheese   1.00 (ref.)  0.87 (0.32-2.38)  1.37 (0.49-3.79)  1.11 (0.85-1.45)  1.01 (0.98-1.04)   1. Eggs   1.00 (ref.)  0.64 (0.23-1.77)  0.86 (0.31-2.34)  1.02 (0.72-1.43)  1.00 (0.97-1.04)   1. Fish and seafood   1.00 (ref.)  0.98 (0.39-2.48)  0.70 (0.26-1.86)  0.97 (0.82-1.15)   1. Fish   1.00 (ref.)  0.58 (0.22-1.51)  0.69 (0.26-1.85)  0.98 (0.80-1.19)   1. Preserved and tinned fish   1.00 (ref.)  0.85 (0.32-2.29)  1.22 (0.44-3.36)  1.29 (0.88-1.88)  1.03 (0.99-1.07)   1. Non-piscivorous fish   1.00 (ref.)  0.86 (0.33-2.26)  0.81 (0.29-2.27)  0.91 (0.64-1.28)  0.99 (0.96-1.03)   1. Piscivorous fish   1.00 (ref.)  0.91 (0.35-2.32)  0.48 (0.16-1.49)  0.72 (0.46-1.14)  0.97 (0.92-1.01)   1. Crustaceans and molluscs   1.00 (ref.)  0.59 (0.21-1.65)  1.22 (0.48-3.10)  0.92 (0.55-1.53)  0.99 (0.94-1.04)   1. All vegetables   1.00 (ref.)  0.23 (0.08-0.70)  0.53 (0.21-1.38)  0.95 (0.90-1.00)   1. Leafy vegetables   1.00 (ref.)  0.56 (0.21-1.53)  0.43 (0.15-1.19)  0.82 (0.66-1.02)  0.98 (0.96-1.00)   1. Tomatoes   1.00 (ref.)  0.68 (0.26-1.78)  0.74 (0.27-1.97)  0.95 (0.87-1.04)   1. Root vegetables   1.00 (ref.)  0.57 (0.21-1.50)  0.47 (0.18-1.27)  0.89 (0.77-1.02)  0.99 (0.97-1.00)   1. Cabbage   1.00 (ref.)  0.90 (0.33-2.41)  0.76 (0.26-2.18)  0.95 (0.58-1.57)  1.00 (0.95-1.05)   1. Other vegetables   1.00 (ref.)  0.81 (0.31-2.11)  0.38 (0.14-1.06)  0.83 (0.68-1.02)  0.98 (0.96-1.00)   1. Mushrooms   1.00 (ref.)  0.63 (0.21-1.90)  0.55 (0.22-1.40)  0.51 (0.11-2.36)  0.93 (0.80-1.09)   1. Legumes   1.00 (ref.)  0.93 (0.35-2.48)  1.27 (0.48-3.31)  0.93 (0.76-1.15)  0.99 (0.97-1.01)   1. Potatoes   1.00 (ref.)  0.54 (0.20-1.45)  0.70 (0.27-1.83)  0.88 (0.69-1.12)  0.99 (0.96-1.01)   1. Fresh fruit   1.00 (ref.)  0.77 (0.28-2.12)  0.97 (0.36-2.58)  1.00 (0.97-1.03)   1. Citrus fruit   1.00 (ref.)  0.94 (0.37-2.43)  0.53 (0.19-1.46)  0.91 (0.83-1.00)   1. All other fruit   1.00 (ref.)  0.55 (0.19-1.57)  1.23 (0.45-3.35)  1.01 (0.98-1.05)   1. Dry fruits, nuts and seeds   1.00 (ref.)  0.38 (0.15-1.00)  0.24 (0.08-0.72)  0.89 (0.80-0.98)   1. Dry fruits   1.00 (ref.)  0.52 (0.21-1.28)  0.23 (0.06-0.80)  0.74 (0.51-1.07)   1. Nuts and seeds   1.00 (ref.)  0.43 (0.16-1.17)  0.23 (0.08-0.68)  0.89 (0.79-1.00)   1. Sweets, chocolate, cakes, etc.   1.00 (ref.)  1.47 (0.50-4.38)  2.61 (0.82-8.34)  1.00 (0.96-1.06)   1. Sugar, non-chocolate confectionery   1.00 (ref.)  0.85 (0.31-2.30)  0.79 (0.30-2.07)  0.98 (0.88-1.09)   1. Chocolate, candy bars, etc.   1.00 (ref.)  0.77 (0.26-2.28)  0.83 (0.28-2.49)  0.70 (0.45-1.08)  0.96 (0.92-1.01)   1. Ice-cream   1.00 (ref.)  1.37 (0.48-3.87)  2.69 (1.00-7.22)  1.15 (0.97-1.37)  1.01 (1.00-1.03)   1. Cakes, pies and pastries   1.00 (ref.)  1.26 (0.43-3.66)  3.16 (1.15-8.69)  0.99 (0.93-1.05)   1. Biscuits, dry cakes   1.00 (ref.)  1.04 (0.36-3.04)  1.72 (0.63-4.66)  1.19 (0.94-1.51)  1.02 (0.99-1.04)   1. Oils and fats   1.00 (ref.)  0.37 (0.13-1.03)  0.58 (0.21-1.61)  0.66 (0.46-0.97)  0.96 (0.92-1.00)   1. Vegetable fats and non-olive oils   1.00 (ref.)  0.99 (0.37-2.66)  0.77 (0.27-2.21)  0.39 (0.11-1.38)  0.91 (0.80-1.03)   1. Olive oil   1.00 (ref.)  0.72 (0.26-1.94)  0.79 (0.29-2.18)  0.73 (0.50-1.06)  0.97 (0.93-1.01)   1. Butter and other animal fats   1.00 (ref.)  2.22 (0.85-5.80)  1.23 (0.42-3.56)  0.78 (0.26-2.36)  0.98 (0.87-1.09)   1. Coffee and tea   1.00 (ref.)  0.46 (0.17-1.24)  0.56 (0.21-1.46)  0.99 (0.96-1.02)   1. Coffee   1.00 (ref.)  0.77 (0.27-2.19)  0.71 (0.29-1.78)  0.99 (0.92-1.06)   1. Tea   1.00 (ref.)  0.78 (0.24-2.54)  0.61 (0.19-1.93)  0.99 (0.96-1.02)   1. Wine   1.00 (ref.)  0.17 (0.02-1.47)  1.31 (0.54-3.16)  0.99 (0.97-1.02)   1. Red wine   1.00 (ref.)  0.38 (0.09-1.56)  1.70 (0.58-5.00)  1.02 (0.97-1.08)   1. White wine   1.00 (ref.)  1.50 (0.55-4.07)  0.20 (0.04-1.10)  0.99 (0.95-1.02)   1. Aperitif wines and beers   1.00 (ref.)  0.40 (0.12-1.30)  0.25 (0.06-1.01)  0.95 (0.90-1.01)   1. Spirits and liqueurs   1.00 (ref.)  0.28 (0.08-0.96)  0.11 (0.01-1.15)  0.42 (0.16-1.09)   1. Fruit juices   1.00 (ref.)  2.50 (0.84-7.43)  1.94 (0.66-5.71)  1.02 (0.99-1.04)   1. Soft drinks   1.00 (ref.)  1.67 (0.38-7.40)  0.62 (0.18-2.18)  0.98 (0.95-1.01)   1. Alcohol   1.00 (ref.)  0.81 (0.30-2.21)  1.04 (0.37-2.93)  0.99 (0.97-1.02) | Sex, age, educational attainment, and total energy intake |
| Adani G, 2020 | OR | YOD | 1. Sex (women vs. men) 2. Age (continuous) 3. Educational attainment (high school or more vs. middle school or less) 4. Marital status (single/separated/widowed vs. married/unmarried partner) 5. Occupational exposure (+ vs. -) 6. toxic agents 7. Lead 8. Mercury 9. Selenium 10. Cadmium 11. Arsenic 12. Aluminum 13. Overall pesticides 14. Insecticides 15. Herbicides 16. Fungicides 17. Overall solvents and dyes 18. Oil paints 19. Thinner 20. Paint remover 21. Paints 22. Adhesives 23. Print inks and dyes 24. Lubricating oils 25. Refrigerants, antifreezes, cooling liquids 26. Degreasing agents 27. Solvents (e.g., toluene, xylene) 28. Dry clean products 29. Anesthetic gas 30. Electric and electronic equipment 31. Electromagnetic fields 32. Fertilizers 33. Pesticides 34. Disinfectants 35. Detergents 36. Solvents 37. Oils 38. Diesel/gasoline 39. Work accident with toxicant exposure 40. Nausea/indisposition due to occupational exposure 41. Trauma that need medical evaluation 42. Head trauma 43. Trunk trauma 44. Upper arm trauma 45. Lower arm trauma 46. Electric shock/trauma 47. Hunting 48. Fishing 49. Painting 50. Model-making 51. Gardening 52. Using pesticides during gardening 53. Using herbicides during gardening 54. Using fungicides during gardening 55. Developing pictures in darkroom 56. Playing sport 57. Playing competitive sport 58. Football 59. Volleyball 60. Cycling 61. Swimming 62. Athletics 63. Running/trekking 64. Use of dietary supplements containing selenium in the past 20 years 65. Excluding specific neuroprotective supplements after onset of disease 66. Use of selenized potatoes 67. Ever smoking 68. Current smoking 69. Passive smoking exposure | | | | | 1. 33/64 vs. 25/48 2. NA 3. 22/54 vs. 36/58 4. 10/16 vs. 48/96 5. Occupational exposure 6. 6/15 vs. 47/88 7. 5/11 vs. 53/101 8. 1/3 vs. 57/109 9. 1/2 vs. 57/100 10. 1/1vs. 57/101 11. 0/0 vs. 58/112 12. 4/6 vs. 54/106 13. 9/14 vs. 49/98 14. 8/13 vs. 50/99 15. 7/11 vs. 51/101 16. 2/4 vs. 56/108 17. 12/20 vs. 46/92 18. 5/8 vs. 12/63 19. 8/13 vs. 50/99 20. 4/6 vs. 54/106 21. 6/10 vs. 52/102 22. 2/3 vs. 56/109 23. 4/7 vs. 54/105 24. 3/7 vs. 55/105 25. 1/2 vs. 57/110 26. 3/4 vs. 55/108 27. 2/4 vs. 56/108 28. 3/3 vs. 55/109 29. 1/1 vs. 57/111 30. 4/13 vs. 54/99 31. 1/4 vs. 57/108 32. 4/7 vs. 54/105 33. 7/10 vs. 51/102 34. 2/3 vs. 56/109 35. 2/3 vs. 56/109 36. 2/2 vs. 56/110 37. 4/7 vs. 54/105 38. 4/8 vs. 54/104 39. 0/1 vs. 58/111 40. 1/2 vs. 57/110 41. 23/45 vs. 32/63 42. 9/15 vs. 46/93 43. 0/2 vs. 55/106 44. 10/18 vs. 45/90 45. 13/26 vs. 42/82 46. 1/3 vs. 57/109 47. 2/4 vs. 56/108 48. 6/13 vs. 52/99 49. 7/13 vs. 47/93 50. 3/6 vs. 55/106 51. 22/48 vs. 36/64 52. 7/15 vs. 51/97 53. 4/8 vs. 54/104 54. 6/10 vs. 52/102 55. 1/2 vs. 57/110 56. 15/43 vs. 43/69 57. 4/12 vs. 54/100 58. 7/11 vs. 51/101 59. 2/8 vs. 56/104 60. 5/7 vs. 53/105 61. 2/14 vs. 56/98 62. 2/8 vs. 56/104 63. 1/5 vs. 57/107 64. 14/22 vs. 44/90 65. 11/19 vs. 47/93 66. 7/18 vs. 51/94 67. 35/65 vs. 23/47 68. 10/20 vs. 48/92 69. 15/27 vs. 43/85 | 1. 0.98 (0.45-2.12) 2. 1.03 (0.97-1.08) 3. 0.43 (0.20-0.94) 4. 1.78 (0.56-5.59) 5. Occupational exposure 6. 0.85 (0.22-3.29) 7. 0.83 (0.22-3.15) 8. 0.31 (0.02–4.01) 9. 1.56 (0.09–27.77) 10. NA 11. NA 12. 2.59 (0.43–15.66) 13. 2.28 (0.67–7.77) 14. 1.98 (0.56–6.96) 15. 2.36 (0.61–9.14) 16. 1.68 (0.21–13.31) 17. 1.74 (0.61–5.02) 18. 2.51 (0.62–10.15) 19. 2.07 (0.58–7.46) 20. 1.77 (0.30–10.56) 21. 1.34 (0.34–5.37) 22. 2.68 (0.22–32.63) 23. 1.74 (0.35–8.72) 24. 0.80 (0.16–3.94) 25. 1.73 (0.10–29.84) 26. 2.38 (0.23–24.40) 27. 1.04 (0.13–8.22) 28. NA 29. NA 30. 0.54 (0.14–2.00) 31. 0.37 (0.04–3.81) 32. 1.96 (0.39–9.74) 33. 3.11 (0.72–13.32) 34. 2.65 (0.2–34.84) 35. 2.26 (0.17–30.78) 36. NA 37. 1.71 (0.34–8.48) 38. 1.14 (0.26–5.06) 39. NA 40. 0.98 (0.06–16.23) 41. 0.93 (0.42–2.07) 42. 1.54 (0.47–4.99) 43. NA 44. 1.38 (0.47–4.03) 45. 0.85 (0.34–2.13) 46. 0.18 (0.01–2.22) 47. 1.03 (0.13–8.25) 48. 0.65 (0.18–2.35) 49. 1.08 (0.32–3.66) 50. 1.11 (0.19–6.42) 51. 0.71 (0.32–1.57) 52. 0.84 (0.27–2.55) 53. 1.31 (0.28–6.08) 54. 1.56 (0.40–6.10) 55. 1.00 (0.06–17.79) 56. 0.36 (0.15–0.89) 57. 0.45 (0.11–1.74) 58. 2.23 (0.54–9.26) 59. 0.36 (0.07–1.92) 60. 2.28 (0.39–13.43) 61. 0.17 (0.03–0.84) 62. 0.29 (0.05–1.56) 63. 0.34 (0.03–3.25) 64. 2.50 (0.89–7.02) 65. 1.73 (0.60–4.99) 66. 0.55 (0.19–1.62) 67. 1.28 (0.58–2.86) 68. 1.01 (0.37–2.79) 69. 1.17 (0.47–2.88) | Sex, age and educational attainment |
| Keret O, 2018 | OR | AD (YO) | 1. Age 2. Sex (female vs. male) 3. Yemenite origin 4. Years of education 5. Diabetes 6. Hypertension 7. Smoking | | | | | NR | 1. 1.19 (1.14-1.24) 2. 1.44 (0.96-2.16) 3. 2.54 (1.44-4.86) 4. 0.83 (0.78-0.88) 5. 2.93 (1.73-4.96) 6. 0.94 (0.61-1.45) 7. 0.58 (0.35-0.96) | Age, sex, Yemenite origin, years of education, diabetes, hypertension, and smoking status |
| Cations M, 2018 | OR | YOD | 1. Years of education 2. Main lifetime occupation code 3. Lifetime cognitive leisure activity (low vs. high) 4. Stroke or transient ischemic attack (+ vs. -) 5. Distal hypertension (+ vs. -) 6. Distal hypercholesterolemia (+ vs. -) 7. Smoking (current vs. never) 8. Proximal depression (+ vs. -) 9. Alcohol use (heavy vs. mild to moderate) | | | | | 1. NA 2. NA 3. 59/74 vs. 37/105 4. 21/12 vs. 75/167 5. 23/23 vs. 51/108 6. 11/8 vs. 68/134 7. 21/21 vs. 33/83 8. 30/23 vs. 55/125 9. 11/8 vs. 35/53 | 1. 0.87 (0.79-0.95) 2. 0.99 (0.85-1.15) 3. 3.62 (1.47-8.95) 4. 13.18 (2.62-66.4) 5. 2.13 (0.98-3.72) 6. 2.53 (0.70-9.13) 7. 2.39 (0.86-6.62) 8. 3.36 (1.46-7.73) 9. 3.36 (0.88-12.9) | Family history of dementia, years of education, main occupation code, cognitive leisure activity, lifetime stroke or transient ischemic attack, distal hypertension and hypercholesterolemia, current smoking, proximal depression, and very heavy alcohol use |
| Kadohara K, 2017 | OR | AD (YO) | 1. Diabetes mellitus (+ vs. -) 2. Antidepressants use (+ vs. -) 3. Antipsychotics (+ vs. -) 4. Antithrombotics (+ vs. -) 5. Antihypertensives (+ vs. -) 6. Antihyperlipidemics (+ vs. -) 7. Polypharmacy (≥5 non-AD drugs vs. 0-4) | | | | | 1. 65/306 vs. 175/1,309 2. 85/286 vs. 43/1,441 3. 155/216 vs. 182/1,302 4. 67/304 vs. 115/1,369 5. 113/258 vs. 391/1,093 6. 77/294 vs. 270/1,214 7. 128/243 vs. 234/1,250 | 1. 1.31 (0.90-1.92) 2. 4.27 (2.68-6.81) 3. 3.19 (2.26-4.50) 4. 2.28 (1.51-3.45) 5. 0.84 (0.61-1.15) 6. 0.86 (0.61-1.22) 7. 1.17 (0.82-1.68) | Diabetes mellitus, prescription of antidepressants, antipsychotics, antithrombotics, antihypertensive, antihyperlipidemic agents, and polypharmacy |
| Chen Y, 2017 | OR | AD (YO) | 1. Educational level (high vs. low) 2. Alcohol (+ vs. -) 3. Smoking   Never (ref.)  Former  Current   1. BMI (kg/m^2^) 2. Systolic blood pressure (mmHg) 3. Blood pressure-lowering medication (+ vs. -) 4. Hypercholesterolemia (+ vs. -) 5. Hypertriglyceridemia (+ vs. -) 6. Lipid-lowering medication (+ vs. -) 7. Diabetes mellitus (+ vs. -) | | | | | NR | 1. 0.82 (0.75-0.89) 2. 0.08 (0.02-0.28) 3. Smoking   1.00 (ref.)  1.25 (0.62-2.52)  0.42 (0.15-1.17)   1. 0.90 (0.85-0.95) 2. 0.98 (0.96-0.99) 3. 1.93 (1.06-3.51) 4. 2.03 (0.96-4.32) 5. 0.33 (0.11-0.98) 6. 0.72 (0.31-1.68) 7. 2.35 (0.57-9.66) | Age, gender, educational level, smoking status, APOE ɛ4 status, regular alcohol consumption, BMI, blood pressure, blood pressure-lowering medication, hypercholesterolemia, hypertriglyceridemia, lipid-lowering medication, and diabetes mellitus |
| Mendez MF, 2015 | OR | AD (YO) | Traumatic brain injury (+ vs. -) | | | | | 193/161 vs. 1,234/1,279 | 1.28 (1.00-1.57) | Gender, age at initial visit, years of education, and race |
| Heath CA, 2015 | OR | YOD | 1. Stroke or transient ischaemic attack (+ vs. -) 2. Chronic kidney disease (+ vs. -) 3. Ischaemic heart disease (+ vs. -) 4. Diabetes (+ vs. -) 5. Hypertension (+ vs. -) 6. Peripheral vascular disease (+ vs. -) 7. Number of vascular comorbidities   None (ref.)  One  Two  Three  Four or more   1. Parkinson’s disease (+ vs. -) 2. Learning disability (+ vs. -) 3. Multiple sclerosis (+ vs. -) | | | | | 1. 67/8,338 vs. 994/606,846 2. 40/4,810 vs. 1,021/610,374 3. 99/21,414 vs. 962/593,770 4. 129/30,491 vs. 932/584,693 5. 287/92,255 vs. 774/522,929 6. 38/7,018 vs. 1,023/608,166 7. Number of vascular comorbidities   630/ 488,251 (ref.)  257/ 96,994  131/ 24,262  33/ 5,107  10/ 1,001   1. 9/389 vs. 1,952/ 614,795 2. 27/ 2,679 vs. 1,034/ 612,705 3. 9/ 2,525 vs. 1,052/ 612,659 | 1. 3.1 (2.4-4.0) 2. 2.9 (2.1-4.0) 3. 1.9 (1.5-2.4) 4. 2.0 (1.7-2.5) 5. 1.4 (1.2-1.6) 6. 2.2 (1.6-3.1) 7. Number of vascular comorbidities   1.0 (ref.)  1.5 (1.3-1.7)  2.8 (2.3-3.4)  3.1 (2.2-4.5)  4.6 (2.5-8.8)   1. 8.7 (4.4-16.9) 2. 7.1 (4.8-10.4) 3. 1.8 (0.9-3.4) | 1), 2), 3), 4), 5), 6) and 7) age, gender, neurodegenerative disorder, learning disability, and socioeconomic status  8), 9), and 10) age, gender, and socioeconomic status |
| Wu KY, 2013 | OR | YOD | Bipolar disorder (+ vs. -) | | | | | 23/25 vs. 1,190/7,253 | 3.77 (1.78-8.01) | Cerebrovascular disease, diabetes mellitus, hypertension, head injury, chronic pulmonary disease, alcohol-related disorder, substance-related disorder, outpatient visit, and inpatient visit |
| Draper B, 2011 | OR | YOD | 1. Circulatory (+ vs. -) 2. Stroke (+ vs. -) 3. Subdural (+ vs. -) 4. Other mental and behavioral (+ vs. -) 5. Alcohol (+ vs. -) 6. Respiratory (+ vs. -) 7. Lower respiratory tract infection (+ vs. -) 8. Ill-defined conditions (+ vs. -) 9. Syncope/collapse (+ vs. -) 10. Fractures (+ vs. -) 11. Femur (+ vs. -) 12. Other injury/poisoning (+ vs. -) 13. Head injuries/fractures (+ vs. -) 14. Digestive (+ vs. -) 15. Constipation (+ vs. -) 16. Genitourinary (+ vs. -) 17. Urinary tract infection (+ vs. -) 18. Neoplasms (+ vs. -) 19. Endocrine, nutritional, metabolic & immunity (+ vs. -) 20. Other nervous disorders (+ vs. -) 21. Transient ischemic attack (+ vs. -) 22. Epilepsy (+ vs. -) 23. Musculoskeletal (+ vs. -) 24. Infectious and parasitic (+ vs. -) 25. Septicemia (+ vs. -) 26. Have no procedure (+ vs. -) 27. Allied health (+ vs. -) 28. Imaging services (+ vs. -) 29. Computed Tomography head scans (+ vs. -) 30. Non-invasive, cognitive and other interventions (+ vs. -) 31. Procedures on musculoskeletal system (+ vs. -) 32. Procedures on pelvis/hip (+ vs. -) 33. Procedures on digestive system (+ vs. -) 34. Procedures on cardiovascular system (+ vs. -) 35. Procedures on coronary arteries (+ vs. -) 36. Procedures on urinary system (+ vs. -) 37. Procedures on urinary catheterization (+ vs. -) | | | | | NR | 1. 0.52 (0.44-0.62) 2. 1.26 (0.83-1.91) 3. 3.09 (0.97-9.87) 4. 5.39 (4.76-6.31) 5. 7.52 (6.29-8.99) 6. 0.91 (0.71-1.10) 7. 1.54 (1.20-1.98) 8. 0.91 (0.77-1.07) 9. 1.31 (0.83-2.07) 10. 1.08 (0.84-1.40) 11. 4.03 (2.61-6.21) 12. 0.97 (0.80-1.18) 13. 2.97 (2.09-4.23) 14. 0.77 (0.65-0.90) 15. 2.71 (1.55-4.73) 16. 0.58 (0.44-0.75) 17. 3.23 (2.29-4.55) 18. 0.28 (0.21-0.37) 19. 1.79 (1.43-2.24) 20. 3.86 (3.26-4.57) 21. 1.92 (1.22-3.04) 22. 10.17 (7.93-13.05) 23. 0.48 (0.37-0.63) 24. 0.97 (0.65-1.45) 25. 1.41 (0.81-2.45) 26. 0.82 (0.74-0.91) 27. 2.69 (2.39-3.04) 28. 2.04 (1.81-2.29) 29. 4.81 (4.23-5.48) 30. 1.23 (1.02-1.47) 31. 0.42 (0.33-0.55) 32. 0.83 (0.52-1.33) 33. 0.47 (0.38-0.57) 34. 0.23 (0.16-0.32) 35. 0.18 (0.11-0.29) 36. 0.67 (0.48-0.94) 37. 2.28 (1.07-4.87) | Age and sex |
| Slooter AJ, 1999 | OR | AD (YO) | Estrogen use (+ vs. -) | | | | | 6/18 vs. 98/86 | 0.34 (0.12-0.94) | Age and education level |
| van Duijn CM, 1995 | OR | AD (YO) | All subjects | | | History of cigarette smoking (APOE4 carriers):  No history of cigarette smoking (ref.)  History of cigarette smoking  History of cigarette smoking (APOE4 non-carriers):  No history of cigarette smoking (ref.)  History of cigarette smoking | | APOE4 carriers:  47/12 (ref.)  45/31  APOE4 non-carriers:  42/52 (ref.)  41/64 | APOE4 carriers:  1.00 (ref.)  0.25 (0.07-0.87)  APOE4 non-carriers:  1.00 (ref.)  0.70 (0 30-1.66) | Sex, age, place of residence, and family history of dementia |
|  |  |  | Subjects with family history of dementia | | | History of cigarette smoking (APOE4 carriers):  No history of cigarette smoking (ref.)  History of cigarette smoking  History of cigarette smoking (APOE4 non-carriers):  No history of cigarette smoking (ref.)  History of cigarette smoking | | APOE4 carriers:  35/1 (ref.)  30/13  APOE4 non-carriers:  22/14 (ref.)  20/16 | APOE4 carriers:  1.00 (ref.)  0.10 (0.01-0.87)  APOE4 non-carriers:  1.00 (ref.)  0.62 (0.19-2.07) | Sex, age, and place of residence |
|  |  |  | Subjects without family history of dementia | | | History of cigarette smoking (APOE4 carriers):  No history of cigarette smoking (ref.)  History of cigarette smoking  History of cigarette smoking (APOE4 non-carriers):  No history of cigarette smoking (ref.)  History of cigarette smoking | | APOE4 carriers:  12/11 (ref.)  15/18  APOE4 non-carriers:  20/38 (ref.)  21/48 | APOE4 carriers:  1.00 (ref.)  0.47 (0.10-2.28)  APOE4 non-carriers:  1.00 (ref.)  0.77 (0.17-3.42) | Sex, age, and place of residence |
| van Duijn CM, 1992 | OR | AD (YO) | 1. Head trauma with loss of consciousness (+ vs. -) 2. Head trauma with loss of consciousness   No head trauma (ref.)  Head trauma ≤ 10 years before  Head trauma > 10 years before   1. Family history of dementia in first-degree relatives (+ vs. -) 2. Educational attainment (< 7 years vs. ≥ 7 years) | | | | | 1. 22/17 vs. 176/181 2. Head trauma with loss of consciousness   176/181 (ref.)  8/1  12/15   1. 96/37 vs. 102/161 2. 112/88 vs. 86/110 | 1. 1.6 (0.8-3.4) 2. Head trauma with loss of consciousness   1.00 (ref.)  10.0 (1.0-96.8)  0.9 (0.4-2.2)   1. 4.9 (2.8-8.4) 2. 1.7 (1.1-2.7) | 1) and 2) dementia in first-degree relatives and education  3) education  4) dementia in first-degree relatives |
| van Duijn CM, 1991 | OR | AD (YO) | 1. Family history of dementia in first-degree relatives (+ vs. -) 2. Smoking status (Smoked vs. never smoked) 3. Number of cigarettes smoked daily   0  1-10  11-20  ≥21 | | | | | 1. 96/37 vs. 102/161 2. 89/102 vs. 104/93 3. Number of cigarettes smoked daily   104/93  47/46  27/30  15/26 | 1. 4.32 (2.71-6.82) 2. 0.70 (0.43-1.15) 3. Number of cigarettes smoked daily   1.00 (ref.)  0.79 (0.46-1.34)  0.59 (0.29-1.21)  0.39 (0.17-0.90) | 1) Age, sex, area of residence, and number of first-degree relatives  2) and 3) Age, sex, residence, and intake of alcohol |

Abbreviation: γ-GT, gamma-glutamyltransferase; AD, Alzheimer's disease; ADRD, Alzheimer's disease and related dementias; APOE, apolipoprotein E; ASD, autism spectrum disorder; BMI, body mass index; DASH, dietary approaches to stop hypertension; eGFR, estimated glomerular filtration rate; FTD, frontotemporal dementia; GM, Greek Mediterranean; GP, general population; HBA1C, hemoglobin A1c; HDL-C, high-density lipoprotein cholesterol; HIV, human immunodeficiency virus; HR, hazard ratio; ID, intellectual disability; IRR, incidence rate ratio; LDL-C, low-density lipoprotein cholesterol; MCI, mild cognitive impairment; MIND, Mediterranean-DASH diet intervention for neurodegenerative delay; NA, not applicable; NAFLD, non-alcoholic fatty liver disease; NR, not reported; OR, odds ratio; PPE, personal protective equipment; PR, prevalence ratio; PRS, polygenic risk score; PSM, propensity score matching; PY, person-year; RR, relative risk; SD, standard deviation; VaD, vascular dementia; WTC, World Trade Center; YO, young-onset; YOD, young-onset dementia.

# Supplementary Table 6. Diagnostic codes and/or prescription records for case ascertainment

| **Study** | **Databases for identifying dementia diagnoses and/or prescriptions** | **Detailed information** |
| --- | --- | --- |
| Park D, 2025 | KNHIS database | **Dementia:**  F00, F01, F02.0, F02.1, F02.2, F02.3, F02.8, F03, F11.78, G10, G20, G30, G31.0, G31.82, G35 |
|  |  | Prescription for dementia-related medications (donepezil, galantamine, rivastigmine, and memantine) |
| Yang YS, 2025 | KNHIS database | **All-cause dementia:**  F00, F01, F02, F03, G30, and G31 (ICD-10)  **Alzheimer’s disease:**  F00 and/or G30 (ICD-10)  **Vascular dementia:**  F01 (ICD-10) |
|  |  | At least two prescriptions for anti-dementia medications (rivastigmine, galantamine, memantine, or donepezil) |
| Yuan S, 2024 | UK Biobank | **Dementia:**  3310, 3311, 290, 2900, 2901, 2902, 2903, 2904, 2908, 2909, 3334, 294, 2940, 2941, 2948, 2949, 293, 2930, 2931, 2938, 2939, 2921, 2922 (ICD-9)  F01, F010, F011, F012, F013, F018, F019, F02, F020, F021, F022, F023, F024, F028, F03, F051, F106, G30, G300, G301, G308, G309, G31, G310, G311, G318, I673, A810, F00, F000, F001, F002, F009 (ICD-10)  A411., A4110, F11x7, Eu021, F21y2, G678., F1100, Eu000, F1101, Eu001, Fyu30, Eu002, F110., Eu00., Eu00z, F11., F111., Eu020, F112., F11x0, F1440, F10y., F10y0, F10y1, F10y2, F10yz, F118., F11y., F11y2, F11yz, Fyu31, Eu025, F116., F10., F10z., F11xz, F11z., Eu0., Eu01., Eu010, Eu011, Eu012, Eu013, Eu01y, Eu01z, Eu02., Eu02y, Eu02z, Eu041, Eu106 (Read v2)  F110., F110., X002x, X002x, XaIKB, XaIKB, F110., F110., X0030, X0030, XaIKC, XaIKC, Eu002, Eu002, F110., F110., Eu00., Eu00., Eu00z, Eu00z, F110., F110., X00Qz, E0040, E0041, E0042, E0043, X00Qz, XE1Xs, Xa0lH, X003R, Xa0lH, XaIRJ, X003T, X003V, Eu01y, E0040, E0041, E0042, E0043, Eu01z, XE1Xs, Eu020, Eu020, F111., F111., X0034, X0034, Xa0s2, Xa0s2, Eu021, Eu021, XaA1S, XaA1S, XabVp, XabVp, Eu022, Eu022, Eu023, Eu023, X003l, X003l, XaOfZ, XaOfZ, X003P, X003P, Eu02., Eu02y, X003A, X003A, X003Y, X003Y, X00Dx, X00Dx, X00R2, X00R2, Xa0s2, Xa0s2, XaE74, XaE74, XaKyY, XaKyY, Eu02., X002w, X00Qz, E...., E000., E001., E0010, E0012, E0013, E002., E0020, E0021, Eu02z, X002w, X00Qz, X00R0, X00R2, X00SO, XE1Xr, XE1Z6, XM09N, XM09O, Xa0sE, XaX51, E0011, E003., E0041, Eu041, X002w, X00R2, XM09N, XM09O, 1B1A0, E011., E011., E0111, E0112, E011z, E02y2, E031., Eu106, X00RH, XE1Xv, XE1YQ, F110., X002y, X002z, XaIKB, F110., F110., X002x, X002x, XaIKB, XaIKB, X0031, X0032, XaIKC, F110., F110., X0030, X0030, X00R2, X00R2, XaIKC, XaIKC, Fyu30, X0033, Eu002, Eu002, F110., F110., F110., Eu00., Eu00., Eu00z, Eu00z, F110., F110., Xa0s2, Xa0s3, F111., X0035, X0036, X003E, Eu020, Eu020, F111., F111., X0034, X0034, Xa0s2, Xa0s2, F112., F11x0, F1440, X0056, XM0qi, XM0z3, Xa7nD, F10y., F10y0, F10y1, F10yz, F11yz, Fyu31, X002U, X002V, X002W, X0037, X0039, X003F, X003G, X003W, X003X, X003m, X004B, X004E, X005L, X005M, X005N, X005O, X005P, Xa0sC, XaPws, X003A, X003A, Xa0s2, Xa0s2, XaE74, XaE74, XaKyY, XaKyY, F1..., F10.., F10z., F11.., F11xz, F11z., X005K, X77qx, XE15F, Xa0s2, Xa0s3, Xa1GB, F21y2, A411., F11x7, X003K, XaA1S, XabVp, Eu021, Eu021, XaA1S, XaA1S, XabVp, XabVp (Read v3)  **Alzheimer's disease:**  3310 (ICD-9)  F00, G30 (ICD-10)  F1100, Eu000, F1101, Eu001, Fyu30, Eu002, F110., Eu00., Eu00z (Read v2)  F110., F110., X002x, X002x, XaIKB, XaIKB, F110., F110., X0030, X0030, XaIKC, XaIKC, Eu002, Eu002, F110., F110., Eu00., Eu00., Eu00z, Eu00z, F110., F110., X00Qz, F110., X002y, X002z, XaIKB, F110., F110., X002x, X002x, XaIKB, XaIKB, X0031, X0032, XaIKC, F110., F110., X0030, X0030, X00R2, X00R2, XaIKC, XaIKC, Fyu30, X0033, Eu002, Eu002, F110., F110., F110., Eu00., Eu00., Eu00z, Eu00z, F110., F110. (Read v3)  **Vascular dementia:**  2904 (ICD-9)  F01, I673 (ICD-10)  Eu01., Eu010, Eu011, Eu012, Eu013, Eu01y, Eu01z, F21y2, G678. (Read v2)  E0040, E0041, E0042, E0043, X00Qz, XE1Xs, Xa0lH, X003R, Xa0lH, XaIRJ, X003T, X003V, Eu01y, E0040, E0041, E0042, E0043, Eu01z, XE1Xs, F21y2 (Read v3) |
| Yoo JE, 2024 | KNHIS database | **Alzheimer’s disease:**  F00, G30 (ICD-10)  **Vascular dementia:**  F01 (ICD-10)  **Other dementias:**  F02, F03, G23.1, G31 (ICD-10) |
|  |  | **Acetylcholinesterase inhibitors:**  Donepezil hydrochloride, rivastigmine, and galantamine  **N-methyl-D-aspartate receptor antagonist:**  Memantine |
| Hendriks S, 2024 | UK Biobank | **Alzheimer’s disease:**  331.0 (ICD-9)  F00., F00.0, F00.1, F00.2, F00.9, G30, G30.0, G30.1, G30.8, G30.9 (ICD-10)  **Vascular dementia:**  290.4 (ICD-9)  F01, F01.0, F01.1, F01.2, F01.3, F01.8, F01.9, I67.3 (ICD-10)  **Frontotemporal dementia:**  331.1 (ICD-9)  F02.0, G31.0 (ICD-10)  **Other codes for all-cause dementia:**  290.2, 290.3, 291.2, 294.1, 331.2, 331.5 (ICD-9)  A81.0, F02, F02.1, F02.2, F02.3, F02.4, F02.8, F03, F05.1, F10.6, G31.1, G31.8 (ICD-10) |
| Chun MY, 2024 | KNHIS database | **Dementia in Alzheimer disease:**  F00.0, F00.1, F00.2, F00.9 (ICD-10)  **Alzheimer disease:**  G30.0, G30.1, G30.8, and G30.9 (ICD-10) |
|  |  | **Prescriptions for anti-dementia medications (e.g., donepezil, galantamine, rivastigmine, memantine):**  148602ATB, 148601ATB, 148602ATD, 148601ATD, 148603ATB, 643402ATD, 643401ATD, 385203ATR, 385204ATR, 385205ATR, 385204ACR, 385203ACR, 385205ACR, 224507CPC, 224506CPC, 224503ACH, 224501ACH, 224508CPC, 224504ACH, 224505ACH, 190004ATB, 190001ATB, 190002ASY, 190004ATD, 190003ATD, 190001ALQ, 190030ASY, 190031ALQ (ATC-codes) |
| Shang X, 2023 | UK Biobank | **Dementia:**  331.0, 290.4, 331.1, 290.2, 290.3, 291.2, 294.1, 331.2, 331.5 (ICD-9)  F00, F00.0, F00.1, F00.2, F00.9, G30, G30.0, G30.1, G30.8, G30.9, F01, F01.0, F01.1, F01.2, F01.3, F01.8, F01.9, I67.3, F02.0, G31.0, A81.0, F02, F02.1, F02.2, F02.3, F02.4, F02.8, F03, F05.1, F10.6, G31.1, G31.8 (ICD-10)  1263 (Self-reported fields)  **Alzheimer’s disease:**  331.0 (ICD-9)  F00, F00.0, F00.1, F00.2, F00.9, G30, G30.0, G30.1, G30.8, G30.9 (ICD-10)  **Vascular dementia:**  290.4 (ICD-9)  F01, F01.0, F01.1, F01.2, F01.3, F01.8, F01.9 (ICD-10) |
| Li R, 2023 | UK Biobank | **Dementia:**  290.2, 290.3, 290.4, 291.2, 294.1, 331.0, 331.1, 331.2, 331.5 (ICD-9)  A81.0, F00, F00.0, F00.1, F00.2, F00.9, F01, F01.0, F01.1, F01.2, F01.3, F01.8, F01.9, F02, F02.0, F02.1, F02.2, F02.3, F02.4, F02.8, F03, F05.1, F10.6, G30, G30.0, G30.1, G30.8, G30.9, G31.0, G31.1, G31.8, I67.3 (ICD-10) |
|  |  | **Medical history information on dementia-specific prescriptions at baseline (identify prevalent dementia cases at baseline):**  Memantine, donepezil, galantamine, and rivastigmine |
| Hu Y, 2023 | Finnish Hospital Discharge Register | **Dementia:**  290, 291.2A, 292.8C, 294.1A, 331.0, 331.1A, 437.8A (ICD-9)  F00-F03, G30 (ICD-10)  **Alzheimer’s disease:**  331.0 (ICD-9)  F00, G30 (ICD-10) |
|  | Finnish Drug Reimbursement Register | **Special Reimbursement for Medical Expenses:**  307  **Prescriptions for anti-dementia medications:**  N06D (ATC-codes) |
| Wieland DR, 2022 | The Taiwanese National Health Insurance Research Database | **Dementia:**  290, 331.0, 331.1, 331.2, 331.8 (ICD-9-CM) |
| Shang X, 2022 | UK Biobank | **Dementia:**  331.0, 290.4, 331.1, 290.2, 290.3, 291.2, 294.1, 331.2, 331.5 (ICD-9)  F00, F00.0, F00.1, F00.2, F00.9, G30, G30.0, G30.1, G30.8, G30.9, F01, F01.0, F01.1, F01.2, F01.3, F01.8, F01.9, I67.3, F02.0, G31.0, A81.0, F02, F02.1, F02.2, F02.3, F02.4, F02.8, F03, F05.1, F10.6, G31.1, G31.8 (ICD-10)  1263 (Self-reported fields)  **Alzheimer’s disease:**  331.0 (ICD-9)  F00, F00.0, F00.1, F00.2, F00.9, G30, G30.0, G30.1, G30.8, G30.9 (ICD-10)  **Vascular dementia:**  290.4 (ICD-9)  F01, F01.0, F01.1, F01.2, F01.3, F01.8, F01.9 (ICD-10) |
| Mahmoudi E, 2022 | OptumInsight | **Alzheimer’s Disease and Related Dementia:**  290.0, 290.10, 290.11, 290.12, 290.13, 290.20, 290.21, 290.3, 290.40, 290.41, 290.42, 290.43, 290.8, 290.9, 294.11, 294.12, 294.20, 294.21, 331.0 (ICD-9)  F0150, F0151, F0280, F0281, F0390, F0391, G300, G301, G308, G309 (ICD-10) |
| Kuang TT, 2022 | The Taiwanese National Health Insurance Research Database | **Dementia:**  290.0, 290.10, 290.11, 290.12, 290.13, 290.20, 290.21, 290.3, 290.4, 294.1, 331.0, 331.1 (ICD-9-CM)  F02.80, F02.81, F01.50, F01.51, F03.90, F03.91, G30 (ICD-10-CM) |
| Kennedy E, 2022 | National Veterans Affairs and Department of Defense health system data | **Alzheimer’s Disease:**  331.0 (ICD-9)  G30.9 (ICD-10)  **Frontotemporal dementia:**  331.1 (ICD-9)  G31.0 (ICD-10) |
| Downing KF, 2022 | The IBM® MarketScan® Commercial Database | **Dementia:**  290.0, 290.10, 290.11, 290.12, 290.13, 290.20, 290.21, 290.3, 290.40, 290.41, 290.42, 290.43, 294.10, 294.11, 294.20, 294.21, 331.0, 331.11, 331.19, 331.2, 331.82 (ICD-9-CM)  F01.50, F01.51, F02.80, F02.81, F03.90, F03.91, G30.0, G30.1, G30.8, G30.9, G31.1, G31.01, G31.09, G31.83 (ICD-10-CM) |
| Vivanti G, 2021 | Medicaid Analytic eXtract data | **Alzheimer's Disease and Related Disorders or Senile Dementia:**  331.0, 331.11, 331.19, 331.2, 331.7, 290.0, 290.10, 290.11, 290.12, 290.13, 290.20, 290.21, 290.3, 290.40, 290.41, 290.42, 290.43, 294.0, 294.10, 294.11, 294.20, 294.21, 294.8, 797 (ICD-9) |
| Mahmoudi E, 2021 | OptumInsight | **Alzheimer’s Disease and Related Dementia:**  290.0, 290.10, 290.11, 290.12, 290.13, 290.20, 290.21, 290.3, 290.40, 290.41, 290.42, 290.43, 290.8, 290.9, 294.11, 294.12, 294.20, 294.21, 331.0 (ICD-9)  F0150, F0151, F0280, F0281, F0390, F0391, G300, G301, G308, G309 (ICD-10) |
| Cheng YF, 2021 | The Taiwanese National Health Insurance Research Database | **Dementia:**  290.0-290.4, 294.1, 331.0-331.2, 331.82 (ICD-9-CM)  F03.90 (ICD-10-CM) |
| Carcaillon-Bentata L, 2021 | The French national health data system | **≥ 2 reimbursements over a 1-year period of anti-Alzheimer drugs (acetylcholinesterase inhibitors):**  Donepezil, N06DA02 (ATC-codes)  Rivastigmine, N06DA03 (ATC-codes)  Galantamine, N06DA04 (ATC-codes)  Memantine, N06DX01 (ATC-codes) |
|  |  | **Long-term chronic diseases registration for dementia:**  F00 to F03, G30, G31.0, G31.1, F05.1 (ICD-10) |
|  |  | **≥ 1 inpatient diagnosis of dementia:**  F00 to F03, G30, G31.0, G31.1, F05.1 (ICD-10) |
| Osler M, 2020 | Danish Psychiatric Central Register, Danish National Patient Registry, and the Danish Civil Registration System | **Dementia:**  290.00-290.99 (ICD-8)  F00.0-F03.9, G30.0-G30.9 (ICD-10) |
|  | Danish National Prescription Registry | **Acetylcholinesterase inhibitor:**  N06D (ATC-codes) |
| Kivimäki M, 2020 | Health and Social Support and Finnish Public Sector studies | **Dementia:**  29000, 29010, 29011, 29019, 34791, 34792 (ICD-8)  2900A, 2941A, 3310A, 137 3311A, 3312X, 4378A (ICD-9)  F00, F01, F02, F03, G30, G31 (ICD-10) |
| Kim D, 2020 | KNHIS database | **Alzheimer’s dementia:**  F00, G30 (ICD-10)  **Vascular dementia:**  F01 (ICD-10)  **Dementia with other diseases classified elsewhere:**  F02 (ICD-10)  **Unspecified dementia:**  F03, G31 (ICD-10) |
|  |  | **Prescriptions for anti-dementia medications:**  Rivastigmine, galantamine, memantine, or donepezil |
| Han E, 2020 | KNHIS database | **Dementia:**  F00, G30, F01, F02, F03, G23.1, G31.0, G31.1, G31.82, G31.83, G31.88, F10.7 (ICD-10) |
|  |  | **Prescriptions for anti-dementia medications:**  Acetylcholinesterase inhibitor (rivastigmine, galantamine, or aricept)  N-methyl-D-aspartate receptor antagonist (memantine) |
| Rantalainen V, 2018 | Finnish Hospital Discharge and Causes of Death Registers | **Dementia:**  290.00-290.10 (ICD-8)  290, 2912A, 2928C, 2941A, 3310A, 3311A (ICD-9)  F00, F01, F03, F051, G30 (ICD-10)  **Alzheimer’s dementia:**  331.0, 290.1 (ICD-9)  G30, F00 (ICD-10) |
| Basit S, 2018 | The National Patient Register | **Dementia:**  290.00, 290.10, 290.11, 290.19, 299.99 (ICD-8)  F00.0-F02.0, F03.9, G30.0, G30.1, G30.8, G30.9 (ICD-10)  **Alzheimer’s dementia:**  F00.0-F00.9, G30.0, G30.1, G30.8, G30.9 (ICD-10)  **Vascular dementia:**  F01.0-F01.9 (ICD-10)  **Other/unspecified dementia:**  F02.0, F03.9 (ICD-10) |
| Bagge CN, 2018 | The Danish National Patient Registry | **Alzheimer’s dementia:**  290.09, 290.10 (ICD-8)  F00 series (includes F00.0x, F00.1x, F00.2x, F00.9x), G30 (includes G30, G30.0, G30.1, G30.8, G30.9) (ICD-10)  **Vascular dementia:**  293.09, 293.19 (ICD-8)  F01 series (includes F01.0x, F01.1x, F01.2x, F01.3x, F01.8x, F01.9x) (ICD-10)  **Other dementias:**  094.19, 290.11, 290.18, 290.19, 292.09 (ICD-8)  F02 series, F03 series, F1x.73 series (includes F10.73 through F19.73), G23.1, G31.0, G31.1, G31.8B, G31.8E, G31.85 (ICD-10)  **Diagnoses related to dementia (mild cognitive impairment and amnestic syndromes):**  291.19 (ICD-8)  F04, F04.9, F05.1, F06.7, F06.7x, F1x.6 (F10.6, F18.6, F19.6) (ICD-10) |
| Kadohara K, 2017 | The Japan Medical Data Center database | **Prescriptions for anti-Alzheimer’s disease medications (donepezil, memantine, rivastigmine and galantamine):**  N07 (ATC-codes) |
| Ribe AR, 2015 | The Danish Psychiatric Central Register, the Danish National Patient Register | **Alzheimer’s dementia:**  290.10 (ICD-8)  F00.0, F00.1, F00.2, F00.9, G30.0, G30.1, G30.8, G30.9 (ICD-10)  **Vascular dementia:**  293.09-19 (ICD-8)  F01.0, F01.1, F01.2, F01.3, F01.8, F01.9 (ICD-10)  **Frontotemporal dementia:**  290.11 (ICD-8)  F02.0 (ICD-10)  **Dementia without specification:**  290.09-19 (ICD-8)  F03.9 (ICD-10) |
|  | The Danish National Prescription Registry | **Anticholinesterases:**  Tacrine, N06DA01 (ATC-codes)  Donepezile, N06DA02 (ATC-codes)  Rivastigmine, N06DA03 (ATC-codes)  Galantamine, N06DA04 (ATC-codes)  Donepezile and Memantine, N06DA52 (ATC-codes)  **Other anti-dementia drugs:**  Memantine, N06DX1 (ATC-codes) |
| Heath CA, 2015 | Electronic medical records in UK general practitioners | **Read Codes (the standard morbidity coding used by NHS general practices):**  66h.., 6AB.., E00.., E000., E001., E0010, E0011, E0012, E0013, E001z, E002., E0020, E0021, E002z, E003., E004., E0040, E0041, E0042, E0043, E004z, E00y., E00z., E041.,  Eu00., Eu000, Eu001, Eu002, Eu00z, Eu01., Eu010, Eu011, Eu012, Eu013, Eu01y, Eu01z, Eu02., Eu020, Eu021, Eu022, Eu023, Eu024, Eu025, Eu02y, Eu02z, F110., F1100, F1101, F111., F112., F116., Fyu30 |
|  |  | **Prescriptions for anti-dementia medications:**  Donepezil, galantamine, rivastigmine, and memantine |
| Nyberg J, 2014 | The Swedish National Hospital Discharge Register | **Dementia:**  290, 294, 331 (ICD-9)  F00-F03, G30, G31, G91.2 (ICD-10) |
| Nordström P, 2014 | The National Patient Register and/or other registers | **Alzheimer’s dementia:**  F00.x, G30.x, 290.x  **Vascular dementia:**  F01.x  **Alcohol dementia:**  F10.7A  **Dementia of unspecified type:**  F03.9 |
| Wu KY, 2013 | The Taiwanese National Health Insurance Research Database | **Dementia:**  290.0-290.4, 331.0-331.2, 294.1, 294.10, 294.11 (ICD-9-CM) |
| Nordström P, 2013 | The Swedish National Hospital Discharge Patient Register | **Alzheimer’s dementia:**  F00.x, G30.x, 290.x  **Vascular dementia:**  F01.x  **Alcohol dementia:**  F10.7A  **Dementia of unspecified type:**  F03.9  **Dementia associated with Parkinson disease:**  F02.3  **Lewy body dementia:**  G31.8A |

# Supplementary Table 7. Quality assessment for case-control studies included in the quantitative analysis using the Newcastle-Ottawa Scale^*^

| **Study** | **Selection** | | | | **Comparability** | | **Exposure** | | | **Overall** |
| --- | --- | --- | --- | --- | --- | --- | --- | --- | --- | --- |
| Park D, 2025 | ⚝ | ⚝ | ⚝ | ⚝ | **-** | **-** | ⚝ | ⚝ | - | 6 |
| Mazzoli R, 2024 | ⚝ | - | ⚝ | ⚝ | **-** | ⚝ | ⚝ | ⚝ | - | 6 |
| Damsgaard L, 2024_6 | - | ⚝ | ⚝ | ⚝ | **-** | ⚝ | ⚝ | ⚝ | - | 6 |
| Damsgaard L, 2024_7 | ⚝ | ⚝ | ⚝ | ⚝ | **-** | ⚝ | ⚝ | ⚝ | - | 7 |
| Kennedy E, 2022 | ⚝ | ⚝ | ⚝ | ⚝ | **-** | ⚝ | ⚝ | ⚝ | - | 7 |
| Filippini T, 2020 | - | - | ⚝ | ⚝ | **-** | ⚝ | ⚝ | ⚝ | - | 5 |
| Adani G, 2020 | - | - | ⚝ | ⚝ | **-** | ⚝ | ⚝ | ⚝ | - | 5 |
| Keret O, 2018 | ⚝ | - | ⚝ | ⚝ | **-** | ⚝ | ⚝ | ⚝ | - | 6 |
| Cations M, 2018 | ⚝ | - | ⚝ | ⚝ | ⚝ | ⚝ | ⚝ | ⚝ | - | 7 |
| Kadohara K, 2017 | ⚝ | ⚝ | ⚝ | ⚝ | **-** | **-** | ⚝ | ⚝ | - | 6 |
| Chen Y, 2017 | ⚝ | - | ⚝ | ⚝ | ⚝ | ⚝ | ⚝ | ⚝ | - | 7 |
| Mendez MF, 2015 | - | ⚝ | ⚝ | ⚝ | **-** | ⚝ | ⚝ | ⚝ | - | 6 |
| van Duijn CM, 1992 | ⚝ | - | ⚝ | ⚝ | ⚝ | ⚝ | ⚝ | ⚝ | - | 7 |
| van Duijn CM, 1991 | ⚝ | - | ⚝ | ⚝ | **-** | **-** | ⚝ | ⚝ | - | 5 |

*A study can be awarded a maximum of one star for each numbered item within the Selection and Exposure categories. A maximum of two stars can be given for Comparability.

Note: Selection 1) Is the case definition adequate? Selection 2) Representativeness of the cases Selection; 3) Selection of controls; Selection 4) Definition of controls; Comparability 1) Study controls for apolipoprotein E status (or family history of dementia); Comparability 2) Study controls for age, sex, and educational level (or socioeconomic status); Exposure 1) Ascertainment of exposure; Exposure 2) Same method of ascertainment for cases and controls; Exposure 3) Non-Response rate

# Supplementary Table 8. Quality assessment for studies not included in the quantitative analysis using the Newcastle-Ottawa Scale

| **Study** | **Selection** | | | | **Comparability** | | **Outcome/Exposure** | | |
| --- | --- | --- | --- | --- | --- | --- | --- | --- | --- |
| Yuan S, 2024 | ⚝ | ⚝ | ⚝ | ⚝ | ⚝ | ⚝ | ⚝ | **-** | **-** |
| Olié V, 2024 | ⚝ | ⚝ | - | ⚝ | **-** | **-** | **-** | ⚝ | **-** |
| Clouston SAP, 2024 | ⚝ | ⚝ | ⚝ | ⚝ | **-** | ⚝ | ⚝ | **-** | **-** |
| Shang X, 2023 | ⚝ | ⚝ | ⚝ | ⚝ | **-** | **-** | ⚝ | **-** | **-** |
| Mahmoudi E, 2021 | - | ⚝ | ⚝ | ⚝ | **-** | ⚝ | ⚝ | **-** | **-** |
| Basit S, 2018 | ⚝ | ⚝ | ⚝ | ⚝ | **-** | ⚝ | ⚝ | **-** | **-** |
| Bagge CN, 2018 | ⚝ | ⚝ | ⚝ | ⚝ | **-** | **-** | ⚝ | **-** | **-** |
| Phung TK, 2010 | ⚝ | ⚝ | ⚝ | ⚝ | **-** | **-** | ⚝ | **-** | ⚝ |
| Mazzoleni E, 2023 | - | - | ⚝ | ⚝ | **-** | ⚝ | ⚝ | ⚝ | **-** |
| Damsgaard L, 2023 | - | ⚝ | ⚝ | ⚝ | **-** | ⚝ | ⚝ | ⚝ | - |
| Wieland DR, 2022 | ⚝ | ⚝ | ⚝ | ⚝ | **-** | **-** | ⚝ | ⚝ | - |
| Kuang TT, 2022 | ⚝ | ⚝ | ⚝ | ⚝ | **-** | ⚝ | ⚝ | ⚝ | - |
| Cheng YF, 2021 | ⚝ | ⚝ | ⚝ | ⚝ | **-** | ⚝ | ⚝ | ⚝ | - |
| Wu KY, 2013 | ⚝ | ⚝ | ⚝ | ⚝ | **-** | **-** | ⚝ | ⚝ | - |
| Slooter AJ, 1999 | ⚝ | - | ⚝ | ⚝ | **-** | ⚝ | ⚝ | ⚝ | - |
| van Duijn CM, 1995 | ⚝ | - | ⚝ | ⚝ | ⚝ | **-** | ⚝ | ⚝ | - |

# Supplementary Table 9. Pooled relative risks of predictors for young-onset dementia

| **Risk factors** | **Reference/Comparison/Effect size (95% CI)** | **YOD cases** | **Participants^#^** | **Study design** | **Random-effects summary relative risk (95% CI)** | ***p* random** | **I^2^ (95% CI)** | **Egger test *p* value** |
| --- | --- | --- | --- | --- | --- | --- | --- | --- |
| **Demographic characteristics** | | | | | | | | |
| Older age | 1. [11]/continuous/1.17 (1.14-1.19) 2. [19]/continuous/1.08 (1.08-1.08) 3. [27]/continuous/1.03 (0.97-1.08) 4. [29]/continuous/1.19 (1.14-1.24) | >689 | 714,537 | CS (2), CCS (2) | 1.12 (1.05-1.18) | 2.2×10^-4^ | 96% (93%-98%) | 0.35 |
| Female sex | 1. [11]/male vs. female/1.54 (1.29-1.84) 2. [19]/female vs. male/0.99 (0.96-1.02) 3. [51]/male vs. female/1.48 (1.39-1.57) 4. [27]/female vs. male/0.98 (0.45-2.12) 5. [29]/female vs. male/1.44 (0.96-2.16) | >4,763 | >714,537 | CS (2), CCS (2), CSS (1) | 0.86 (0.66-1.13) | 0.28 | 92% (87%-95%) | 0.65 |
| Higher educational attainment | 1. [11]/low vs. high/3.76 (2.90-4.87) 2. [22]/high vs. low/0.88 (0.77-1.00) 3. [27]/high vs. low/0.43 (0.20-0.94) 4. [30]/continuous/0.87 (0.79-0.95) 5. [29]/continuous/0.83 (0.78-0.88) 6. [34]/high vs. low/0.82 (0.75-0.89) 7. [40]/ low vs. high/1.02 (0.83-1.25) 8. [45]/low vs. high/1.70 (1.10-2.70) | 5,736 | > 748,924 | CS (3), CCS (5) | 0.71 (0.60-0.85) | 1.2×10^-4^ | 92% (87%-95%) | 0.24 |
| **Cardiovascular risk factors** | | | | | | | | |
| Smoking | 1. [11]/smoking vs. no smoking/1.46 (1.22-1.74) 2. [27]/current smoking vs. never smoking/1.01 (0.37-2.79) 3. [29]/smoking vs. no smoking/0.58 (0.35-0.96) 4. [30]/current smoking vs. never smoking/2.39 (0.86-6.62) 5. [34]/current smoking vs. never smoking/0.42 (0.15-1.17) 6. [46]/smoking vs. no smoking/0.70 (0.43-1.15) 7. [add_2]/current smoker vs. non-smoker/0.906 (0.806-1.020) | 6,237 | 270,782 | CS (1), CCS (6) | 0.93 (0.67-1.28) | 0.65 | 81% (57%-89%) | 0.76 |
| Alcohol consumption | 1. [11]/excessive alcohol intake vs. no excessive alcohol intake/1.55 (1.30-1.86) 2. [26]/alcohol intake (T3 vs. T1)/1.04 (0.37-2.93) 3. [30]/excessive alcohol intake vs. no excessive alcohol intake/3.36 (0.88-12.9) 4. [34]/alcohol intake (yes vs. no)/0.08 (0.02-0.28) 5. [add_2]/alcohol consumption (heavy vs. no)/1.149 (0.928–1.422) | 5,911 | 268,450 | CS (1), CCS (4) | 1.07 (0.65-1.75) | 0.80 | 83% (54%-91%) | 0.47 |
| Alcohol use disorders | 1. [7]/alcohol-related mental disorders (+ vs. -)/2.12 (1.62-2.79) 2. [12]/alcohol use disorders (+ vs. -)/5.49 (4.23-7.13) for male; 6.08 (4.15-8.89) for female 3. [16]/alcohol problems (+ vs. -)/3.20 (1.66-6.17) 4. [22]/alcohol abuse (+ vs. -)/3.87 (3.22-4.66) 5. [23]/alcohol-induced loss of consciousness (+ vs. -)/2.21 (1.46-3.34) 6. [40]/alcohol intoxication (+ vs. -)/4.82 (3.83-6.05) 7. [54]/alcohol disorders (+ vs. -)/7.52 (6.29-8.99) | >7,773 | >1,149,437 | CS (5), CCS (1), CSS (1) | 4.11 (2.97-5.68) | 1.3×10^-17^ | 91% (85%-95%) | 0.33 |
| Obesity | 1. [7]/obesity (+ vs. -)/0.59 (0.43-0.81) 2. [16]/obesity (+ vs. -)/1.44 (1.06-1.96) 3. [51]/morbid obesity (+ vs. -)/1.75 (1.57-1.95) 4. [34]/body mass index (continuous)/0.90 (0.85-0.95) 5. [add_1]/morbid obesity vs. normal weight/1.26 (1.10-1.45) | 10,920 | > 1,349,138 | CS (2), CCS (2), CSS (1) | 1.12 (0.79-1.58) | 0.52 | 97% (96%-98%) | 0.60 |
| Hypertension | 1. [8]/hypertension (+ vs. -)/1.15 (1.03-1.28) 2. [4]/hypertension (+ vs. -)/0.54 (0.23-1.24) 3. [7]/essential hypertension (+ vs. -)/1.38 (1.19-1.60) 4. [12]/hypertension (+ vs. -)/0.91 (0.71-1.16) for male; 0.84 (0.67-1.05) for female 5. [16]/hypertension (+ vs. -)/1.43 (1.16-1.77) 6. [49]/hypertension (+ vs. -)/1.42 (1.39-1.46) 7. [29]/hypertension (+ vs. -)/0.94 (0.61-1.45) 8. [30]/hypertension (+ vs. -)/2.13 (0.98-3.72) 9. [53]/hypertension (+ vs. -)/1.40 (1.20-1.60) | 31,891 | 8,003,327 | CS (3), CCS (4), CSS (2) | 1.20 (1.06-1.36) | 5.1×10^-3^ | 84% (71%-91%) | 0.05 |
| Diabetes | 1. [4]/diabetes (+ vs. -)/2.72 (0.67-10.97) 2. [7]/type 2 diabetes mellitus (+ vs. -)/1.20 (0.96-1.51) 3. [12]/diabetes (+ vs. -)/1.18 (0.86-1.62) for male; 1.34 (0.95-1.90) for female 4. [16]/diabetes (+ vs. -)/1.84 (1.25-2.69) 5. [49]/diabetes (+ vs. -)/1.92 (1.87-1.98) 6. [51]/diabetes (+ vs. -)/1.36 (1.24-1.49) 7. [25]/prevalent diabetes (+ vs. -)/1.73 (1.66-1.81) 8. [29]/diabetes (+ vs. -)/2.93 (1.73-4.96) 9. [33]/diabetes (+ vs. -)/1.31 (0.90-1.92) 10. [34]/diabetes (+ vs. -)/2.35 (0.57-9.66) 11. [53]/diabetes (+ vs. -)/2.00 (1.70-2.50) | 53,889 | >7,584,086 | CS (3), CCS (5), CSS (3) | 1.61 (1.44-1.80) | 8.9×10^-17^ | 88% (80%-92%) | 0.21 |
| Disorders of lipoprotein metabolism | 1. [8]/hyperlipidemia (+ vs. -)/0.95 (0.85-1.07) 2. [4]/dyslipidemia (+ vs. -)/1.43 (0.63-3.27) 3. [7]/disorders of lipoprotein metabolism (+ vs. -)/1.64 (1.35-1.99) 4. [12]/hyperlipidemia (+ vs. -)/0.84 (0.63-1.11) for male; 1.17 (0.91-1.52) for female 5. [16]/disorders of lipoprotein metabolism and other lipidaemias (+ vs. -)/1.33 (1.02-1.73) 6. [30]/hypercholesterolemia (+ vs. -)/ 2.53 (0.70-9.13) 7. [34]/hypercholesterolemia (+ vs. -)/2.03 (0.96-4.32) | 5,565 | 987,067 | CS (3), CCS (4) | 1.24 (0.98-1.56) | 0.07 | 79% (58%-89%) | 0.23 |
| **Cerebrovascular/cardiovascular diseases** | | | | | | | | |
| Stroke | 1. [4]/stroke (+ vs. -)/4.01 (0.41-39.19) 2. [16]/stroke (+ vs. -)/4.06 (2.80-5.88) 3. [30]/stroke or transient ischemic attack (+ vs. -)/13.18 (2.62-66.40) 4. [53]/stroke or transient ischemic attack (+ vs. -)/3.10 (2.40-4.00) 5. [40]/stroke (+ vs. -)/2.96 (2.02-4.35) | 2,206 | 1,482,294 | CS (2), CCS (2), CSS (1) | 3.38 (2.74-4.17) | 7.1×10^-30^ | 12% (0%-82%) | 0.25 |
| Transient ischemic attack | 1. [7]/transient ischemic attack (+ vs. -)/1.56 (1.05-2.31) 2. [30]/stroke or transient ischemic attack (+ vs. -)/13.18 (2.62-66.40) 3. [53]/stroke or transient ischemic attack (+ vs. -)/3.10 (2.40-4.00) 4. [54]/transient ischemic attack (+ vs. -)/1.92 (1.22-3.04) | >2,902 | >623,500 | CCS (2), CSS (2) | 2.45 (1.49-4.04) | 4.1×10^-4^ | 78% (40%-92%) | 0.87 |
| Atrial fibrillation | 1. [5]/atrial fibrillation (+ vs. -)/1.00 (0.42-2.45) 2. [4]/atrial fibrillation (+ vs. -)/2.07 (0.35-12.27) 3. [24]/atrial fibrillation (+ vs. -)/3.81 (2.75-5.29) | 1,655 | 775,486 | CS (2), CCS (1) | 2.12 (0.77-5.82) | 0.15 | 75% (17%-93%) | 0.47 |
| Ischemic heart disease | 1. [7]/chronic ischemic heart disease (+ vs. -)/1.11 (0.85-1.44) 2. [51]/chronic ischemic heart disease or history of acute ischemic heart disease (+ vs. -)/1.55 (1.36-1.76) 3. [53]/ischemic heart disease (+ vs. -)/1.90 (1.50-2.40) | 6,880 | >623,225 | CCS (1), CSS (2) | 1.50 (1.16-1.93) | 1.7×10^-3^ | 78% (28%-93%) | 0.84 |
| Heart diseases | 1. [5]/heart disease (+ vs. -)/ 1.61 (1.10-2.36) 2. [6]/cardiac therapy (+ vs. -)/0.92 (0.75-1.13) 3. [56]/cardiac disease (+ vs. -)/1.36 (1.10-1.67) | 3,203 | 364,978 | CS (1), CCS (2) | 1.23 (0.89-1.71) | 0.21 | 80% (37%-94%) | 0.60 |
| Cardiovascular diseases | 1. [6]/prescription medication use in the cardiovascular system/1.07 (0.95-1.20) 2. [12]/cardiovascular diseases/1.43 (1.12-1.83) male; 1.11 (0.87-1.43) for female 3. [54]/procedures on cardiovascular system/ 0.23 (0.16-0.32) | >2,437 | >187,829 | CS (1), CCS (1), CSS (1) | 0.80 (0.46-1.42) | 0.45 | 96% (93%-98%) | 0.50 |
| **Mental and behavioral disorders** | | | | | | | | |
| Depression | 1. [2]/depression (+ vs. -)/ 2.67 (2.32-3.07) for premenopausal women; 2.50 (2.34-2.67) for postmenopausal women 2. [7]/depressive episode (+ vs. -)/3.48 (2.54-4.77) 3. [16]/depression (+ vs. -)/2.59 (2.05-3.28) 4. [56]/depression (+ vs. -)/1.35 (1.12-1.63) 5. [19]/depression (+ vs. -)/1.76 (1.71-1.82) 6. [22]/depression (+ vs. -)/1.87 (1.39-2.8) 7. [30]/depression (+ vs. -)/3.36 (1.46-7.73) 8. [40]/depression or use of antidepressants/1.89 (1.53-2.34) | >17,166 | >2,951,354 | CS (5), CCS (3) | 2.19 (1.83-2.63) | 2.6×10^-17^ | 95% (92%-97%) | 0.24 |
| Schizophrenia | 1. [7]/schizophrenia, schizotypal and delusional disorders (+ vs. -)/2.28 (1.46-3.57) 2. [49]/schizophrenia (+ vs. -)/16.71 (16.03-17.41) 3. [16]/schizophrenia (+ vs. -)/3.77 (2.23-6.38) 4. [51]/schizophrenia and psychotic disorders (+ vs. -)/7.12 (6.36-7.97) 5. [52]/schizophrenia (+ vs. -)/3.77 (3.29-4.33) | 40,311 | >6,782,677 | CS (2), CCS (1), CSS (2) | 5.31 (2.53-11.14) | 9.8×10^-6^ | 99% (99%-100%) | 0.10 |
| Mood/affective disorders | 1. [7]/mood/affective disorders (+ vs. -)/3.14 (2.48-3.98) 2. [49]/mental health conditions (+ vs. -)/3.26 (3.18-3.34) 3. [19]/mental health disorders (+ vs. -)/2.14 (2.05-2.23) | >26,983 | 6,860,603 | CS (1), CCS (1), CSS (1) | 2.78 (1.98-3.91) | 3.3×10^-9^ | 99% (99%-100%) | 0.77 |
| **Cognitive abilities and neurodegenerative disorders** | | | | | | | | |
| Lower cognitive ability in young adulthood | 1. [22]/highest tertile cognitive ability vs. lowest tertile cognitive ability/0.51 (0.46-0.56) 2. [28]/continuous/1.77 (1.07-2.95) 3. [37]/low cognitive performance vs. high cognitive performance/ 3.82 (2.96-4.93) | 4,841 | >1,174,916 | CS (3) | 2.40 (1.47-3.95) | 5.2×10^-4^ | 92% (78%-97%) | 0.68 |
| Epilepsy | 1. [6]/use of antiepileptic drugs (+ vs. -)/1.05 (0.88-1.26) 2. [16]/epilepsy (+ vs. -)/2.97 (1.88-4.67) 3. [56]/epilepsy (+ vs. -)/4.80 (3.30-6.97) 4. [49]/epilepsy (+ vs. -)/11.35 (10.91-11.81) 5. [51]/epilepsy (+ vs. -)/16.06 (14.43-17.89) 6. [54]/epilepsy (+ vs. -)/10.17 (7.93-13.05) | >32,534 | >6,784,623 | CS (1), CCS (2), CSS (3) | 5.55 (2.72-11.29) | 2.3×10^-6^ | 99% (99%-100%) | 0.31 |
| Parkinson’s disease | 1. [16]/Parkinson’s disease (+ vs. -)/20.67 (11.56-36.96) 2. [49]/Parkinsonism (+ vs. -)/12.53 (11.84-13.27) 3. [51]/Parkinson’s disease (+ vs. -)/26.21 (23.15-29.68) 4. [53]/Parkinson’s disease (+ vs. -)/8.70 (4.40-16.90) | 30,877 | >7,391,942 | CS (1), CSS (3) | 16.08 (9.42-27.43) | 2.3×10^-24^ | 97% (96%-99%) | 0.65 |
| Multiple sclerosis | 1. [16]/multiple sclerosis (+ vs. -)/3.12 (1.48-6.59) 2. [17]/multiple sclerosis (+ vs. -)/4.25 (3.40-5.32) 3. [49]/multiple sclerosis (+ vs. -)/10.27 (9.55-11.03) 4. [51]/multiple sclerosis (+ vs. -)/8.99 (7.35-10.97) 5. [53]/multiple sclerosis (+ vs. -)/1.80 (0.90-3.40) | 32890 | >7907480 | CS (2), CSS (3) | 5.14 (3.18-8.32) | 2.4×10^-11^ | 95% (92%-97%) | 0.07 |
| **Medication use** | | | | | | | | |
| Use of antihypertensives | 1. [6]/antihypertensives (use vs. no use)/0.94 (0.84-1.06) 2. [51]/antihypertensives (use vs. no use)/1.06 (0.99-1.13) 3. [33]/antihypertensives (use vs. no use)/0.84 (0.61-1.15) 4. [34]/antihypertensives (use vs. no use)/1.93 (1.06-3.51) | 6,292 | >9,243 | CCS (3), CSS (1) | 1.01 (0.87-1.17) | 0.87 | 66% (1%-89%) | 0.95 |
| Use of antidiabetics | 1. [6]/antidiabetics (use vs. no use)/1.03 (0.86-1.24) 2. [25]/use of antidiabetics (more than 3 years) vs. normoglycemia/1.70 (1.62-1.78) 3. [40]/antidiabetics (use vs. no use)/0.77 (0.55-1.09) | 22,147 | >495,428 | CS (2), CCS (1) | 1.13 (0.71-1.80) | 0.61 | 96% (90%-98%) | 0.95 |
| Use of antidepressants | 1. [6]/antidepressants (use vs. no use)/2.46 (2.19-2.78) 2. [33]/antidepressants (use vs. no use)/4.27 (2.68-6.81) 3. [40]/depression or use of antidepressants (+ vs. -)/1.89 (1.53-2.34) | 2,603 | 497,283 | CS (1), CCS (2) | 2.52 (1.85-3.44) | 4.5×10^-9^ | 82% (43%-94%) | 0.77 |
| Use of antipsychotics | 1. [6]/antipsychotics (use vs. no use)/2.08 (1.7-2.54) 2. [33]/antipsychotics (use vs. no use)/3.19 (2.26-4.50) 3. [40]/neuroleptics (use vs. no use)/2.75 (2.09-3.60) | 2,603 | 497,283 | CS (1), CCS (2) | 2.56 (1.98-3.31) | 6.2×10^-13^ | 64% (0%-90%) | 0.11 |
| **Other factors** | | | | | | | | |
| Traumatic brain injury | 1. [56]/traumatic brain injury (+ vs. -)/3.05 (2.42-3.83) 2. [51]/traumatic brain injury (+ vs. -)/14.02 (12.35-15.92) 3. [22]/traumatic brain injury (+ vs. -)/5.49 (4.97-6.06) 4. [27]/head trauma (+ vs. -)/1.54 (0.47-4.99) 5. [36]/traumatic brain injury (+ vs. -)/1.28 (1.00-1.57) 6. [38]/traumatic brain injury (+ vs. -)/2.30 (1.50-3.60) 7. [45]/head trauma (+ vs. -)/ 10.00 (1.00-96.80) | 11460 | >816943 | CS (2), CCS (4), CSS (1) | 3.58 (1.77-7.26) | 4.1×10^-4^ | 99% (98%-99%) | 0.40 |
| Fractures | 1. [7]/fracture of forearm (+ vs. -)1.45 (1.15-1.84) 2. [16]/fracture (+ vs. -)/5.10 (1.90-13.67) 3. [22]/fracture (+ vs. -)/1.92 (1.78-2.06) 4. [54]/fracture (+ vs. -)/1.08 (0.84-1.40) | >6,413 | >384,194 | CS (2), CCS (1), CSS (1) | 1.64 (1.15-2.34) | 6.0×10^-3^ | 88% (73%-95%) | 0.66 |
| Chronic obstructive pulmonary disease | 1. [7]/chronic obstructive pulmonary disease (+ vs.-)/1.06 (0.79-1.41) 2. [16]/chronic obstructive pulmonary disease (+ vs.-)/2.92 (1.37-6.22) 3. [49]/chronic obstructive pulmonary disease (+ vs.-)/2.09 (2.03-2.15) | 27,487 | 6,782,677 | CS (1), CCS (1), CSS (1) | 1.76 (1.02-3.03) | 0.04 | 91% (76%-97%) | 0.64 |
| Asthma | 1. [7]/asthma (+ vs.-)/1.09 (0.77-1.54) 2. [16]/asthma (+ vs.-)/1.51 (1.20-1.90) 3. [49]/asthma (+ vs.-)/1.45 (1.40-1.50) | 27,487 | 6,782,677 | CS (1), CCS (1), CSS (1) | 1.43 (1.28-1.58) | 3.2×10^-11^ | 26% (0%-92%) | 0.62 |

^#^Refers to all individuals included in the young-onset dementia analysis.

Abbreviation: CCS, case-control study; CI, confidence interval; CS, cohort study; CSS, cross-sectional study; YOD, young-onset dementia.

# Supplementary Table 10. The quality of evidence for the associations between modifiable risk factors and young-onset dementia

| **Risk factors** | **95% PI** | **Largest study** | | **Excess significance** | | **Random-effects summary estimate with *p* value under a 10% credibility ceiling** | **Class of evidence** |
| --- | --- | --- | --- | --- | --- | --- | --- |
|  |  | **Effect size (95% CI)** | **SE** | **O/E** | ***p* value** |  |  |
| **Demographic characteristics** | | | | | | | |
| Older age | 0.85-1.47 | 1.08 (1.08-1.08) | 1.2×10^-3^ | 3/NA | NA | 1.05 (1.00-1.10); 0.052 | Weak evidence (class IV) |
| Female sex | 0.34-2.22 | 0.99 (0.96-1.02) | 0.02 | 2/NA | NA | 0.97 (0.81-1.15); 0.70 | Not significant |
| Higher educational attainment | 0.40-1.27 | 0.83 (0.78-0.88) | 0.03 | 7/NA | NA | 0.88 (0.79-0.97); 0.01 | Suggestive evidence (class III) |
| **Cardiovascular risk factors** | | | | | | | |
| Smoking | 0.37-2.32 | 0.91 (0.81-1.02) | 0.06 | 2/1.34 | 0.63 | 0.91 (0.71-1.18); 0.47 | Not significant |
| Alcohol consumption | 0.27-4.21 | 1.55 (1.30-1.86) | 0.09 | 2/3.28 | NP | 1.18 (0.97-1.44); 0.10 | Not significant |
| Alcohol use disorders | 1.31-12.87 | 7.52 (6.29-8.99) | 0.09 | 8/NA | NA | 2.96 (1.58-5.55); 0.001 | Highly suggestive evidence (class II) |
| Obesity | 0.35-3.58 | 0.90 (0.85-0.95) | 0.03 | 5/NA | NA | 1.08 (0.81-1.43); 0.59 | Not significant |
| Hypertension | 0.80-1.80 | 1.42 (1.39-1.46) | 0.01 | 6/7.96 | NP | 1.07 (0.92-1.25); 0.38 | Weak evidence (class IV) |
| Diabetes | 1.15-2.25 | 1.92 (1.87-1.98) | 0.02 | 6/NA | NA | 1.33 (1.14-1.54); 2.4×10^-4^ | Highly suggestive evidence (class II) |
| Disorders of lipoprotein metabolism | 0.62-2.47 | 0.95 (0.85-1.07) | 0.06 | 2/0.88 | 0.22 | 1.08 (0.91-1.28); 0.36 | Not significant |
| **Cerebrovascular/cardiovascular diseases** | | | | | | | |
| Stroke | 2.17-5.26 | 3.10 (2.40-4.00) | 0.13 | 4/4.90 | NP | 3.63 (1.44-9.14); 0.006 | Convincing evidence (class I) |
| Transient ischemic attack | 0.30-20.03 | 3.10 (2.40-4.00) | 0.13 | 4/NA | NA | 1.83 (1.08-3.11); 0.03 | Suggestive evidence (class III) |
| Atrial fibrillation | 0.00-214752.91 | 3.81 (2.75-5.29) | 0.17 | 1/2.97 | NP | 1.35 (0.65-2.82); 0.43 | Not significant |
| Ischemic heart disease | 0.08-29.18 | 1.55 (1.36-1.76) | 0.07 | 2/NA | NA | 1.20 (0.94-1.52); 0.14 | Weak evidence (class IV) |
| Heart diseases | 0.03-57.72 | 0.92 (0.75-1.13) | 0.10 | 2/0.73 | 0.15 | 1.13 (0.80-1.60); 0.47 | Not significant |
| Cardiovascular diseases | 0.05-12.34 | 1.07 (0.95-1.2) | 0.06 | 2/NA | NA | 1.09 (0.98-1.20); 0.13 | Not significant |
| **Mental and behavioral disorders** | | | | | | | |
| Depression | 1.18-4.09 | 1.76 (1.71-1.82) | 0.02 | 9/NA | NA | 1.73 (1.26-2.36); 0.001 | Highly suggestive evidence (class II) |
| Schizophrenia | 0.29-96.56 | 16.71 (16.03-17.41) | 0.02 | 5/NA | NA | 3.31 (1.37-8.02); 0.008 | Suggestive evidence (class III) |
| Mood/affective disorders | 0.04-207.23 | 3.26 (3.18-3.34) | 0.01 | 3/NA | NA | 2.58 (1.10-6.05); 0.03 | Highly suggestive evidence (class II) |
| **Cognitive abilities and neurodegenerative disorders** | | | | | | | |
| Lower cognitive ability in young adulthood | 0.01-1071.21 | 1.96 (1.79-2.17) | 0.05 | 3/NA | NA | 1.98 (1.05-3.73); 0.04 | Suggestive evidence (class III) |
| Epilepsy | 0.40-77.44 | 11.35 (10.91-11.81) | 0.02 | 5/NA | NA | 2.36 (0.90-6.17); 0.08 | Suggestive evidence (class III) |
| Parkinson’s disease | 1.37-188.61 | 12.53 (11.84-13.27) | 0.03 | 4/NA | NA | 13.57 (1.80-102.38); 0.01 | Highly suggestive evidence (class II) |
| Multiple sclerosis | 0.86-30.69 | 10.27 (9.55-11.03) | 0.04 | 4/NA | NA | 2.50 (1.22-5.13); 0.01 | Highly suggestive evidence (class II) |
| **Medication use** | | | | | | | |
| Use of antihypertensives | 0.58-1.78 | 1.06 (0.99-1.13) | 0.03 | 1/NA | NA | 0.99 (0.88-1.12); 0.89 | Not significant |
| Use of antidiabetics | 0.00-409.89 | 1.70 (1.62-1.78) | 0.02 | 1/NA | NA | 0.99 (0.75-1.32); 0.97 | Not significant |
| Use of antidepressants | 0.07-94.50 | 2.46 (2.19-2.78) | 0.06 | 3/3.00 | 1 | 2.24 (1.06-4.74); 0.04 | Highly suggestive evidence (class II) |
| Use of antipsychotics | 0.15-43.13 | 2.08 (1.70-2.54) | 0.10 | 3/3.00 | 1 | 2.45 (1.09-5.50); 0.03 | Highly suggestive evidence (class II) |
| **Other factors** | | | | | | | |
| Traumatic brain injury | 0.31-41.12 | 5.49 (4.97-6.06) | 0.05 | 6/NA | NA | 1.48 (1.06-2.07); 0.02 | Suggestive evidence (class III) |
| Fractures | 0.35-7.76 | 1.92 (1.78-2.06) | 0.04 | 3/NA | NA | 1.21 (0.93-1.57); 0.16 | Weak evidence (class IV) |
| Chronic obstructive pulmonary disease | 0.00-1240.59 | 2.09 (2.03-2.15) | 0.01 | 2/3.00 | NP | 1.31 (0.78-2.21); 0.31 | Weak evidence (class IV) |
| Asthma | 0.52-3.92 | 1.45 (1.40-1.50) | 0.02 | 2/3.00 | NP | 1.23 (0.94-1.61); 0.13 | Highly suggestive evidence (class II) |

Abbreviation: CI, confidence interval; E, the number of expected significant studies; NA, not applicable; NP, not pertinent (because the number of expected significant studies was larger than the number of observed significant studies); O, the number of observed significant studies; PI, prediction interval; SE, standard error.

# Supplementary Table 11. Sensitivity analyses of Class I-III evidence by including only cohort studies

| **Risk factors** | **Reference/Comparison/Effect size (95% CI)** | **YOD cases** | **Random-effects summary relative risk (95% CI)** | ***p* random** | **I^2^** | **Egger test *p* value** | **95% PI** | **Largest study** | **Excess significance** | | ***p* value under a 10% credibility ceiling** | **Class of evidence** |
| --- | --- | --- | --- | --- | --- | --- | --- | --- | --- | --- | --- | --- |
|  |  |  |  |  |  |  |  |  | **O/E** | ***p* value** |  |  |
| **Convincing evidence (class I)** | | | | | | | | | | | | |
| Stroke | 1. [16]/stroke (+ vs. -)/4.06 (2.80-5.88) 2. [40]/stroke (+ vs. -)/2.96 (2.02-4.35) | 991 | 3.48 (2.55-4.74) | 2.9×10^-15^ | 26% | NA | NA | 4.06 (2.80-5.88) | 2/2.00 | 1 | 0.07 | Class IV |
| **Highly suggestive evidence (class II)** | | | | | | | | | | | | |
| Alcohol use disorders | 1. [12]/Alcohol use disorders (+ vs. -)/5.49 (4.23-7.13) for male; 6.08 (4.15-8.89) for female 2. [16]/alcohol problems (+ vs. -)/3.20 (1.66-6.17) 3. [22]/alcohol abuse (+ vs. -)/3.87 (3.22-4.66) 4. [23]/alcohol-induced loss of consciousness (+ vs. -)/2.21 (1.46-3.34) 5. [40]/alcohol intoxication (+ vs. -)/4.82 (3.83-6.05) | 6,028 | 4.21 (3.29-5.38) | 2.6×10^-30^ | 74% | 0.69 | 1.92-9.22 | 3.87 (3.22-4.66) | 6/NA | NA | 0.003 | Class II |
| Diabetes | 1. [12]/diabetes (+ vs. -)/1.18 (0.86-1.62) for male; 1.34 (0.95-1.90) for female 2. [16]/diabetes (+ vs. -)/1.84 (1.25-2.69) 3. [25]/prevalent diabetes (+ vs. -)/1.73 (1.66-1.81) | 21,111 | 1.54 (1.25-1.88) | 3.3×10^-5^ | 61% | 0.29 | 0.69-3.44 | 1.73 (1.66-1.81) | 2/NA | NA | 0.03 | Class III |
| Depression | 1. [2]/depression (+ vs. -)/ 2.67 (2.32-3.07) for premenopausal women; 2.50 (2.34-2.67) for postmenopausal women 2. [16]/depression (+ vs. -)/2.59 (2.05-3.28) 3. [19]/depression (+ vs. -)/1.76 (1.71-1.82) 4. [22]/depression (+ vs. -)/1.87 (1.39-2.8) 5. [40]/depression or use of antidepressants/1.89 (1.53-2.34) | 14,352+ | 2.19 (1.79-2.69) | 3.5×10^-14^ | 96% | 0.26 | 1.07-4.47 | 1.76 (1.71-1.82) | 6/NA | NA | 0.002 | Class II |
| Mood/affective disorders | 1. [19]/mental health disorders (+ vs. -)/2.14 (2.05-2.23) | NR | NA | NA | NA | NA | NA | NA | NA | NA | NA | NA |
| Parkinson’s disease | 1. [16]/Parkinson’s disease (+ vs. -)/20.67 (11.56-36.96) | 504 | NA | NA | NA | NA | NA | NA | NA | NA | NA | NA |
| Multiple sclerosis | 1. [16]/multiple sclerosis (+ vs. -)/3.12 (1.48-6.59) 2. [17]/multiple sclerosis (+ vs. -)/4.25 (3.40-5.32) | 2,517 | 4.14 (3.34-5.13) | 1.3×10^-38^ | 0% | NA | NA | 4.25 (3.40-5.32) | 2/2.00 | 1 | 0.072 | Class II |
| Use of antidepressants | 1. [40]/depression or use of antidepressants (+ vs. -)/1.89 (1.53-2.34) | 487 | NA | NA | NA | NA | NA | NA | NA | NA | NA | NA |
| Use of antipsychotics | 1. [40]/depression or use of antidepressants (+ vs. -)/1.89 (1.53-2.34) | 487 | NA | NA | NA | NA | NA | NA | NA | NA | NA | NA |
| Asthma | 1. [16]/asthma (+ vs.-)/1.51 (1.20-1.90) | 504 | NA | NA | NA | NA | NA | NA | NA | NA | NA | NA |
| **Suggestive evidence (class III)** | | | | | | | | | | | | |
| Higher educational attainment | 1. [11]/low vs. high/3.76 (2.90-4.87) 2. [22]/high vs. low/0.88 (0.77-1.00) 3. [40]/ low vs. high/1.02 (0.83-1.25) | 5,153 | 0.62 (0.31-1.23) | 0.173 | 98% | 0.524 | 0.00-4384.70 | 0.88 (0.77-1.00) | 2/NA | NA | 0.254 | Not significant |
| Transient ischemic attack | No cohort studies | NA | NA | NA | NA | NA | NA | NA | NA | NA | NA | NA |
| Schizophrenia | 1. [16]/schizophrenia (+ vs. -)/3.77 (2.23-6.38) 2. [52]/schizophrenia (+ vs. -)/3.77 (3.29-4.33) | 9,254 | 3.77 (3.30-4.31) | 2.6×10^-85^ | 0% | NA | NA | 3.77 (3.29-4.33) | 2/NA | NA | 0.070 | Class II |
| Lower cognitive ability in young adulthood | 1. [22]/highest tertile cognitive ability vs. lowest tertile cognitive ability/0.51 (0.46-0.56) 2. [28]/continuous/1.77 (1.07-2.95) 3. [37]/low cognitive performance vs. high cognitive performance/ 3.82 (2.96-4.93) | 4,841 | 2.40 (1.47-3.95) | 5.2×10^-4^ | 92% | 0.68 | 0.01-1071.21 | 1.96 (1.79-2.17) | 3/NA | NA | 0.04 | Class III |
| Epilepsy | 1. [16]/epilepsy (+ vs. -)/2.97 (1.88-4.67) | 504 | NA | NA | NA | NA | NA | NA | NA | NA | NA | NA |
| Traumatic brain injury | 1. [22]/traumatic brain injury (+ vs. -)/5.49 (4.97-6.06) 2. [38]/traumatic brain injury (+ vs. -)/2.30 (1.50-3.60) | 4,730 | 3.65 (1.56-8.55) | 0.003 | 93% | NA | NA | 5.49 (4.97-6.06) | 2/NA | NA | 0.086 | Class IV |

Abbreviation: CI, confidence interval; E, the number of expected significant studies; NA, not applicable; NR, not reported; O, the number of observed significant studies; PI, prediction interval; YOD, young-onset dementia.

# Supplementary Table 12. Sensitivity analyses of Class I-III evidence by including only cohort studies with high methodological quality

| **Risk factors** | **Reference/Comparison/Effect size (95% CI)** | **YOD cases** | **Random-effects summary relative risk (95% CI)** | ***p* random** | **I^2^** | **Egger test *p* value** | **95% PI** | **Largest study** | **Excess significance** | | ***p* value under a 10% credibility ceiling** | **Class of evidence** |
| --- | --- | --- | --- | --- | --- | --- | --- | --- | --- | --- | --- | --- |
|  |  |  |  |  |  |  |  |  | **O/E** | ***p* value** |  |  |
| **Convincing evidence (class I)** | | | | | | | | | | | | |
| Stroke | 1. [16]/stroke (+ vs. -)/4.06 (2.80-5.88) 2. [40]/stroke (+ vs. -)/2.96 (2.02-4.35) | 991 | 3.48 (2.55-4.74) | 2.9×10^-15^ | 26% | NA | NA | 4.06 (2.80-5.88) | 2/2.00 | 1 | 0.07 | Class IV |
| **Highly suggestive evidence (class II)** | | | | | | | | | | | | |
| Alcohol use disorders | 1. [12]/Alcohol use disorders (+ vs. -)/5.49 (4.23-7.13) for male; 6.08 (4.15-8.89) for female 2. [16]/alcohol problems (+ vs. -)/3.20 (1.66-6.17) 3. [22]/alcohol abuse (+ vs. -)/3.87 (3.22-4.66) 4. [23]/alcohol-induced loss of consciousness (+ vs. -)/2.21 (1.46-3.34) 5. [40]/alcohol intoxication (+ vs. -)/4.82 (3.83-6.05) | 6,028 | 4.21 (3.29-5.38) | 2.6×10^-30^ | 74% | 0.69 | 1.92-9.22 | 3.87 (3.22-4.66) | 6/NA | NA | 0.003 | Class II |
| Diabetes | 1. [12]/diabetes (+ vs. -)/1.18 (0.86-1.62) for male; 1.34 (0.95-1.90) for female 2. [16]/diabetes (+ vs. -)/1.84 (1.25-2.69) | 1,196 | 1.40 (1.09-1.80) | 8.3×10^-3^ | 36% | 0.12 | 0.13-14.67 | 1.18 (0.86-1.62) | 1/1.33 | NP | 0.063 | Class IV |
| Depression | 1. [16]/depression (+ vs. -)/2.59 (2.05-3.28) 2. [19]/depression (+ vs. -)/1.76 (1.71-1.82) 3. [22]/depression (+ vs. -)/1.87 (1.39-2.8) 4. [40]/depression or use of antidepressants/1.89 (1.53-2.34) | 5,155+ | 1.97 (1.65-2.35) | 3.8×10^-14^ | 72% | 0.28 | 0.95-4.12 | 1.76 (1.71-1.82) | 4/NA | NA | 0.012 | Class II |
| Mood/affective disorders | 1. [19]/mental health disorders (+ vs. -)/2.14 (2.05-2.23) | NR | NA | NA | NA | NA | NA | NA | NA | NA | NA | NA |
| Parkinson’s disease | 1. [16]/Parkinson’s disease (+ vs. -)/20.67 (11.56-36.96) | 504 | NA | NA | NA | NA | NA | NA | NA | NA | NA | NA |
| Multiple sclerosis | 1. [16]/multiple sclerosis (+ vs. -)/3.12 (1.48-6.59) | 504 | NA | NA | NA | NA | NA | NA | NA | NA | NA | NA |
| Use of antidepressants | 1. [40]/depression or use of antidepressants (+ vs. -)/1.89 (1.53-2.34) | 487 | NA | NA | NA | NA | NA | NA | NA | NA | NA | NA |
| Use of antipsychotics | 1. [40]/depression or use of antidepressants (+ vs. -)/1.89 (1.53-2.34) | 487 | NA | NA | NA | NA | NA | NA | NA | NA | NA | NA |
| Asthma | 1. [16]/asthma (+ vs.-)/1.51 (1.20-1.90) | 504 | NA | NA | NA | NA | NA | NA | NA | NA | NA | NA |
| **Suggestive evidence (class III)** | | | | | | | | | | | | |
| Higher educational attainment | 1. [11]/low vs. high/3.76 (2.90-4.87) 2. [22]/high vs. low/0.88 (0.77-1.00) 3. [40]/ low vs. high/1.02 (0.83-1.25) | 5,153 | 0.62 (0.31-1.23) | 0.173 | 98% | 0.524 | 0.00-4384.70 | 0.88 (0.77-1.00) | 2/NA | NA | 0.254 | Not significant |
| Transient ischemic attack | No cohort studies | NA | NA | NA | NA | NA | NA | NA | NA | NA | NA | NA |
| Schizophrenia | 1. [16]/schizophrenia (+ vs. -)/3.77 (2.23-6.38) 2. [52]/schizophrenia (+ vs. -)/3.77 (3.29-4.33) | 9,254 | 3.77 (3.30-4.31) | 2.6×10^-85^ | 0% | NA | NA | 3.77 (3.29-4.33) | 2/NA | NA | 0.070 | Class II |
| Lower cognitive ability in young adulthood | 1. [22]/highest tertile cognitive ability vs. lowest tertile cognitive ability/0.51 (0.46-0.56) 2. [37]/low cognitive performance vs. high cognitive performance/ 3.82 (2.96-4.93) | 4,821 | 2.71 (1.41-5.20) | 0.003 | 96% | NA | NA | 0.51 (0.46-0.56) | 2/NA | NA | 0.085 | Class IV |
| Epilepsy | 1. [16]/epilepsy (+ vs. -)/2.97 (1.88-4.67) | 504 | NA | NA | NA | NA | NA | NA | NA | NA | NA | NA |
| Traumatic brain injury | 1. [22]/traumatic brain injury (+ vs. -)/5.49 (4.97-6.06) 2. [38]/traumatic brain injury (+ vs. -)/2.30 (1.50-3.60) | 4,730 | 3.65 (1.56-8.55) | 0.003 | 93% | NA | NA | 5.49 (4.97-6.06) | 2/NA | NA | 0.086 | Class IV |

Abbreviation: CI, confidence interval; E, the number of expected significant studies; NA, not applicable; NP, not pertinent (because the number of expected significant studies was larger than the number of observed significant studies); NR, not reported; O, the number of observed significant studies; PI, prediction interval; YOD, young-onset dementia.

# Supplementary Table 13. Sensitivity analyses of Class I-III evidence by including only studies with high methodological quality

| **Risk factors** | **Reference/Comparison/Effect size (95% CI)** | **YOD cases** | **Random-effects summary relative risk (95% CI)** | ***p* random** | **I^2^** | **Egger test *p* value** | **95% PI** | **Largest study** | **Excess significance** | | ***p* value under a 10% credibility ceiling** | **Class of evidence** |
| --- | --- | --- | --- | --- | --- | --- | --- | --- | --- | --- | --- | --- |
|  |  |  |  |  |  |  |  |  | **O/E** | ***p* value** |  |  |
| **Convincing evidence (class I)** | | | | | | | | | | | | |
| Stroke | 1. [16]/stroke (+ vs. -)/4.06 (2.80-5.88) 2. [30]/stroke or transient ischemic attack (+ vs. -)/13.18 (2.62-66.40) 3. [40]/stroke (+ vs. -)/2.96 (2.02-4.35) | 1,087 | 3.78 (2.47-5.80) | 1.1×10^-9^ | 49% | 0.42 | 0.05-266.27 | 4.06 (2.80-5.88) | 3/3.00 | 1 | 0.035 | Class II |
| **Highly suggestive evidence (class II)** | | | | | | | | | | | | |
| Alcohol use disorders | 1. [7]/alcohol-related mental disorders (+ vs. -)/2.12 (1.62-2.79) 2. [12]/Alcohol use disorders (+ vs. -)/5.49 (4.23-7.13) for male; 6.08 (4.15-8.89) for female 3. [16]/alcohol problems (+ vs. -)/3.20 (1.66-6.17) 4. [22]/alcohol abuse (+ vs. -)/3.87 (3.22-4.66) 5. [23]/alcohol-induced loss of consciousness (+ vs. -)/2.21 (1.46-3.34) 6. [40]/alcohol intoxication (+ vs. -)/4.82 (3.83-6.05) | 7,773 | 3.74 (2.79-5.03) | 2.2×10^-18^ | 86% | 0.70 | 1.38-10.17 | 3.87 (3.22-4.66) | 7/NA | NA | 0.001 | Class II |
| Diabetes | 1. [7]/T2DM (+ vs. -)/1.20 (0.96-1.51) 2. [12]/diabetes (+ vs. -)/1.18 (0.86-1.62) for male; 1.34 (0.95-1.90) for female 3. [16]/diabetes (+ vs. -)/1.84 (1.25-2.69) 4. [34]/diabetes (+ vs. -)/2.35 (0.57-9.66) | 3,034 | 1.32 (1.12-1.57) | 1.02×10^-3^ | 15% | 0.24 | 0.92-1.90 | 1.20 (0.96-1.51) | 1/2.64 | NP | 0.017 | Class IV |
| Depression | 1. [7]/depressive episode (+ vs. -)/3.48 (2.54-4.77) 2. [16]/depression (+ vs. -)/2.59 (2.05-3.28) 3. [56]/depression (+ vs. -)/1.35 (1.12-1.63) 4. [19]/depression (+ vs. -)/1.76 (1.71-1.82) 5. [22]/depression (+ vs. -)/1.87 (1.39-2.8) 6. [30]/depression (+ vs. -)/3.36 (1.46-7.73) 7. [40]/depression or use of antidepressants/1.89 (1.53-2.34) | 7,969+ | 2.06 (1.69-2.51) | 1.1×10^-12^ | 85% | 0.27 | 1.09-3.87 | 1.76 (1.71-1.82) | 7/NA | NA | 0.003 | Class II |
| Mood/affective disorders | 1. [7]/mood/affective disorders (+ vs. -)/3.14 (2.48-3.98) 2. [19]/mental health disorders (+ vs. -)/2.14 (2.05-2.23) | 1,745+ | 2.54 (1.75-3.70) | 9.8×10^-7^ | 90% | NA | NA | 2.14 (2.05-2.23) | 2/NA | NA | 0.076 | Class II |
| Parkinson’s disease | 1. [16]/Parkinson’s disease (+ vs. -)/20.67 (11.56-36.96) | 504 | NA | NA | NA | NA | NA | NA | NA | NA | NA | NA |
| Multiple sclerosis | 1. [16]/multiple sclerosis (+ vs. -)/3.12 (1.48-6.59) | 504 | NA | NA | NA | NA | NA | NA | NA | NA | NA | NA |
| Use of antidepressants | 1. [40]/depression or use of antidepressants (+ vs. -)/1.89 (1.53-2.34) | 487 | NA | NA | NA | NA | NA | NA | NA | NA | NA | NA |
| Use of antipsychotics | 1. [40]/depression or use of antidepressants (+ vs. -)/1.89 (1.53-2.34) | 487 | NA | NA | NA | NA | NA | NA | NA | NA | NA | NA |
| Asthma | 1. [7]/asthma (+ vs.-)/1.09 (0.77-1.54) 2. [16]/asthma (+ vs.-)/1.51 (1.20-1.90) | 2,249 | 1.32 (0.96-1.81) | 0.086 | 58% | NA | NA | 1.51 (1.20-1.90) | 1/2.00 | NP | 0.296 | Not significant |
| **Suggestive evidence (class III)** | | | | | | | | | | | | |
| Higher educational attainment | 1. [11]/low vs. high/3.76 (2.90-4.87) 2. [22]/high vs. low/0.88 (0.77-1.00) 3. [30]/continuous/0.87 (0.79-0.95) 4. [34]/high vs. low/0.82 (0.75-0.89) 5. [40]/ low vs. high/1.02 (0.83-1.25) 6. [45]/low vs. high/1.70 (1.10-2.70) | 5,549 | 0.70 (0.54-0.89) | 0.004 | 94% | 0.31 | 0.29-1.69 | 0.82 (0.75-0.89) | 5/NA | NA | 0.030 | Class IV |
| Transient ischemic attack | 1. [7]/Transient ischemic attack (+ vs. -)/1.56 (1.05-2.31) 2. [30]/stroke or transient ischemic attack (+ vs. -)/13.18 (2.62-66.40) | 1,841 | 3.90 (0.49-30.95) | 0.197 | 84% | NA | NA | 1.56 (1.05-2.31) | 2/1.49 | 1 | 0.273 | Not significant |
| Schizophrenia | 1. [7]/schizophrenia, schizotypal and delusional disorders (+ vs. -)/2.28 (1.46-3.57) 2. [16]/schizophrenia (+ vs. -)/3.77 (2.23-6.38) 3. [52]/schizophrenia (+ vs. -)/3.77 (3.29-4.33) | 10,999 | 3.31 (2.43-4.49) | 1.9×10^-14^ | 55% | 0.550 | 0.13-86.60 | 3.77 (3.29-4.33) | 3/NA | NA | 0.031 | Class II |
| Lower cognitive ability in young adulthood | 1. [22]/highest tertile cognitive ability vs. lowest tertile cognitive ability/0.51 (0.46-0.56) 2. [37]/low cognitive performance vs. high cognitive performance/ 3.82 (2.96-4.93) | 4,821 | 2.71 (1.41-5.20) | 0.003 | 96% | NA | NA | 0.51 (0.46-0.56) | 2/NA | NA | 0.085 | Class IV |
| Epilepsy | 1. [16]/epilepsy (+ vs. -)/2.97 (1.88-4.67) 2. [56]/epilepsy (+ vs. -)/4.80 (3.30-6.97) | 1,477 | 3.85 (2.41-6.15) | 1.8×10^-8^ | 61% | NA | NA | 4.80 (3.30-6.97) | 2/2.00 | 1 | 0.075 | Class II |
| Traumatic brain injury | 1. [56]/traumatic brain injury (+ vs. -)/3.05 (2.42-3.83) 2. [22]/traumatic brain injury (+ vs. -)/5.49 (4.97-6.06) 3. [38]/traumatic brain injury (+ vs. -)/2.30 (1.50-3.60) 4. [45]/head trauma (+ vs. -)/ 10.00 (1.00-96.80) | 5,901 | 3.64 (2.19-6.06) | 6.8×10^-7^ | 91% | 0.429 | 0.41-32.62 | 5.49 (4.97-6.06) | 4/NA | NA | 0.017 | Class II |

Abbreviation: CI, confidence interval; E, the number of expected significant studies; NA, not applicable; NP, not pertinent (because the number of expected significant studies was larger than the number of observed significant studies); O, the number of observed significant studies; PI, prediction interval; YOD, young-onset dementia.

# Supplementary Table 14. Sensitivity analyses of Class I-III evidence with primary studies using all-cause dementia as an outcome

| **Risk factors** | **Reference/Comparison/Effect size (95% CI)** | **YOD cases** | **Random-effects summary relative risk (95% CI)** | ***p* random** | **I^2^** | **Egger test *p* value** | **95% PI** | **Largest study** | **Excess significance** | | ***p* value under a 10% credibility ceiling** | **Class of evidence** |
| --- | --- | --- | --- | --- | --- | --- | --- | --- | --- | --- | --- | --- |
|  |  |  |  |  |  |  |  |  | **O/E** | ***p* value** |  |  |
| **Convincing evidence (class I)** | | | | | | | | | | | | |
| Stroke | 1. [4]/stroke (+ vs. -)/4.01 (0.41-39.19) 2. [16]/stroke (+ vs. -)/4.06 (2.80-5.88) 3. [30]/stroke or transient ischemic attack (+ vs. -)/13.18 (2.62-66.40) 4. [53]/stroke or transient ischemic attack (+ vs. -)/3.10 (2.40-4.00) 5. [40]/stroke (+ vs. -)/2.96 (2.02-4.35) | 2,206 | 3.38 (2.74-4.17) | 7.1×10^-30^ | 12% | 0.25 | 2.17-5.26 | 3.10 (2.40-4.00) | 4/4.90 | NP | 0.006 | Class I |
| **Highly suggestive evidence (class II)** | | | | | | | | | | | | |
| Alcohol use disorders | 1. [12]/Alcohol use disorders (+ vs. -)/5.49 (4.23-7.13) for male; 6.08 (4.15-8.89) for female 2. [16]/alcohol problems (+ vs. -)/3.20 (1.66-6.17) 3. [22]/alcohol abuse (+ vs. -)/3.87 (3.22-4.66) 4. [23]/alcohol-induced loss of consciousness (+ vs. -)/2.21 (1.46-3.34) 5. [40]/alcohol intoxication (+ vs. -)/4.82 (3.83-6.05) 6. [54]/alcohol disorders (+ vs. -)/7.52 (6.29-8.99) | 6,028+ | 4.59 (3.45-6.10) | 1.2×10^-25^ | 87% | 0.35 | 1.75-12.03 | 7.52 (6.29-8.99) | 7/NA | NA | 0.001 | Class II |
| Diabetes | 1. [4]/diabetes (+ vs. -)/2.72 (0.67-10.97) 2. [12]/diabetes (+ vs. -)/1.18 (0.86-1.62) for male; 1.34 (0.95-1.90) for female 3. [16]/diabetes (+ vs. -)/1.84 (1.25-2.69) 4. [49]/diabetes (+ vs. -)/1.92 (1.87-1.98) 5. [51]/diabetes (+ vs. -)/1.36 (1.24-1.49) 6. [25]/prevalent diabetes (+ vs. -)/1.73 (1.66-1.81) 7. [53]/diabetes (+ vs. -)/2.00 (1.70-2.50) | 51,542 | 1.65 (1.47-1.85) | 6.6×10^-17^ | 90% | 0.27 | 1.17-2.32 | 1.92 (1.87-1.98) | 5/NA | NA | 0.002 | Class II |
| Depression | 1. [2]/depression (+ vs. -)/ 2.67 (2.32-3.07) for premenopausal women; 2.50 (2.34-2.67) for postmenopausal women 2. [16]/depression (+ vs. -)/2.59 (2.05-3.28) 3. [19]/depression (+ vs. -)/1.76 (1.71-1.82) 4. [22]/depression (+ vs. -)/1.87 (1.39-2.8) 5. [30]/depression (+ vs. -)/3.36 (1.46-7.73) 6. [40]/depression or use of antidepressants/1.89 (1.53-2.34) | 14,448+ | 2.23 (1.83-2.72) | 2.3×10^-15^ | 95% | 0.22 | 1.16-4.31 | 1.76 (1.71-1.82) | 7/NA | NA | 0.001 | Class II |
| Mood/affective disorders | 1. [49]/mental health conditions (+ vs. -)/3.26 (3.18-3.34) 2. [19]/mental health disorders (+ vs. -)/2.14 (2.05-2.23) | 28,238+ | 2.64 (1.75-3.99) | 3.9×10^-6^ | 100% | NA | NA | 3.26 (3.18-3.34) | 2/NA | NA | 0.077 | Class III |
| Parkinson’s disease | 1. [16]/Parkinson’s disease (+ vs. -)/20.67 (11.56-36.96) 2. [49]/Parkinsonism (+ vs. -)/12.53 (11.84-13.27) 3. [51]/Parkinson’s disease (+ vs. -)/26.21 (23.15-29.68) 4. [53]/Parkinson’s disease (+ vs. -)/8.70 (4.40-16.90) | 30,877 | 16.08 (9.42-27.43) | 2.3×10^-24^ | 97% | 0.65 | 1.37-188.61 | 12.53 (11.84-13.27) | 4/NA | NA | 0.01 | Class II |
| Multiple sclerosis | 1. [16]/multiple sclerosis (+ vs. -)/3.12 (1.48-6.59) 2. [49]/multiple sclerosis (+ vs. -)/10.27 (9.55-11.03) 3. [51]/multiple sclerosis (+ vs. -)/8.99 (7.35-10.97) 4. [53]/multiple sclerosis (+ vs. -)/1.80 (0.90-3.40) | 30,877 | 5.71 (3.60-9.04) | 1.2×10^-13^ | 92% | 0.06 | 0.74-43.72 | 10.27 (9.55-11.03) | 3/NA | NA | 0.03 | Class II |
| Use of antidepressants | 1. [40]/depression or use of antidepressants (+ vs. -)/1.89 (1.53-2.34) | 487 | NA | NA | NA | NA | NA | NA | NA | NA | NA | NA |
| Use of antipsychotics | 1. [40]/depression or use of antidepressants (+ vs. -)/1.89 (1.53-2.34) | 487 | NA | NA | NA | NA | NA | NA | NA | NA | NA | NA |
| Asthma | 1. [16]/asthma (+ vs.-)/1.51 (1.20-1.90) 2. [49]/asthma (+ vs.-)/1.45 (1.40-1.50) | 25,742 | 1.45 (1.40-1.50) | 1.4×10^-101^ | 0% | NA | NA | 1.45 (1.40-1.50) | 2/2.00 | 1 | 0.070 | Class II |
| **Suggestive evidence (class III)** | | | | | | | | | | | | |
| Higher educational attainment | 1. [11]/low vs. high/3.76 (2.90-4.87) 2. [22]/high vs. low/0.88 (0.77-1.00) 3. [27]/high vs. low/0.43 (0.20-0.94) 4. [30]/continuous/0.87 (0.79-0.95) 5. [40]/ low vs. high/1.02 (0.83-1.25) | 5,307 | 0.64 (0.44-0.94) | 0.023 | 96% | 0.37 | 0.15-2.68 | 0.87 (0.79-0.95) | 4/NA | NA | 0.077 | Class IV |
| Transient ischemic attack | 1. [30]/stroke or transient ischemic attack (+ vs. -)/13.18 (2.62-66.40) 2. [53]/stroke or transient ischemic attack (+ vs. -)/3.10 (2.40-4.00) 3. [54]/transient ischemic attack (+ vs. -)/1.92 (1.22-3.04) | 1,157 | 2.96 (1.67-5.24) | 2.0×10^-4^ | 70% | 0.770 | 0.01-1511.05 | 3.10 (2.40-4.00) | 3/NA | NA | 0.047 | Class III |
| Schizophrenia | 1. [49]/schizophrenia (+ vs. -)/16.71 (16.03-17.41) 2. [16]/schizophrenia (+ vs. -)/3.77 (2.23-6.38) 3. [51]/schizophrenia and psychotic disorders (+ vs. -)/7.12 (6.36-7.97) 4. [52]/schizophrenia (+ vs. -)/3.77 (3.29-4.33) | 38,566 | 6.51 (2.93-14.46) | 4.3×10^-6^ | 100% | 0.185 | 0.13-314.12 | 16.71 (16.03-17.41) | 4/NA | NA | 0.014 | Class III |
| Lower cognitive ability in young adulthood | 1. [22]/highest tertile cognitive ability vs. lowest tertile cognitive ability/0.51 (0.46-0.56) 2. [28]/continuous/1.77 (1.07-2.95) 3. [37]/low cognitive performance vs. high cognitive performance/ 3.82 (2.96-4.93) | 4,841 | 2.40 (1.47-3.95) | 5.2×10^-4^ | 92% | 0.68 | 0.01-1071.21 | 1.96 (1.79-2.17) | 3/NA | NA | 0.04 | Class III |
| Epilepsy | 1. [16]/epilepsy (+ vs. -)/2.97 (1.88-4.67) 2. [49]/epilepsy (+ vs. -)/11.35 (10.91-11.81) 3. [51]/epilepsy (+ vs. -)/16.06 (14.43-17.89) 4. [54]/epilepsy (+ vs. -)/10.17 (7.93-13.05) | 29,816+ | 9.51 (6.88-13.15) | 3.4×10^-42^ | 96% | 0.765 | 2.12-42.74 | 11.35 (10.91-11.81) | 4/NA | NA | 0.018 | Class II |
| Traumatic brain injury | 1. [51]/traumatic brain injury (+ vs. -)/14.02 (12.35-15.92) 2. [22]/traumatic brain injury (+ vs. -)/5.49 (4.97-6.06) 3. [27]/head trauma (+ vs. -)/1.54 (0.47-4.99) 4. [38]/traumatic brain injury (+ vs. -)/2.30 (1.50-3.60) | 8,862 | 4.63 (2.21-9.70) | 4.9×10^-5^ | 98% | 0.711 | 0.15-143.54 | 5.49 (4.97-6.06) | 3/NA | NA | 0.051 | Class III |

Abbreviation: CI, confidence interval; E, the number of expected significant studies; NA, not applicable; NP, not pertinent (because the number of expected significant studies was larger than the number of observed significant studies); O, the number of observed significant studies; PI, prediction interval; YOD, young-onset dementia.

# Supplementary Table 15. Sensitivity analyses of Class I-III evidence with primary studies defining YOD as onset of dementia symptoms before age 65

| **Risk factors** | **Reference/Comparison/Effect size (95% CI)** | **YOD cases** | **Random-effects summary relative risk (95% CI)** | ***p* random** | **I^2^** | **Egger test *p* value** | **95% PI** | **Largest study** | **Excess significance** | | ***p* value under a 10% credibility ceiling** | **Class of evidence** |
| --- | --- | --- | --- | --- | --- | --- | --- | --- | --- | --- | --- | --- |
|  |  |  |  |  |  |  |  |  | **O/E** | ***p* value** |  |  |
| **Convincing evidence (class I)** | | | | | | | | | | | | |
| Stroke | 1. [4]/stroke (+ vs. -)/4.01 (0.41-39.19) 2. [30]/stroke or transient ischemic attack (+ vs. -)/13.18 (2.62-66.40) | 154 | 8.85 (2.37-33.09) | 0.0012 | 0% | NA | NA | 13.18 (2.62-66.40) | 2/2.00 | 1 | 0.09 | Class IV |
| **Highly suggestive evidence (class II)** | | | | | | | | | | | | |
| Alcohol use disorders | No studies meet the age cut-off criterion | NA | NA | NA | NA | NA | NA | NA | NA | NA | NA | NA |
| Diabetes | 1. [4]/diabetes (+ vs. -)/2.72 (0.67-10.97) | 58 | NA | NA | NA | NA | NA | NA | NA | NA | NA | NA |
| Depression | 1. [30]/depression (+ vs. -)/3.36 (1.46-7.73) | 96 | NA | NA | NA | NA | NA | NA | NA | NA | NA | NA |
| Mood/affective disorders | No studies meet the age cut-off criterion | NA | NA | NA | NA | NA | NA | NA | NA | NA | NA | NA |
| Parkinson’s disease | No studies meet the age cut-off criterion | NA | NA | NA | NA | NA | NA | NA | NA | NA | NA | NA |
| Multiple sclerosis | No studies meet the age cut-off criterion | NA | NA | NA | NA | NA | NA | NA | NA | NA | NA | NA |
| Use of antidepressants | No studies meet the age cut-off criterion | NA | NA | NA | NA | NA | NA | NA | NA | NA | NA | NA |
| Use of antipsychotics | No studies meet the age cut-off criterion | NA | NA | NA | NA | NA | NA | NA | NA | NA | NA | NA |
| Asthma | No studies meet the age cut-off criterion | NA | NA | NA | NA | NA | NA | NA | NA | NA | NA | NA |
| **Suggestive evidence (class III)** | | | | | | | | | | | | |
| Higher educational attainment | 1. [27]/high vs. low/0.43 (0.20-0.94) 2. [30]/continuous/0.87 (0.79-0.95) | 154 | 0.68 (0.35-1.32) | 0.254 | 68% | NA | NA | 0.87 (0.79-0.95) | 2/0.16 | 6.6×10^-3^ | 0.301 | Not significant |
| Transient ischemic attack | 1. [30]/stroke or transient ischemic attack (+ vs. -)/13.18 (2.62-66.40) | 96 | NA | NA | NA | NA | NA | NA | NA | NA | NA | NA |
| Schizophrenia | No studies meet the age cut-off criterion | NA | NA | NA | NA | NA | NA | NA | NA | NA | NA | NA |
| Lower cognitive ability in young adulthood | No studies meet the age cut-off criterion | 657 | NA | NA | NA | NA | NA | NA | NA | NA | NA | NA |
| Epilepsy | No studies meet the age cut-off criterion | NA | NA | NA | NA | NA | NA | NA | NA | NA | NA | NA |
| Traumatic brain injury | 1. [27]/head trauma (+ vs. -)/1.54 (0.47-4.99) 2. [36]/traumatic brain injury (+ vs. -)/1.28 (1.00-1.57) | 1,485 | 1.29 (1.03-1.61) | 0.025 | 0% | NA | NA | 1.28 (1.00-1.57) | 1/1.06 | NP | 0.15 | Class IV |

Abbreviation: CI, confidence interval; E, the number of expected significant studies; NA, not applicable; NP, not pertinent (because the number of expected significant studies was larger than the number of observed significant studies); O, the number of observed significant studies; PI, prediction interval; YOD, young-onset dementia.

# Supplementary Table 16. Sensitivity analyses of Class I-III evidence with primary studies adjusting for multiple key covariates (age, sex, and educational level/socioeconomic status, and apolipoprotein E status/family history of dementia)

| **Risk factors** | **Reference/Comparison/Effect size (95% CI)** | **YOD cases** | **Random-effects summary relative risk (95% CI)** | ***p* random** | **I^2^** | **Egger test *p* value** | **95% PI** | **Largest study** | **Excess significance** | | ***p* value under a 10% credibility ceiling** | **Class of evidence** |
| --- | --- | --- | --- | --- | --- | --- | --- | --- | --- | --- | --- | --- |
|  |  |  |  |  |  |  |  |  | **O/E** | ***p* value** |  |  |
| **Convincing evidence (class I)** | | | | | | | | | | | | |
| Stroke | 1. [30]/stroke or transient ischemic attack (+ vs. -)/13.18 (2.62-66.40) 2. [40]/stroke (+ vs. -)/2.96 (2.02-4.35) | 583 | 5.04 (1.24-20.47) | 0.02 | 68% | NA | NA | 2.96 (2.02-4.35) | 2/2.00 | 1 | 0.09 | Class IV |
| **Highly suggestive evidence (class II)** | | | | | | | | | | | | |
| Alcohol use disorders | 1. [40]/alcohol intoxication (+ vs. -)/4.82 (3.83-6.05) | 487 | NA | NA | NA | NA | NA | NA | NA | NA | NA | NA |
| Diabetes | 1. [34]/diabetes (+ vs. -)/2.35 (0.57-9.66) | 102 | NA | NA | NA | NA | NA | NA | NA | NA | NA | NA |
| Depression | 1. [30]/depression (+ vs. -)/3.36 (1.46-7.73) 2. [40]/depression or use of antidepressants/1.89 (1.53-2.34) | 583 | 2.18 (1.34-3.53) | 0.002 | 42% | NA | NA | 1.89 (1.53-2.34) | 2/1.79 | 1 | 0.084 | Class IV |
| Mood/affective disorders | No studies meet the adjustment criterion | NA | NA | NA | NA | NA | NA | NA | NA | NA | NA | NA |
| Parkinson’s disease | No studies meet the adjustment criterion | NA | NA | NA | NA | NA | NA | NA | NA | NA | NA | NA |
| Multiple sclerosis | No studies meet the adjustment criterion | NA | NA | NA | NA | NA | NA | NA | NA | NA | NA | NA |
| Use of antidepressants | 1. [40]/depression or use of antidepressants (+ vs. -)/1.89 (1.53-2.34) | 487 | NA | NA | NA | NA | NA | NA | NA | NA | NA | NA |
| Use of antipsychotics | 1. [40]/depression or use of antidepressants (+ vs. -)/1.89 (1.53-2.34) | 487 | NA | NA | NA | NA | NA | NA | NA | NA | NA | NA |
| Asthma | No studies meet the adjustment criterion | NA | NA | NA | NA | NA | NA | NA | NA | NA | NA | NA |
| **Suggestive evidence (class III)** | | | | | | | | | | | | |
| Higher educational attainment | 1. [30]/continuous/0.87 (0.79-0.95) 2. [34]/high vs. low/0.82 (0.75-0.89) 3. [40]/ low vs. high/1.02 (0.83-1.25) 4. [45]/low vs. high/1.70 (1.10-2.70) | 883 | 0.85 (0.78-0.94) | 0.001 | 44% | 0.80 | 0.61-1.19 | 0.82 (0.75-0.89) | 3/1.17 | 0.08 | 0.095 | Class IV |
| Transient ischemic attack | 1. [30]/stroke or transient ischemic attack (+ vs. -)/13.18 (2.62-66.40) | 96 | NA | NA | NA | NA | NA | NA | NA | NA | NA | NA |
| Schizophrenia | No studies meet the adjustment criterion | NA | NA | NA | NA | NA | NA | NA | NA | NA | NA | NA |
| Lower cognitive ability in young adulthood | No studies meet the adjustment criterion | NA | NA | NA | NA | NA | NA | NA | NA | NA | NA | NA |
| Epilepsy | No studies meet the adjustment criterion | NA | NA | NA | NA | NA | NA | NA | NA | NA | NA | NA |
| Traumatic brain injury | 1. [38]/traumatic brain injury (+ vs. -)/2.30 (1.50-3.60) 2. [45]/head trauma (+ vs. -)/ 10.00 (1.00-96.80) | 764 | 3.07 (0.98-9.65) | 0.055 | 35% | NA | NA | 2.30 (1.50-3.60) | 2/2.00 | 1 | 0.101 | Not significant |

Abbreviation: CI, confidence interval; E, the number of expected significant studies; NA, not applicable; O, the number of observed significant studies; PI, prediction interval; YOD, young-onset dementia.

# Supplementary Table 17. Summary of sensitivity analyses

| **Risk factors (no. of studies in primary analysis)** | **Class of evidence based on a series of sensitivity analyses (no. of studies)** | | | | | |
| --- | --- | --- | --- | --- | --- | --- |
|  | **Including only cohort studies** | **Including only cohort studies with high methodological quality** | **Including only studies with high methodological quality** | **Including only studies using all-cause dementia as an outcome** | **Including only studies defining YOD as the onset of dementia symptoms before the age of 65** | **Including only studies adjusting for multiple key covariates** |
| **Convincing evidence based on primary analysis (class I)** | | | | | | |
| Stroke (5) | Class IV (2) | Class IV (2) | Class II (3) | Class I (5) | Class IV (2) | Class IV (2) |
| **Highly suggestive evidence based on primary analysis (class II)** | | | | | | |
| Alcohol use disorders (7) | Class II (5) | Class II (5) | Class II (6) | Class II (6) | NA (0) | NA (1) |
| Diabetes (11) | Class III (3) | Class IV (2) | Class IV (4) | Class II (7) | NA (1) | NA (1) |
| Depression (8) | Class II (5) | Class II (4) | Class II (7) | Class II (6) | NA (1) | Class IV (2) |
| Mood/affective disorders (3) | NA (1) | NA (1) | Class II (2) | Class III (2) | NA (0) | NA (0) |
| Parkinson’s disease (4) | NA (1) | NA (1) | NA (1) | Class II (4) | NA (0) | NA (0) |
| Multiple sclerosis (5) | Class II (2) | NA (1) | NA (1) | Class II (4) | NA (0) | NA (0) |
| Use of antidepressants (3) | NA (1) | NA (1) | NA (1) | NA (1) | NA (0) | NA (1) |
| Use of antipsychotics (3) | NA (1) | NA (1) | NA (1) | NA (1) | NA (0) | NA (1) |
| Asthma (3) | NA (1) | NA (1) | Not significant (2) | Class II (2) | NA (0) | NA (0) |
| **Suggestive evidence based on primary analysis (class III)** | | | | | | |
| Higher educational attainment (8) | Not significant (3) | Not significant (3) | Class IV (6) | Class IV (5) | Not significant (2) | Class IV (4) |
| Transient ischemic attack (4) | NA (0) | NA (0) | Not significant (2) | Class III (3) | NA (1) | NA (1) |
| Schizophrenia (5) | Class II (2) | Class II (2) | Class II (3) | Class III (4) | NA (0) | NA (0) |
| Lower cognitive ability in young adulthood (3) | Class III (3) | Class IV (2) | Class IV (2) | Class III (3) | NA (0) | NA (0) |
| Epilepsy (6) | NA (1) | NA (1) | Class II (2) | Class II (4) | NA (0) | NA (0) |
| Traumatic brain injury (7) | Class IV (2) | Class IV (2) | Class II (4) | Class III (4) | Class IV (2) | Not significant (2) |

Abbreviation: NA, not applicable; YOD, young-onset dementia.

# Supplementary Table 18. Sensitivity analyses using various credibility ceilings and assumed true effect sizes in meta-analyses of prior stroke and risk of YOD meeting convincing evidence criteria

| **Risk factors** | **Excess significance bias** | | | | | | | | **Random-effects summary estimate with *p* value** | | |
| --- | --- | --- | --- | --- | --- | --- | --- | --- | --- | --- | --- |
|  | **Primary analysis (assuming the true effect size equals that of the largest study)** | | **Assuming the true effect size equals that of the study with the largest sample size** | | **Assuming the true effect size equals that of the study with the largest number of YOD cases** | | **Assuming the true effect size equals the summary random effects estimate** | | **Primary analysis (10% credibility ceiling)** | **Under a 15% credibility ceiling** | **Under a 20% credibility ceiling** |
|  | **O/E** | ***p* value** | **O/E** | ***p* value** | **O/E** | ***p* value** | **O/E** | ***p* value** | **ES, *p* value** | **ES, *p* value** | **ES, *p* value** |
| Stroke | 4/4.90 | NP | 4/4.90 | NP | 4/4.90 | NP | 4/4.94 | NP | 3.63 (1.44-9.14); 0.006 | 3.64 (1.18-11.24); 0.025 | 3.64 (0.91-14.60); 0.069 |

Abbreviation: E, the number of expected significant studies; ES, estimate size; NP, not pertinent (because the number of expected significant studies was larger than the number of observed significant studies); O, the number of observed significant studies; YOD, young-onset dementia.


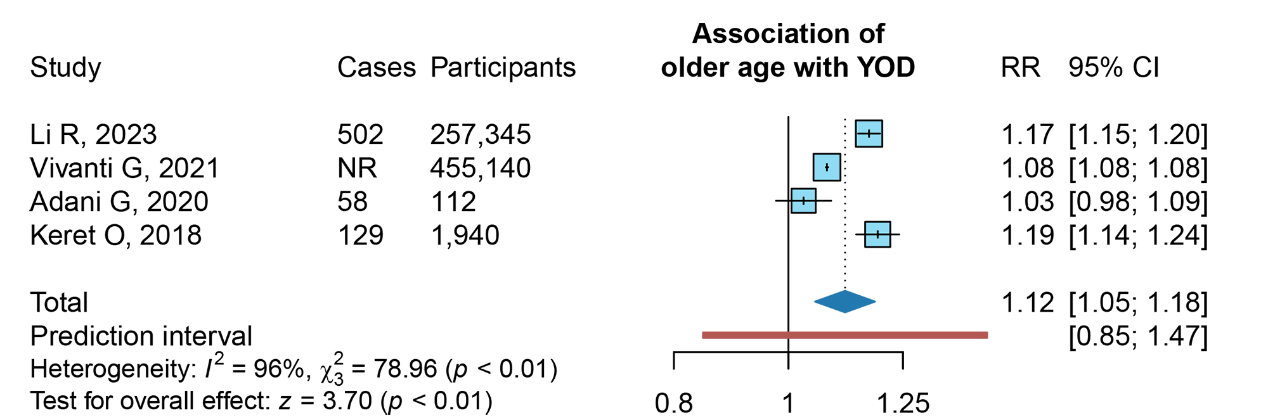


# Supplementary Figure 1. Forest plot of the association between older age and risk of young-onset dementia


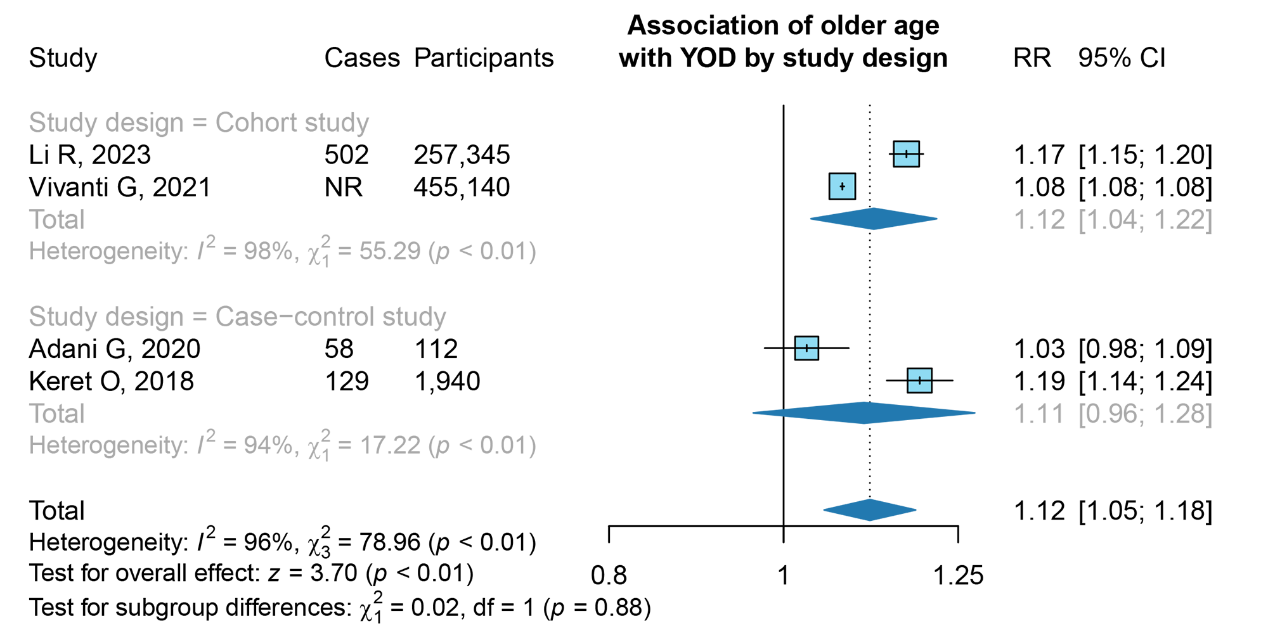


# Supplementary Figure 2. Subgroup analysis of older age and young-onset dementia risk by study design


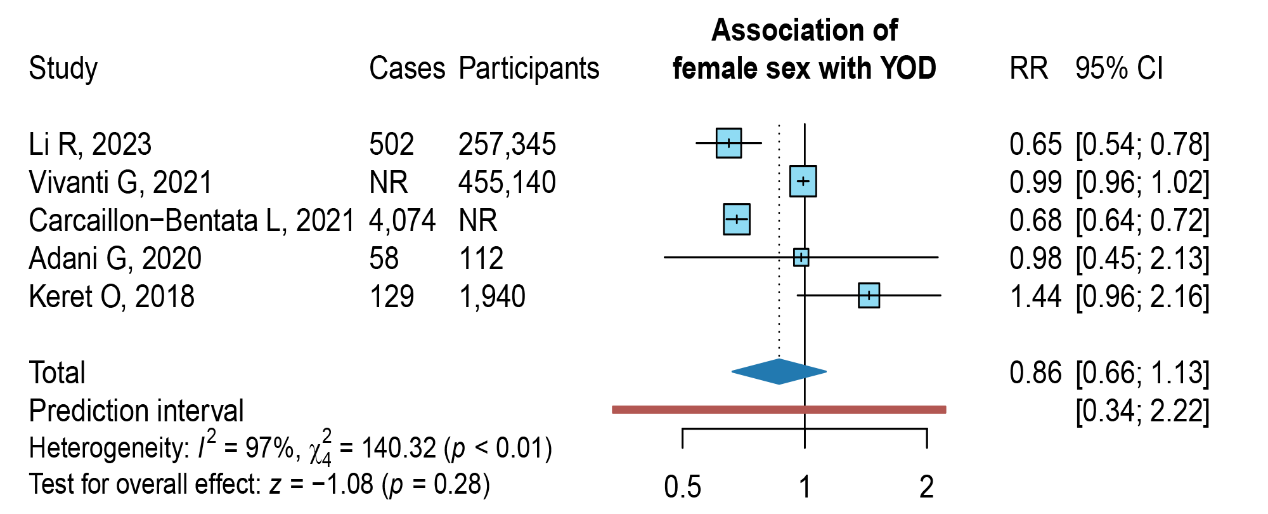


# Supplementary Figure 3. Forest plot of the association between female and risk of young-onset dementia


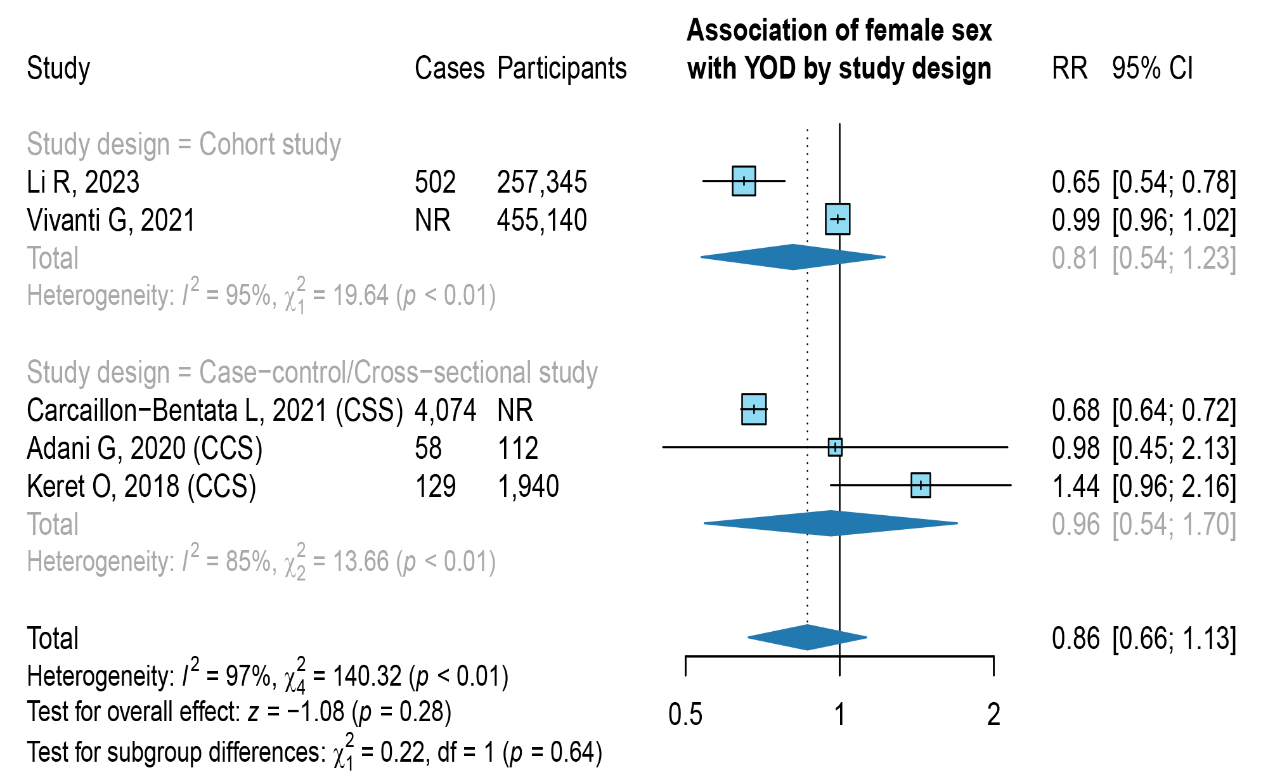


# Supplementary Figure 4. Subgroup analysis of female and young-onset dementia risk by study design


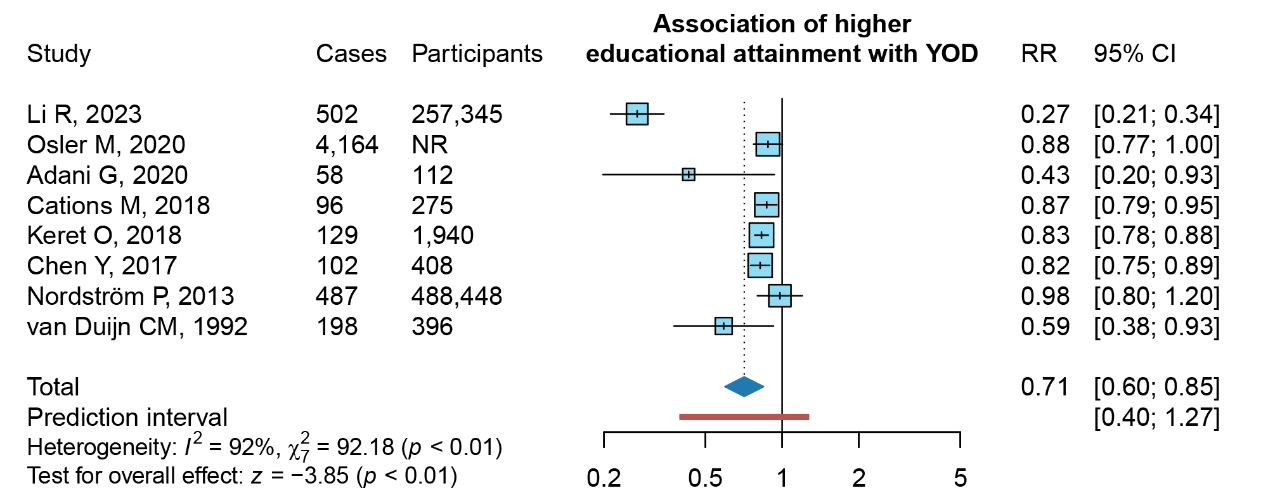


# Supplementary Figure 5. Forest plot of the association between higher educational attainment and risk of young-onset dementia


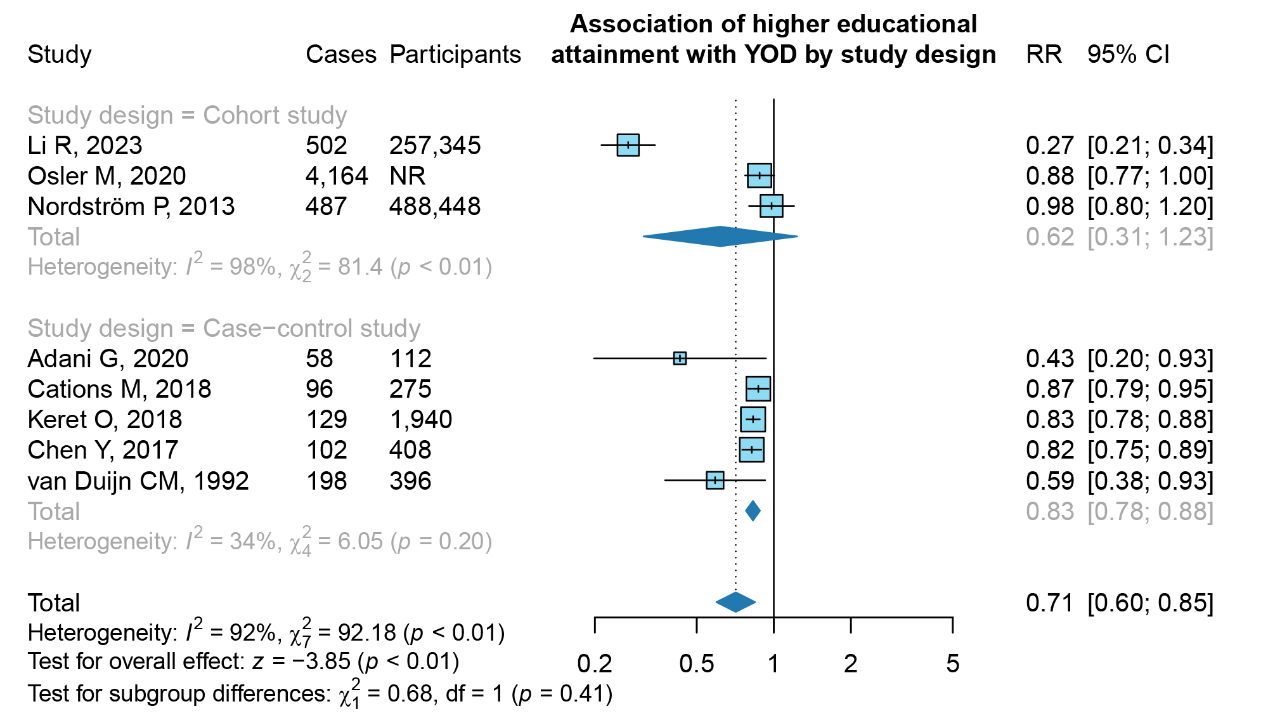


# Supplementary Figure 6. Subgroup analysis of higher educational attainment and young-onset dementia risk by study design


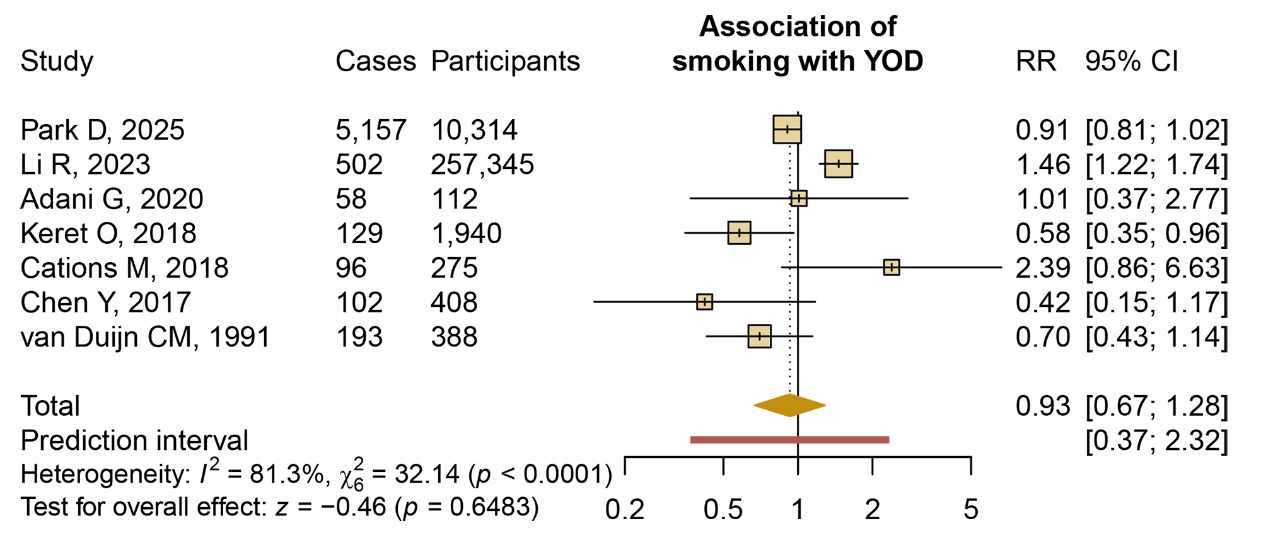


# Supplementary Figure 7. Forest plot of the association between smoking and risk of young-onset dementia


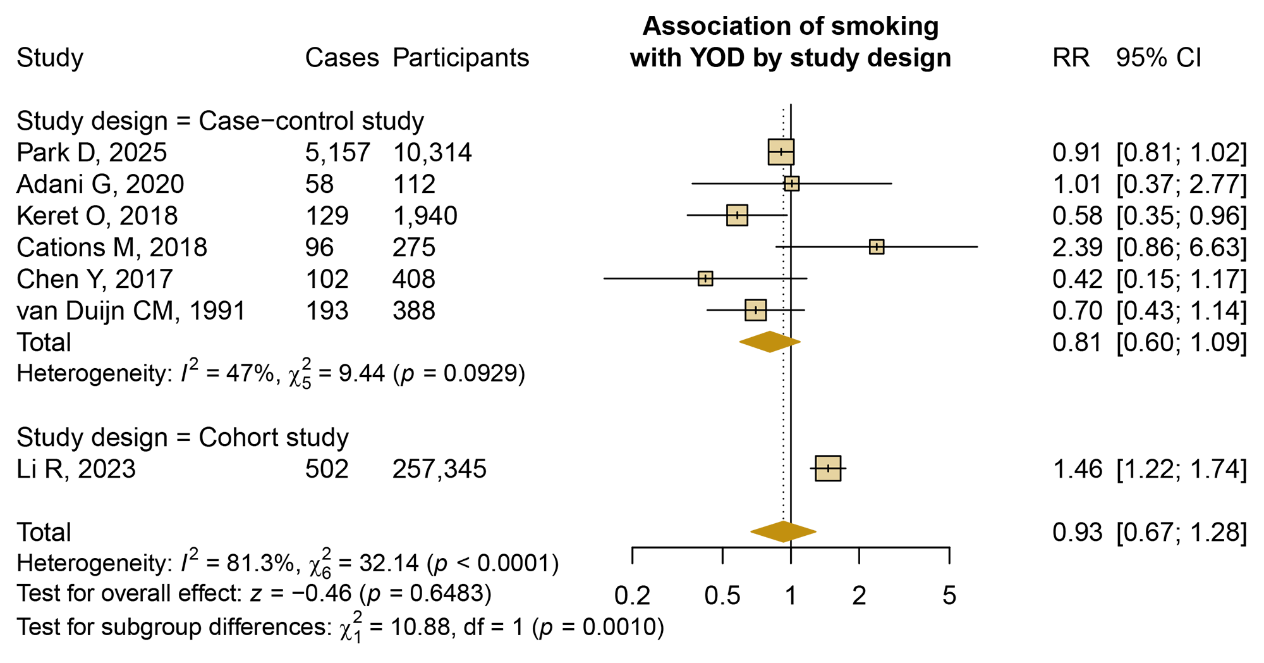


# Supplementary Figure 8. Subgroup analysis of smoking and young-onset dementia risk by study design


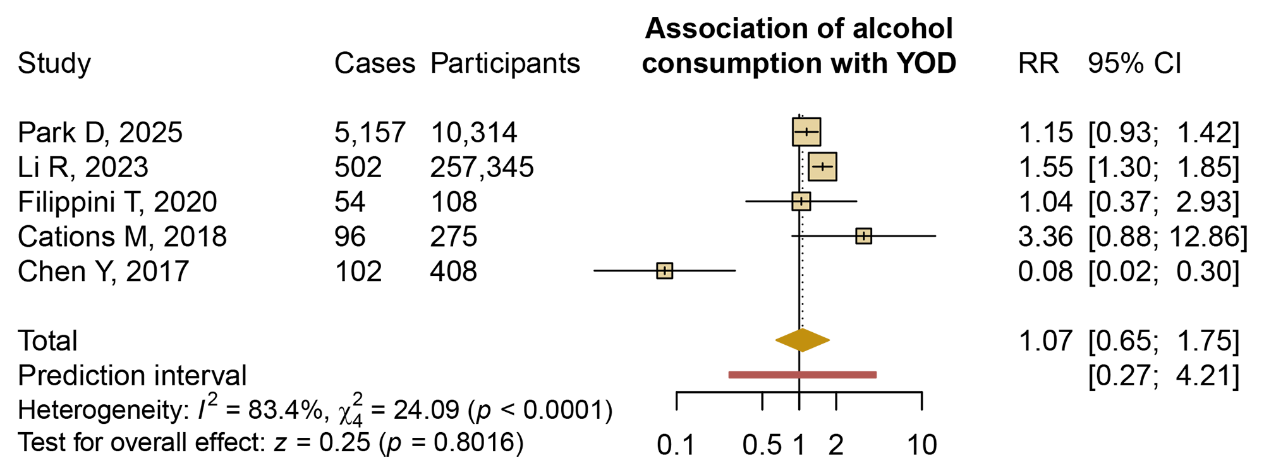


# Supplementary Figure 9. Forest plot of the association between alcohol consumption and risk of young-onset dementia


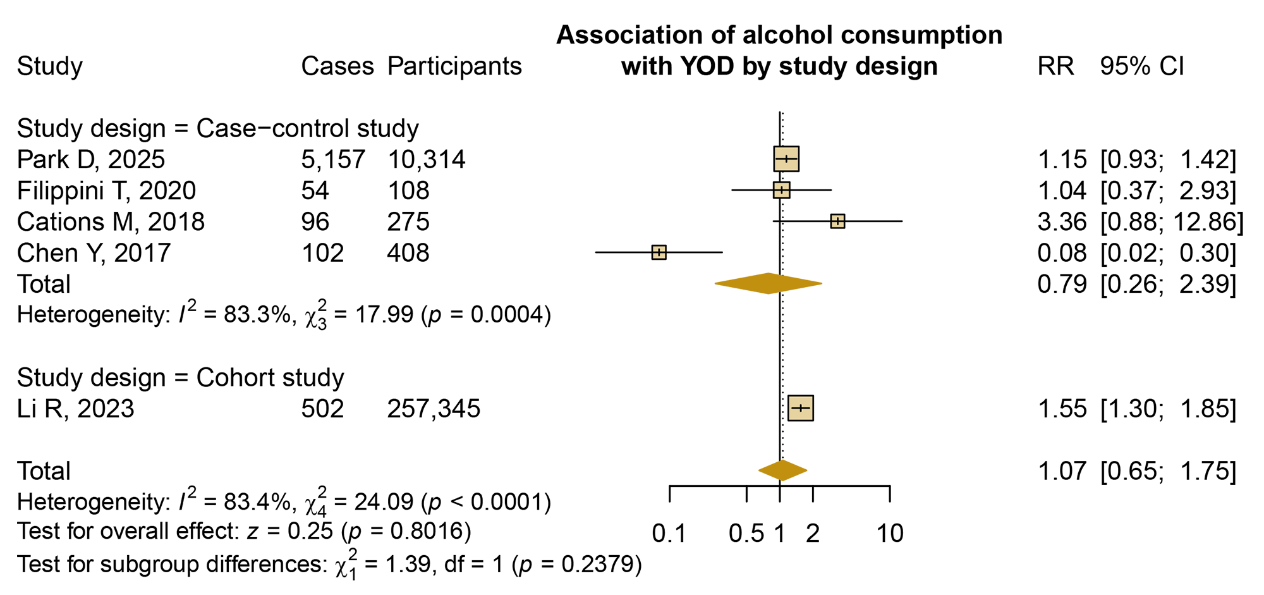


# Supplementary Figure 10. Subgroup analysis of alcohol consumption and young-onset dementia risk by study design


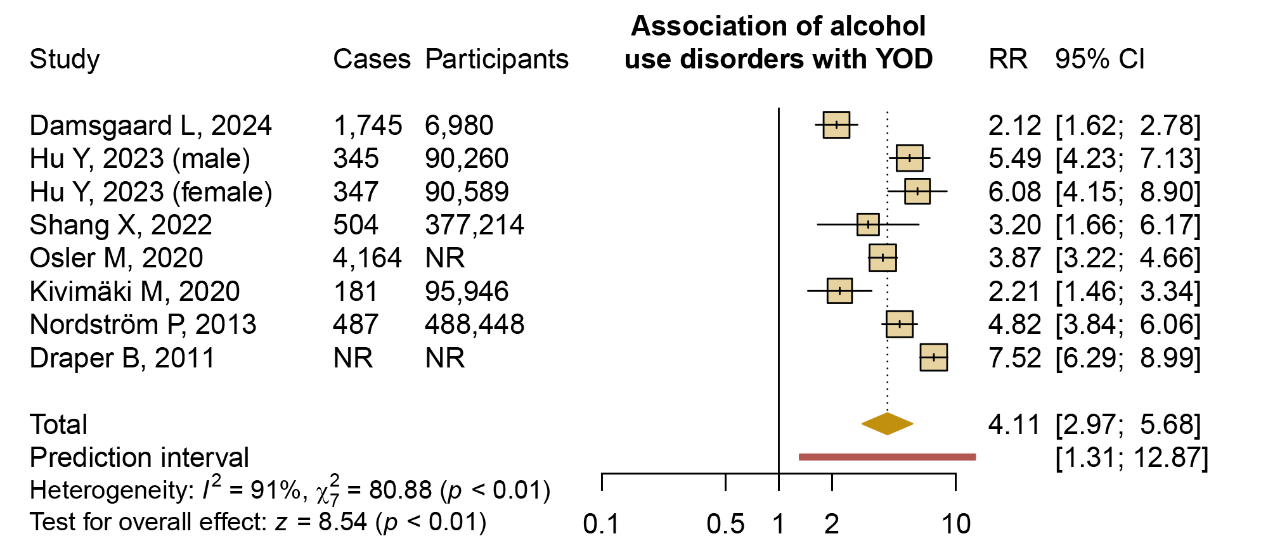


# Supplementary Figure 11. Forest plot of the association between alcohol use disorders and risk of young-onset dementia


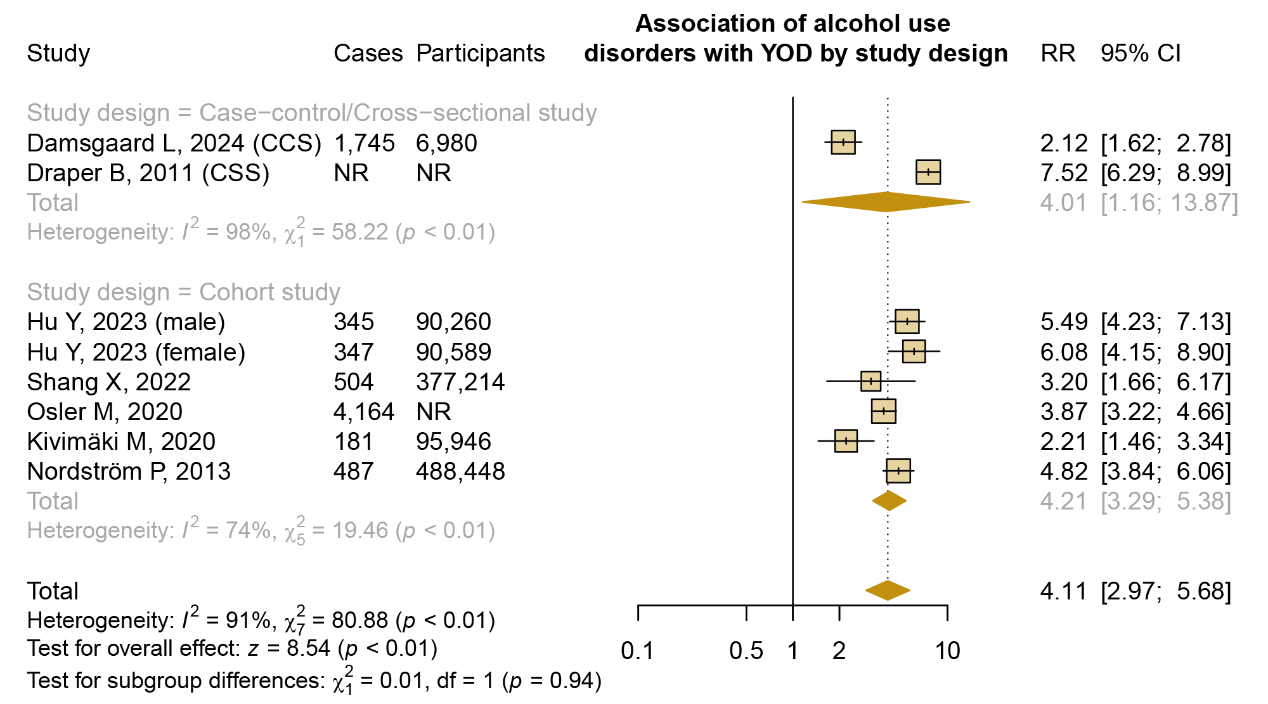


# Supplementary Figure 12. Subgroup analysis of alcohol use disorders and young-onset dementia risk by study design


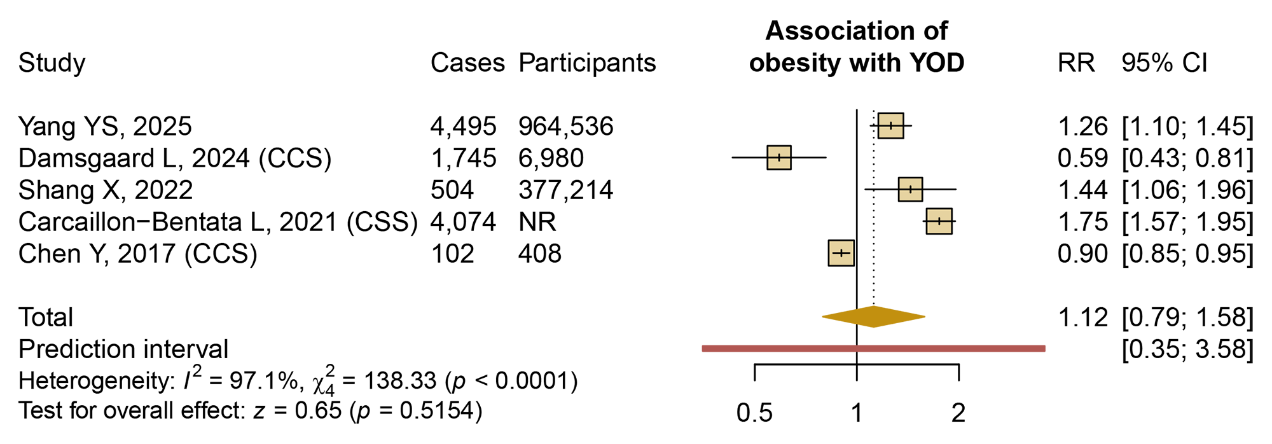


# Supplementary Figure 13. Forest plot of the association between obesity and risk of young-onset dementia


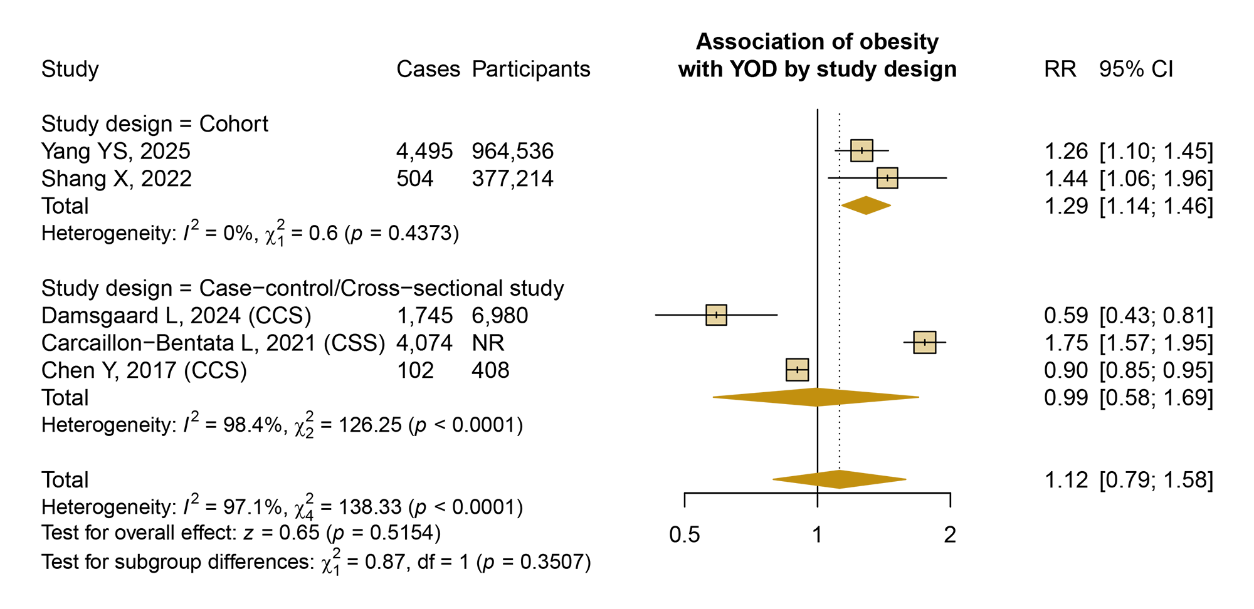


# Supplementary Figure 14. Subgroup analysis of obesity and young-onset dementia risk by study design


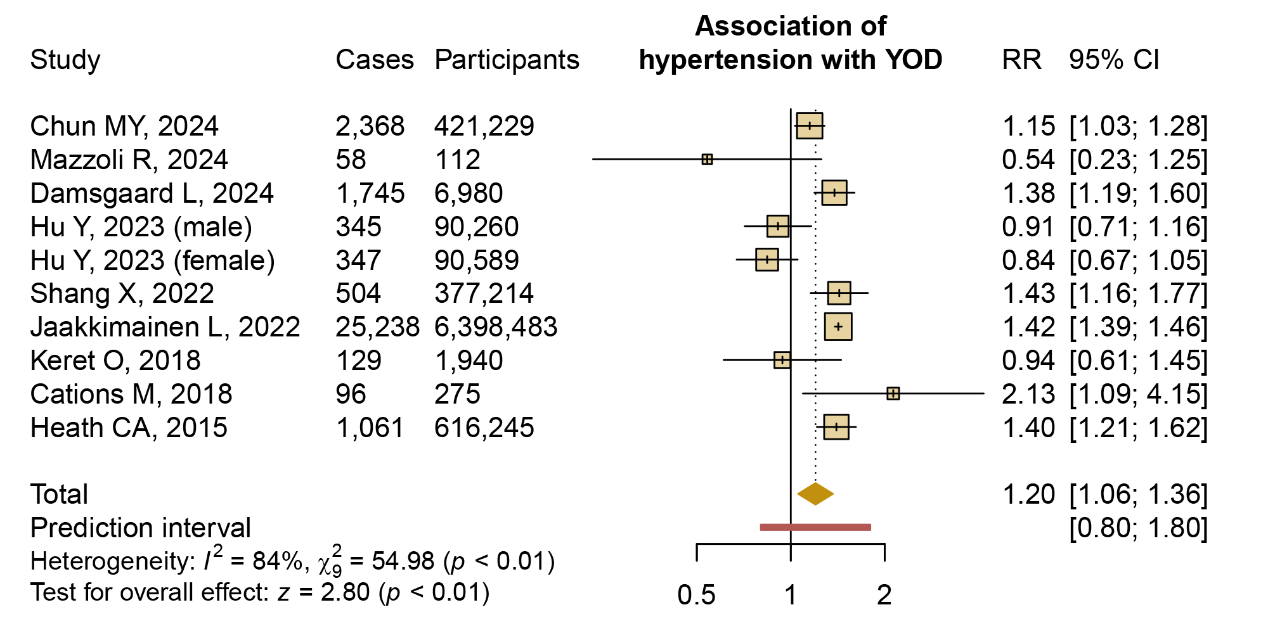


# Supplementary Figure 15. Forest plot of the association between hypertension and risk of young-onset dementia


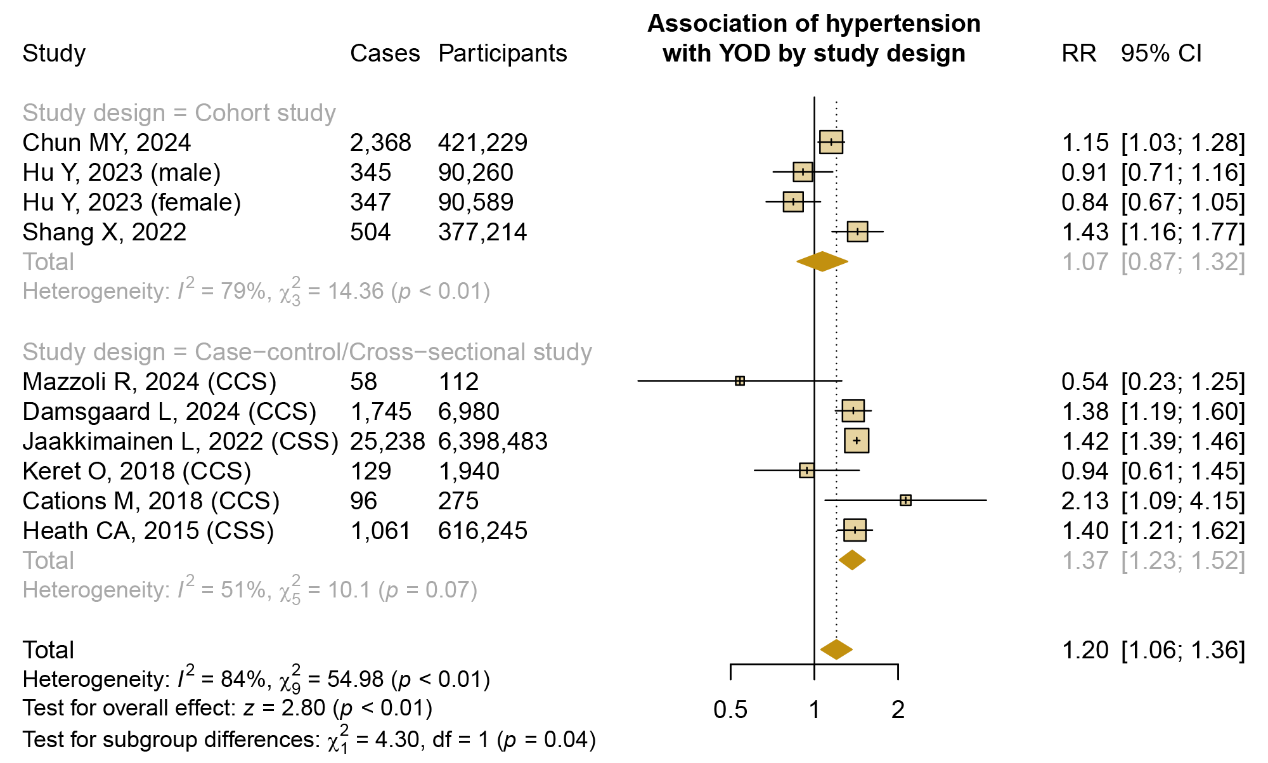


# Supplementary Figure 16. Subgroup analysis of hypertension and young-onset dementia risk by study design


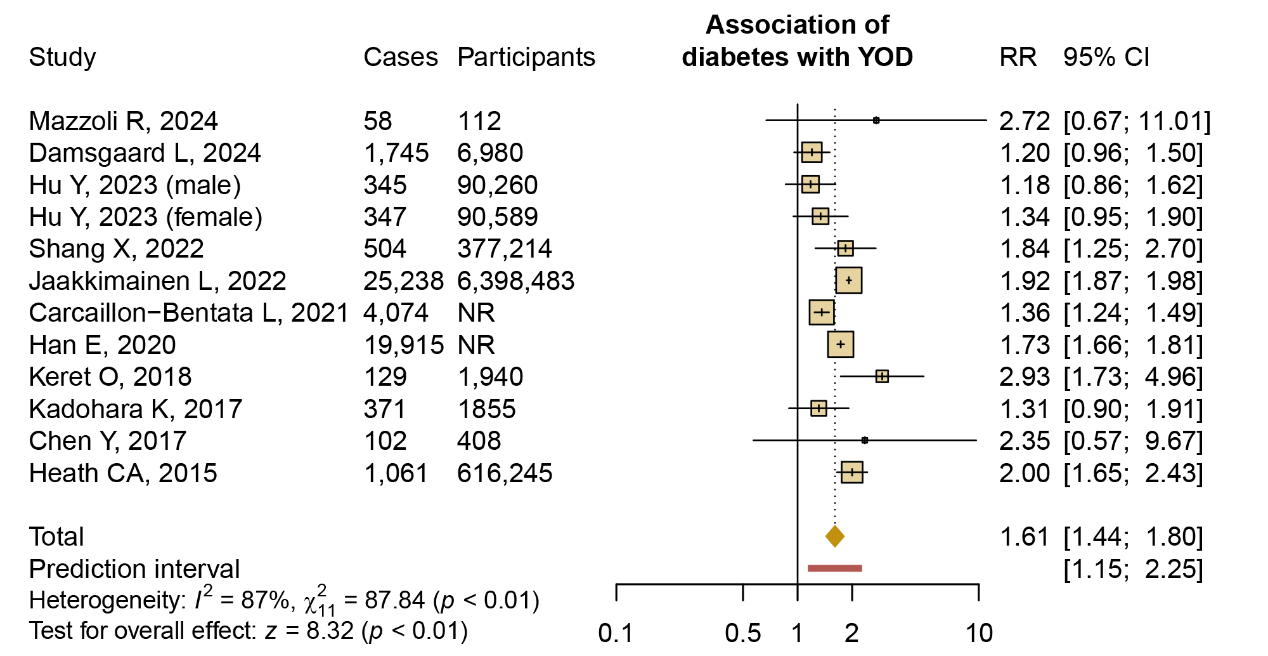


# Supplementary Figure 17. Forest plot of the association between diabetes and risk of young-onset dementia


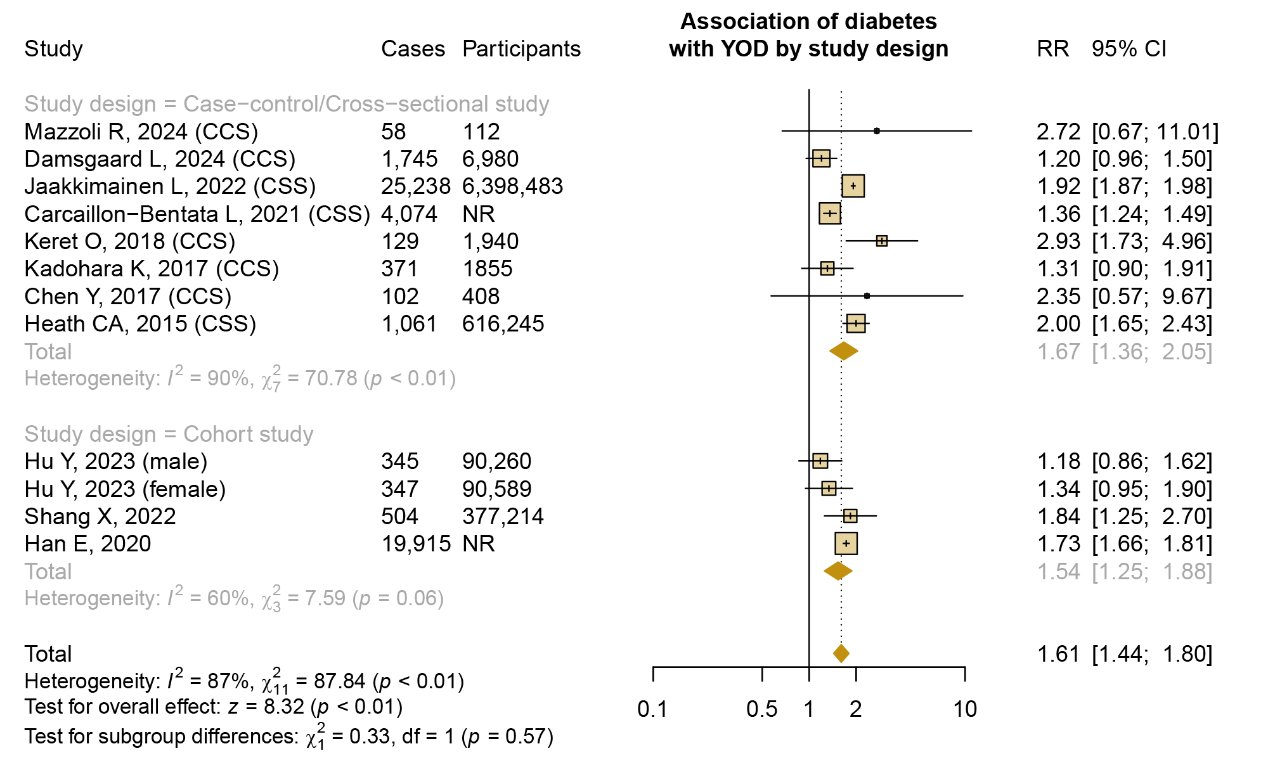


# Supplementary Figure 18. Subgroup analysis of diabetes and young-onset dementia risk by study design


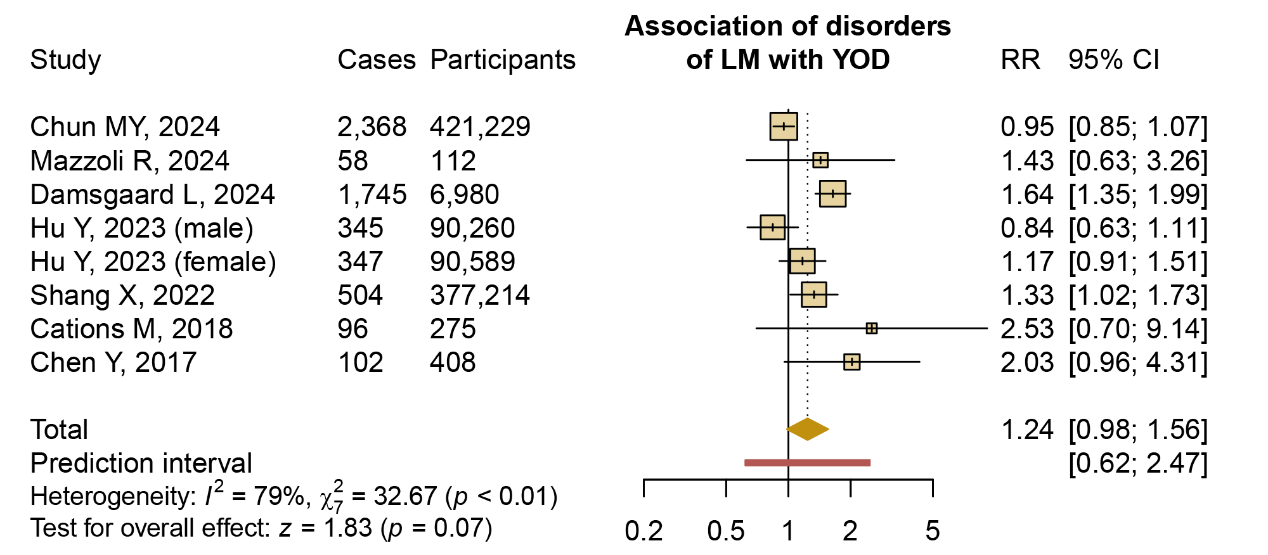


# Supplementary Figure 19. Forest plot of the association between disorders of lipoprotein metabolism and risk of young-onset dementia


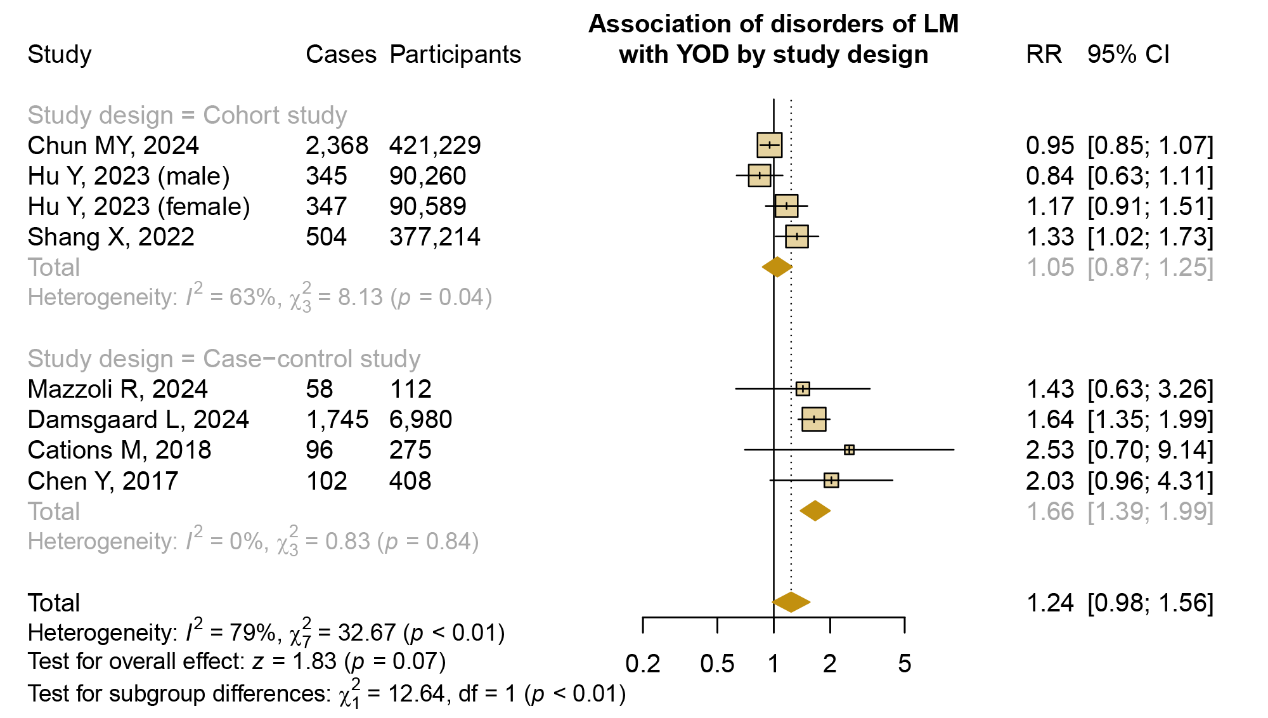


# Supplementary Figure 20. Subgroup analysis of disorders of lipoprotein metabolism and young-onset dementia risk by study design


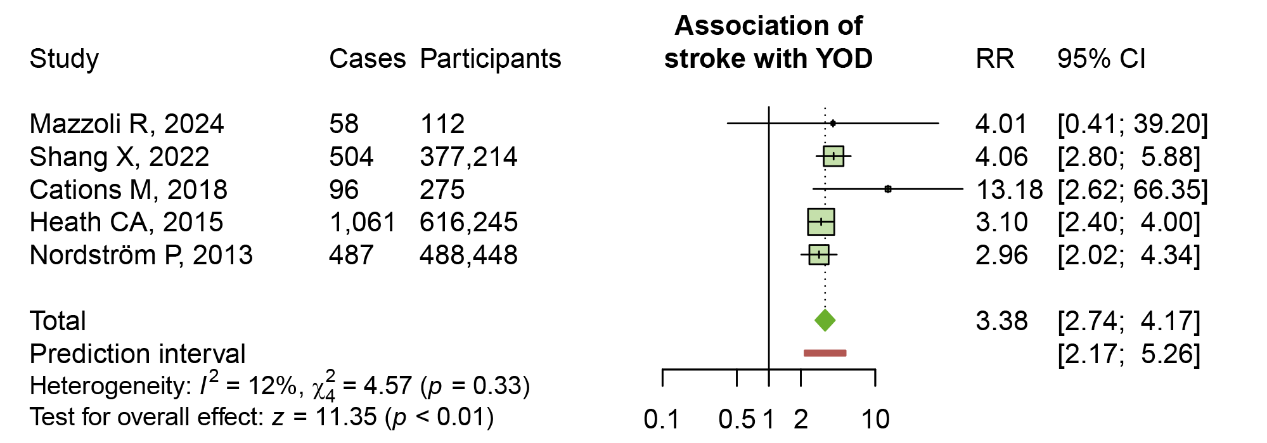


# Supplementary Figure 21. Forest plot of the association between stroke and risk of young-onset dementia


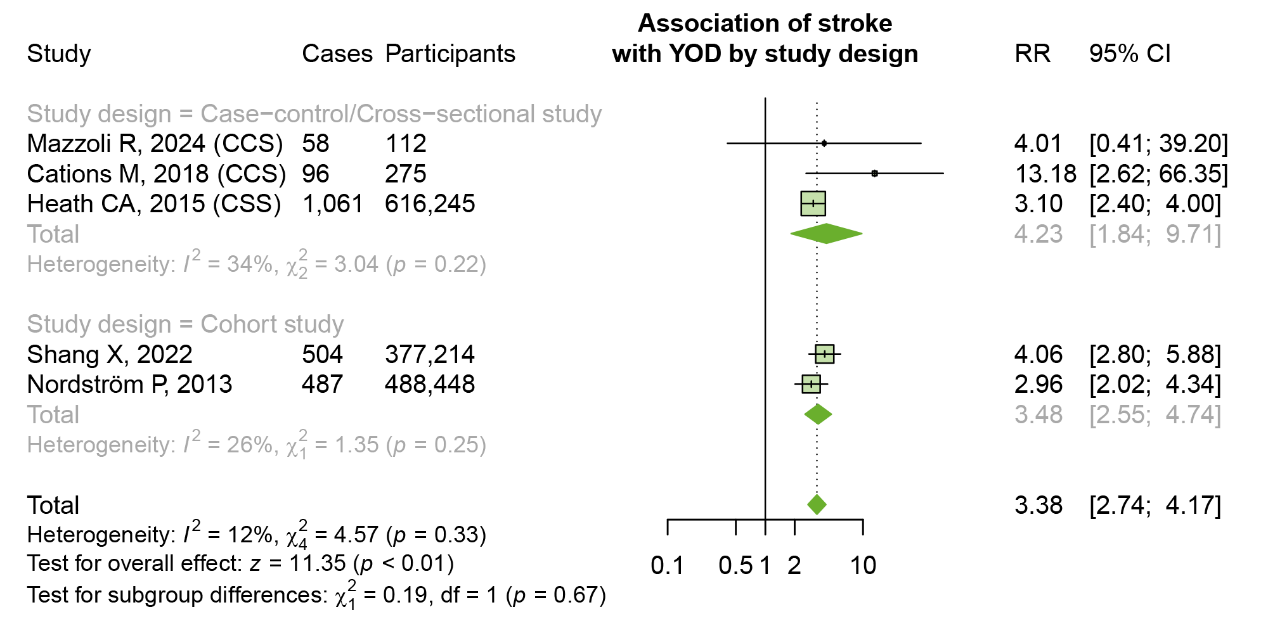


# Supplementary Figure 22. Subgroup analysis of stroke and young-onset dementia risk by study design


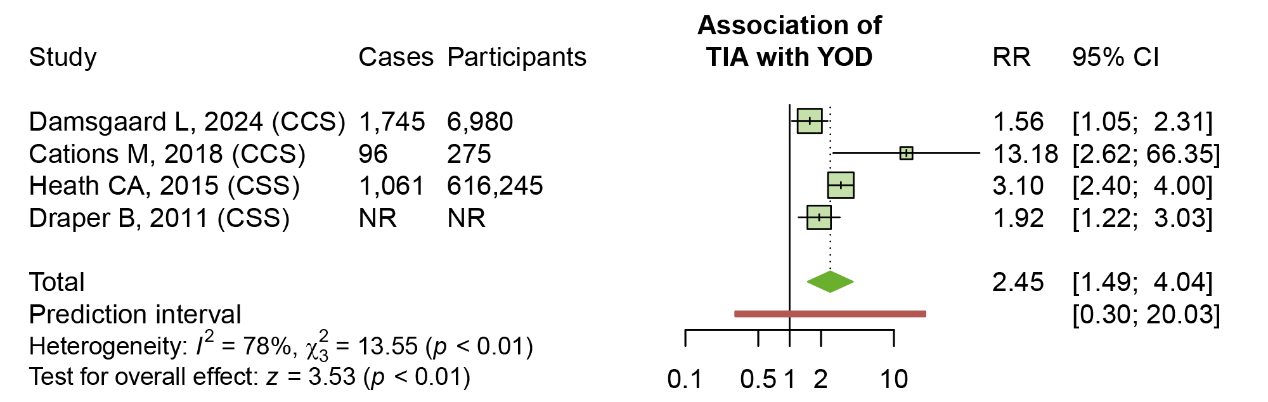


# Supplementary Figure 23. Forest plot of the association between TIA and risk of young-onset dementia


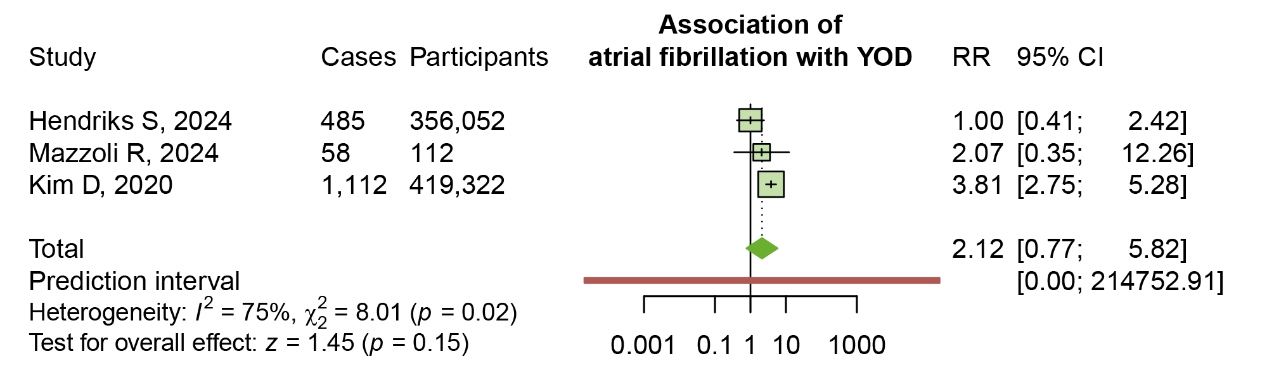


# Supplementary Figure 24. Forest plot of the association between atrial fibrillation and risk of young-onset dementia


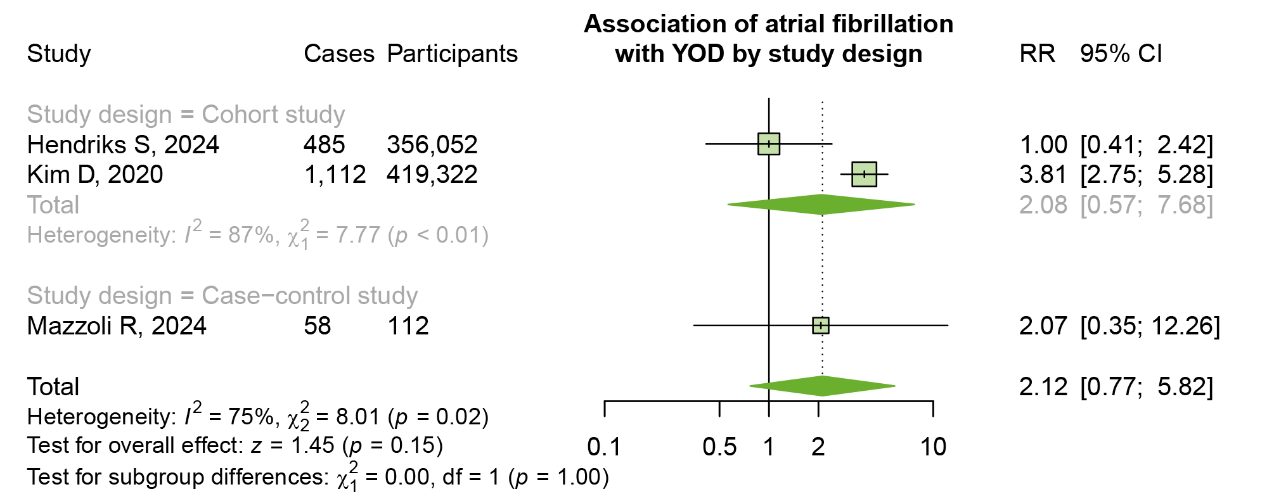


# Supplementary Figure 25. Subgroup analysis of atrial fibrillation and young-onset dementia risk by study design


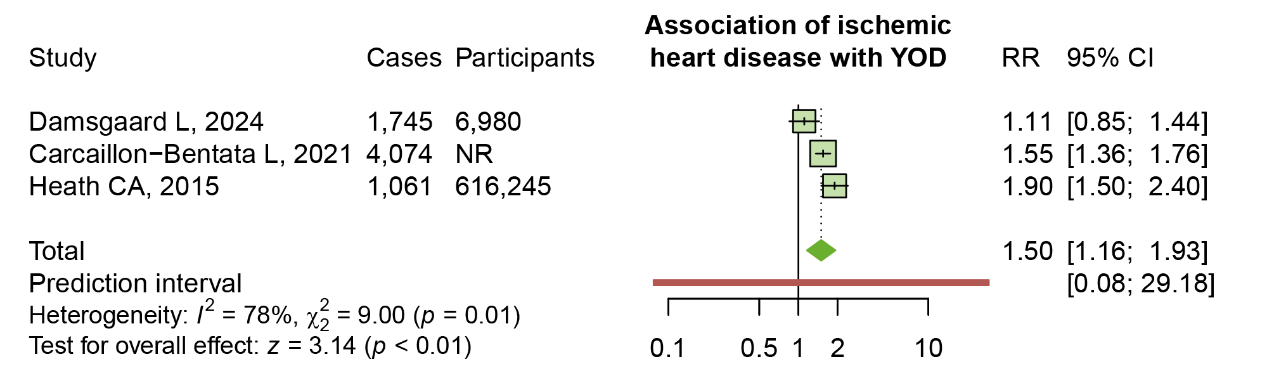


# Supplementary Figure 26. Forest plot of the association between ischemic heart disease and risk of young-onset dementia


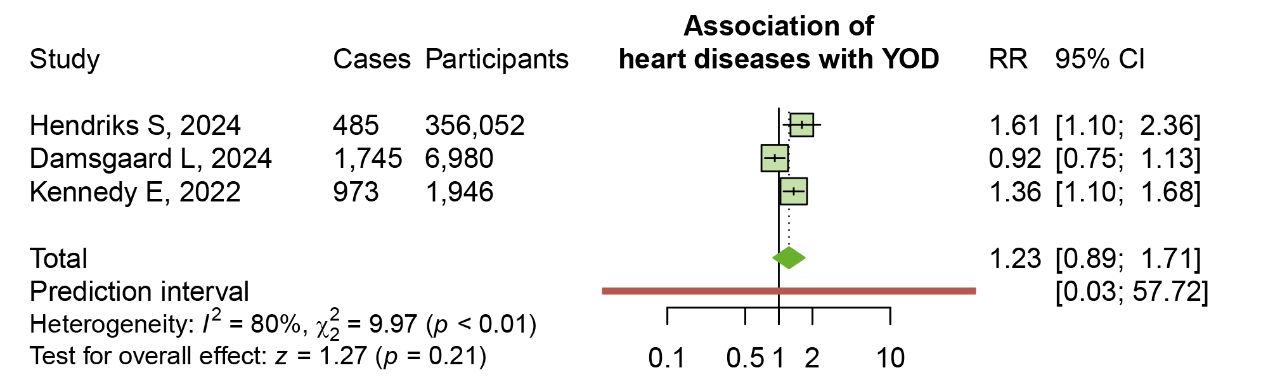


# Supplementary Figure 27. Forest plot of the association between heart diseases and risk of young-onset dementia


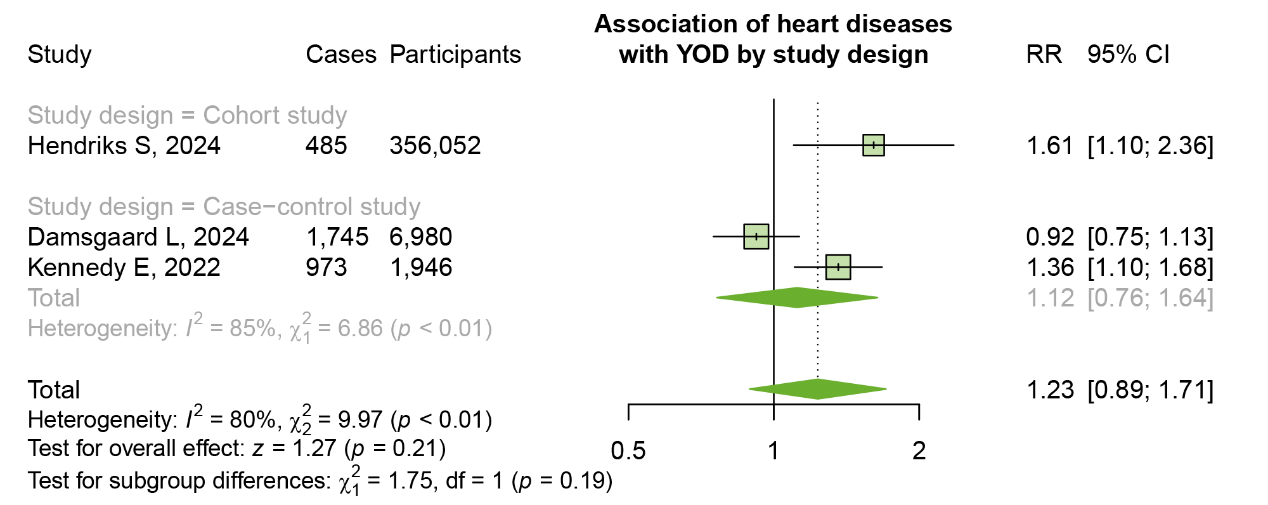


# Supplementary Figure 28. Subgroup analysis of heart diseases and young-onset dementia risk by study design


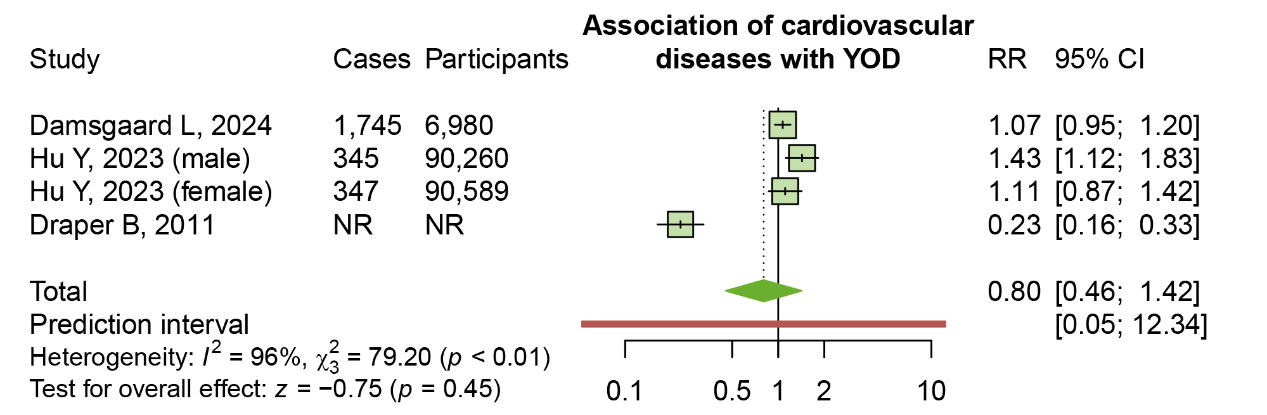


# Supplementary Figure 29. Forest plot of the association between cardiovascular diseases and risk of young-onset dementia


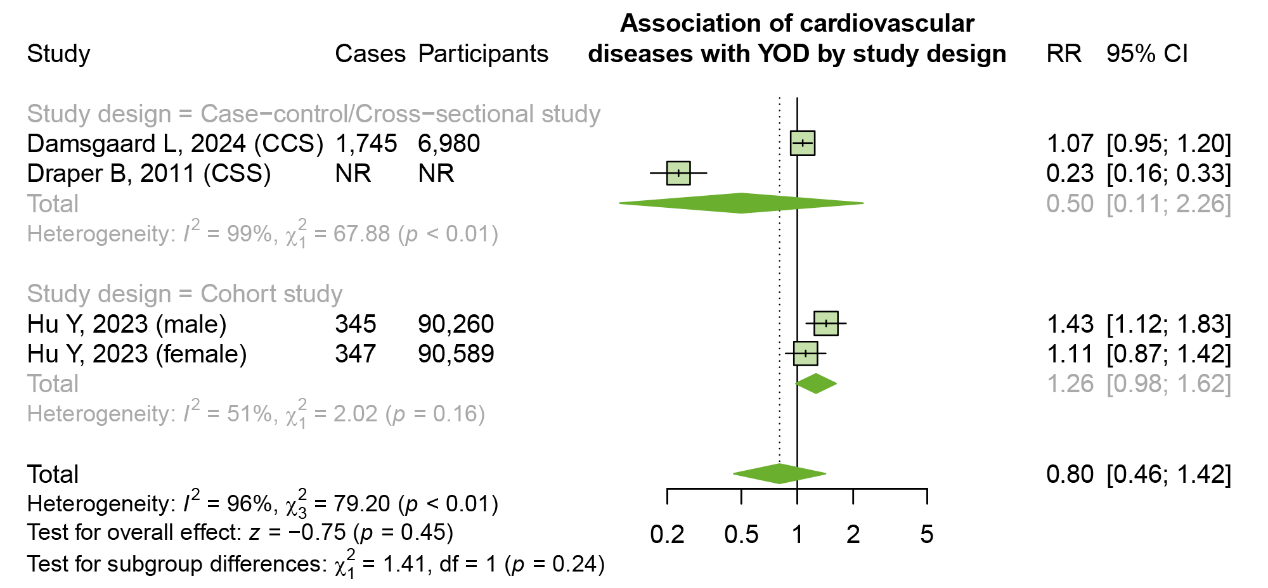


# Supplementary Figure 30. Subgroup analysis of cardiovascular diseases and young-onset dementia risk by study design


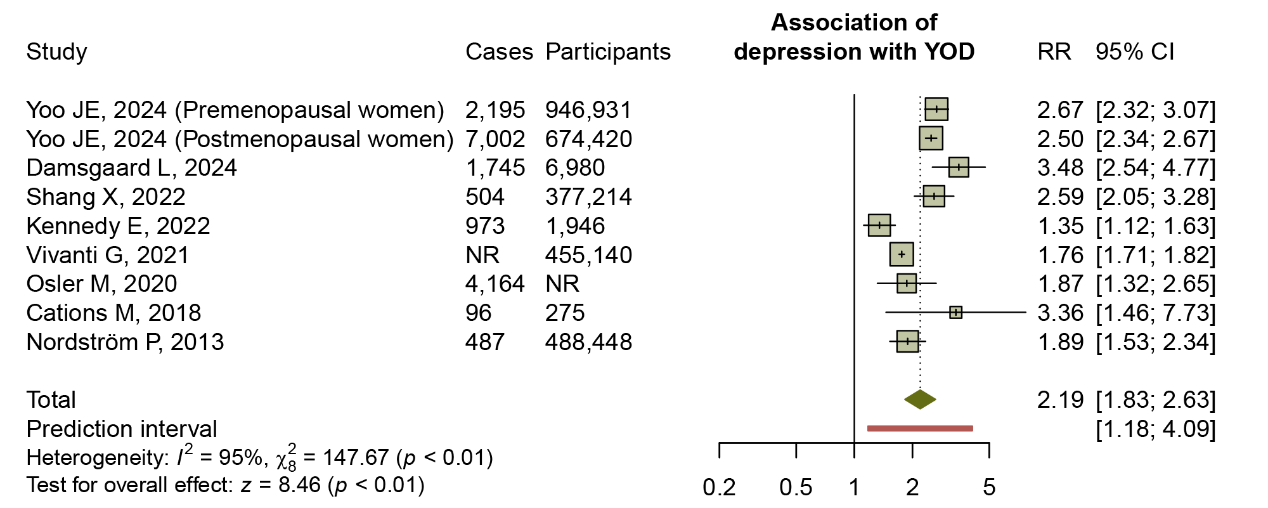


# Supplementary Figure 31. Forest plot of the association between depression and risk of young-onset dementia


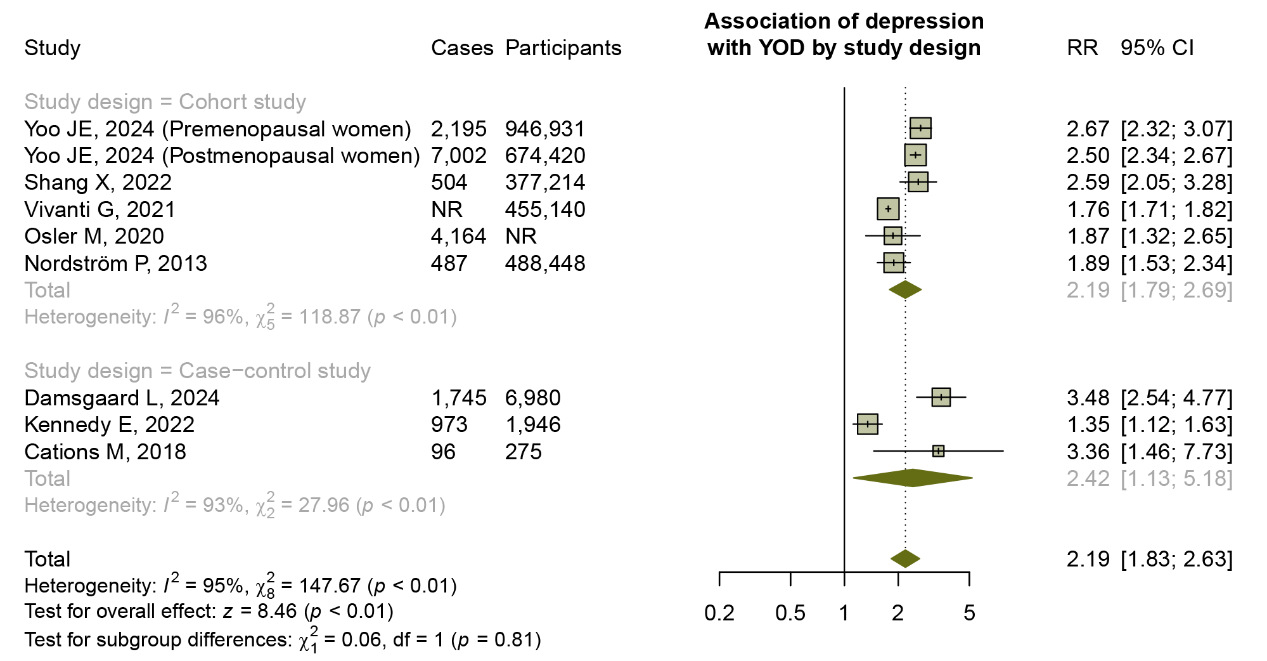


# Supplementary Figure 32. Subgroup analysis of depression and young-onset dementia risk by study design


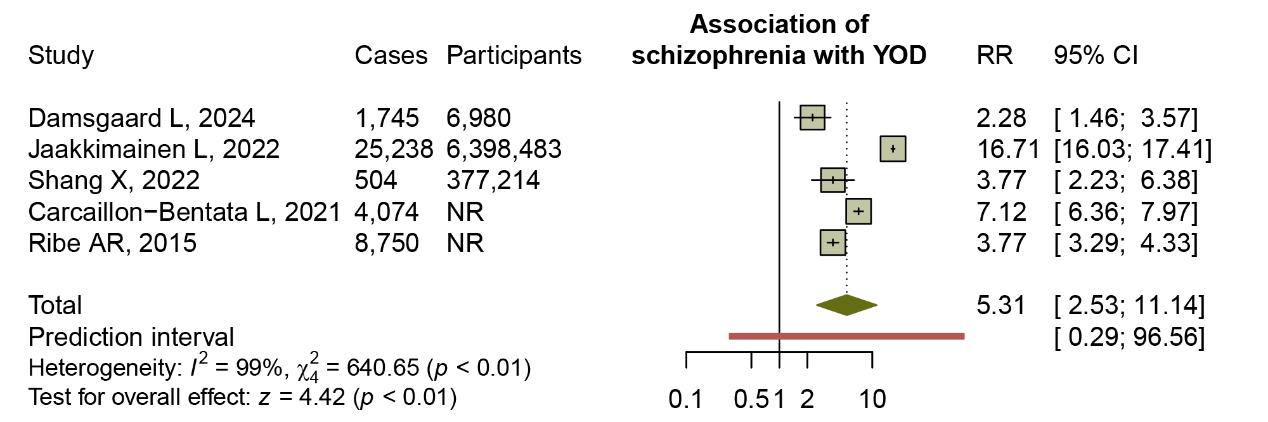


# Supplementary Figure 33. Forest plot of the association between schizophrenia and risk of young-onset dementia


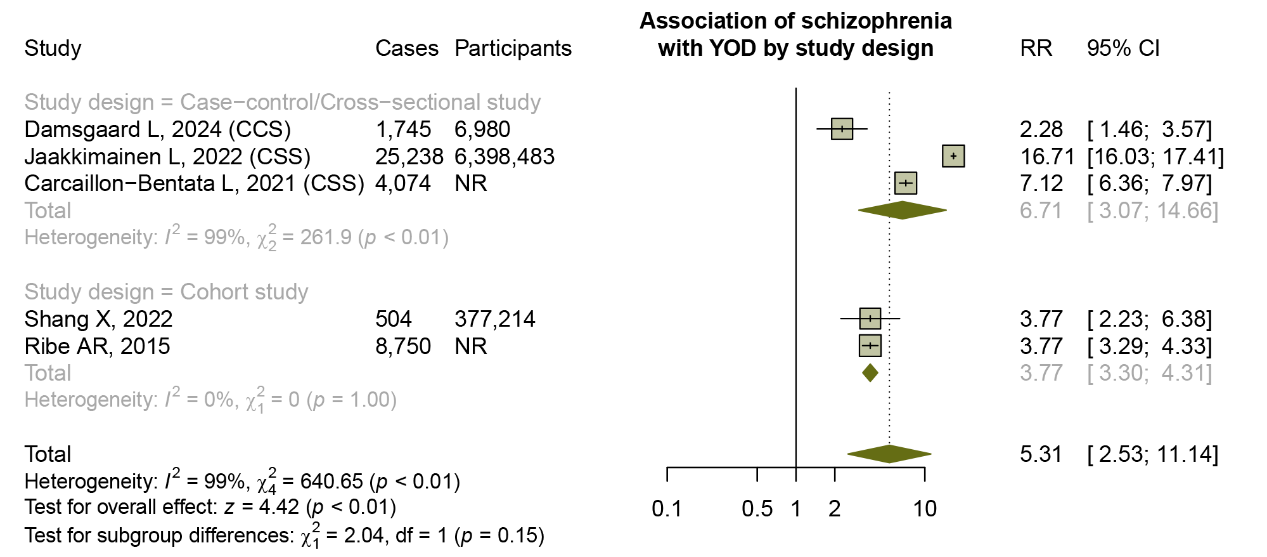


# Supplementary Figure 34. Subgroup analysis of schizophrenia and young-onset dementia risk by study design


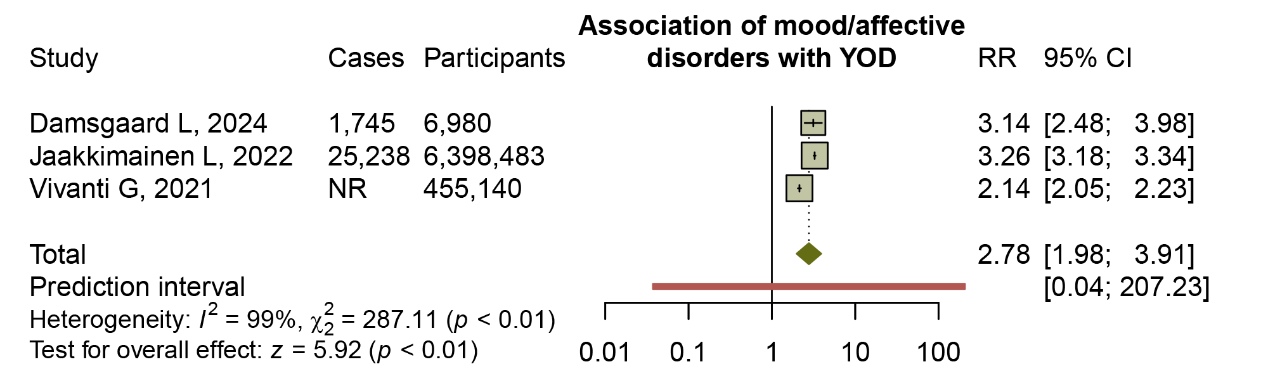


# Supplementary Figure 35. Forest plot of the association between mood/affective disorders and risk of young-onset dementia


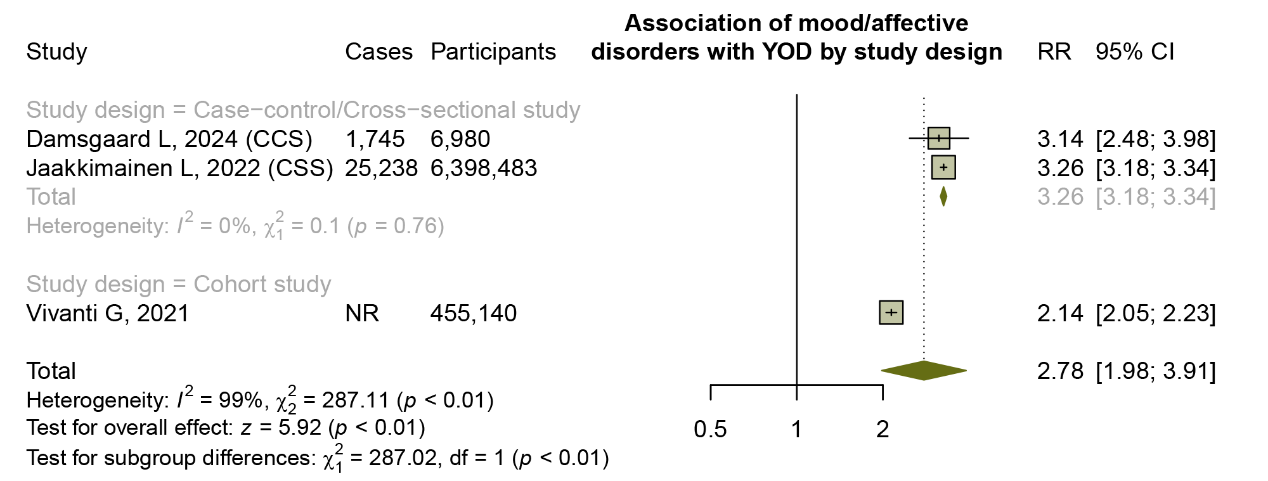


# Supplementary Figure 36. Subgroup analysis of mood/affective disorders and young-onset dementia risk by study design


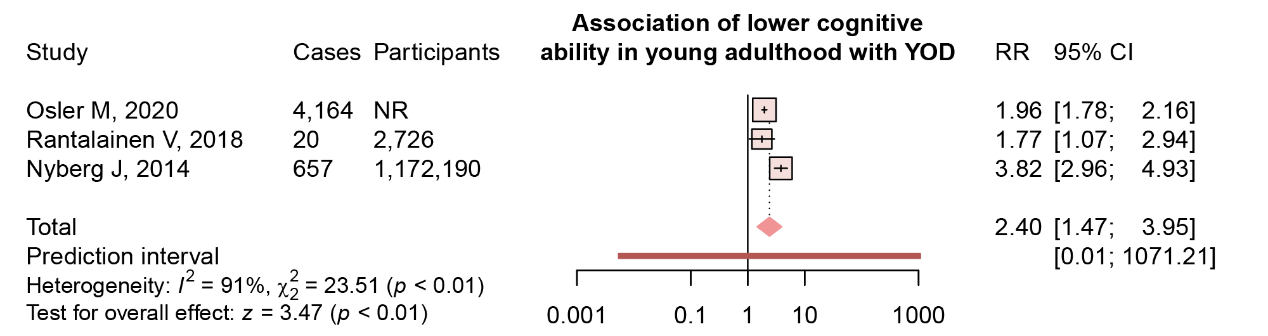


# Supplementary Figure 37. Forest plot of the association between lower cognitive ability in young adulthood and risk of young-onset dementia


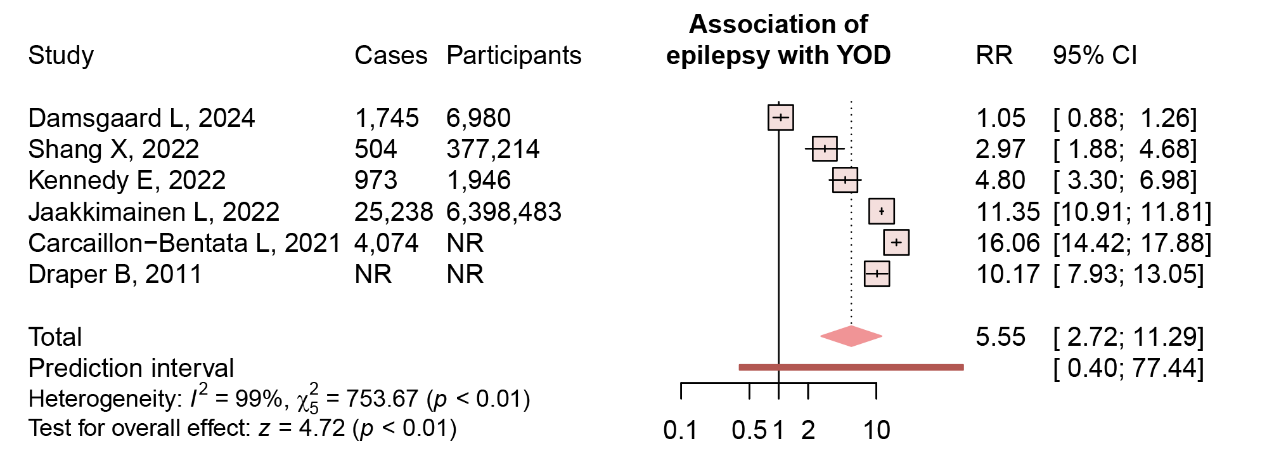


# Supplementary Figure 38. Forest plot of the association between epilepsy and risk of young-onset dementia


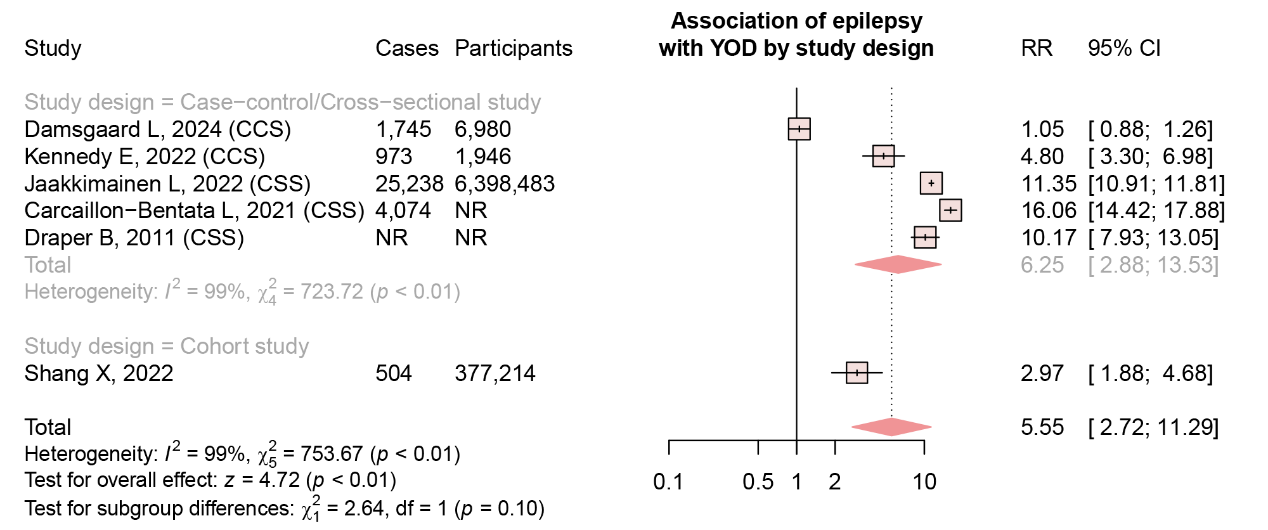


# Supplementary Figure 39. Subgroup analysis of epilepsy and young-onset dementia risk by study design


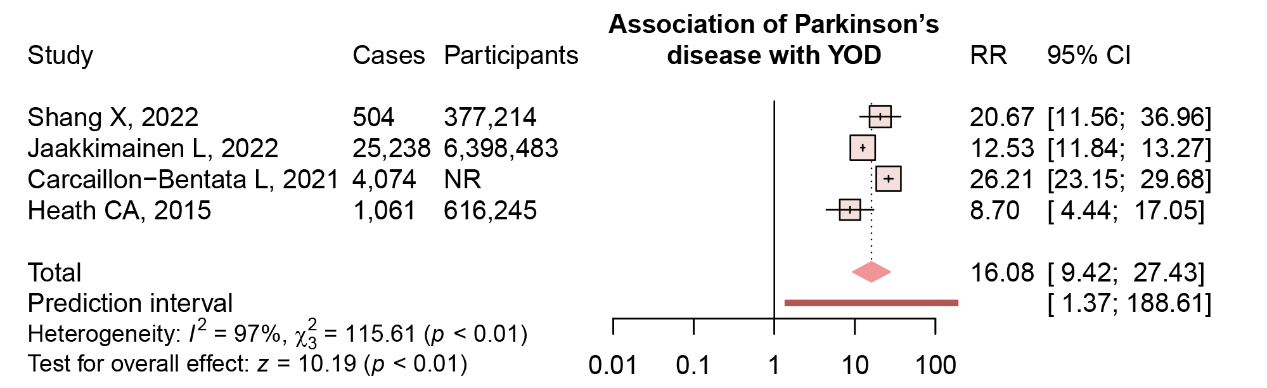


# Supplementary Figure 40. Forest plot of the association between Parkinson’s disease and risk of young-onset dementia


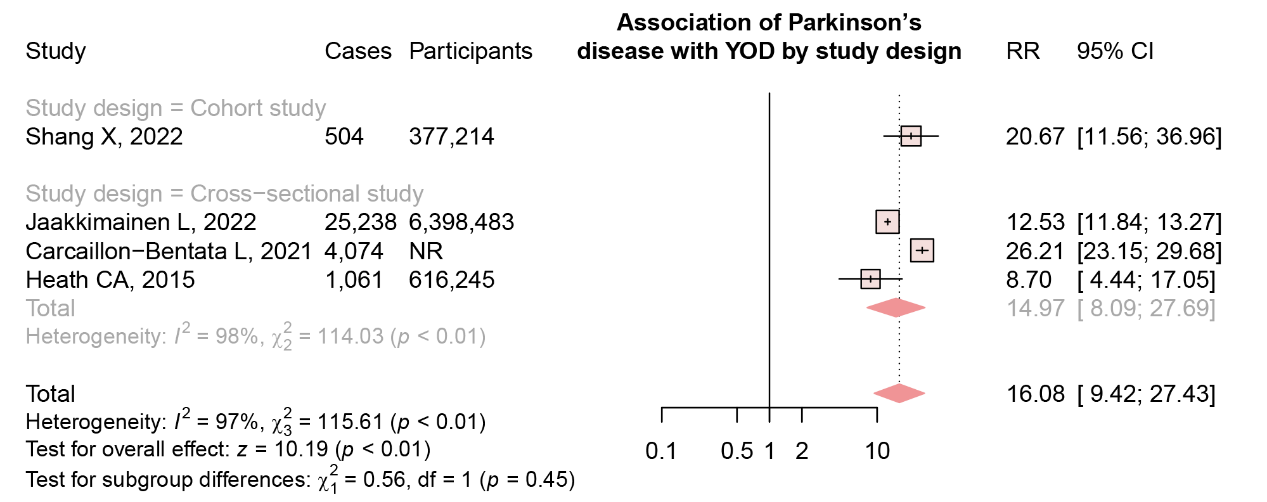


# Supplementary Figure 41. Subgroup analysis of Parkinson’s disease and young-onset dementia risk by study design


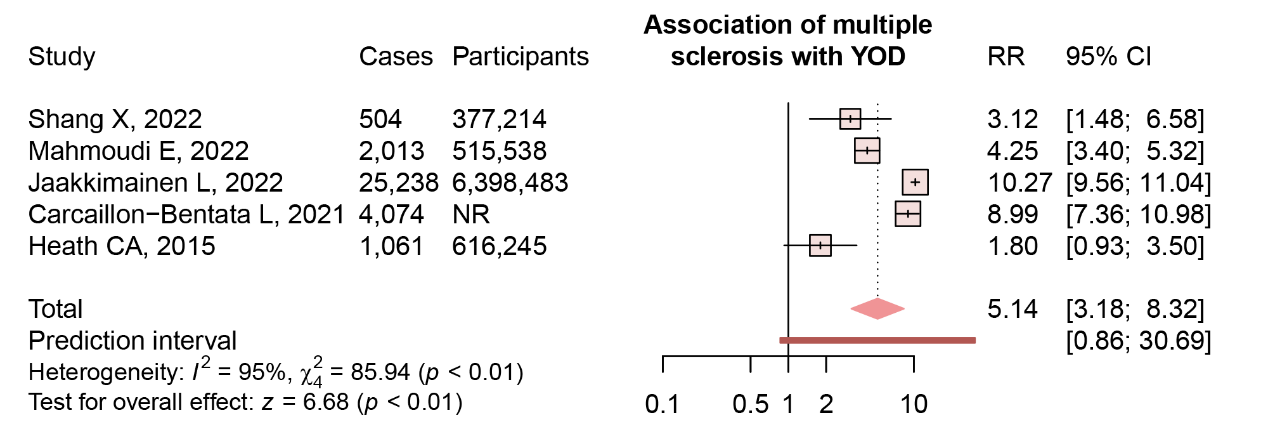


# Supplementary Figure 42. Forest plot of the association between multiple sclerosis and risk of young-onset dementia


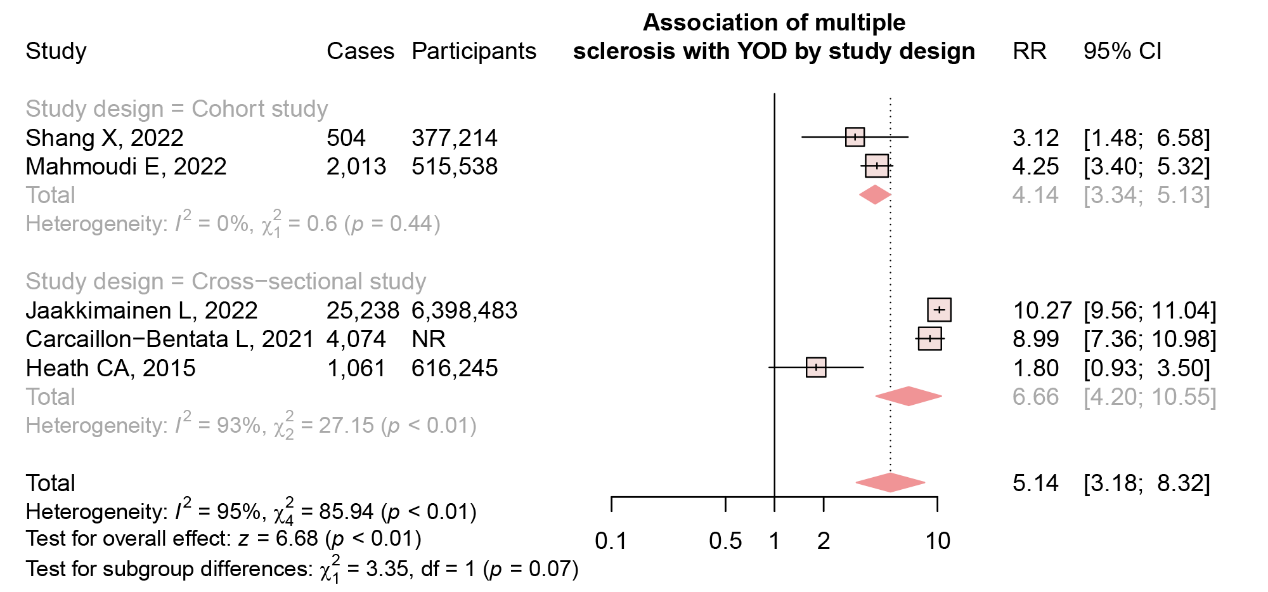


# Supplementary Figure 43. Subgroup analysis of multiple sclerosis and young-onset dementia risk by study design


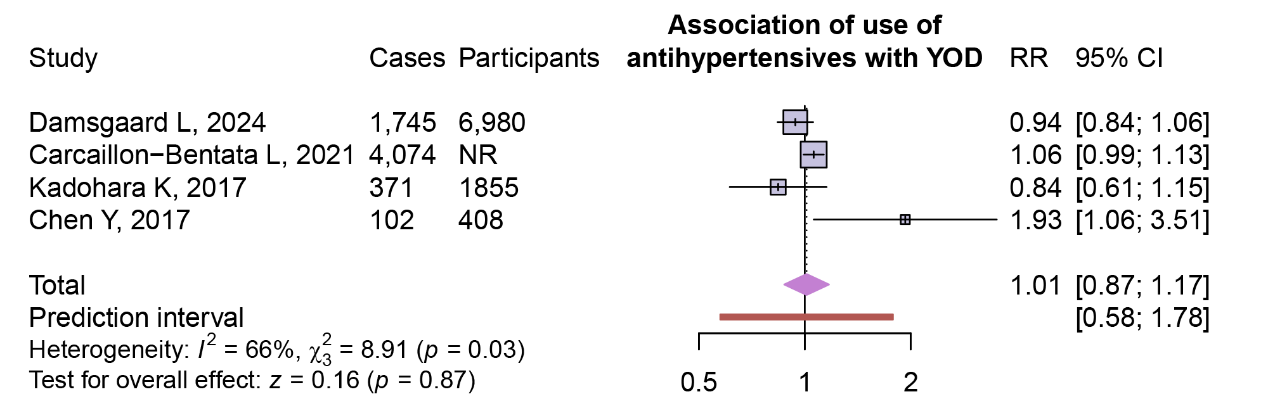


# Supplementary Figure 44. Forest plot of the association between use of antihypertensives and risk of young-onset dementia


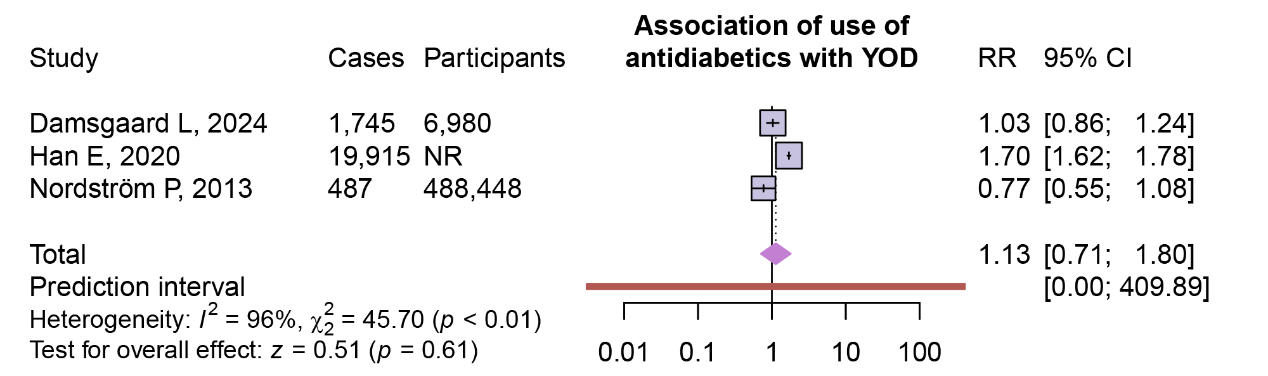


# Supplementary Figure 45. Forest plot of the association between use of antidiabetics and risk of young-onset dementia


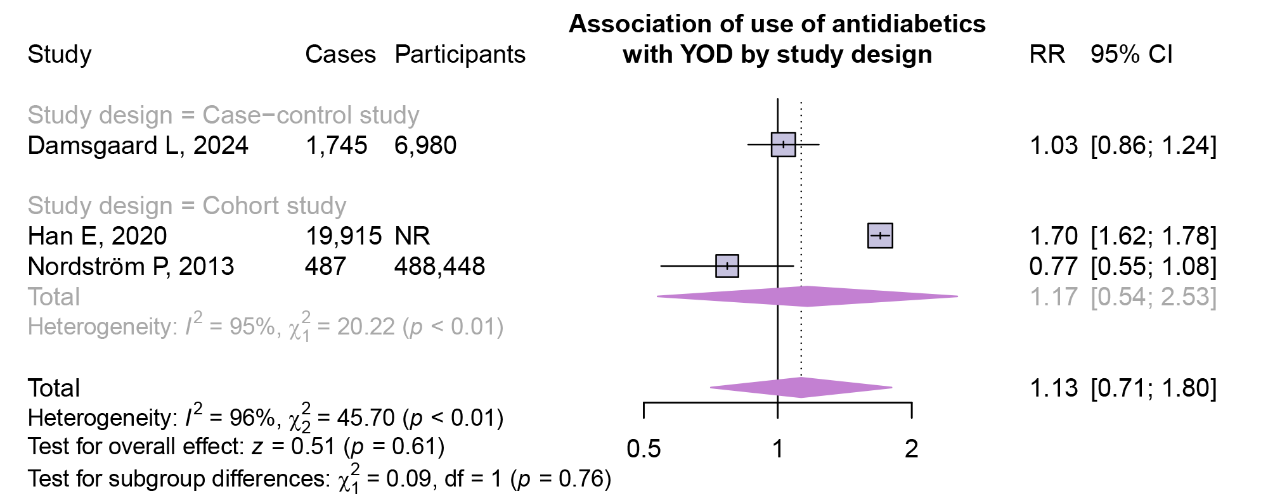


# Supplementary Figure 46. Subgroup analysis of use of antidiabetics and young-onset dementia risk by study design


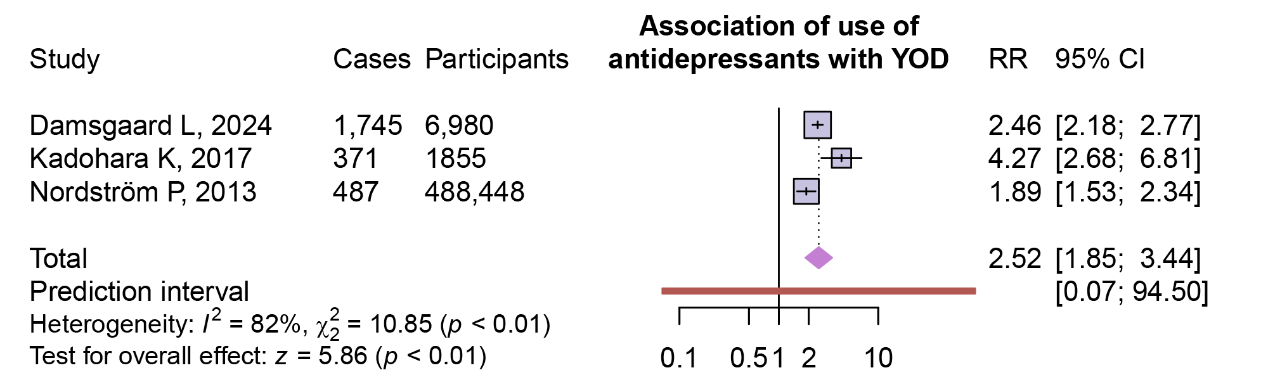


# Supplementary Figure 47. Forest plot of the association between use of antidepressants and risk of young-onset dementia


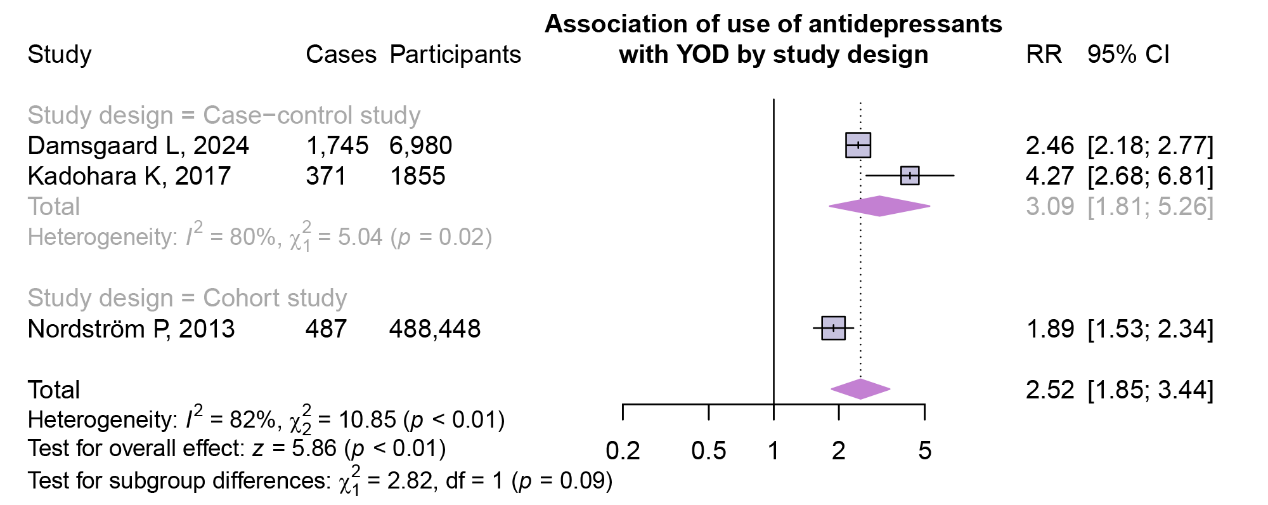


# Supplementary Figure 48. Subgroup analysis of use of antidepressants and young-onset dementia risk by study design


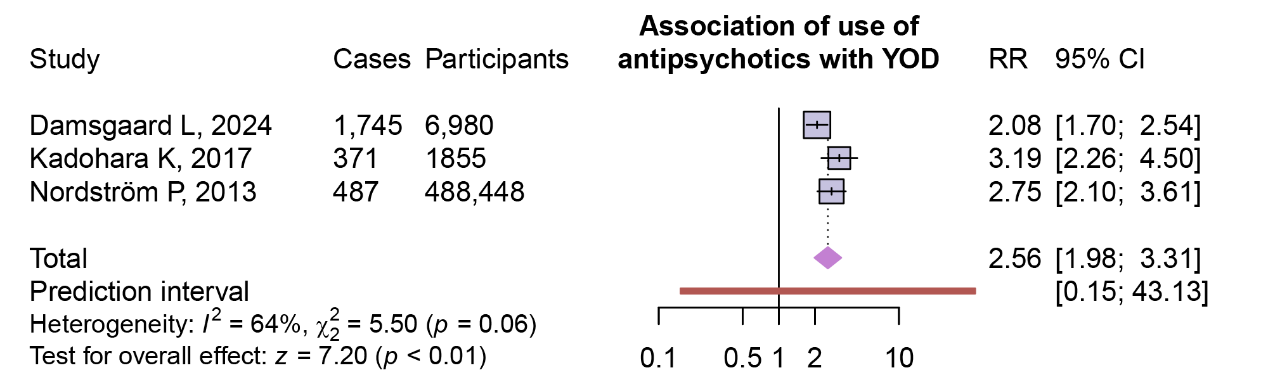


# Supplementary Figure 49. Forest plot of the association between use of antipsychotics and risk of young-onset dementia


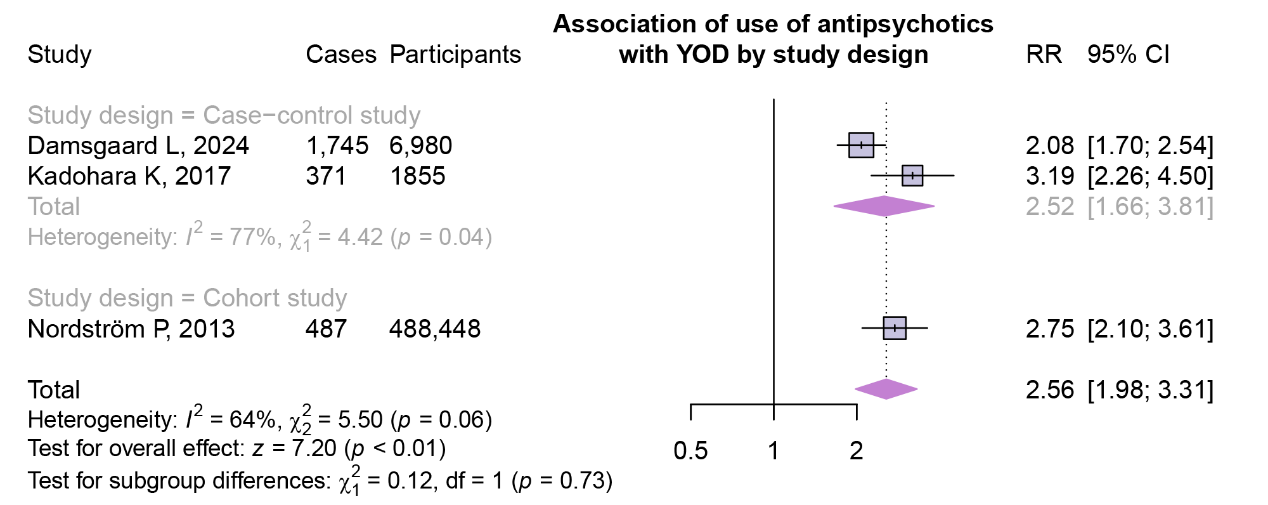


# Supplementary Figure 50. Subgroup analysis of use of antipsychotics and young-onset dementia risk by study design


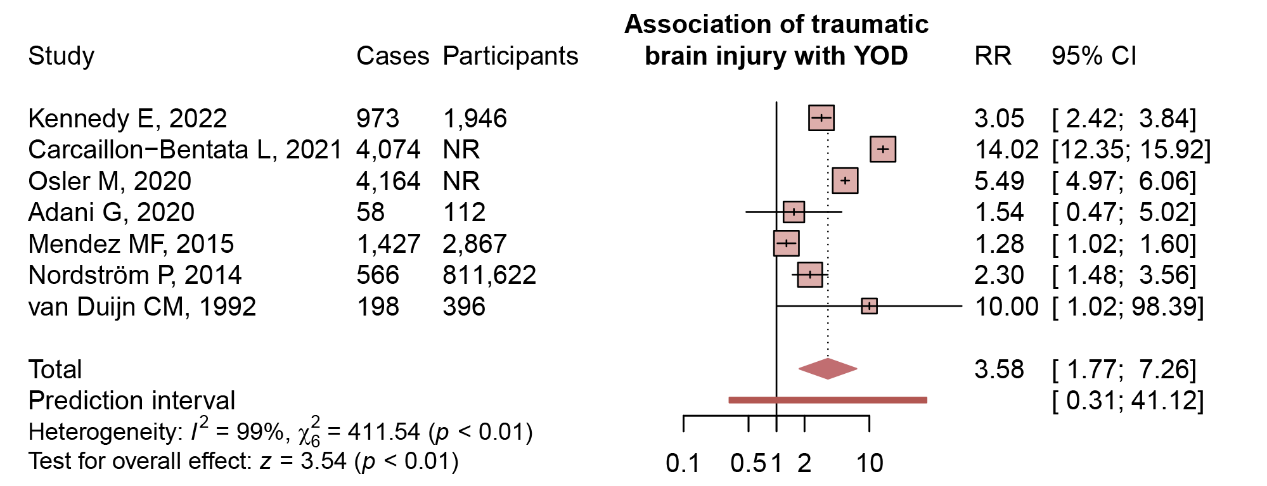


# Supplementary Figure 51. Forest plot of the association between traumatic brain injury and risk of young-onset dementia


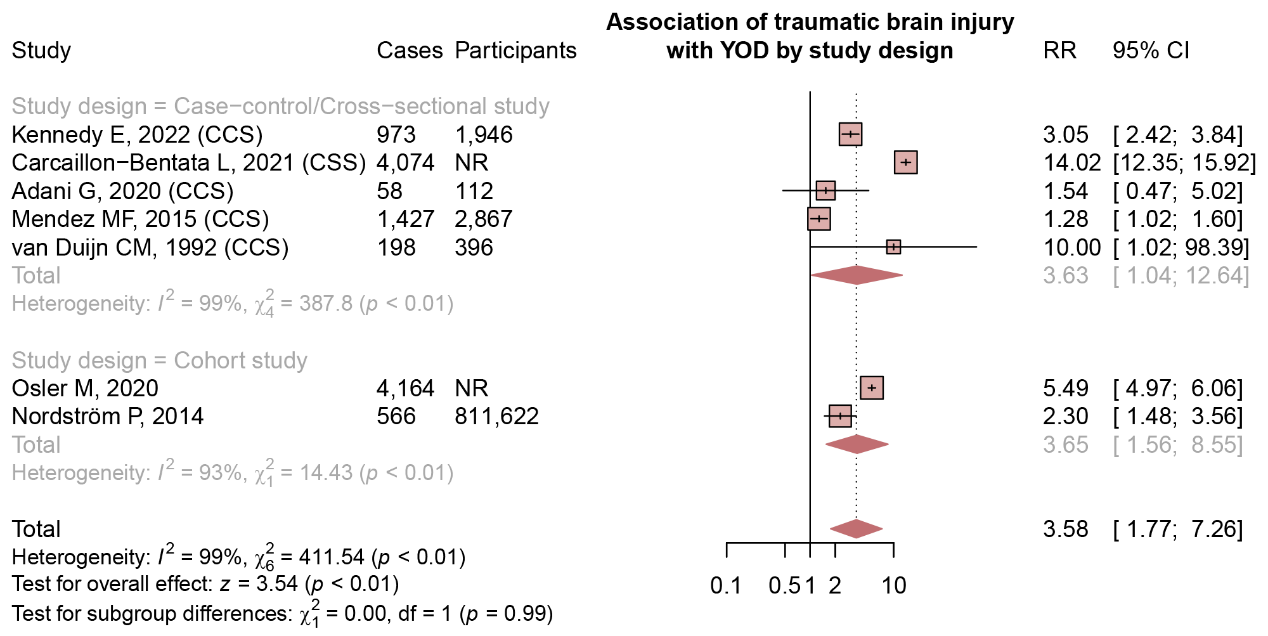


# Supplementary Figure 52. Subgroup analysis of traumatic brain injury and young-onset dementia risk by study design


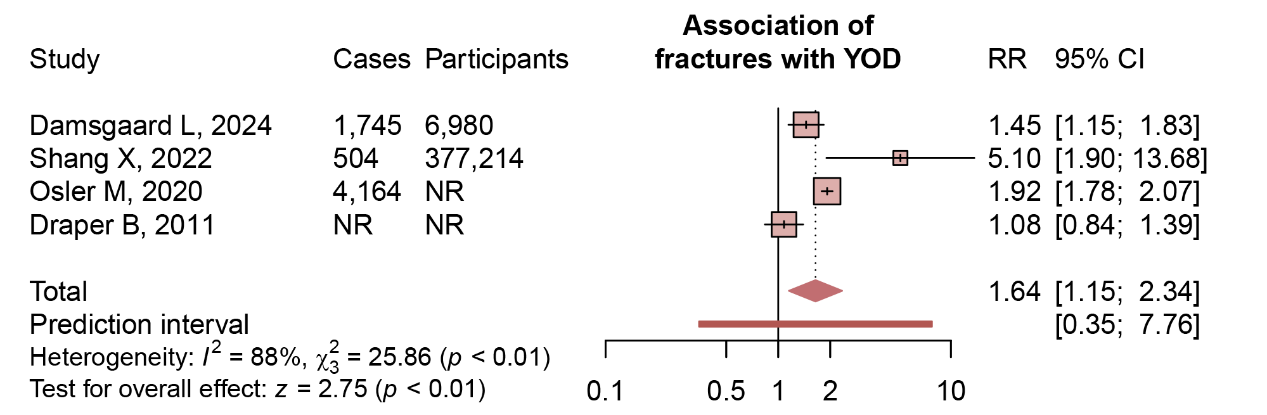


# Supplementary Figure 53. Forest plot of the association between fractures and risk of young-onset dementia


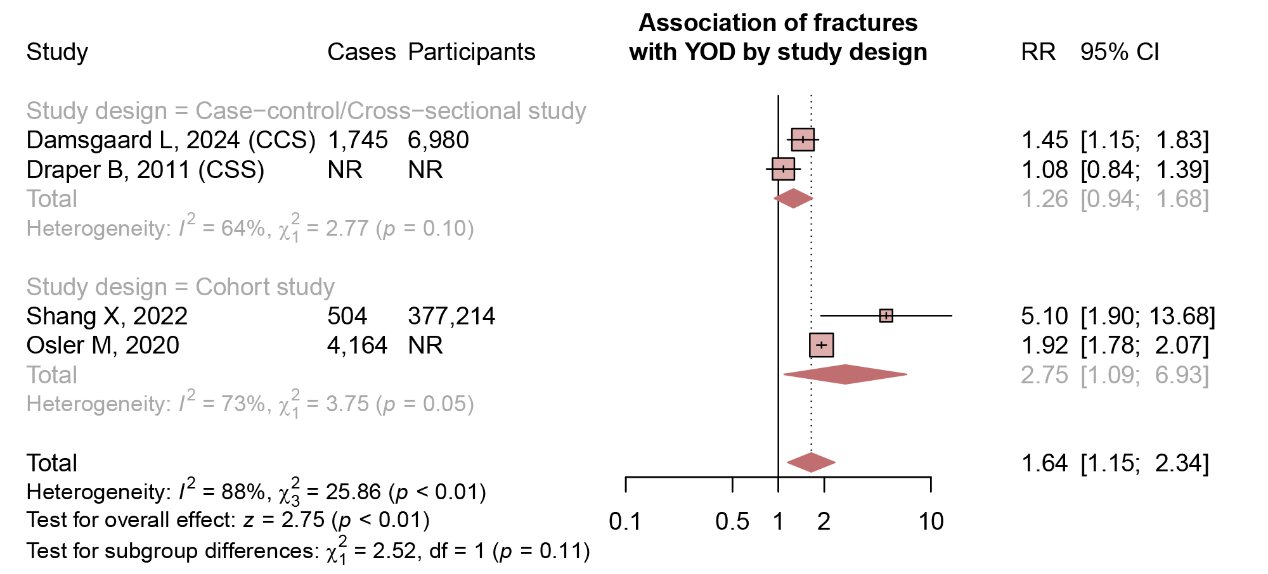


# Supplementary Figure 54. Subgroup analysis of fractures and young-onset dementia risk by study design


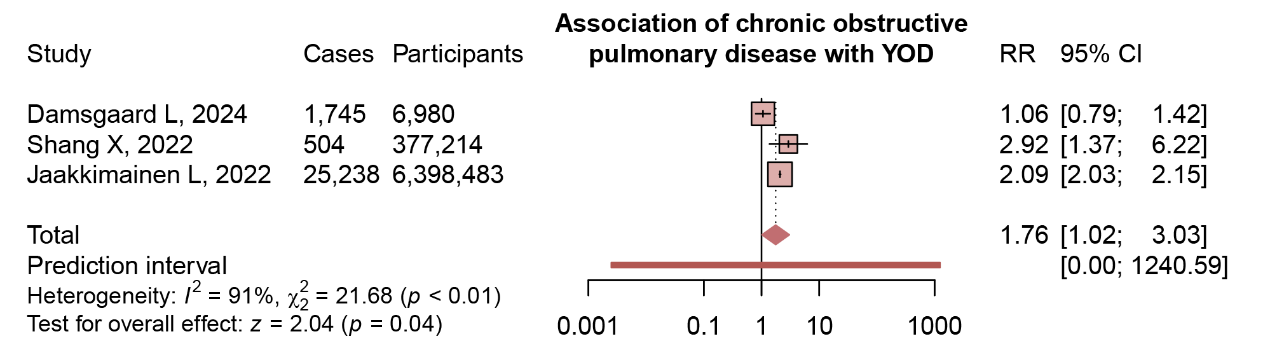


# Supplementary Figure 55. Forest plot of the association between chronic obstructive pulmonary disease and risk of young-onset dementia


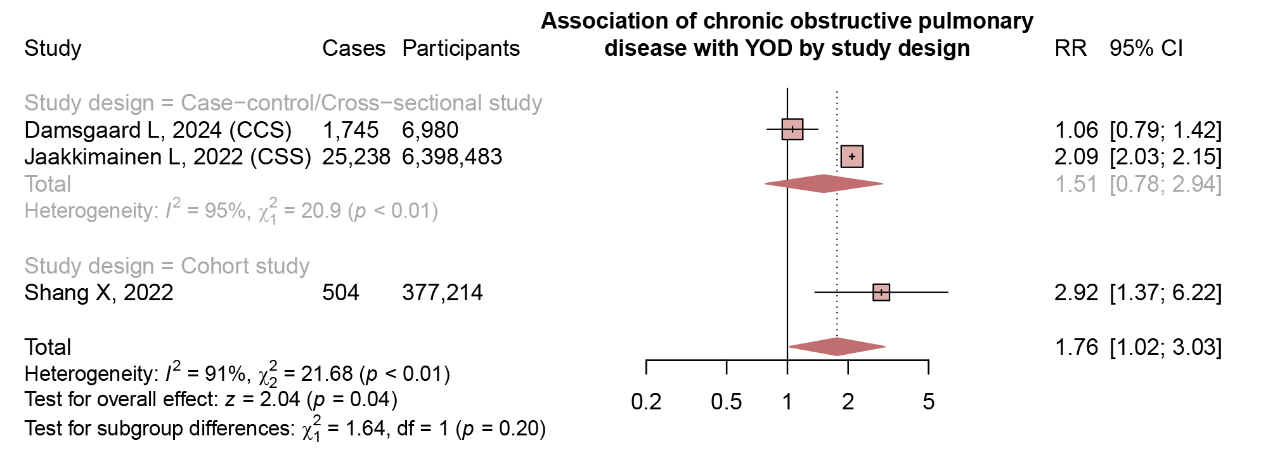


# Supplementary Figure 56. Subgroup analysis of chronic obstructive pulmonary disease and young-onset dementia risk by study design


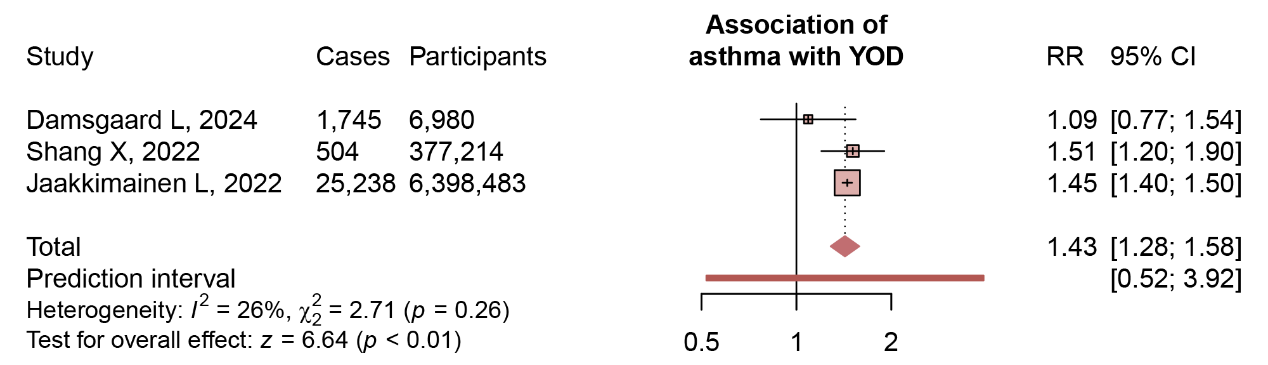


# Supplementary Figure 57. Forest plot of the association between asthma and risk of young-onset dementia


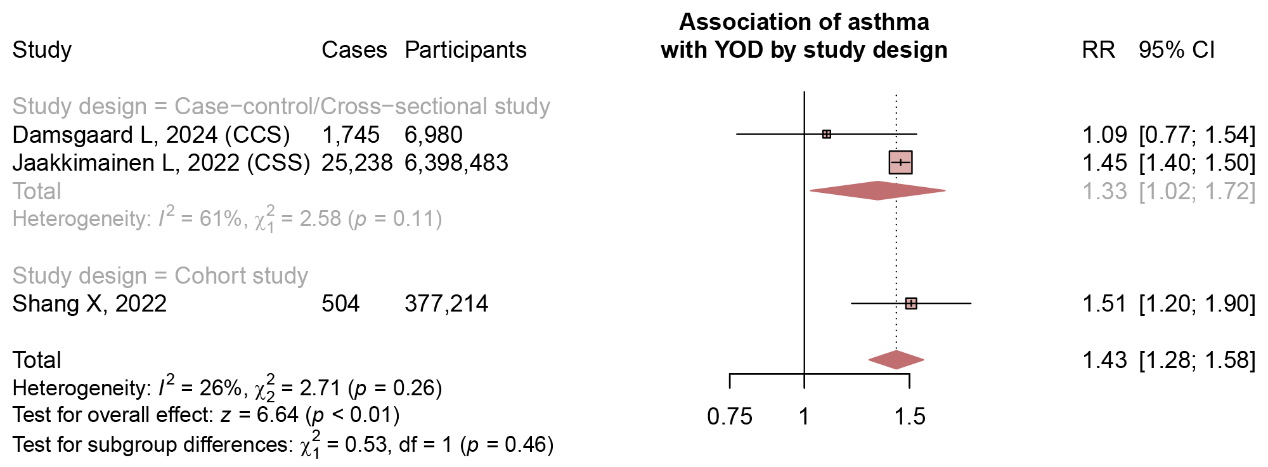


# Supplementary Figure 58. Subgroup analysis of asthma and young-onset dementia risk by study design
